# Treatment of Locally Advanced Rectal Cancer in the Era of Total Neoadjuvant Therapy: A Systematic Review and Network Meta-Analysis

**DOI:** 10.1001/jamanetworkopen.2024.14702

**Published:** 2024-06-04

**Authors:** Giulia Turri, Giovanni Ostuzzi, Giovanni Vita, Valeria Barresi, Aldo Scarpa, Michele Milella, Renzo Mazzarotto, Andrea Ruzzenente, Corrado Barbui, Corrado Pedrazzani

**Affiliations:** 1 Division of General and Hepatobiliary Surgery, Department of Surgical Sciences, Dentistry, Gynecology and Pediatrics, University of Verona, Verona, Italy; 2 World Health Organization Collaborating Centre for Research and Training in Mental Health and Service Evaluation, Department of Neuroscience, Biomedicine and Movement Sciences, Section of Psychiatry, University of Verona, Verona, Italy; 3 Department of Diagnostics and Public Health, Section of Pathology, University of Verona, Italy; 4 Section of Oncology, Department of Engineering for Innovation Medicine, University of Verona Hospital Trust, Verona, Italy; 5 Section of Radiotherapy, Department of Medicine, University of Verona Hospital Trust, Verona, Italy; 6 Division of General and Hepatobiliary Surgery, Department of Engineering for Innovation Medicine, University of Verona, Verona, Italy

## Abstract

**Importance:**

Treatment of locally advanced rectal cancer (LARC) involves neoadjuvant chemoradiotherapy plus total mesorectal excision and adjuvant chemotherapy. However, total neoadjuvant therapy (TNT) protocols (ie, preoperative chemotherapy in addition to radiotherapy) may allow better adherence and early treatment of distant micrometastases and may increase pathological complete response (pCR) rates.

**Objective:**

To assess the efficacy and tolerability of TNT protocols for LARC.

**Data Sources:**

MEDLINE, Embase, Cochrane Central Register of Controlled Trials (CENTRAL), and Web of Science Core Collection electronic databases and ClinicalTrials.gov for unpublished studies were searched from inception to March 2, 2024.

**Study Selection:**

Randomized clinical trials including adults with LARC who underwent rectal resection as a final treatment were included. Studies including nonoperative treatment (watch-and-wait strategy), treatments other than rectal resection, immunotherapy, or antiangiogenic agents were excluded. Among the initially identified studies, 2.9% met the selection criteria.

**Data Extraction and Synthesis:**

Two authors independently screened the records and extracted data. Preferred Reporting Items for Systematic Reviews and Meta-analyses (PRISMA)–compliant pairwise and network meta-analyses with a random-effects model were performed in a frequentist framework, and the certainty of evidence was assessed according to the confidence in network meta-analysis approach.

**Main Outcomes and Measures:**

The primary outcome was pCR, defined as the absence of residual tumor at pathological assessment after surgery. Secondary outcomes included tolerability, toxic effects, perioperative outcomes, and long-term survival.

**Results:**

Of 925 records identified, 27 randomized clinical trials, including 13 413 adults aged 18 years or older (median age, 60.0 years [range, 42.0-63.5 years]; 67.2% male) contributed to the primary network meta-analysis. With regard to pCR, long-course chemoradiotherapy (L-CRT) plus consolidation chemotherapy (relative risk [RR], 1.96; 95% CI, 1.25-3.06), short-course radiotherapy (S-RT) plus consolidation chemotherapy (RR, 1.76; 95% CI, 1.34-2.30), and induction chemotherapy plus L-CRT (RR, 1.57; 95% CI, 1.09-2.25) outperformed standard L-CRT with single-agent fluoropyrimidine-based chemotherapy. Considering 3-year disease-free survival, S-RT plus consolidation chemotherapy (RR, 1.08; 95% CI, 1.01-1.14) and induction chemotherapy plus L-CRT (RR, 1.12; 95% CI, 1.01-1.24) outperformed L-CRT, in spite of an increased 5-year locoregional recurrence rate of S-RT plus consolidation chemotherapy (RR, 1.65; 95% CI, 1.03-2.63).

**Conclusions and Relevance:**

In this systematic review and network meta-analysis, 3 TNT protocols were identified to outperform the current standard of care in terms of pCR rates, with good tolerability and optimal postoperative outcomes, suggesting they should be recognized as first-line treatments.

## Key Points

**Question:**

Which neoadjuvant regimen is associated with the highest pathological complete response (pCR) in the treatment of locally advanced rectal cancer?

**Findings:**

In this systematic review and network meta-analysis of 27 randomized clinical trials (including a total of 13 413 individuals), the pCR rates achieved with total neoadjuvant therapy regimens outperformed standard long-course chemoradiotherapy, with long-course chemoradiotherapy plus consolidation chemotherapy showing the greatest effect estimate.

**Meaning:**

The findings of this study suggest that total neoadjuvant therapy regimens should be recognized as first-line treatments when aiming at increasing pCR rates in locally advanced rectal cancer.

## Teaser

This systematic review and network meta-analysis assesses which total neoadjuvant treatment regimens are associated with the highest pathological complete response rates compared with standard long-course chemoradiotherapy in the treatment of locally advanced rectal cancer.

## Introduction

Treatment of locally advanced rectal cancer (LARC) involves a multidisciplinary approach. The standard of care in most high-income countries consists of neoadjuvant chemoradiotherapy followed by total mesorectal excision and adjuvant chemotherapy.^1,2,3,4^ However, only two-thirds of patients receive planned adjuvant chemotherapy because of postoperative or ostomy-related complications or patients’ preference.^5^ Some pilot and phase 2 single-arm studies^6,7,8^ followed by randomized clinical trials (RCTs)^9,10,11,12^ investigated the role of total neoadjuvant therapy (TNT), which is preoperative chemotherapy in addition to radiotherapy. According to the rationale of these studies, the advantages of preoperative chemotherapy include better adherence, early treatment of micrometastases, and higher pathological complete response (pCR) rates.^11,13^ The initial results of these studies were extremely encouraging, showing high pCR rates, and TNT protocols were rapidly incorporated in some US guidelines,^2,14^ even though results on locoregional and distant recurrence rates, disease-free survival (DFS), and overall survival (OS) were not yet available.

Some systematic reviews and meta-analyses assessed the efficacy and tolerability of TNT protocols compared with standard treatment,^15,16,17,18^ but they used standard pairwise meta-analyses. More importantly, all TNT protocols were grouped together, regardless of the timing of chemotherapy and the type of radiotherapy. Current literature has not yet clarified which TNT protocol bears the best results in terms of pathological and long-term outcomes.

This study aimed to assess the efficacy of available neoadjuvant strategies in individuals with LARC by applying a network meta-analysis (NMA) approach, which permits incorporation of evidence from both direct and indirect comparisons. The primary outcome chosen was pCR, since it is unequivocally measurable and it is associated with improved long-term outcomes.^19,20,21^

## Methods

This systematic review and NMA was conducted and reported according to the Preferred Reporting Items for Systematic Reviews and Meta-Analyses (PRISMA) reporting guideline.^22^ The study protocol was registered in advance with PROSPERO (CRD42023406169).

### Study Selection and Data Extraction

We searched for RCTs including adults aged 18 years or older of both sexes who were diagnosed with LARC and scheduled to undergo rectal resection as a final treatment. Studies were considered eligible if they reported data on the primary outcome (ie, pCR). Studies including participants undergoing nonoperative treatment (watch-and-wait strategy) or treatments other than rectal resection (ie, local excision) were excluded. Studies involving immunotherapy or antiangiogenic agents were also excluded, since the use of these is limited to individuals with peculiar molecular features that could hinder the generalizability of results.

We searched MEDLINE, Embase, the Cochrane Central Register of Controlled Trials (CENTRAL), and Web of Science Core Collection electronic databases and ClinicalTrials.gov for unpublished studies from database inception to March 2, 2024 (eAppendix 1 in Supplement 1). Two authors independently screened the records and extracted data (G.T. and G.O.).

All neoadjuvant strategies were eligible, namely induction chemotherapy plus long-course chemoradiotherapy (L-CRT; induction + L-CRT); L-CRT plus consolidation chemotherapy (L-CRT + consolidation); short-course radiotherapy (S-RT) plus consolidation chemotherapy (S-RT + consolidation); neoadjuvant chemotherapy alone (CHT); S-RT plus early rectal resection (7-10 days; S-RTearly); S-RT plus delayed rectal resection (4-6 weeks; S-RTdelayed); long-course RT (L-RT); L-CRT with single-agent fluoropyrimidine-based chemotherapy (L-CRT1); and L-CRT with duplex chemotherapy drug (fluoropyrimidine plus oxaliplatin; L-CRT2). The L-CRT1 was selected as the common comparator, since it is the recommended treatment in international guidelines^1,2,4^ and it was established as standard of care in most of the included RCTs.

### Outcomes

The primary outcome was the number of participants achieving pCR, defined as the absence of residual tumor at pathological assessment after surgery (ypT0N0). Secondary outcomes included tolerability (rate of participants who received the complete planned treatment dose); toxic effects (rate of participants experiencing chemotherapy- or radiotherapy-associated adverse events of grade 3 or above, in which adverse events were assessed and graded from 1 to 5 by the investigators using Common Terminology Criteria for Adverse Events, version 4, with grade 5 indicating death); dropouts by any cause; preoperative treatment–related deaths; rate of participants undergoing surgery after neoadjuvant treatment; rate of potentially curative resections (R0); rate of negative circumferential resection margins; rate of participants who were node negative on pathological examination (ypN0); rate of severe postoperative complications, graded as Clavien-Dindo grade III or above (which ranges from grades I to V, with higher numbers indicating more severe adverse events)^23^; anastomotic leak rate; locoregional recurrence rate, defined as local recurrence after R0 to R1 resection at 3 and 5 years; distant recurrence rate at 3 and 5 years; locoregional failure, defined as locally progressive disease leading to an unresectable tumor, R2 resection, or locoregional recurrence at 3 and 5 years; DFS at 3 and 5 years; and OS at 3 and 5 years.

### Statistical Analysis

For each outcome, we performed both pairwise and NMA with a random-effects model in a frequentist framework using RStudio, version 2023.06.0-421 (R Project for Statistical Computing) using package netmeta, version 2.9-0 and Stata, version 17.0 (StataCorp LLC) using package mvmeta, version 2.3. We calculated dichotomous data on a strict intention-to-treat basis, considering all randomized participants as the denominator and calculated pooled relative risks (RRs) with 95% CIs. For the secondary outcomes’ postoperative complications and anastomotic leak rate, we conducted per-protocol analyses, considering patients undergoing surgery as the denominator. For the primary outcome, we assumed that participants excluded from the trial had experienced a negative outcome (ie, no pCR). In case of missing data, we contacted trial authors or, alternatively, used validated statistical methods of imputation.^24^ For the primary outcome, we calculated the number needed to treat, defined as the number of individuals needed to be treated with 1 treatment vs another for 1 individual to have an additional desirable (number needed to treat to benefit) or undesirable (number needed to treat to harm) outcome.^25,26^ We assessed global heterogeneity using τ^2^ (low: τ^2^ ≤ 0.010, moderate: 0.010 < τ^2^ ≤ 0.242, and high: τ^2^ > 0.242) and *I*^2^ (low: 0%-40%, moderate: 30%-60%, substantial: 50%-90%, and considerable: 75%-100%).^27^ For the NMA, common heterogeneity across all comparisons^28^ was assumed and estimated in each network.

To assess transitivity assumption (ie, when effect modifiers are equally distributed across the comparisons), we extracted key potential effect modifiers, namely study design (open label or double blind), sample size, definition of LARC, doses and cycles of chemotherapy agents, doses and modality of radiotherapy, months of follow-up, median year of study conduct, participants discontinuing treatment before the end point, sex, mean age, percentage of clinical T4 (cT4), participants with clinically suspected nodal metastases, mean distance from the anal verge, and percentage of pathological T4 (ypT4). By comparing their distribution across comparisons, we formulated a judgment on whether differences in their distributions were large enough to threaten the validity of the analysis.^29^ We considered such differences as relevant when significant imbalances emerged according to the Kruskal-Wallis test (continuous variables) and meta-regression analyses showing an association with the treatment effect.^30,31^ For the primary outcome, we calculated mean ranks of treatments using the R gemtc package, version 1.0-2 (R Project for Statistical Computing).

If more than 10 studies were included in the primary outcome, we assessed publication bias by visually inspecting the funnel plot and performing the Egger’s regression test (eAppendix 3 in Supplement 1).^32^ For the primary outcome, we assessed the confidence of evidence according to the confidence in network meta-analysis (CINeMA) method (eAppendix 5 in Supplement 1).^33,34^ For the primary outcome, we conducted sensitivity analyses excluding trials with an overall high risk of bias according to Risk of Bias, version 2.0 (Cochrane Methods),^27^ a high risk of indirectness, and CHT as 1 of the treatment arms. We also conducted an additional analysis for the primary outcome using the per-protocol population as the denominator. A 2-sided *P *< .05 was the threshold for statistical significance. Complete statistical methods are reported in the eMethods in Supplement 1.

## Results

We identified 925 records after a database and hand search. After removing duplicates and examining titles and abstracts, we selected 80 records for full-text assessment. Of these, 27 studies (2.9%) were eligible for inclusion,^10,11,12,35,36,37,38,39,40,41,42,43,44,45,46,47,48,49,50,51,52,53,54,55,56,57,58,59,60,61,62,63,64,65,66,67,68^ accounting for 13 413 participants aged 18 years or older (median age, 60.0 years [range, 42.0-63.5 years]; 32.8% female and 67.2% male) (eFigure 1 in Supplement 1). The full list of studies is provided in eTables 1 and 2 in Supplement 1.

### Primary Outcome

**Figure 1.**
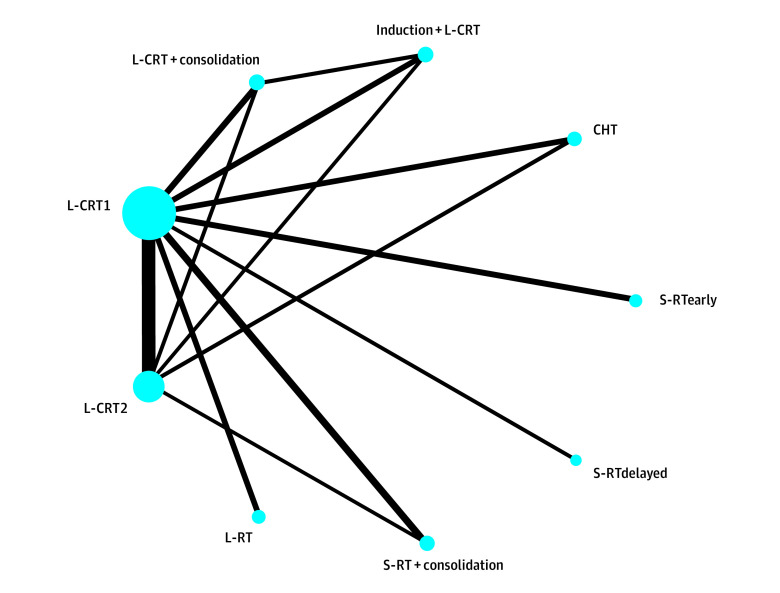
Network Plot Comparing Each Treatment With the Common Comparator Long-Course Chemoradiotherapy With Single-Agent Fluoropyrimidine (L-CRT1) for Primary Outcome Pathological Complete Response The thickness of lines is proportional to the number of studies comparing the 2 treatments, and the size of circles is proportional to the number of individuals for each treatment. CHT indicates chemotherapy; induction + L-CRT, induction CHT plus consolidation L-CRT; L-CRT + consolidation, L-CRT plus consolidation CHT; L-CRT2, L-CRT with duplex CHT drug (fluoropyrimidine plus oxaliplatin); L-RT, long-course radiotherapy; S-RT + consolidation, short-course RT plus consolidation CHT; S-RTdelayed, S-RT plus delayed rectal resection; S-RTearly, S-RT plus early rectal resection.

**Figure 2.**
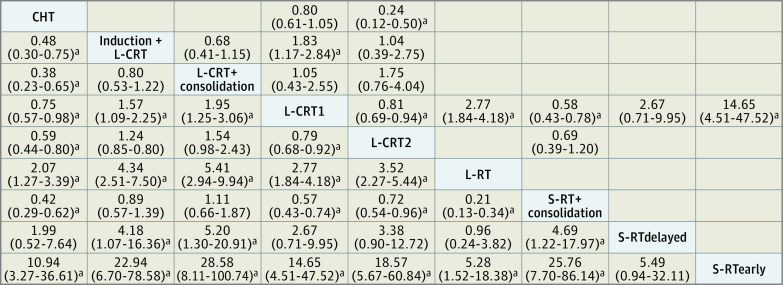
League Table for the Primary Outcome of Pathological Complete Response Treatments included in the analysis are shown in boldface on a diagonal in alphabetical order. Results of the network meta-analysis are reported in the lower left part of the matrix, and results from the pairwise meta-analysis are reported in the upper right matrix of the table. Each cell presents the relative risk (RR) and the corresponding 95% CI, and RRs greater than 1 favor the column-defining treatment (ie, the left-most cell on the diagonal). ^a^Significant RR (95% CI).

**Figure 3.**
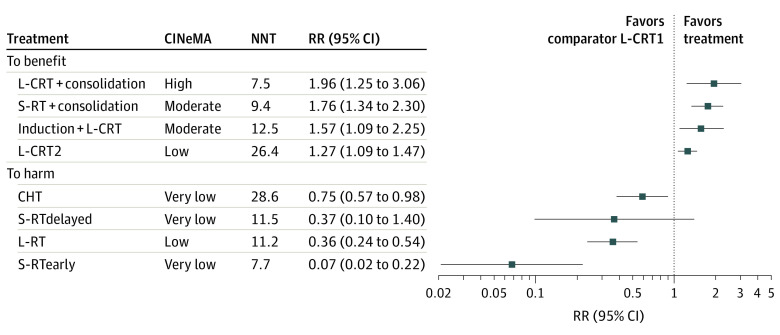
Forest Plot Comparing Each Treatment With the Common Comparator Long-Course Chemoradiotherapy With Single-Agent Fluoropyrimidine (L-CRT1) for the Primary Outcome Pathological Complete Response Relative risks (RRs) greater than 1 favor the treatment over L-CRT1. Squares indicate RRs; horizontal lines, 95% CIs for RRs. CHT indicates chemotherapy; CINeMA, confidence in network meta-analysis; induction + L-CRT, induction CHT plus consolidation long-course chemoradiotherapy; L-CRT + consolidation, L-CRT plus consolidation CHT; L-CRT2, L-CRT with duplex CHT drug (fluoropyrimidine plus oxaliplatin); L-RT, long-course radiotherapy; NNT, number needed to treat (for details, see eAppendix 4 in Supplement 1); S-RT + consolidation, short-course RT plus consolidation CHT; S-RTdelayed, S-RT plus delayed rectal resection; S-RTearly, S-RT plus early rectal resection.

For the primary outcome, pCR, the transitivity assumption was not violated for any of the potential effect modifiers analyzed (eFigures 2 and 3 in Supplement 1). The network plot (Figure 1) shows that all interventions were compared with L-CRT1 in at least 1 study. The league table (Figure 2) shows all head-to-head comparisons between treatments according to the network and the pairwise meta-analyses. Figure 3 shows a more detailed comparison between each treatment and the common comparator L-CRT1, which was outperformed (by decreasing effect size) by L-CRT + consolidation (RR, 1.96; 95% CI, 1.25-3.06, high CINeMA certainty), S-RT + consolidation (RR, 1.76; 95% CI, 1.34-2.30, moderate certainty), induction + L-CRT (RR, 1.57; 95% CI, 1.09-2.25, moderate certainty), and L-CRT2 (RR, 1.27; 95% CI, 1.09-1.47, low certainty). No significant differences emerged between S-RTdelayed and L-CRT1 (RR, 0.37; 95% CI, 0.10-1.40, very low certainty), while CHT (RR, 0.75; 95% CI, 0.57-0.98, very low certainty), L-RT (RR, 0.36; 95% CI, 0.24-0.54, low certainty), and S-RTearly (RR, 0.07; 95% CI, 0.02-0.22, very low certainty) were outperformed by L-CRT1. The rank test supported the following ranking of treatments, ordered from the best performing to worst: L-CRT + consolidation, S-RT + consolidation, induction + L-CRT, L-CRT2, L-CRT1, CHT, S-RTdelayed, L-RT, and S-RTearly (eAppendix 2 in Supplement 1). The weighted mean absolute risks of pCR were 21.5% for induction + L-CRT, 21.5% for L-CRT + consolidation, 18.6% for S-RT + consolidation, 17.2% for L-CRT2, 14.9% for CHT, 14.3% for L-CRT1, 4.3% for L-RT, 4.0% for S-RTdelayed, and 0.9% for S-RTearly. Overall, the NMA showed moderate heterogeneity (τ^2^ = 0.019; *I*^2^ = 26.3%; 95% CI, 0%-56.8%) and inconsistency according to the global approach (Cochran *Q* = 15.18; *df*, 6; *P* = .02). The local approach showed significant inconsistency for 2 of 10 comparisons (namely, L-CRT1 vs CHT and L-CRT2 vs CHT) (eAppendix 2 in Supplement 1). Sensitivity analyses provided results largely consistent with the primary analysis. The analysis excluding CHT arms showed no heterogeneity and inconsistency, and overall results were not affected. Similarly, the additional analysis on the per-protocol population showed results that were overall consistent with those from the primary analysis (eAppendix 6 in Supplement 1).

### Secondary Outcomes

#### Toxic Effects and Tolerability

The results of the NMA for toxic effects and tolerability outcomes are reported in Table 1 (see details in eAppendices 7 and 8 in Supplement 1). In comparison with L-CRT1, S-RT + consolidation (RR, 0.90; 95% CI, 0.82-0.99) and L-CRT2 (RR, 0.91; 95% CI, 0.86-0.97) showed better tolerability, while L-RT showed worse tolerability (RR, 1.23; 95% CI, 1.10-1.40). Nevertheless, S-RT + consolidation (RR, 2.01; 95% CI, 1.39-2.91), L-CRT2 (RR, 1.81; 95% CI, 1.44-2.27), CHT (RR, 1.67; 95% CI, 1.04-2.64), and induction + L-CRT (RR, 1.64; 95% CI, 1.05-2.56) showed higher toxic effects, while L-RT (RR, 0.19; 95% CI, 0.08-0.44) and S-RTearly (RR, 0.12; 95% CI, 0.05-0.28) showed reduced toxic effects compared with L-CRT1. No differences were found in terms of dropping out for any reason (eAppendix 9 in Supplement 1), preoperative treatment–related deaths (eAppendix 10 in Supplement 1), and number of patients who underwent surgery (eAppendix 11 in Supplement 1).

**Table 1.** Preoperative and Short-Term Secondary Outcomes

Characteristics and treatments	Outcome									
	Tolerability of treatment	Toxic effects of treatment^a^	Dropped out for any reason	Treatment-related deaths	Underwent surgery	Rate of R0	Rate of negative CRM	Rate of ypN0	Severe postoperative complications^b^	Anastomotic leak
Network characteristics										
Studies, No.	25	22	27	24	27	18	11	21	9	17
Participants, No.	11 987	11 568	13 383	11 963	13 413	9145	4963	10 070	3525	8333
τ^2^	0.007	0.080	0.003	0	<0.001	<0.001	0.008	0.002	0.006	0
Consistency										
CnG, Cochran *Q*; *df*	3.73; 6	2.00; 4	8.56; 6	0.98; 6	8.50; 6	4.55; 5	11.68; 1	6.19; 4	2.50; 1	4.88; 5
*P* value	.71	.74	.20	.99	.20	.47	<.001	.18	.11	.43
CnL, comparisons, No./total No.^c^	0/10	0/8	0/10	0/10	0/10	0/9	3/3	0/8	0/3	0/9
Comparison vs L-CRT1, RR (95% CI)										
L-CRT + consolidation	0.93 (0.86-1.03)	1.65 (0.93-2.93)	1.03 (0.51-2.09)	1.19 (0.29-4.93)	1.01 (0.96-1.06)	0.95 (0.87-1.03)	1.00 (0.83-1.20)	1.14 (0.98-1.33)	0.99 (0.53-1.83)	1.04 (0.21-5.07)
S-RT + consolidation	0.90 (0.82-0.99)^d^	2.01 (1.39-2.91)^d^	0.91 (0.67-1.23)	0.71 (0.25-1.99)	1.02 (0.98-1.05)	1.04 (0.99-1.09)	1.03 (0.85-1.25)	1.08 (1.00-1.18)^d^	0.92 (0.61-1.41)	1.29 (0.63-2.63)
Induction + L-CRT	0.95 (0.86-1.06)	1.64 (1.05-2.56)^d^	1.42 (0.76-2.66)	1.30 (0.35-4.77)	0.99 (0.96-1.03)	0.97 (0.91-1.03)	1.05 (0.91-1.22)	1.08 (0.96-1.22)	NA	0.90 (0.53-1.52)
L-CRT2	0.91 (0.86-0.97)^d^	1.81 (1.44-2.27)^d^	1.14 (0.88-1.50)	1.48 (0.76-2.88)	0.99 (0.98-1.01)	1.00 (0.97-1.02)	1.02 (0.90-1.16)	1.02 (0.95-1.09)	1.13 (0.87-1.47)	1.08 (0.85-1.36)
CHT	1.01 (0.91-1.11)	1.67 (1.04-2.64)^d^	1.05 (0.74-1.48)	1.90 (0.49-7.39)	0.99 (0.96-1.03)	1.00 (0.96-1.04)	1.01 (0.84-1.22)	0.93 (0.85-1.02)	NA	0.62 (0.39-0.96)^d^
S-RTdelayed	NA	NA	2.33 (0.60-9.07)	NA	0.94 (0.86-1.03)	0.89 (0.76-1.05)	0.94 (0.74-1.18)	0.80 (0.62-1.03)	NA	0.85 (0.24-3.02)
L-RT	1.23 (1.10-1.40)^d^	0.19 (0.08 to 0.44)^d^	0.59 (0.33-1.08)	1.01 (0.14-7.17)	1.02 (0.99-1.04)	NA	NA	0.92 (0.83-1.01)	NA	1.00 (0.48-2.06)
S-RTearly	1.05 (0.88-1.25)	0.12 (0.05-0.28)^d^	0.50 (0.23-1.06)	0.68 (0.11-4.12)	1.02 (0.98-1.06)	1.00 (0.93-1.07)	0.94 (0.76-1.16)	0.94 (0.77-1.14)	1.39 (0.68-2.82)	1.74 (0.52-5.82)

Abbreviations: CHT, chemotherapy; CnG, consistency–global test; CnL, consistency–local test; CRM, circumferential resection margin; induction + L-CRT, induction CHT plus consolidation long-course chemoradiotherapy; L-CRT + consolidation, L-CRT plus consolidation CHT; L-CRT1, L-CRT with single-agent fluoropyrimidine-based CHT; L-CRT2, L-CRT with duplex CHT drug (fluoropyrimidine plus oxaliplatin); L-RT, long-course radiotherapy; NA, not available; R0, potentially curative resections; RR, relative risk; S-RT + consolidation, short-course RT plus consolidation CHT; S-RTdelayed, S-RT plus delayed rectal resection; S-RTearly, S-RT plus early rectal resection; ypN0, node-negative tumor on a pathological examination.

^a^ Rate of participants experiencing chemotherapy- or radiotherapy-associated adverse events of grade 3 or above, in which adverse events were assessed and graded from 1 to 5 by the investigators using Common Terminology Criteria for Adverse Events, version 4, with grade 5 indicating death.

^b^ Graded as Clavien-Dindo grade III or above; scores range from grades I to V, with higher numbers indicating more severe adverse events.^23^

^c^ The denominator is the total number of comparisons included in the analysis; the numerator is the number of inconsistencies between direct and indirect estimates.

^d^ Significant RR (95% CI).

#### Pathological and Surgical Outcomes

No significant differences were found in terms of negativity of circumferential resection margins, and the rate of curative resections (Table 1 and eAppendices 12 and 13 in Supplement 1). With regard to ypN0 rates, S-RT + consolidation (RR, 1.08; 95% CI, 1.00-1.18) showed better results compared with L-CRT1 (eAppendix 14 in Supplement 1). None of the treatments showed a higher risk of severe postoperative complications graded as a Clavien-Dindo score of III or above (eAppendix 15 in Supplement 1). Considering anastomotic leak, only CHT showed better performance (RR, 0.62; 95% CI, 0.39-0.96), while other treatments did not show any differences (eAppendix 16 in Supplement 1).

#### Recurrence

Data for the NMA on long-term outcomes were available from a limited number of studies (Table 2). As compared with L-CRT1, L-RT (RR, 2.08; 95% CI, 1.34-3.22) and S-RT + consolidation (RR, 1.65; 95% CI, 1.03-2.63) showed significantly higher risk of locoregional recurrence at 5 years (eAppendices 17 and 18 in Supplement 1). With regard to locoregional failure, L-CRT + consolidation (RR, 0.41; 95% CI, 0.22-0.78), induction + L-CRT (RR, 0.48; 95% CI, 0.27-0.87), and L-CRT2 (RR, 0.72; 95% CI, 0.53-0.98) showed better results at 3 years compared with L-CRT1 (eAppendix 19 in Supplement 1), while only L-RT (RR, 1.70; 95% CI, 1.22-2.36) showed a worse performance at 5 years (eAppendix 20 in Supplement 1). In contrast, S-RT + consolidation (RR, 0.83; 95% CI, 0.71-0.98) and L-CRT2 (RR, 0.80; 95% CI, 0.69-0.93) showed significantly lower risk of distant recurrence at 3 years compared with L-CRT1 (eAppendix 21 in Supplement 1). The result for S-RT + consolidation was supported at 5 years (RR, 0.76; 95% CI, 0.61-0.95) (eAppendix 22 in Supplement 1).

**Table 2.** Long-Term Secondary Outcomes

Characteristic or treatment	Time of outcome, y									
	Locoregional recurrence		Locoregional failure		Distant recurrence		Disease-free survival		Overall survival	
	3	5	3	5	3	5	3	5	3	5
Studies, No.	11	7	10	6	8	5	13	7	11	8
Participants, No.	5749	4886	5234	5234	4811	3016	7409	5302	5576	5625
τ^2^	0	0	0	NA	0	0	0	0	0.002	0.005
Consistency										
CnG, Cochran *Q*; *df*	0.88; 3	NA	0.44; 2	NA	0.24; 1	NA	0.66; 3	NA	2.06; 3	NA
*P* value	.83	NA	.80	NA	.62	NA	.88	NA	.56	NA
CnL, comparisons, No./total No.^a^	0/8	NA	0/6	NA	0/3	NA	0/8	NA	0/8	NA
Comparison vs L-CRT1, RR (95% CI)										
L-CRT + consolidation	0.59 (0.25-1.25)	NA	0.41 (0.22-0.78)^b^	NA	0.68 (0.38-1.23)	NA	1.15 (0.99-1.33)	NA	1.08 (0.96-1.21)	NA
S-RT + consolidation	1.08 (0.76-1.53)	1.65 (1.03-2.63)^b^	1.04 (0.74-1.45)	1.43 (0.96-2.15)	0.83 (0.71-0.98)^b^	0.76 (0.61-0.95)^b^	1.08 (1.01-1.14)^b^	1.10 (1.00-1.20)^b^	1.07 (1.01-1.14)^b^	1.02 (0.97-1.19)
Induction + L-CRT	0.76 (0.37-1.54)	2.10 (0.22-19.99)	0.48 (0.27-0.87)^b^	2.70 (0.55-13.35)	0.76 (0.55-1.05)	0.98 (0.48-2.03)	1.12 (1.01-1.24)^b^	0.98 (0.74-1.30)	1.06 (0.97-1.16)	0.96 (0.75-1.24)
L-CRT2	0.67 (0.49-0.93)^b^	0.77 (0.55-1.08)	0.72 (0.53-0.98)^b^	0.85 (0.52-1.38)	0.80 (0.69-0.93)^b^	0.92 (0.77-1.09)	1.05 (1.01-1.08)^b^	1.02 (0.97-1.08)	1.00 (0.95-1.05)	1.04 (0.95-1.14)
CHT	0.96 (0.46-2.00)	1.13 (0.47-2.72)	1.05 (0.60-1.84)	1.07 (0.51-2.23)	NA	NA	1.00 (0.89-1.12)	1.03 (0.97-1.09)	1.01 (0.92-1.11)	0.99 (0.86-1.15)
S-RTdelayed	NA	NA	NA	NA	NA	NA	0.79 (0.62-0.99)^b^	NA	0.94 (0.78-1.12)	NA
L-RT	NA	2.08 (1.34-3.22)^b^	NA	1.70 (1.22-2.36)^b^	NA	NA	NA	0.93 (0.83-1.06)	NA	1.01 (0.85-1.19)
S-RTearly	1.72 (0.70-4.27)	1.34 (0.58-3.10)	1.72 (0.70-4.27)	1.34 (0.58-3.10)	NA	0.92 (0.65-1.30)	NA	NA	NA	1.07 (0.88-1.30)

Abbreviations: CHT, chemotherapy; CnG, consistency–global test; CnL, consistency–local test; induction + L-CRT, induction CHT plus consolidation long-course chemoradiotherapy; L-CRT + consolidation, L-CRT plus consolidation CHT; L-CRT1, L-CRT with single-agent fluoropyrimidine-based CHT; L-CRT2, L-CRT with duplex CHT drug (fluoropyrimidine plus oxaliplatin); L-RT, long-course radiotherapy; NA, not available; RR, relative risk; S-RT + consolidation, short-course RT plus consolidation CHT; S-RTdelayed, S-RT plus delayed rectal resection; S-RTearly, S-RT plus early rectal resection.

^a^ The denominator is the total number of comparisons included in the analysis; the numerator is the number of inconsistencies between direct and indirect estimates.

^b^ Significant RR (95% CI).

#### Survival

Considering 3-year DFS, induction + L-CRT (RR, 1.12; 95% CI, 1.01-1.24), S-RT + consolidation (RR, 1.08; 95% CI, 1.01-1.14), and L-CRT2 (RR, 1.05; 95% CI, 1.01-1.08) showed significantly better results, while S-RTdelayed (RR, 0.79; 95% CI, 0.62-0.99) showed worse 3-year DFS (eAppendix 23 in Supplement 1). Short-course RT + consolidation showed better outcomes in terms of 5-year DFS (RR, 1.10; 95% CI, 1.00-1.20) (eAppendix 24 in Supplement 1). With regard to 3-year OS, S-RT + consolidation (RR, 1.07; 95% CI, 1.01-1.14) showed better results (eAppendix 25 in Supplement 1), but no treatment outperformed the others at 5 years (eAppendix 26 in Supplement 1).

## Discussion

This is the first study, to our knowledge, comparing all available RCTs on neoadjuvant treatments for LARC using the NMA technique. In the era of TNT and personalized medicine, it is of utmost importance to clearly define the efficacy, tolerability, and oncologic benefits of the new protocols against current standard, L-CRT.

Considering the primary outcome pCR, our study showed significantly higher rates with L-CRT + consolidation, S-RT + consolidation, induction + L-CRT, and L-CRT2 compared with L-CRT1. According to our results, we expect that 7.5 individuals should be treated with L-CRT + consolidation to obtain 1 more individual with pCR compared with the common comparator (Figure 3). This number needed to treat should be regarded as clinically relevant, considering an association with significant improvement in long-term survival of patients reaching pCR^19,20,21^ and that the active comparator is currently considered the gold standard in many countries.^1,4,69^ Although the Clinical Practice Guidelines from the National Comprehensive Cancer Network and the American Society of Colon and Rectal Surgeons suggest TNT as the preferred treatment for patients with LARC as an alternative to L-CRT1,^2,14^ European and Eastern guidelines still suggest standard chemoradiotherapy.^1,4,69^ Despite some increase in toxic effects with TNT, there were no significant differences in terms of the number of patients undergoing surgery, postoperative complications, and pathological outcomes, including the rate of R0 and circumferential resection margin–negative specimens. Only the CHT arm showed a decreased anastomotic leak rate.

The encouraging results obtained for pCR did not completely translate into survival benefits. Only S-RT + consolidation showed better 5-year distant recurrence and DFS, with the drawback of a higher locoregional recurrence rate. In contrast, L-CRT + consolidation, induction + L-CRT, and L-CRT2 showed better locoregional control at 3 years, but the results were not supported at 5 years. Further studies as well as long-term follow-up data of the included RCTs are awaited to assess whether the encouraging results on pCR can be translated into improved OS.

Pathological complete response was chosen as the primary outcome of our NMA, since it is unequivocally measured and reported in all RCTs as a primary or early end point to assess response to treatment. Nevertheless, it may not represent the best surrogate for OS^70^ or the desired outcome in all patients. As evidence accumulates on the encouraging results of watch-and-wait strategies,^71,72,73^ some clinicians may choose TNT with the aim of pursuing complete clinical response and rectal preservation. However, there is no consensus on the definition of complete clinical response, limiting consistency and comparisons among trials. Future analyses should investigate DFS as the primary clinical end point in patients undergoing operative and nonoperative management, after an ultimate definition of complete clinical response and publications of long-term results of watch-and-wait trials.

### Strengths and Limitations

This study has strengths. To our knowledge, this is by far the largest and most updated systematic review on this important clinical issue and the first using an NMA method. Second, the evidence before our study did not permit the defining of the optimal sequence, type, and duration of neoadjuvant treatment. We used the NMA technique to indirectly compare various protocols, enabling us to identify the ones with the highest pCR. By using the CINeMA appraisal, we found that the most positive results were supported by high to moderate certainty of evidence, which strongly supports their generalizability and applicability. Furthermore, results for the primary outcome were largely supported by sensitivity and meta-regression analyses. Finally, we analyzed data for several efficacy and tolerability outcomes, allowing for an accurate profiling of each treatment in terms of balance between desirable and undesirable effects.

This study also has some limitations. First, most of the analyses were computed on the intention-to-treat population. Although this approach has the advantage of preserving the benefit of randomization in terms of comparability of study arms, 1 possible shortcoming is that individuals who did not receive surgery due to treatment-related complications or disease progression were pooled together with participants who underwent surgery and did not reach pCR. Although technically debatable, this choice is consistent with a conservative approach that might underestimate the actual benefit of treatments. Even though most of the TNT protocols were expected to deliver both radiation and systemic chemotherapy only before surgery, it should be noted that some of them also included adjuvant chemotherapy^12,37,38,51,63^ with potential effects on long-term survival. Further analyses will be required to investigate the role of adjuvant chemotherapy in participants who already received systemic chemotherapy within TNT. Also, since TNT involves compound treatment, future analyses may involve component analyses to elucidate which element plays the most relevant role. Moreover, there was a temporal gap among the analyzed studies, introducing potential bias related to the evolution in staging and radiation technology. However, we only included RCTs with similar inclusion criteria, and the planned doses of radiotherapy were comparable among studies. The primary analysis was burdened by moderate heterogeneity and inconsistency according to the global approach. Although the assessment of transitivity did not highlight relevant differences on several variables measuring baseline severity, we cannot exclude that subtle clinical differences between studies might have contributed to overall heterogeneity. For example, the PROSPECT (Chemotherapy Alone or Chemotherapy Plus Radiation Therapy in Treating Patients With Locally Advanced Rectal Cancer Undergoing Surgery) trial enrolled patients at lower risk and excluded cT4,^62^ whereas some studies on S-RT + consolidation showed higher percentages of cT4 at diagnosis.^11,35,68^ Nevertheless, clinical heterogeneity at baseline did not differ for the distribution of the proportion of clinically suspected nodal metastases, the proportion of pathological T4, and the mean anal verge distance.

## Conclusions

This systematic review and NMA found that TNT protocols compared with standard treatment (L-CRT1) were associated with significantly better results in terms of pCR, with L-CRT + consolidation having the best RR as well as being the protocol with the least toxic effects. Importantly, TNT protocols demonstrated feasibility and were not associated with poorer pathological curability and surgical outcomes, with promising results in terms of DFS. None of the TNT protocols were associated with an increased distant recurrence rate compared with the standard, but S-RT + consolidation was associated with a higher locoregional recurrence rate. These results suggest that TNT regimens should be recognized as first-line treatments when aiming at increased pCR. Further data on long-term follow-up might increase insight into long-term survival outcomes.

## Notes

Supplement 1. **eFigure 1.** PRISMA Flowchart **eAppendix 1.** Search Strategy and Data Extraction **eMethods.** Complete Statistical Methodology **eTable 1.** List of Studies Included/Excluded/Ongoing/Awaiting Assessment **eTable 2.** Characteristics of Included Studies **eTable 3.** Summary of Inclusion and Exclusion Criteria of Included Studies **eFigure 2.** Risk of Bias of Included Studies **eFigure 3.** Transitivity Assessment and Meta-Regression **eAppendix 2.** Primary Outcome: Patients With Pathologic Complete Response **eAppendix 3.** Primary Outcome: Assessment of Publication Bias **eAppendix 4.** Primary Outcome: Number Needed to Treat **eAppendix 5.** Primary Outcome: CINeMA **eAppendix 6.** Primary Outcome: Sensitivity Analyses **eAppendix 7.** Tolerability of Treatment **eAppendix 8.** Toxicity of Treatment **eAppendix 9.** Dropouts by Any Cause **eAppendix 10.** Preoperative Treatment-Related Deaths **eAppendix 11.** Rate of Randomized Patients Who Underwent Surgery **eAppendix 12.** Rate of R0 Resections **eAppendix 13.** Rate of Negative CRM **eAppendix 14.** Rate of ypN0 **eAppendix 15.** Rate of Postoperative Complications Clavien-Dindo III or Greater **eAppendix 16.** Rate of Anastomotic Leak **eAppendix 17.** Locoregional Recurrence at 3 Years **eAppendix 18.** Locoregional Recurrence at 5 Years **eAppendix 19.** Locoregional Failure at 3 Years **eAppendix 20.** Locoregional Failure at 5 Years **eAppendix 21.** Distant Recurrence at 3 Years **eAppendix 22.** Distant Recurrence at 5 Years **eAppendix 23.** Disease-Free Survival at 3 Years **eAppendix 24.** Disease-Free Survival at 5 Years **eAppendix 25.** Overall Survival at 3 Years **eAppendix 26.** Overall Survival at 5 Years
eReferences

Supplement 2.
Data Sharing Statement
