# Supplementary material for: Treatment of Locally Advanced Rectal Cancer in the Era of Total Neoadjuvant Therapy: A Systematic Review and Network Meta-Analysis
Source: JAMA Netw Open. 2024 Jun 4;7(6):e2414702. doi: 10.1001/jamanetworkopen.2024.14702 (PMC11151159; doi:10.1001/jamanetworkopen.2024.14702)
Supplement: Supplement 1. — eFigure 1. PRISMA Flowchart eAppendix 1. Search Strategy and Data Extraction eMethods. Complete Statistical Methodology eTable 1. List of Studies Included/Excluded/Ongoing/Awaiting Assessment eTable 2. Characteristics of Included Studies eTable 3. Summary of Inclusion and Exclusion Criteria of Included Studies eFigure 2. Risk of Bias of Included Studies eFigure 3. Transitivity Assessment and Meta-Regression eAppendix 2. Primary Outcome: Patients With Pathologic Complete Response eAppendix 3. Primary Outcome: Assessment of Publication Bias eAppendix 4. Primary Outcome: Number Needed to Treat eAppendix 5. Primary Outcome: CINeMA eAppendix 6. Primary Outcome: Sensitivity Analyses eAppendix 7. Tolerability of Treatment eAppendix 8. Toxicity of Treatment eAppendix 9. Dropouts by Any Cause eAppendix 10. Preoperative Treatment-Related Deaths eAppendix 11. Rate of Randomized Patients Who Underwent Surgery eAppendix 12. Rate of R0 Resections eAppendix 13. Rate of Negative CRM eAppendix 14. Rate of ypN0 eAppendix 15. Rate of Postoperative Complications Clavien-Dindo III or Greater eAppendix 16. Rate of Anastomotic Leak eAppendix 17. Locoregional Recurrence at 3 Years eAppendix 18. Locoregional Recurrence at 5 Years eAppendix 19. Locoregional Failure at 3 Years eAppendix 20. Locoregional Failure at 5 Years eAppendix 21. Distant Recurrence at 3 Years eAppendix 22. Distant Recurrence at 5 Years eAppendix 23. Disease-Free Survival at 3 Years eAppendix 24. Disease-Free Survival at 5 Years eAppendix 25. Overall Survival at 3 Years eAppendix 26. Overall Survival at 5 Years eReferences [file jamanetwopen-e2414702-s001.pdf]

Supplemental Online Content

Turri G, Ostuzzi G, Vita G, et al. Treatment of locally advanced rectal cancer in the era of total neoadjuvant therapy: a systematic review and network meta-analysis. *JAMA Netw Open*. 2024;7(6):e2414702. doi:10.1001/jamanetworkopen.2024.14702

eFigure 1. PRISMA Flowchart
eAppendix 1. Search Strategy and Data Extraction
eMethods 1. Complete Statistical Methodology
eTable 1. List of Studies Included/Excluded/Ongoing/Awaiting Assessment
eTable 2. Characteristics of Included Studies
eTable 3. Summary of Inclusion and Exclusion Criteria of Included Studies
eFigure 2. Risk of Bias of Included Studies
eFigure 3. Transitivity Assessment and Meta-Regression
eAppendix 2. Primary Outcome: Patients With Pathologic Complete Response
eAppendix 3. Primary Outcome: Assessment of Publication Bias
eAppendix 4. Primary Outcome: Number Needed to Treat
eAppendix 5. Primary Outcome: CINeMA
eAppendix 6. Primary Outcome: Sensitivity Analyses
eAppendix 7. Tolerability of Treatment
eAppendix 8. Toxicity of Treatment
eAppendix 9. Dropouts by Any Cause
eAppendix 10. Preoperative Treatment Related Deaths
eAppendix 11. Rate of Randomized Patients Who Underwent Surgery
eAppendix 12. Rate of R0 Resections
eAppendix 13. Rate of Negative CRM
eAppendix 14. Rate of ypN0
eAppendix 15. Rate of Postoperative Complications Clavien-Dindo 3 or Greater
eAppendix 16. Rate of Anastomotic Leak
eAppendix 17. Locoregional Recurrence at 3 Years
eAppendix 18. Locoregional Recurrence at 5 Years
eAppendix 19. Locoregional Failure at 3 Years
eAppendix 20. Locoregional Failure at 5 Years
eAppendix 21. Distant Recurrence at 3 Years
eAppendix 22. Distant Recurrence at 5 Years
eAppendix 23. Disease-Free Survival at 3 Years
eAppendix 24. Disease-Free Survival at 5 Years
eAppendix 25. Overall Survival at 3 Years
eAppendix 26. Overall Survival at 5 Years
eReferences

This supplemental material has been provided by the authors to give readers additional information about their work.

© 2024 Turri G et al. *JAMA Network Open*.

eFigure 1. PRISMA Flowchart

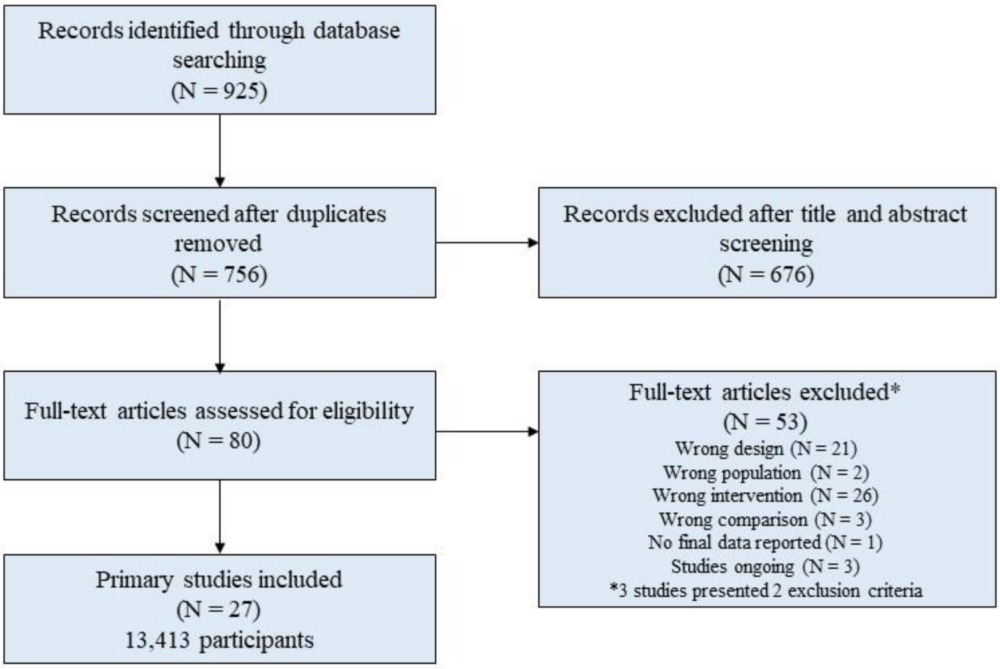

© 2024 Turri G et al. *JAMA Network Open*.

eAppendix 1. Search Strategy and Data Extraction

We searched without time or language restrictions Medline, EMBASE, Cochrane Central Register of Controlled Trials (CENTRAL), and Web of Science Core Collection electronic databases. Further, we searched ClinicalTrials.gov for unpublished studies. We searched records from database inception to June 8, 2023 (full search strategy attached below). Additionally, we screened references of assessed articles for other potential matches for inclusion.

PubMed (08/06/2023; 561 results)

(("cancer"[Title/Abstract] OR "oncolog*"[Title/Abstract] OR "adenocarcinoma"[Title/Abstract] OR "tumor"[Title/Abstract] OR "neoplas*"[Title/Abstract]) AND ("rectum"[Title/Abstract] OR "rectal"[Title/Abstract]) AND ("neoadjuvant"[Title/Abstract] OR "preoperat*"[Title/Abstract])) AND (randomizedcontrolledtrial[Filter])

Web of Science, Core Collection (08/06/2023; 329 results)

#1 (((((TI=(cancer)) OR TI=(oncolog*)) OR TI=(adenocarcinoma)) OR TI=(tumor)) OR TI=(neoplas*))

#2 (TI=(rectum)) OR TI=(rectal)

#3 (TI=(neoadjuvant)) OR TI=(preoperat*)

((((#1) AND #2) AND #3) AND ALL=(randomized controlled trial)) AND LA=(English)

Indexes=SCI-EXPANDED, SSCI, A&HCI, CPCI-S, CPCI-SSH, BKCI-S, BKCI-SSH, ESCI, CCR-EXPANDED, IC Timespan=All years

CENTRAL (08/06/2023; 837 results)

Neoadjuvant therapy rectal cancer in Title Abstract Keyword AND English in Language - (Word variations have been searched)

ClinicalTrials.gov (08/06/2023, 399 results)

Neoadjuvant therapy; preoperative therapy, Condition: Rectal cancer, Filters: Interventional studies, Adults, Older Adults. Also searched for synonyms: preoperative therapy, induction therapy, treatment, rectal carcinoma, cancer of the rectum.

Two authors (GT, GO) independently assessed titles, abstracts and full texts of potentially relevant articles, and extracted data following recommendations of the Cochrane Handbook for Systematic Reviews of Interventions. Two authors (GT, GV) assessed the methodologic quality of included studies using the Cochrane Risk of Bias version 2 (RoB2) tool. Disagreements were resolved by discussion and consensus with a third senior author (CP, CB).

© 2024 Turri G et al. *JAMA Network Open*.

eMethods 1. Complete Statistical Methodology

We performed a standard pairwise, random-effects meta-analysis for every comparison, and, for each outcome, we also conducted a NMA with a random-effects model in a frequentist framework, using RStudio (version 2023.06.0-421) *netmeta* package and Stata (version 17.0) *mvmeta* package. For dichotomous outcomes, we calculated and pooled relative risks (RRs) with 95% confidence intervals (CIs).

We calculated dichotomous data on a strict intention-to-treat (ITT) basis, considering the total number of randomized participants as denominator. For the primary outcome, where participants had been excluded from the trial before the endpoint, we assumed that they experienced a negative outcome by the end of the trial.

When relevant outcomes were not reported, we asked trial authors to supply the data. In the absence of data from authors, we employed validated statistical methods to impute missing outcomes, with due consideration of the possible bias of these procedures, in accordance with the Cochrane Handbook[1]. When standard deviations (SDs) were not reported and not supplied by authors upon request, we calculated them based on the standard error (SE) or t-statistics or P values[1].

For the primary outcome, we calculated the number-needed-to-treat (NNT), defined as the number of individuals needed to be treated with one treatment versus another for one individual to have an additional desirable or undesirable outcome (number-needed-to-treat-to-benefit, NNTB, or to-harm, NNTH, respectively)[2], using validated methodology[3] (see Supplement for details).

For pairwise meta-analyses, we assessed heterogeneity by visual inspection of forest plots, and by the I^2 statistics. For the NMA, common heterogeneity across all comparisons was assumed and estimated in each network[4,5].

We assessed global heterogeneity by using the τ^2 (low: $\tau^2 \leq 0.010$, moderate: $0.010 < \tau^2 \leq 0.242$, high: $\tau^2 > 0.242$) and the I^2 (low, 0–40%; moderate, 30–60%; substantial, 50–90%; and considerable, 75–100%)[6]. For the NMA, common heterogeneity across all comparisons[4] was assumed and estimated in each network.

Transitivity assumption is met when effect modifiers are equally distributed across the comparisons. We expected that the inclusion and exclusion criteria would allow to select studies sufficiently similar in terms of characteristics of participants, study design and outcomes, in order for all treatments included in the network to be considered "exchangeable" (as if all of them were part of a large, multi-arm trial). We extracted key study characteristics judged to be potential effect modifiers, namely: study design (open-label or double-blind); number of participants included; definition of LAR; doses and cycles of chemotherapy agents; doses and modality of radiotherapy; months of follow-up; median year of study conduct; proportion of participants discontinuing treatment before study endpoint; percentage of female participants; mean age; percentage of clinical T3-4 (cT4); percentage of participants with clinically suspected nodal metastases (cN+); mean distance from the anal verge (AV); percentage of pathologic T4 after pre-operative treatment (ypT4). By comparing the distribution of these possible effect modifiers across comparisons contributing to the estimation of the treatment effect, we formulated a judgment on whether differences in their distributions were large enough to threaten the validity of the analysis[7]. We considered that distribution differences in specific study characteristics across the different treatment strategies were relevant in case of both significant imbalances according to the Kruskal-Wallis test (continuous variables), the Pearson χ^2 or the Fisher's exact test (categorical variables), and meta-regression analyses showing an actual impact on treatment effect[8,9].

We assessed the presence of inconsistency (defined as the statistical disagreement between direct and indirect evidence of a treatment comparison) by comparing direct and indirect evidence within each closed loop[10] and comparing the goodness of fit for an NMA model that assumes consistency with a model that allows for inconsistency in a "design by treatment interaction model" framework[11] by using the Stata commands mvmeta[12] and ifplot[13] and the Stata network suite[14].

For the primary outcome, we calculated the probability of each treatment of being at each possible rank, and produced mean ranks of treatments using the R *gemtc* package.

If ≥10 studies were included in the primary outcome, we assessed publication bias by visually inspecting the funnel plot, testing for asymmetry with the Egger's regression test[15], and investigating possible reasons for funnel plot asymmetry.

For the primary outcome, we assessed the confidence of evidence by using the Confidence in Network Meta-Analysis (CINeMA) methodology[16,17] through its web-based application (http://cinema.ispm.ch).

For the primary outcome, we conducted sensitivity analyses excluding trials with (a) overall high risk of bias according to RoB2; (b) high risk of indirectness; (c) CHT as one of the treatment arms.

© 2024 Turri G et al. *JAMA Network Open*.

eTable 1. List of Studies Included/Excluded/Ongoing/Awaiting Assessment

Study	in/out	Reason	Reason - details	Reference
Aschele 2011	Included			Aschele C, Cionini L, Lonardi S, et al. Primary tumor response to preoperative chemoradiation with or without oxaliplatin in locally advanced rectal cancer: Pathologic results of the STAR-01 randomized phase III trial. *J Clin Oncol*. 2011;29(20):2773-2780. doi:10.1200/JCO.2010.34.4911<br>Aschele C, Lonardi S, Cionini L, et al. Final results of STAR-01: A randomized phase III trial comparing preoperative chemoradiation with or without oxaliplatin in locally advanced rectal cancer. *J Clin Oncol*. 2016;34(15_suppl):3521-3521. doi:10.1200/jco.2016.34.15_suppl.3521
Bahadoer 2021	Included			Bahadoer RR, Dijkstra EA, van Etten B, et al. Short-course radiotherapy followed by chemotherapy before total mesorectal excision (TME) versus preoperative chemoradiotherapy, TME, and optional adjuvant chemotherapy in locally advanced rectal cancer (RAPIDO): a randomised, open-label, phase 3 trial. *Lancet Oncol*. 2021;22(1):29-42. doi:10.1016/S1470-2045(20)30555-6<br>Bahadoer RR, Hospers GAP, Marijnen CAM, et al. Risk and location of distant metastases in patients with locally advanced rectal cancer after total neoadjuvant treatment or chemoradiotherapy in the RAPIDO trial. *Eur J Cancer*. 2023;185:139-149. doi: 10.1016/j.ejca.2023.02.027<br>Dijkstra EA, Nilsson PJ, Hospers GAP, et al. Locoregional Failure During and After Short-course Radiotherapy followed by Chemotherapy and Surgery Compared to Long-course Chemoradiotherapy and Surgery – A Five-year Follow-up of the RAPIDO Trial. *Ann Surg*. 2023;Published ahead of print(4):766-772. doi: 10.1097/sla.0000000000005799
Bosset 2005	Included			Bosset JF, Calais G, Mineur L, et al. Enhanced tumorocidal effect of chemotherapy with preoperative radiotherapy for rectal cancer: Preliminary results - EORTC 22921. J Clin Oncol. 2005;23(24):5620-5627. doi:10.1200/JCO.2005.02.113<br>Bosset JF, Calais G, Mineur L, et al. Fluorouracil-based adjuvant chemotherapy after preoperative chemoradiotherapy in rectal cancer: Long-term results of the EORTC 22921 randomised study. Lancet Oncol. 2014;15(2):184-190. doi:10.1016/S1470-2045(13)70599-0
Bujko 2004	Included			Bujko K, Nowacki MP, Nasierowska-Guttmejer A, et al. Sphincter preservation following preoperative radiotherapy for rectal cancer: Report of a randomised trial comparing short-term radiotherapy vs. conventionally fractionated radiochemotherapy. In: *Radiotherapy and Oncology*. Vol 72. Elsevier; 2004:15-24. doi:10.1016/j.radonc.2003.12.006<br>Bujko K, Nowacki MP, Nasierowska-Guttmejer A, Michalski W, Bebenek M, Kryj M. Long-term results of a randomized trial comparing preoperative short-course radiotherapy with preoperative conventionally fractionated chemoradiation for rectal cancer. *Br J Surg*. 2006;93(10):1215-1223. doi:10.1002/bjs.5506
Bujko 2013	Included			Bujko K, Nasierowska-Guttmejer A, Wyrwicz L, et al. Neoadjuvant treatment for unresectable rectal cancer: An interim analysis of a multicentre randomized study. *Radiother Oncol*. 2013;107(2):171-177. doi:10.1016/j.radonc.2013.03.001<br>Bujko K, Wyrwicz L, Rutkowski A, et al. Long-course oxaliplatin-based preoperative chemoradiation versus 5 × 5 Gy and consolidation chemotherapy for cT4 or fixed cT3 rectal cancer: Results of a randomized phase III study. *Ann Oncol*. 2016;27(5):834-842. doi:10.1093/annonc/mdw062
Chakrabarti 2021	Included			Chakrabarti D, Rajan S, Akhtar N, et al. Short-course radiotherapy with consolidation chemotherapy versus conventionally fractionated long-course chemoradiotherapy for locally advanced rectal cancer: randomized clinical trial. *Br J Surg*. 2021;108(5):511-520. doi:10.1093/bjs/znab020
Conroy 2021	Included			Conroy T, Bosset JF, Etienne PL, et al. Neoadjuvant chemotherapy with FOLFIRINOX and preoperative chemoradiotherapy for patients with locally advanced rectal cancer (UNICANCER-PRODIGE 23): a multicentre, randomised, open-label, phase 3 trial. *Lancet Oncol*. 2021;22(5):702-715. doi:10.1016/S1470-2045(21)00079-6
Deng 2016	Included			Deng Y, Chi P, Lan P, et al. Modified FOLFOX6 with or without radiation versus fluorouracil and leucovorin with radiation in neoadjuvant treatment of locally advanced rectal cancer: Initial results of the Chinese FOWARC multicenter, open-label, randomized three-arm phase III trial. *J Clin Oncol*. 2016;34(27):3300-3307. doi:10.1200/JCO.2016.66.6198<br>Deng Y, Chi P, Lan P, et al. Neoadjuvant modified folfox6 with or without radiation versus fluorouracil plus radiation for locally advanced rectal cancer: Final results of the Chinese FOWARC trial. *J Clin Oncol*. 2019;37(34):3223-3233. doi:10.1200/JCO.18.02309

© 2024 Turri G et al. *JAMA Network Open*.

Fernández-Martos 2015	Included			Fernández-Martos C, Garcia-Albeniz X, Pericay C, et al. Chemoradiation, surgery and adjuvant chemotherapy versus induction chemotherapy followed by chemoradiation and surgery: Long-term results of the Spanish GCR-3 phase II randomized trial. *Ann Oncol*. 2015;26(8):1722-1728. doi:10.1093/annonc/mdv223
Fokas 2019	Included			Fokas E, Allgäuer M, Polat B, et al. Randomized phase II trial of chemoradiotherapy plus induction or consolidation chemotherapy as total neoadjuvant therapy for locally advanced rectal cancer: CAO/ArO/AIO-12. *J Clin Oncol*. 2019;37(34):3212-3222. doi:10.1200/JCO.19.00308<br>Fokas E, Schlenska-Lange A, Polat B, et al. Chemoradiotherapy Plus Induction or Consolidation Chemotherapy as Total Neoadjuvant Therapy for Patients with Locally Advanced Rectal Cancer: Long-term Results of the CAO/ARO/AIO-12 Randomized Clinical Trial. *JAMA Oncol*. 2022;8(1):e215445-e215445. doi:10.1001/jamaoncol.2021.5445
Gérard 2006	Included			Gérard JP, Conroy T, Bonnetain F, et al. Preoperative radiotherapy with or without concurrent fluorouracil and leucovorin in T3-4 rectal cancers: Results of FFCD 9203. *J Clin Oncol*. 2006;24(28):4620-4625. doi:10.1200/JCO.2006.06.7629
Gérard 2010	Included			Gérard JP, Azria D, Gourgou-Bourgade S, et al. Comparison of two neoadjuvant chemoradiotherapy regimens for locally advanced rectal cancer: Results of the phase III trial accord 12/0405-Prodige 2. *J Clin Oncol*. 2010;28(10):1638-1644. doi:10.1200/JCO.2009.25.8376<br>Gérard JP, Azria D, Gourgou-Bourgade S, et al. Clinical outcome of the ACCORD 12/0405 PRODIGE 2 randomized trial in rectal cancer. *J Clin Oncol*. 2012;30(36):4558-4565. doi:10.1200/JCO.2012.42.8771<br>Azria D, Doyen J, Jarlier M, et al. Late toxicities and clinical outcome at 5 years of the ACCORD 12/0405-PRODIGE 02 trial comparing two neoadjuvant chemoradiotherapy regimens for intermediate-risk rectal cancer. *Ann Oncol*. 2017;28(10):2436-2442. doi:10.1093/annonc/mdx351
Haddad 2017	Included			Haddad P, Miraie M, Farhan F, et al. Addition of oxaliplatin to neoadjuvant radiochemotherapy in MRI-defined T3, T4 or N+ rectal cancer: a randomized clinical trial. *Asia Pac J Clin Oncol*. 2017;13(6):416-422. doi:10.1111/ajco.12675
Jiao 2015	Included			Jiao D, Zhang R, Gong Z, et al. Fluorouracil-based preoperative chemoradiotherapy with or without oxaliplatin for stage ii/iii rectal cancer: A 3-year follow-up study. *Chinese J Cancer Res*. 2015;27(6):588-596. doi:10.3978/j.issn.1000-9604.2015.12.05
Jin 2022	Included			Jin J, Tang Y, Hu C, et al. Multicenter, Randomized, Phase III Trial of Short-Term Radiotherapy Plus Chemotherapy Versus Long-Term Chemoradiotherapy in Locally Advanced Rectal Cancer (STELLAR). *J Clin Oncol*. 2022;40(15):1681-1692. doi:10.1200/jco.21.01667
Kim 2018	Included			Kim SY, Joo J, Kim TW, et al. A Randomized Phase 2 Trial of Consolidation Chemotherapy After Preoperative Chemoradiation Therapy Versus Chemoradiation Therapy Alone for Locally Advanced Rectal Cancer: KCSG CO 14-03. *Int J Radiat Oncol Biol Phys*. 2018;101(4):889-899. doi:10.1016/j.ijrobp.2018.04.013
Latkauskas 2012	Included			Latkauskas T, Pauzas H, Gineikiene I, et al. Initial results of a randomized controlled trial comparing clinical and pathological downstaging of rectal cancer after preoperative short-course radiotherapy or long-term chemoradiotherapy, both with delayed surgery. *Color Dis*. 2012;14(3):294-298. doi:10.1111/j.1463-1318.2011.02815.x<br>Latkauskas T, Pauzas H, Kairevice L, et al. Preoperative conventional chemoradiotherapy versus short-course radiotherapy with delayed surgery for rectal cancer: Results of a randomized controlled trial. *BMC Cancer*. 2016;16(1):1-7. doi:10.1186/s12885-016-2959-9
Maréchal 2012	Included			Maréchal R, Vos B, Polus M, et al. Short course chemotherapy followed by concomitant chemoradiotherapy and surgery in locally advanced rectal cancer: A randomized multicentric phase II study. *Ann Oncol*. 2012;23(6):1525-1530. doi:10.1093/annonc/mdr473
Mei 2023	Included			Mei W-J, Wang X-Z, Li Y-F, et al. Neoadjuvant Chemotherapy with CAPOX versus Chemoradiation for Locally Advanced Rectal Cancer with Uninvolved Mesorectal Fascia (CONVERT): Initial Results of a Phase III Trial. *Ann Surg*. 2023;277(4):557-564. doi:10.1097/sla.0000000000005780
Mohiuddin 2006	Included			Mohiuddin M, Winter K, Mitchell E, et al. Randomized phase II study of neoadjuvant combined-modality chemoradiation for distal rectal cancer: Radiation therapy oncology group trial 0012. *J Clin Oncol*. 2006;24(4):650-655. doi:10.1200/JCO.2005.03.6095
Moore 2017	Included			Moore J, Price T, Carruthers S, et al. Prospective randomized trial of neoadjuvant chemotherapy during the 'wait period' following preoperative chemoradiotherapy for rectal cancer: results of the WAIT trial. *Color Dis*. 2017;19(11):973-979. doi:10.1111/codi.13724
Ngan 2012	Included			Ngan SY, Burmeister B, Fisher RJ, et al. Randomized trial of short-course radiotherapy versus long-course chemoradiation comparing rates of local recurrence in patients with T3 rectal cancer: Trans-Tasman Radiation Oncology Group Trial 01.04. *J Clin Oncol*. 2012;30(31):3827-3833. doi:10.1200/JCO.2012.42.9597

© 2024 Turri G et al. *JAMA Network Open*.

				Ansari N, Solomon MJ, Fisher RJ, et al. Acute Adverse Events and Postoperative Complications in a Randomized Trial of Preoperative Short-course Radiotherapy Versus Long-course Chemoradiotherapy for T3 Adenocarcinoma of the Rectum: Trans-Tasman Radiation Oncology Group Trial (TROG 01.04). *Ann Surg*. 2017;265(5):882-888. doi:10.1097/SLA.0000000000001987
O'Connell 2014	Included			O'Connell MJ, Colangelo LH, Beart RW, et al. Capecitabine and oxaliplatin in the preoperative multimodality treatment of rectal cancer: Surgical end points from national surgical adjuvant breast and bowel project trial R-04. *J Clin Oncol*. 2014;32(18):1927-1934. doi:10.1200/JCO.2013.53.7753
Rodel 2015	Included			Rödel C, Graeven U, Fietkau R, et al. Oxaliplatin added to fluorouracil-based preoperative chemoradiotherapy and postoperative chemotherapy of locally advanced rectal cancer (the German CAO/ARO/AIO-04 study): final results of the multicentre, open-label, randomised, phase 3 trial. *Lancet Oncol*. 2015;16(8):979-989. doi:10.1016/S1470-2045(15)00159-X
Schmoll 2021	Included			Schmoll HJ, Stein A, van Cutsem E, et al. Pre- And postoperative capecitabine without or with oxaliplatin in locally advanced rectal cancer: PETACC 6 trial by EORTC GITCG and ROG, AIO, AGITG, BGDO, and FFCD. *J Clin Oncol*. 2021;39(1):17-29. doi:10.1200/JCO.20.01740
Schrag 2023	Included			Schrag D, Shi Q, Weiser M et al. Preoperative treatment for locally advanced rectal cancer. *N Engl J Med*. 2023 Jun 4. doi: 10.1056/NEJMoa2303269. Online ahead of print.
Wang 2019	Included			Wang J, Guan Y, Gu W, et al. Long-course neoadjuvant chemoradiotherapy with versus without a concomitant boost in locally advanced rectal cancer: A randomized, multicenter, phase II trial (FDRT-002). *Radiat Oncol*. 2019;14(1). doi:10.1186/s13014-019-1420-z
Choi 2023	ONGOING			NCT05673772. Preoperative Sequential Short-course Radiation Therapy and FOLFOX for Locally Advanced Rectal Cancer (SOLAR). https://clinicaltrials.gov/ct2/show/NCT05673772?term=solar&type=Intr&cond=Rectal+Cancer&cntry=KR&draw=2&rank=1
Kim 2018	ONGOING			Kim CW, Kang BM, Kim IY, et al. Korean Society of Coloproctology (KSCP) trial of cONsolidation Chemotherapy for Locally advanced mid or low rectal cancer after neoadjUvant concurrent chemoraDiothErapy: A multicenter, randomized controlled trial (KONCLUDE). *BMC Cancer*. 2018;18(1):1-8. doi:10.1186/S12885-018-4466-7/TABLES/1
NCT03177382	ONGOING			Total Neoadjuvant Treatment vs. Chemoradiotherapy in Local Advanced Rectal Cancer With High Risk Factors (TNTCRT). ClinicalTrials.gov Identifier: NCT03177382.
Wang 2022	ONGOING			Wang Y, Shen L, Wan J, et al. Short-course radiotherapy combined with CAPOX and Toripalimab for the total neoadjuvant therapy of locally advanced rectal cancer: a randomized, prospective, multicentre, double-arm, phase II trial (TORCH). *BMC Cancer*. 2022;22(1):1-8. doi:10.1186/S12885-022-09348-Z/FIGURES/2
Aboelnaga 2015	Excluded	WRONG DESIGN	Single arm	Aboelnaga EM, Daoud MA, Eladl EI, Zaid AM. Induction FOLFOX followed by preoperative hyperfractionated radiotherapy plus bolus 5-fluorouracil in locally advanced rectal carcinoma: single arm phase I-II study. *Med Oncol.* 2015 Apr;32(4):108. doi: 10.1007/s12032-015-0556-4.
Aghili 2018	Excluded	WRONG DESIGN	Single arm	Aghili M, Sotoudeh S, Ghalehtaki R et al. Preoperative short course radiotherapy with concurrent and consolidation chemotherapies followed by delayed surgery in locally advanced rectal cancer: Preliminary results. *Radiat Oncol J* 2018; 36(1): 17-24. Doi: 10.3857/roj.2017.00185
Borg 2014	Excluded	WRONG INTERVENTION	Immunotherapy	Borg C, André T, Mantion G et al. Pathological response and safety of two neoadjuvant strategies with bevacizumab in MRI-defined locally advanced T3 resectable rectal cancer: a randomized, noncomparative phase II study. *Ann Oncol*. 2014 Nov;25(11):2205-2210. doi: 10.1093/annonc/mdu377.
Boulis-Wassif 1984	Excluded	WRONG INTERVENTION	Wrong schedule of treatment	Boulis-Wassif S, Gerard A, Loygue J, Camelot D, Buyse M, Duez N. Final results of a randomized trial on the treatment of rectal cancer with preoperative radiotherapy alone or in combination with 5-fluorouracil, followed by radical surgery. Trial of the European Organization on Research and Treatment of Cancer Gastrointestinal Tract Cancer Cooperative Group. *Cancer.* 1984 May 1;53(9):1811-8. doi: 10.1002/1097-0142(19840501)53:9<1811::aid-cncr2820530902>3.0.co;2-h.
Cedermark 1995	Excluded	WRONG INTERVENTION	No preoperative therapy in control group	Cedermark B, Johansson H, Rutqvist LE, et al. The Stockholm I trial of preoperative short term radiotherapy in operable rectal carcinoma. A prospective randomized trial. Stockholm Colorectal Cancer Study Group. *Cancer.* 1995;75:2269 –2275. doi: 10.1002/1097-0142(19950501)75:9<2269::aid-cncr2820750913>3.0.co;2-i.
Cercek 2012	Excluded	WRONG DESIGN	Single arm	Cercek A, Goodman KA, Hajj C et al. Neoadjuvant chemotherapy first, followed by chemoradiation and then surgery, in the management of locally advanced rectal cancer. *J Natl Compr Canc Netw*. 2014 Apr;12(4):513-9. doi: 10.6004/jnccn.2014.0056
Chiorean 2012	Excluded	WRONG DESIGN	Single arm	Chiorean EG, Sanghani S, Schiel MA et al. Phase II and gene expression analysis trial of neoadjuvant capecitabine plus irinotecan followed by capecitabine-based chemoradiotherapy for locally advanced rectal cancer: Hoosier Oncology Group GI03-53. *Cancer Chemother Pharmacol*. 2012 Jul;70(1):25-32. doi: 10.1007/s00280-012-1883-1

© 2024 Turri G et al. *JAMA Network Open*.

Chua 2010	Excluded	WRONG DESIGN	Single arm	Chua YJ, Barbachano Y, Cunningham D et al. Neoadjuvant capecitabine and oxaliplatin before chemoradiotherapy and total mesorectal excision in MRI-defined poor-risk rectal cancer: a phase 2 trial. *Lancet Oncol.* 2010 Mar;11(3):241-8. doi: 10.1016/S1470-2045(09)70381-X.
Cotte 2015	Excluded	WRONG INTERVENTION	Wrong schedule of treatment	Cotte E, Passot G, Decullier E et al. Pathologic Response, When Increased by Longer Interval, Is a Marker but Not the Cause of Good Prognosis in Rectal Cancer: 17-year Follow-up of the Lyon R90-01 Randomized Trial. *Int J Radiat Oncol Biol Phys*. 2016 Mar 1;94(3):544-53. doi: 10.1016/j.ijrobp.2015.10.061.
De Felice 2021	Excluded	WRONG DESIGN	Single arm	De Felice F, D'Ambrosio G, Iafrate F et al. Intensified Total Neoadjuvant Therapy in Patients With Locally Advanced Rectal Cancer: A Phase II Trial. *Clin Oncol (R Coll Radiol).* 2021 Dec;33(12):788-794. doi: 10.1016/j.clon.2021.06.006.
Dewdney 2012	Excluded	WRONG INTERVENTION	Immunotherapy	Dewdney A, Cunningham D, Tabernero J et al. Multicenter randomized phase II clinical trial comparing neoadjuvant oxaliplatin, capecitabine, and preoperative radiotherapy with or without cetuximab followed by total mesorectal excision in patients with high-risk rectal cancer (EXPERT-C). *J Clin Oncol*. 2012 May 10;30(14):1620-7. doi: 10.1200/JCO.2011.39.6036.
Fernández-Martos 2019	Excluded	WRONG INTERVENTION	Immunotherapy	Fernández-Martos C, Pericay C, Losa F et al. Effect of Aflibercept Plus Modified FOLFOX6 Induction Chemotherapy Before Standard Chemoradiotherapy and Surgery in Patients With High-Risk Rectal Adenocarcinoma: The GEMCAD 1402 Randomized Clinical Trial. *JAMA Oncol*. 2019 Nov 1;5(11):1566-1573. doi: 10.1001/jamaoncol.2019.2294.
Fisher 1988	Excluded	WRONG INTERVENTION	Adjuvant radiotherapy	Fisher B, Wolmark N, Rockette H, et al. Postoperative adjuvant chemotherapy or radiation therapy for rectal cancer: results from NSABP protocol R-01. *J Natl Cancer Inst*. 1988;80:21–29. doi: 10.1093/jnci/80.1.21.
Folkesson 2005	Excluded	WRONG INTERVENTION	No preoperative therapy in control group	Folkesson J, Birgisson H, Pahlman L, et al. Swedish Rectal Cancer Trial: long lasting benefits from radiotherapy on survival and local recurrence rate. *J Clin Oncol*. 2005;23:5644 –5650. doi: 10.1200/JCO.2005.08.144.
Gao 2014	Excluded	WRONG DESIGN	Single arm	Gao YH, An X, Sun WJ et al. Evaluation of capecitabine and oxaliplatin administered prior to and then concomitant to radiotherapy in high risk locally advanced rectal cancer. *J Surg Oncol*. 2014 Apr;109(5):478-82. doi: 10.1002/jso.23516.
Gao 2014	Excluded	WRONG DESIGN	Single arm	Gao YH, Lin JZ, An X et al. Neoadjuvant sandwich treatment with oxaliplatin and capecitabine administered prior to, concurrently with, and following radiation therapy in locally advanced rectal cancer: a prospective phase 2 trial. *Int J Radiat Oncol Biol Phys*. 2014 Dec 1;90(5):1153-60. doi: 10.1016/j.ijrobp.2014.07.021.
Garcia-Aguilar 2015	Excluded	WRONG DESIGN	Non randomized	Garcia-Aguilar J, Chow OS, Smith DD, et al. Effect of adding mFOLFOX6 after neoadjuvant chemoradiation in locally advanced rectal cancer: A multicentre, phase 2 trial. *Lancet Oncol*. 2015;16(8):957-966. doi:10.1016/S1470-2045(15)00004-2
Garcia-Aguilar 2022	Excluded	WRONG INTERVENTION	Non-operative management	Garcia-Aguilar J, Patil S, Gollub MJ et al. Organ Preservation in Patients With Rectal Adenocarcinoma Treated With Total Neoadjuvant Therapy. *J Clin Oncol*. 2022 Aug 10;40(23):2546-2556. doi: 10.1200/JCO.22.00032.
Gerard 1988	Excluded	WRONG INTERVENTION	No preoperative therapy in control group	Gérard A, Buyse M, Nordlinger M et al. Preoperative radiotherapy as adjuvant treatment in rectal cancer. Final results of a randomized study of the European Organization for Research and Treatment of Cancer (EORTC). *Ann Surg*. 1988 Nov;208(5):606-14. doi: 10.1097/00000658-198811000-00011.
Gerard 2023	Excluded	WRONG DESIGN, WRONG COMPARISON	Wrong schedule of treatment, non-operative management	Gerard JP, Barbet N, Schiappa R et al. Neoadjuvant chemoradiotherapy with radiation dose escalation with contact x-ray brachytherapy boost or external beam radiotherapy boost for organ preservation in early cT2-cT3 rectal adenocarcinoma (OPERA): a phase 3, randomised controlled trial. *Lancet Gastroenterol Hepatol*. 2023 Apr;8(4):356-367. doi: 10.1016/S2468-1253(22)00392-2
Goldberg 1994	Excluded	WRONG INTERVENTION	No preoperative therapy in control group	Goldberg PA, Nicholls RJ, Porter NH, et al. Long-term results of a randomised trial of short-course low-dose adjuvant pre-operative radiotherapy for rectal cancer: reduction in local treatment failure. *Eur J Cancer.* 1994;30A:1602–1606. doi: 10.1016/0959-8049(94)00312-s.
Golo 2018	Excluded	WRONG DESIGN	Single arm	Golo D, But-Hadzic J, Anderluh F et al. Induction chemotherapy, chemoradiotherapy and consolidation chemotherapy in preoperative treatment of rectal cancer - long-term results of phase II OIGIT-01 Trial. *Radiol Oncol*. 2018 Sep 11;52(3):267-274. doi: 10.2478/raon-2018-0028.
Guillem 2005	Excluded	WRONG DESIGN	Single arm	Guillem JG, Chessin DB, Cohen AM et al. Long-term oncologic outcome following preoperative combined modality therapy and total mesorectal excision of locally advanced rectal cancer. *Ann Surg*. 2005 May;241(5):829-36; discussion 836-8. doi: 10.1097/01.sla.0000161980.46459.96.
Hartley 2005	Excluded	WRONG DESIGN	Pooled analysis	Hartley A, Ho KF, McConkey C, Geh JI. Pathological complete response following pre-operative chemoradiotherapy in rectal cancer: analysis of phase II/III trials. *Br J Radiol*. 2005 Oct;78(934):934-8. doi: 10.1259/bjr/86650067.
Helbling 2012	Excluded	WRONG INTERVENTION	Monoclonal antibody	Helbling D, Bodoky G, Gautschi O et al. Neoadjuvant chemoradiotherapy with or without panitumumab in patients with wild-type KRAS, locally advanced rectal cancer (LARC): a randomized, multicenter, phase II trial SAKK 41/07. *Ann Oncol*. 2013 Mar;24(3):718-25. doi: 10.1093/annonc/mds519.

© 2024 Turri G et al. *JAMA Network Open*.

Hofheinz 2012	Excluded	WRONG INTERVENTION	Comparison of two chemotherapy regimens	Hofheinz RD, Wenz F, Post S et al. Chemoradiotherapy with capecitabine versus fluorouracil for locally advanced rectal cancer: a randomised, multicentre, non-inferiority, phase 3 trial. *Lancet Oncol*. 2012 Jun;13(6):579-88. doi: 10.1016/S1470-2045(12)70116-X.
Jung 2015	Excluded	WRONG INTERVENTION	Comparison of two chemotherapy regimens	Jung M, Shin SJ, Koom WS et al. A Randomized Phase 2 Study of Neoadjuvant Chemoradiaton Therapy With 5-Fluorouracil/Leucovorin or Irinotecan/S-1 in Patients With Locally Advanced Rectal Cancer. *Int J Radiat Oncol Biol Phys*. 2015 Dec 1;93(5):1015-22. doi: 10.1016/j.ijrobp.2015.08.037.
Kapiteijn 2001	Excluded	WRONG COMPARISON	No preoperative therapy in control group	Kapiteijn E, Marijnen CA, Nagtegaal ID, et al. Preoperative radiotherapy combined with total mesorectal excision for resectable rectal cancer. *N Engl J Med*. 2001;345:638–646. doi: 10.1056/NEJMoa010580.
Kayal 2014	Excluded	WRONG INTERVENTION	Comparison of two chemotherapy regimens	Kayal PK, Saha A, Dastidar AG, Mahata A, Das A, Sarkar R. A randomized comparative study between neoadjuvant 5-fluorouracil and leukovorin versus 5-fluorouracil and cisplatin along with concurrent radiation in locally advanced carcinoma rectum. *Clin Cancer Investig J* 2014;3:32-7.
Lefevre 2016	Excluded	WRONG INTERVENTION	Wrong schedule of treatment	Lefevre JH, Mineur L, Kotti S et al. Effect of Interval (7 or 11 weeks) Between Neoadjuvant Radiochemotherapy and Surgery on Complete Pathologic Response in Rectal Cancer: A Multicenter, Randomized, Controlled Trial (GRECCAR-6). *J Clin Oncol*. 2016 Nov 1;34(31):3773-3780. doi: 10.1200/JCO.2016.67.6049.
Marco 2018	Excluded	WRONG DESIGN	Not randomized	Marco MR, Zhou L, Patil S, et al. Consolidation mFOLFOX6 chemotherapy after chemoradiotherapy improves survival in patients with locally advanced rectal cancer: Final results of a multicenter phase II trial. *Dis Colon Rectum*. 2018;61(10):1146-1155. doi:10.1097/DCR.0000000000001207
Markovina 2017	Excluded	WRONG DESIGN	Not randomized	Markovina S, Youssef F, Roy A, et al. Improved Metastasis- and Disease-Free Survival With Preoperative Sequential Short-Course Radiation Therapy and FOLFOX Chemotherapy for Rectal Cancer Compared With Neoadjuvant Long-Course Chemoradiotherapy: Results of a Matched Pair Analysis. In: *International Journal of Radiation Oncology Biology Physics*. Vol 99. Int J Radiat Oncol Biol Phys; 2017:417-426. doi:10.1016/j.ijrobp.2017.05.048
Masi 2019	Excluded	WRONG DESIGN	Single arm	Masi G, Vivaldi C, Fornaro L et al. Total neoadjuvant approach with FOLFOXIRI plus bevacizumab followed by chemoradiotherapy plus bevacizumab in locally advanced rectal cancer: the TRUST trial. *Eur J Cancer*. 2019 Mar;110:32-41. doi: 10.1016/j.ejca.2019.01.006.
NCT02514278	Excluded	WRONG INTERVENTION	Non-operative management, ongoing	Optimisation of Response for Organ Preservation in Rectal Cancer : Neoadjuvant Chemotherapy and Radiochemotherapy vs. Radiochemotherapy (GRECCAR12). ClinicalTrials.gov Identifier: NCT02514278.
NCT02945566	Excluded	WRONG INTERVENTION , WRONG POPULATION	Non-operative management, early rectal cancer	Can the Rectum be Saved by Watchful Waiting or TransAnal Surgery Following (Chemo)Radiotherapy Versus Total Mesorectal Excision for Early REctal Cancer? (STAR-TREC). ClinicalTrials.gov Identifier: NCT02945566.
NCT04246684	Excluded	WRONG INTERVENTION	Non-operative management, ongoing	Short RT Versus RCT,Followed by Chemo.and Organ Preservation for Interm and High-risk Rectal Cancer Patients. ClinicalTrials.gov Identifier: NCT04246684
Peeters 2007	Excluded	WRONG DESIGN	No preoperative therapy in control group	Peeters KCMJ, Marijnen CAM, Nagtegaal ID et al. The TME trial after a median follow-up of 6 years: increased local control but no survival benefit in irradiated patients with resectable rectal carcinoma. *Ann Surg*. 2007 Nov;246(5):693-701. doi: 10.1097/01.sla.0000257358.56863.ce.
Pettersson 2015	Excluded	WRONG POPULATION	Exclusion of locally-advanced rectal cancer	Pettersson D, Lörinc E, Holm T et al. Tumour regression in the randomized Stockholm III Trial of radiotherapy regimens for rectal cancer. *Br J Surg*. 2015 Jul;102(8):972-8; discussion 978. doi: 10.1002/bjs.9811.
Rahma 2021	Excluded	WRONG INTERVENTION	Immunotherapy	Rahma OE, Yothers G, Hong TS et al. Use of Total Neoadjuvant Therapy for Locally Advanced Rectal Cancer: Initial Results From the Pembrolizumab Arm of a Phase 2 Randomized Clinical Trial. *JAMA Oncol*. 2021 Aug 1;7(8):1225-1230. doi: 10.1001/jamaoncol.2021.1683.
Saha 2015	Excluded	WRONG DESIGN	Pilot study	Saha A, Ghosh SK, Roy C et al. A randomized controlled pilot study to compare capecitabine-oxaliplatin with 5-FU-leucovorin as neoadjuvant concurrent chemoradiation in locally advanced adenocarcinoma of rectum. *J Cancer Res Ther*. 2015 Jan-Mar;11(1):88-93. doi: 10.4103/0973-1482.150341.
Sauer 2012	Excluded	WRONG INTERVENTION	Post-operative chemioradiotherapy	Sauer R, Liersch T, Merkel S et al. Preoperative versus postoperative chemoradiotherapy for locally advanced rectal cancer: results of the German CAO/ARO/AIO-94 randomized phase III trial after a median follow-up of 11 years. *J Clin Oncol*. 2012 Jun 1;30(16):1926-33. doi: 10.1200/JCO.2011.40.1836.
Siegel 2009	Excluded	NO FINAL DATA REPORTED	No final data reported, only protocol published	Siegel R, Burock S, Wernecke KD et al. Preoperative short-course radiotherapy versus combined radiochemotherapy in locally advanced rectal cancer: a multi-centre prospectively randomised study of the Berlin Cancer Society. *BMC Cancer*. 2009 Feb 6;9:50. doi: 10.1186/1471-2407-9-50.

© 2024 Turri G et al. *JAMA Network Open*.

Smith 2015	Excluded	WRONG INTERVENTION, WRONG COMPARISON	Not randomized, non-operative management	Smith JJ, Chow OS, Gollub MJ et al. Organ Preservation in Rectal Adenocarcinoma: a phase II randomized controlled trial evaluating 3-year disease-free survival in patients with locally advanced rectal cancer treated with chemoradiation plus induction or consolidation chemotherapy, and total mesorectal excision or nonoperative management. *BMC Cancer* .2015 Oct 23;15:767. doi: 10.1186/s12885-015-1632-z.
Tveit 1997	Excluded	WRONG INTERVENTION	Post-operative chemioradiotherapy	Tveit KM, Guldvog I, Hagen S, et al. Randomized controlled trial of postoperative radiotherapy and short-term time-scheduled 5-fluorouracil against surgery alone in the treatment of Dukes B and C rectal cancer. Norwegian Adjuvant Rectal Cancer Project Group. *Br J Surg*. 1997;84:1130–1135.
Wang 2018	Excluded	WRONG INTERVENTION	No preoperative therapy in control group	Wang F, Fan W, Peng J et al. Total mesorectal excision with or without preoperative chemoradiotherapy for resectable mid/low rectal cancer: a long-term analysis of a prospective, single-center, randomized trial. *Cancer Commun (Lond)*. 2018 Dec 20;38(1):73. doi: 10.1186/s40880-018-0342-8.
Wang 2018	Excluded	WRONG DESIGN	Single arm	Wang X, Yu Y, Meng W et al. Total neoadjuvant treatment (CAPOX plus radiotherapy) for patients with locally advanced rectal cancer with high risk factors: A phase 2 trial. *Radiother Oncol*. 2018 Nov;129(2):300-305. doi: 10.1016/j.radonc.2018.08.027.
Wong 2012	Excluded	WRONG INTERVENTION	Comparison of two chemotherapy regimens	Wong SJ, Winter K, Meropol NJ et al. Radiation Therapy Oncology Group 0247: a randomized Phase II study of neoadjuvant capecitabine and irinotecan or capecitabine and oxaliplatin with concurrent radiotherapy for patients with locally advanced rectal cancer. *Int J Radiat Oncol Biol Phys*. 2012 Mar 15;82(4):1367-75. doi: 10.1016/j.ijrobp.2011.05.027.
Wiśniowska 2016	Excluded	WRONG DESIGN	Subgroup analysis of RCT	Wiśniowska K, Nasierowska-Guttmejer A, Polkowski W et al. Does the addition of oxaliplatin to preoperative chemoradiation benefit cT4 or fixed cT3 rectal cancer treatment? A subgroup analysis from a prospective study. *Eur J Surg Oncol*. 2016 Dec;42(12):1859-1865. doi: 10.1016/j.ejso.2016.08.001.
Wolmark 2000	Excluded	WRONG INTERVENTION	Post-operative chemioradiotherapy	Wolmark N, Wieand HS, Hyams DM, et al. Randomized trial of postoperative adjuvant chemotherapy with or without radiotherapy for carcinoma of the rectum: National Surgical Adjuvant Breast and Bowel Project Protocol R-02. *J Natl Cancer Inst*. 2000;92:388–396.
Zhu 2013	Excluded	WRONG DESIGN	Single arm	Zhu J, Gu W, Lian P et al. A phase II trial of neoadjuvant IMRT-based chemoradiotherapy followed by one cycle of capecitabine for stage II/III rectal adenocarcinoma. *Radiat Oncol*. 2013 May 29;8:130. doi: 10.1186/1748-717X-8-130.

© 2024 Turri G et al. *JAMA Network Open*.

eTable 2. Characteristics of Included Studies

Legend: pCR=pathologic complete response; AV distance=distance from the anal verge; L-CRT1=long-course chemoradiotherapy with single-agent fluoropyrimidine-based chemotherapy; L-CRT2=long-course chemoradiotherapy with duplex chemotherapy drug (fluoropyrimidine plus oxaliplatin); FU=fluorouracil; OXA=oxaliplatin; Cape=capecitabine; S-RT=short-course radiotherapy; L-RT=long-course radiotherapy; FU-LV=fluorouracil plus leucovorin; NR=not reported; XELOX/CapOX=capecitabine plus oxaliplatin; FOLFIRONOX=oxaliplatin, irinotecan, FU-LV; mFOLFOX6= leucovorin 400 mg/m2 intravenously followed by fluorouracil 400 mg/m2 intravenously and fluorouracil 2.4 g/m2 by 48-h continuous intravenous infusion plus oxaliplatin 85 mg/m^2 on day 1 of each chemotherapy cycle; CHT=chemotherapy. Percentages are computed on the intention-to-treat population.

First author	Year	Number of patients	Treatment control arm	Treatment experimental arm	Women (%)	Mean age	Mean AV distance (cm) / Range (%)	Clinical stage exp. arm (%)	pCR exp. arm (%)	R0 exp. arm (%)	ypN0 exp. arm (%)	ypT1-2 exp. arm (%)	Mean follow-up (months)
Aschele	2011	739	L-RT (50.4 Gy) + FU (L-CRT1)	L-RT (50.4 Gy) + OXA + FU (L-CRT2)	33.0	62.5	Max 12 cm<br><8 cm: 76.9	cT3-4: 95.1<br>cN+: 66.8	16.0	91.0	67.1	35.1	105.6
Bahadoer	2021	912	L-RT (50.4 Gy) + Cape (L-CRT1)	S-RT (25 Gy) → CAPOX x 6 / FOLFOX4 x 9 (S-RT + consolidation)	32.6	61.0	Max 16 cm<br><10 cm: 61.5	cT3-4: 97.0<br>cN+: 90.9	26.0	82.7	68.6	22.3	74.4
Bosset	2005	1011	L-RT (45 Gy)	L-RT (45 Gy) + FU-LV (L-CRT1)	29.6	63.1	Max 15 cm<br><10 cm: 94.3	cT3-4: 100<br>cN+: NR	12.8	NR	67.2	40.5	124.8
Bujko	2004	312	S-RT (25 Gy) → TME (7 days) (S-RTearly)	L-RT (50.4 Gy) + FU-LV (L-CRT1)	35.0	60.0	5.7	cT3-4: NR<br>cN+: NR	14.0	82.8	68.0	39.5	48.0
Bujko	2013	515	L-RT (50.4 Gy) + OXA + FU-LV (L-CRT2)	S-RT (25 Gy) → FOLFOX4 x 3 (S-RT + consolidation)	31.5	60.0	Max 15 cm<br><10 cm: 97.3	cT3-4: 96.9<br>cN+: NR	16.0	77.3	57.5	19.1	35.0
Chakrabarti	2021	140	L-RT (50.4 Gy) + Cape (L-CRT1)	S-RT (25 Gy) → XELOX x 2 (S-RT + consolidation)	33.6	42.0	NR	cT3-4: 100<br>cN+: 68.1	11.6	86.9	75.3	66.7	NR
Conroy	2021	461	L-RT (50 Gy) + Cape (L-CRT1)	FOLFIRINOX x 6 → L-RT + Cape (Induction + L-CRT)	33.6	61.5	Max 15 cm<br><10 cm: 87%	cT3-4: 96.1<br>cN+: 89.6	25.5	87.0	75.8	30.7	47.9
Deng	2016	475	L-RT (46-50.4 Gy) + FU-LV (L-CRT1)	L-RT (46-50.4 Gy) + mFOLFOX6 (L-CRT2)	34.3	54.0	5.4	cT3-4: 98.2<br>cN+:81.8	25.4	81.2	50.9	NR	45.1
				mFOLFOX6 x 4-6 (CHT)	34.3	54.0	6	cT3-4: 99.4<br>cN+:72.1	6.1	82.4	32.7	NR	45.1
Fernández-Martos	2015	103	L-RT + Cape + OXA (L-CRT2)	XELOX x 4 → L-RT + Cape + OXA (Induction + L-CRT)	34.0	61.0	Max 12 cm	cT3-4: NR<br>cN+: NR	14.3	85.7	67.9	NR	69.0
Fokas	2019	306	-	FOLFOX x 3 → L-RT (50.4 Gy) + FU + OXA (Induction + L-CRT)	32	62.0	Max 12 cm<br><10 cm: 84.0	cT3-4: 96.2<br>cN+: 85.9	17.3	83.3	67.3	37.2	43.0
				L-RT (50.4 Gy) + FU + OXA → FOLFOX x 3 (L-CRT + consolidation)	33	61.0	Max 12 cm<br><10 cm: 90.0	cT3-4: 96.7<br>cN+: 90.0	25.3	85.3	74.0	27.3	43.0
Gérard	2006	733	L-RT (45 Gy)	L-RT (45 Gy) + FU-LV (L-CRT1)	33.5	63.5	NR	cT3-4: 98.4<br>cN+: NR	10.9	90.1	67.3	29.9	81.0

© 2024 Turri G et al. *JAMA Network Open*.

Gérard	2010	598	L-RT (45 Gy) + Cape (L-CRT1)	L-RT (45 Gy) + CapOX (L-CRT2)	46.0	62.0	NR	cT3-4: 92.7 cN+: 72.5	18.9	44.0 (44.0% missing)	69.1	30.5	60.2
Haddad	2017	63	L-RT (50-50.4 Gy) + Cape (L-CRT1)	L-RT (50-50.4 Gy) + CapOX (L-CRT2)	31.7	57.0	6.2	cT3-4: 97.0 cN+: 93.5	34.0	NR	NR	NR	NR
Jiao	2015	206	L-RT (50 Gy) + Cape (L-CRT1)	L-RT (50 Gy) + XELOX (L-CRT2)	44.2	55.8	Max 12 cm <8 cm: 79.6	cT3-4: 98.1 cN+: 78.6	23.3	97.1	71.8	39.8	48.7
Jin	2022	599	L-RT (50 Gy) + Cape (L-CRT1)	S-RT (25 Gy) → CapOX x 4 (S-RT + consolidation)	29.0	55.5	Max 10 cm <5 cm: 49.3	cT3-4: 98.9 cN+: 86.9	13.1	72.1	56.0	27.5	35.0
Kim	2018	110	L-RT (50.4 Gy) + Cape (L-CRT1)	L-RT (50.4 Gy) + Cape → CapOX x 2 (L-CRT + consolidation)	24.0	55.5	5.5	cT3-4: 100 cN+: 92.5	11.3	90.6	52.8	24.5	NR
Latkauskas	2012	150	S-RT (25 Gy) → TME (6 weeks) (S-RTdelayed)	L-RT (50 Gy) + FU-LV (L-CRT1)	33.6	64.0	Max 15 cm <10 cm: 89.3	cT3-4: NR cN+: 76.0	10.6	85.3	72.0	32	39.7
Maréchal	2012	57	L-RT (45 Gy) + FU (L-CRT1)	mFOLFOX6 x 2 → L-RT (45 Gy) + FU (Induction + L-CRT)	35.0	62.0	NR	cT3-4: 96.4 cN+: NR	25.0	96.4	46.4	21.4	NR
Mei	2023	589	L-RT (50 Gy) + Cape (L-CRT1)	CapOX x 4 (CHT)	38.0	60.0	Max 12 cm <10 cm: 96.6	cT3-4: 94.7 cN+: 69.3	10.0	90.3	66.7	29.3	NR
Mohiuddin	2006	103	L-RT (45.6 Gy) + FU (L-CRT1)	L-RT (45 Gy) + Irinotecan + FU (L-CRT2)	34.0	57.0	Max 9 cm	cT3-4: 100 cN+: NR	26.4	NR	NR	NR	NR
Moore	2017	49	L-RT (50.4 Gy) + FU (L-CRT1)	L-RT (50.4 Gy) + FU → FU x 3 (L-CRT + consolidation)	26.5	60.0	6.3	cT3-4: 100 cN+: 100	16.0	92.0	64.0	24.0	NR
Ngan	2012	323	S-RT (25 Gy) → TME (7 days) (S-RTearly)	L-RT (50.4 Gy) + FU (L-CRT1)	27.0	63.5	6.6	cT3-4: 100 cN+: 37.9	14.9	93.7	63.3	29.2	70.8
O'Connell	2014	1276	L-RT (50.4 Gy) + FU or Cape (L-CRT1)	L-RT (50.4 Gy) + FU + Oxa or CapOX (L-CRT2)	32.0	NR	Max 12 cm	cT3-4: NR cN+: NR	19.5	NR	NR	NR	54
Rodel	2015	1232	L-RT (50.4 Gy) + FU (L-CRT1)	L-RT (50.4 Gy) + FU + OXA (L-CRT2)	29.0	62.0	Max 12 cm <10 cm: 89.8	cT3-4: 96.2 cN+: 73.7	17.0	92.3	67.9	32.9	49.6
Schmoll	2021	1068	L-RT (45-50.4 Gy) + Cape (L-CRT1)	L-RT (45-50.4 Gy) + CapOX (L-CRT2)	29.2	62	Max 12 cm ≤5 cm: 45.1	cT3-4: 97.5 cN+: 74.1	14.0	95.8	NR	NR	64.0
Schrag	2023	1128	L-RT (50.4 Gy) + FU or Cape (L-CRT1)	mFOLFOX6 x 6 (CHT) (+ selective L-CRT in 53 patients)	34.5	57.2	8.6	cT3-4: 89.0 cN+: 60.2	20.0	90.4	68.4	40.8	58.0
Wang	2019	120	L-RT (50 Gy) + CapOX (L-CRT2)	L-RT (50 Gy + 5 Gy boost) + CapOX → XELOX (L-CRT + consolidation)	30.0	NR	Max 12 cm ≤5 cm: 66.7	cT3-4: 100 cN+: 76.7	23.3	NR	65.0	40.0	42.0

© 2024 Turri G et al. *JAMA Network Open*.

eTable 3. Summary of Inclusion and Exclusion Criteria of Included Studies

First author	Year	cT2	cT3	cT4	cN+	Threathened/involved MRF	EMVI +	Recurrent cancers	Distance from AV
Aschele	2011						Not specified		12 cm
Bahadoer	2021								16 cm
Bosset	2005				Not specified	Not specified	Not specified		15 cm
Bujko	2004				Not specified	Not specified	Not specified		Inferior margin palpable on DRE
Bujko	2013				Not specified		Not specified		15 cm
Chakrabarti	2021					Not specified	Not specified		Mid-lower rectum
Conroy	2021					Not specified	Not specified		15 cm
Deng	2016					Not specified	Not specified		12 cm
Fernández-Martos	2015					Not specified	Not specified		12 cm
Fokas	2019					Not specified			12 cm
Gérard	2006				Not specified	Not specified	Not specified		Accessible to DRE
Gérard	2010				Not specified	Not specified	Not specified		Accessible to DRE
Haddad	2017					Not specified	Not specified		15 cm
Jiao	2015					Not specified	Not specified		12 cm
Jin	2022					Not specified	Not specified		Mid-lower rectum
Kim	2018				Not specified	Not specified	Not specified		12 cm
Latkauskas	2012					Not specified	Not specified		15 cm
Maréchal	2012					Not specified	Not specified		Not specified
Mei	2023						Not specified		12 cm
Mohiuddin	2006				Not specified	Not specified	Not specified		9 cm
Moore	2017								12 cm
Ngan	2012					Not specified	Not specified		12 cm
O'Connell	2014					Not specified	Not specified		12 cm
Rodel	2015					Not specified	Not specified		12 cm
Schmoll	2021					Not specified	Not specified		12 cm
Schrag	2023						Not specified		12 cm
Wang	2019					Not specified	Not specified		12 cm

© 2024 Turri G et al. *JAMA Network Open*.

eFigure 2. Risk of bias of included studies

Risk of Bias Summary

Total number of studies = 27

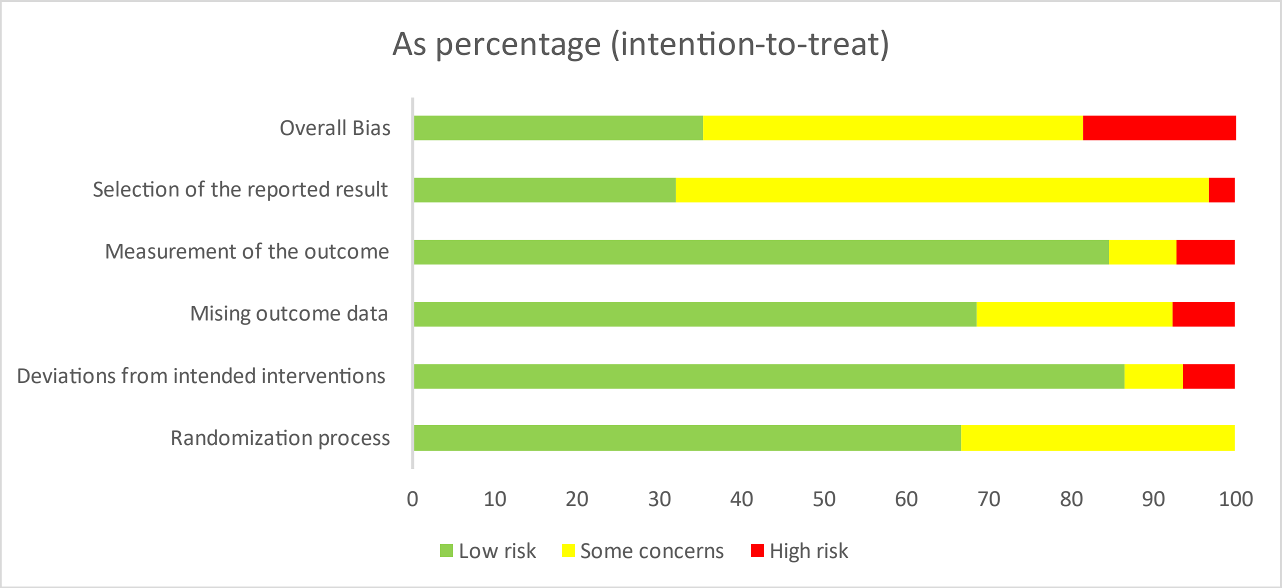

	**Randomization process**	**Deviations from intended interventions**	**Missing outcome data**	**Measurement of the outcome**	**Selection of the reported result**	**Overall Bias**
Low risk	66,7%	92,5%	92,5%	74,1%	63%	33,3%
Some concerns	33,3%	0%	3,7%	25,9%	7%	55,6%
High risk	0%	7,5%	3,7%	0%	0%	11,1%

© 2024 Turri G et al. *JAMA Network Open*.

Risk of Bias Graph

Unique ID	Study ID	Experimental	Comparator	Outcome	Weight	D1	D2	D3	D4	D5	Overall
Aschele 2011	1	NA	NA	NA	NA	+	+	+	+	!	!
Bahadoer 2021	2	NA	NA	NA	NA	+	+	+	+	+	+
Bosset 2005	3	NA	NA	NA	NA	+	+	+	+	+	+
Bujko 2004	4	NA	NA	NA	NA	+	+	+	!	!	!
Bujko (2) 2013	5	NA	NA	NA	NA	+	+	+	!	+	!
Chakrabarty 2(	6	NA	NA	NA	NA	!	+	+	+	!	!
Conroy 2021	7	NA	NA	NA	NA	+	+	+	+	+	+
Deng 2016	8	NA	NA	NA	NA	+	+	+	!	+	!
Fernandez-Ma	9	NA	NA	NA	NA	!	+	+	!	+	!
Fokas 2019	10	NA	NA	NA	NA	+	+	+	+	+	+
Gerard 2006	11	NA	NA	NA	NA	+	+	+	+	!	!
Gerard (2) 201	12	NA	NA	NA	NA	+	+	+	+	!	!
Haddad 2017	13	NA	NA	NA	NA	!	+	-	+	!	-
Jiao 2015	14	NA	NA	NA	NA	+	+	+	+	!	!
Jin 2022	15	NA	NA	NA	NA	+	+	!	+	+	!
Kim 2018	16	NA	NA	NA	NA	!	+	+	+	+	!
Latkauskas 201	17	NA	NA	NA	NA	!	-	+	!	!	-
Marechal 2012	18	NA	NA	NA	NA	+	+	+	+	!	!
Mei 2023	19	NA	NA	NA	NA	+	+	+	+	+	+
Mohiuddin 20(	20	NA	NA	NA	NA	!	+	+	+	!	!
Moore 2017	21	NA	NA	NA	NA	+	+	+	+	+	+
Ngan 2012	22	NA	NA	NA	NA	!	+	+	!	+	!
O'Connell 201	23	NA	NA	NA	NA	!	+	+	+	+	!
Rodel 2015	24	NA	NA	NA	NA	+	+	+	+	+	+
Schmoll 2021	25	NA	NA	NA	NA	+	+	+	+	+	+
Schrag 2023	27	NA	NA	NA	NA	!	-	+	!	+	-
Wang 2019	26	NA	NA	NA	NA	+	+	+	+	+	+

+ Low risk
! Some concerns
- High risk

D1 Randomisation process
D2 Deviations from the intended interventions
D3 Missing outcome data
D4 Measurement of the outcome
D5 Selection of the reported result

© 2024 Turri G et al. *JAMA Network Open*.

eFigure 3. Transitivity Assessment and Meta-Regression

Continuous variables

We represented the distribution of the variable within each treatment strategy as a boxplots and performed the Kruskal-Wallis equality-of-populations rank test. The results of the meta-regression analyses are also reported.

<table>
<tr><th>Potential effect modifiers</th><th>Boxplot</th><th>Kruskal-Wallis equality-of-populations rank test</th><th>Meta-regression analysis</th></tr>
<tr><td>Year of publication</td><td>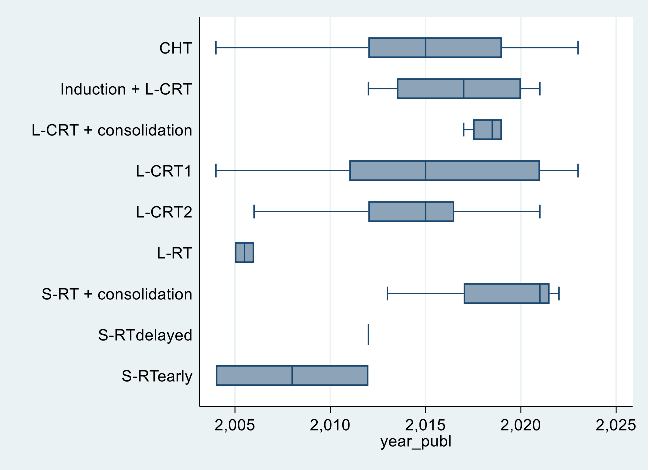
</td><td>chi-squared with ties = 15.938 with 8 d.f. p = 0.0433</td><td>Coeff. = -0.1293549
SE = 0.093929
p = 0.168</td></tr>
<tr><td>Mean follow-up length (weeks)</td><td>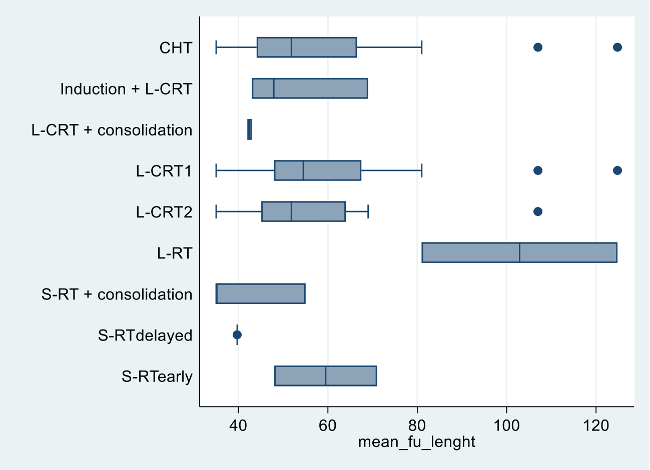
</td><td>chi-squared with ties = 11.771 with 8 d.f. p = 0.1617</td><td>Coeff. = -0.034571
SE = 0.7435485
p = 0.963</td></tr>
</table>

© 2024 Turri G et al. *JAMA Network Open*.

Mean age	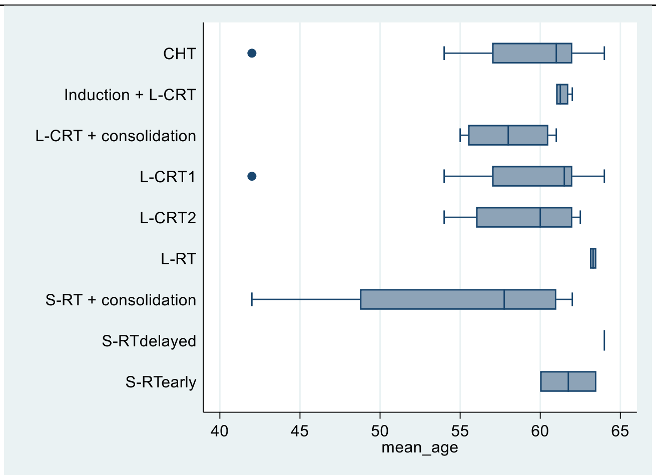	chi-squared with ties = 12.639 with 8 d.f. p = 0.1249	Coeff. = - 0.1482965 SE = 0.1358809 p = 0.275
Proportion of female participants	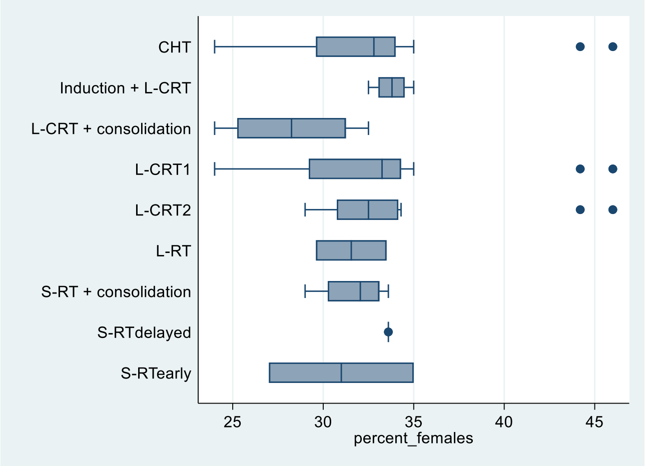	chi-squared with ties = 10.355 with 8 d.f. p = 0.2410	Coeff. = - 0.1126564 SE = 0.1946762 p = 0.563
Proportion of cT4	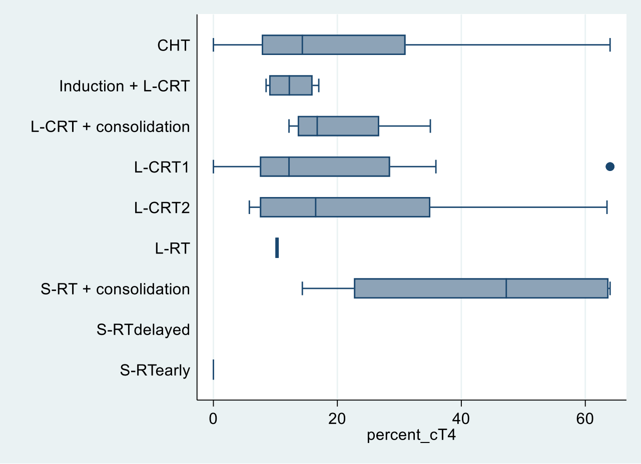	chi-squared with ties = 8.001 with 7 d.f. p = 0.3325	Coeff. = 0.0206261 SE = 0.0198416 p = 0.299

© 2024 Turri G et al. *JAMA Network Open*.

Proportion of cN+	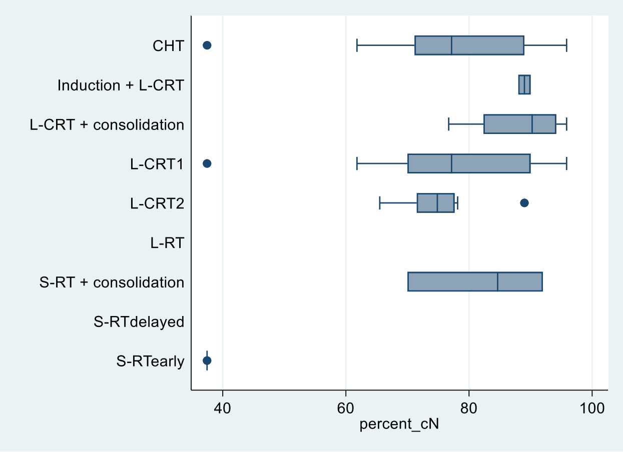	chi-squared with ties = 9.388 with 7 d.f.<br>p = 0.1529	Coeff. = 0.0552351<br>SE = 0.0434548<br>p = 0.204
Mean AV distance	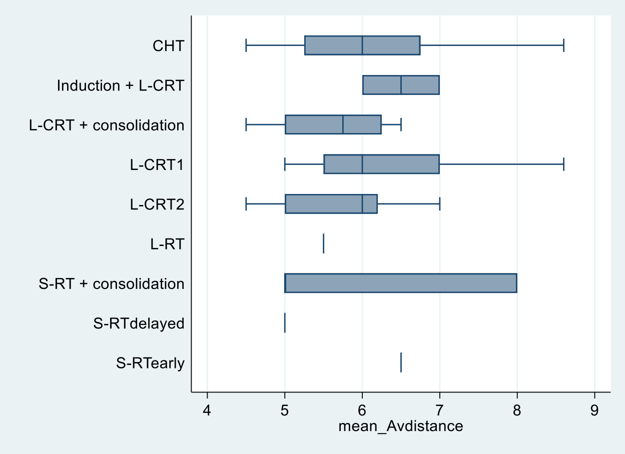	chi-squared with ties = 6.145 with 8 d.f.<br>p = 0.6309	Coeff. = -0.2150342<br>SE = 0.2321012<br>p = 0.354
Proportion of pT4	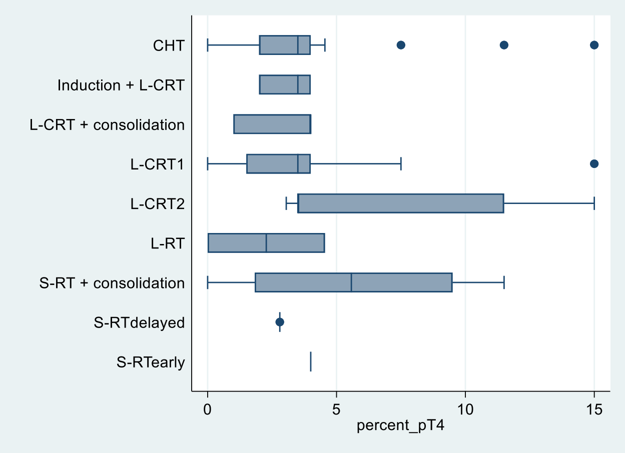	chi-squared with ties = 2.364 with 8 d.f.<br>p = 0.9678	Coeff. = 0.0070797<br>SE = 0.0613205<br>p = 0.908

© 2024 Turri G et al. *JAMA Network Open*.

eAppendix 2. Primary Outcome: Patients With Pathologic Complete Response (PCR)

Characteristics of the network

Number of treatments:

9

Number of studies:

27

Number of individuals included:

13413

Number of individuals randomized to each treatment:

	Treatment name	N. individuals randomized
1	CHT	1050
2	Induction + L-CRT	469
3	L-CRT + consolidation	288
4	L-CRT1	6084
5	L-CRT2	3169
6	L-RT	872
7	S-RT + consolidation	1090
8	S-RTdelayed	75
9	S-RTearly	316

Pairwise meta-analysis
Risk ratios above 1 favor the first treatment of the comparison.

© 2024 Turri G et al. *JAMA Network Open*.

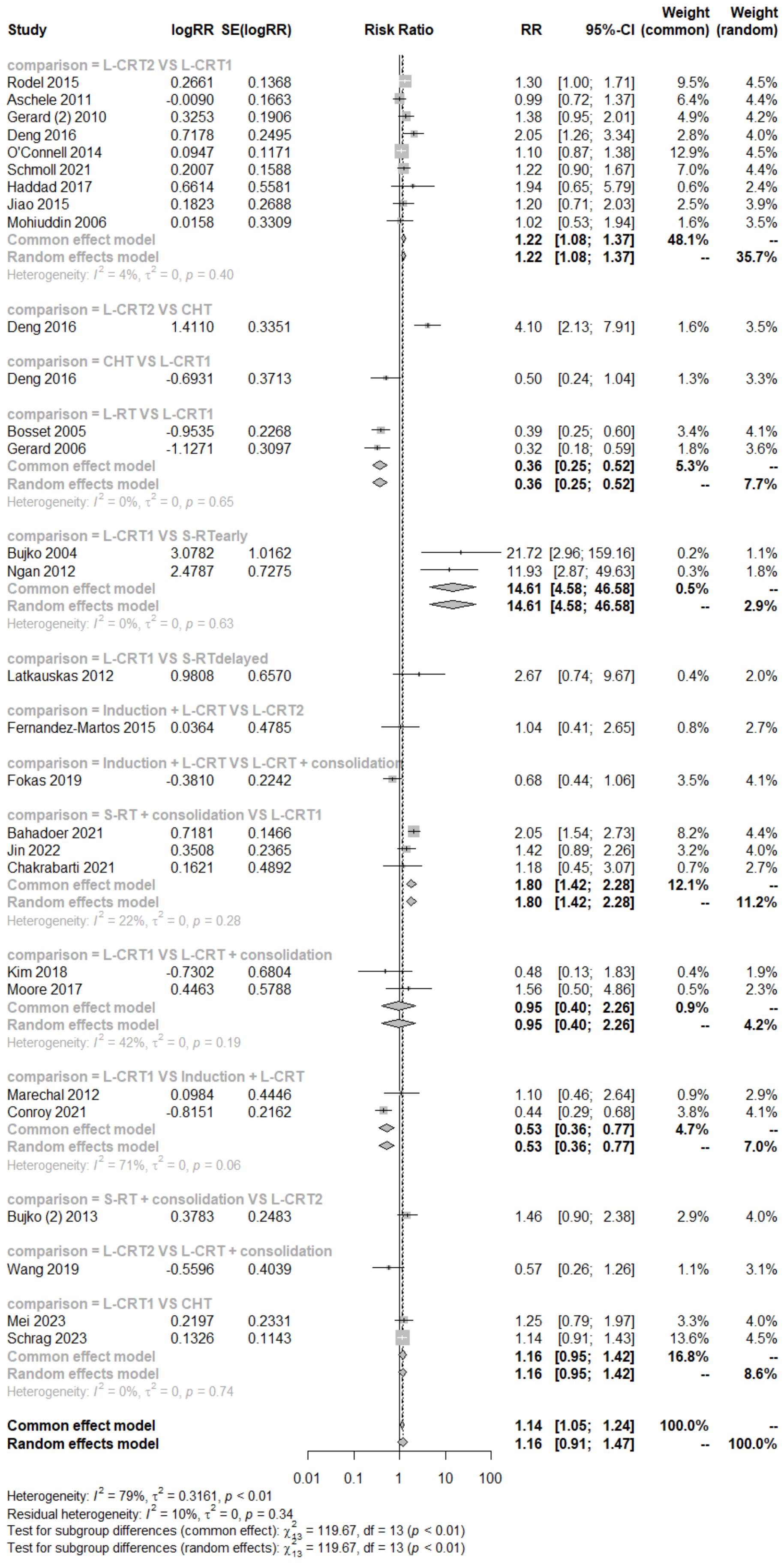

© 2024 Turri G et al. *JAMA Network Open*.

Network map The thickness of lines is proportional to the number of studies comparing the two treatments and the size of circles is proportional to the number of individuals for each treatment.

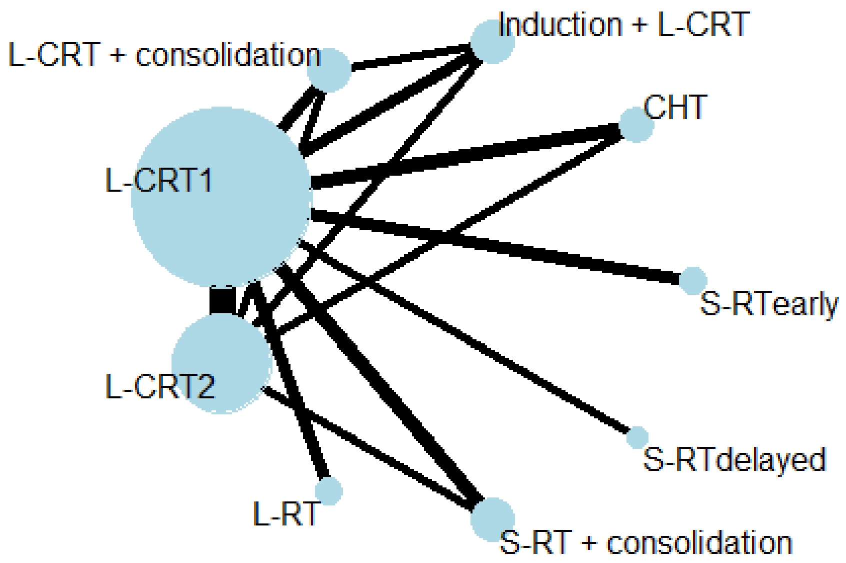

Netleague table Relative risks (RRs) and 95% confidence intervals (CIs) are reported. Results of the network meta-analysis are reported in the lower left part of the table and results from the pairwise meta-analysis are reported in the upper right part of the table. RRs higher than 1 favor the column-defining treatment.

	V1	V2	V3	V4	V5	V6	V7	V8	V9
1	CHT	.	.	0.80 (0.61 to 1.05)	0.24 (0.12 to 0.50)	.	.	.	.
2	0.48 (0.30 to 0.75)	Induction + L-CRT	0.68 (0.41 to 1.15)	1.83 (1.17 to 2.84)	1.04 (0.39 to 2.75)	.	.	.	.
3	0.38 (0.23 to 0.65)	0.80 (0.53 to 1.22)	L-CRT + consolidation	1.05 (0.43 to 2.55)	1.75 (0.76 to 4.04)	.	.	.	.
4	0.75 (0.57 to 0.98)	1.57 (1.09 to 2.25)	1.95 (1.25 to 3.06)	L-CRT1	0.81 (0.69 to 0.94)	2.77 (1.84 to 4.18)	0.58 (0.43 to 0.78)	2.67 (0.71 to 9.95)	14.65 (4.51 to 47.52)
5	0.59 (0.44 to 0.80)	1.24 (0.85 to 1.80)	1.54 (0.98 to 2.43)	0.79 (0.68 to 0.92)	L-CRT2	.	0.69 (0.39 to 1.20)	.	.
6	2.07 (1.27 to 3.39)	4.34 (2.51 to 7.50)	5.41 (2.94 to 9.94)	2.77 (1.84 to 4.18)	3.52 (2.27 to 5.44)	L-RT	.	.	.
7	0.42 (0.29 to 0.62)	0.89 (0.57 to 1.39)	1.11 (0.66 to 1.87)	0.57 (0.43 to 0.74)	0.72 (0.54 to 0.96)	0.21 (0.13 to 0.34)	S-RT + consolidation	.	.
8	1.99 (0.52 to 7.64)	4.18 (1.07 to 16.36)	5.20 (1.30 to 20.91)	2.67 (0.71 to 9.95)	3.38 (0.90 to 12.72)	0.96 (0.24 to 3.82)	4.69 (1.22 to 17.97)	S-RTdelayed	.
9	10.94 (3.27 to 36.61)	22.94 (6.70 to 78.58)	28.58 (8.11 to 100.74)	14.65 (4.51 to 47.52)	18.57 (5.67 to 60.84)	5.28 (1.52 to 18.38)	25.76 (7.70 to 86.14)	5.49 (0.94 to 32.11)	S-RTearly

Forest plot L-CRT-1 was used as a common comparator. RRs above 1 favor the strategy over the common comparator (L-CRT1).

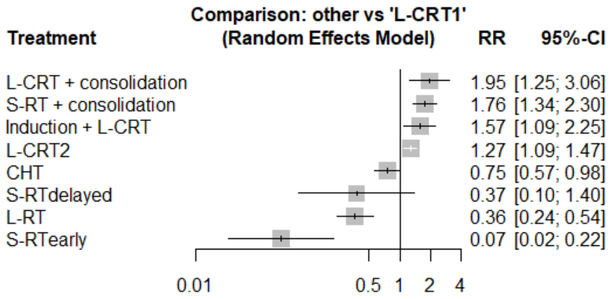

Assessment of heterogeneity and consistency

© 2024 Turri G et al. *JAMA Network Open*.

Global heterogeneity

We interpreted tau^2 as follows: heterogeneity low with tau^2≤0.010, moderate with 0.010<tau^2≤0.242, high with tau^2>0.242, and I^2 statistics as follows: not important (0%-40%), moderate (30%-60%), substantial (50%-90%), considerable (75%-100%).

```
tau^2= 0.0194 ; tau= 0.1393

I^2= 26.44 % ( 0 % to 56.84 %)
```

Consistency: global approach

Q statistic to assess consistency under the assumption of a full design-by-treatment interaction random effects model

Q statistics to assess homogeneity / consistency

	Q	df	p-value
Total	27.19	20	0.1300
Within designs	12.01	14	0.6058
Between designs	15.18	6	0.0189

Design-specific decomposition of within-designs Q statistic

Design	Q	df	p-value
L-CRT1:CHT	0.11	1	0.7373
L-CRT1:Induction + L-CRT	3.41	1	0.0646
L-CRT1:L-CRT + consolidation	1.73	1	0.1878
L-CRT1:L-CRT2	3.76	7	0.8073
L-CRT1:L-RT	0.20	1	0.6512
L-CRT1:S-RT + consolidation	2.55	2	0.2791
L-CRT1:S-RTearly	0.23	1	0.6314

Between-designs Q statistic after detaching of single designs

Detached design	Q	df	p-value
Induction + L-CRT:L-CRT + consolidation	13.59	5	0.0184
Induction + L-CRT:L-CRT2	14.92	5	0.0107
L-CRT + consolidation:L-CRT2	15.15	5	0.0097
L-CRT1:CHT	8.27	5	0.1422
L-CRT1:Induction + L-CRT	13.12	5	0.0223
L-CRT1:L-CRT + consolidation	12.23	5	0.0317
L-CRT1:L-CRT2	9.74	5	0.0828
L-CRT1:S-RT + consolidation	15.18	5	0.0096
L-CRT2:S-RT + consolidation	15.18	5	0.0096
L-CRT1:CHT:L-CRT2	3.74	4	0.4421

Q statistic to assess consistency under the assumption of a full design-by-treatment interaction random effects model

	Q	df	p-value	tau.within	tau2.within
Between designs	15.18	6	0.0189	0	0

Consistency: local approach

Separate indirect from direct evidence (SIDE) using back-calculation method
Random effects model:

comparison	k	prop	nma	direct	indir.	RoR	z	p-value
L-CRT1:CHT	3	0.97	1.34	1.25	9.82	0.13	-2.61	0.0089
CHT:L-CRT2	1	0.18	0.59	0.24	0.72	0.34	-2.68	0.0073
Induction + L-CRT:L-CRT + consolidation	1	0.67	0.80	0.68	1.11	0.62	-1.06	0.2903
L-CRT1:Induction + L-CRT	2	0.67	0.64	0.55	0.87	0.63	-1.18	0.2374

© 2024 Turri G et al. *JAMA Network Open*.

Induction + L-CRT:L-CRT2	1	0.15	1.24	1.04	1.27	0.81	-0.38	0.7037
L-CRT1:L-CRT + consolidation	2	0.26	0.51	0.95	0.41	2.29	1.58	0.1135
L-CRT + consolidation:L-CRT2	1	0.30	1.54	1.75	1.46	1.20	0.36	0.7196
L-CRT1:L-CRT2	9	0.89	0.79	0.81	0.67	1.21	0.78	0.4332
L-CRT1:S-RT + consolidation	3	0.78	0.57	0.58	0.54	1.07	0.21	0.8325
L-CRT2:S-RT + consolidation	1	0.27	0.72	0.69	0.74	0.93	-0.21	0.8325

Legend:
comparison - Treatment comparison
k - Number of studies providing direct evidence
prop - Direct evidence proportion
nma - Estimated treatment effect (RR) in network meta-analysis
direct - Estimated treatment effect (RR) derived from direct evidence
indir. - Estimated treatment effect (RR) derived from indirect evidence
RoR - Ratio of Ratios (direct versus indirect)
z - z-value of test for disagreement (direct versus indirect)
p-value - p-value of test for disagreement (direct versus indirect)

Netrank Higher p-score values indicate higher ranking of treatments

	P-score
L-CRT + consolidation	0.9319
S-RT + consolidation	0.8769
Induction + L-CRT	0.7870
L-CRT2	0.6431
L-CRT1	0.4901
CHT	0.3574
S-RTdelayed	0.2250
L-RT	0.1844
S-RTearly	0.0042

eAppendix 3. Primary Outcome: Assessment of publication bias

We assessed the risk of publication bias only for the comparisons L-CRT2 vs L-CRT1, which included 9 RCTs.

For the remaining comparisons, too few RCTs to assess publication bias were included.

Funnel plot

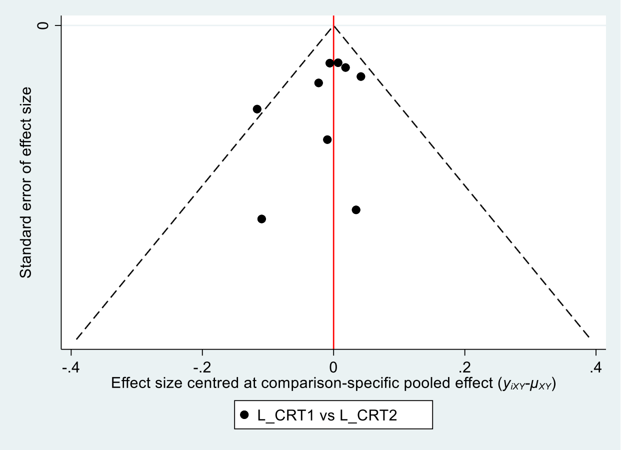

Egger's test

© 2024 Turri G et al. *JAMA Network Open*.

```
Number of studies =  9                              Root MSE      =   .9934
------------------------------------------------------------------------------
     Std_Eff |      Coef.   Std. Err.      t    P>|t|     [95% Conf. Interval]
-------------+----------------------------------------------------------------
       slope |  -.0121893   .0258203    -0.47   0.651    -.0732446     .048866
        bias |  -.8727976   .7336948    -1.19   0.273     -2.60771     .862115
------------------------------------------------------------------------------

Test of H0: no small-study effects          P = 0.273
```

© 2024 Turri G et al. *JAMA Network Open*.

eAppendix 4. Primary Outcome: Number-needed-to-treat

We calculated the control event rate (CER) by calculating the mean proportion of PCR in individuals receiving L-CRT1, as indicated by Veroniki et al. (J Clin Epidemiol 2019;111:11-22) → 22 RCT; mean 0.14; SD 0.056; range 0.05 to 0.27

Therefore, we calculate the number-needed-to-treat-to-benefit or to-harm (NNTB/NNTH) by applying the formula NNT = 1/((1-RR)*CER). Negative values were interpreted as NNTB and positive values as NNTH:

- L-CRT + consolidation vs. L-CRT1: RR 1.95 [1.25; 3.06] → NNTB 7.5 [28.5; 3.5]
- S-RT + consolidation vs. L-CRT1: RR 1.76 [1.34; 2.30] → NNTB 9.4 [21.0; 5.5]
- Induction + L-CRT vs. L-CRT1: RR 1.57 [1.09; 2.25] → NNTB 12.5 [79.4; 5.7]
- L-CRT2 vs. L-CRT1: RR 1.27 [1.09;1.48] → NNTB 26.4 [79.4; 14.8]
- CHT vs. L-CRT1: RR 0.75 [0.57; 0.98] → NNTH 28.6 [16.6; 357.1]
- S-RTdelayed vs. L-CRT1: RR 0.38 [0.10; 1.40] → NNTH 11.5 [NNTH 7.9; ∞; NNTB 17.8]
- L-RT vs. L-CRT1: RR 0.36 [0.24; 0.54] → NNTH 11.2 [9.4; 15.5]
- S-RTearly vs. L-CRT1: RR 0.07 [0.02; 0.22] → NNTH 7.7 [7.3; 9.1]

© 2024 Turri G et al. *JAMA Network Open*.

eAppendix 5. Primary Outcome: CINeMA

The analysis of the certainty of the evidence was performed with the online application CINeMA, which follows the principles of the GRADE methodology. The following criteria were applied:

- Within-study bias: the “overall” risk of bias of each study was calculated as follows: (a) LOW risk if all domains of the Cochrane RoB2 were at low risk; (b) HIGH risk if at least one domain was at high risk; (c) UNCLEAR RISK if for at least one domain there were “some concerns”. For each comparison, the histogram was interpreted according to a “Average risk of bias” rule;
- Reporting bias: a “low risk” was considered for all the included studies;
- Indirectness: the histogram was interpreted according to a “Average risk of bias” rule;
- Imprecision, Heterogeneity, Incoherence: Relative effect estimates below 0.67 and above 1.50 are considered clinically important.

Risk of bias contributions

The bar chart shows the contributions of each piece of study to the network estimate

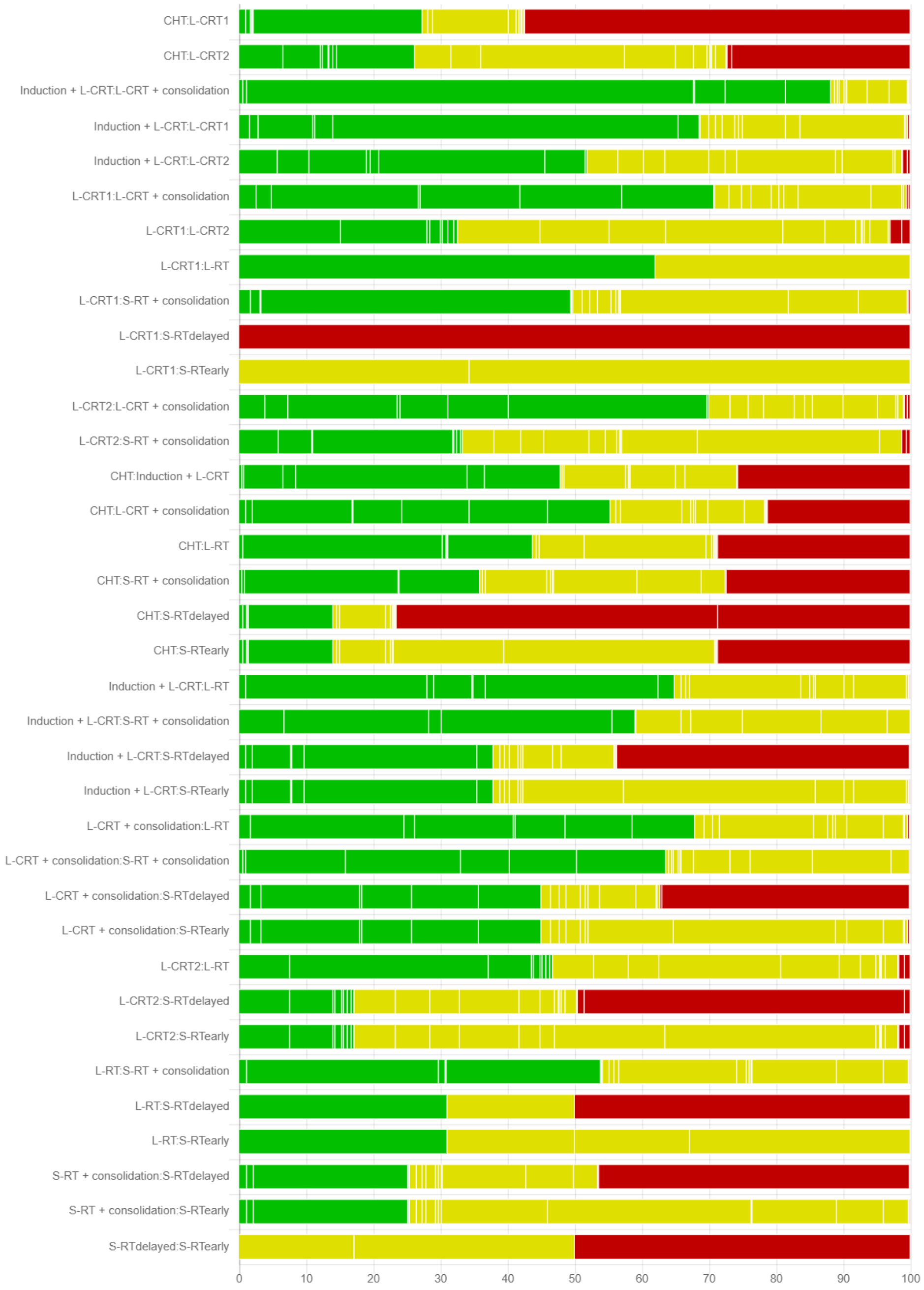

© 2024 Turri G et al. *JAMA Network Open*.

Indirectness contributions
The bar chart shows the contributions of each study to the network estimate

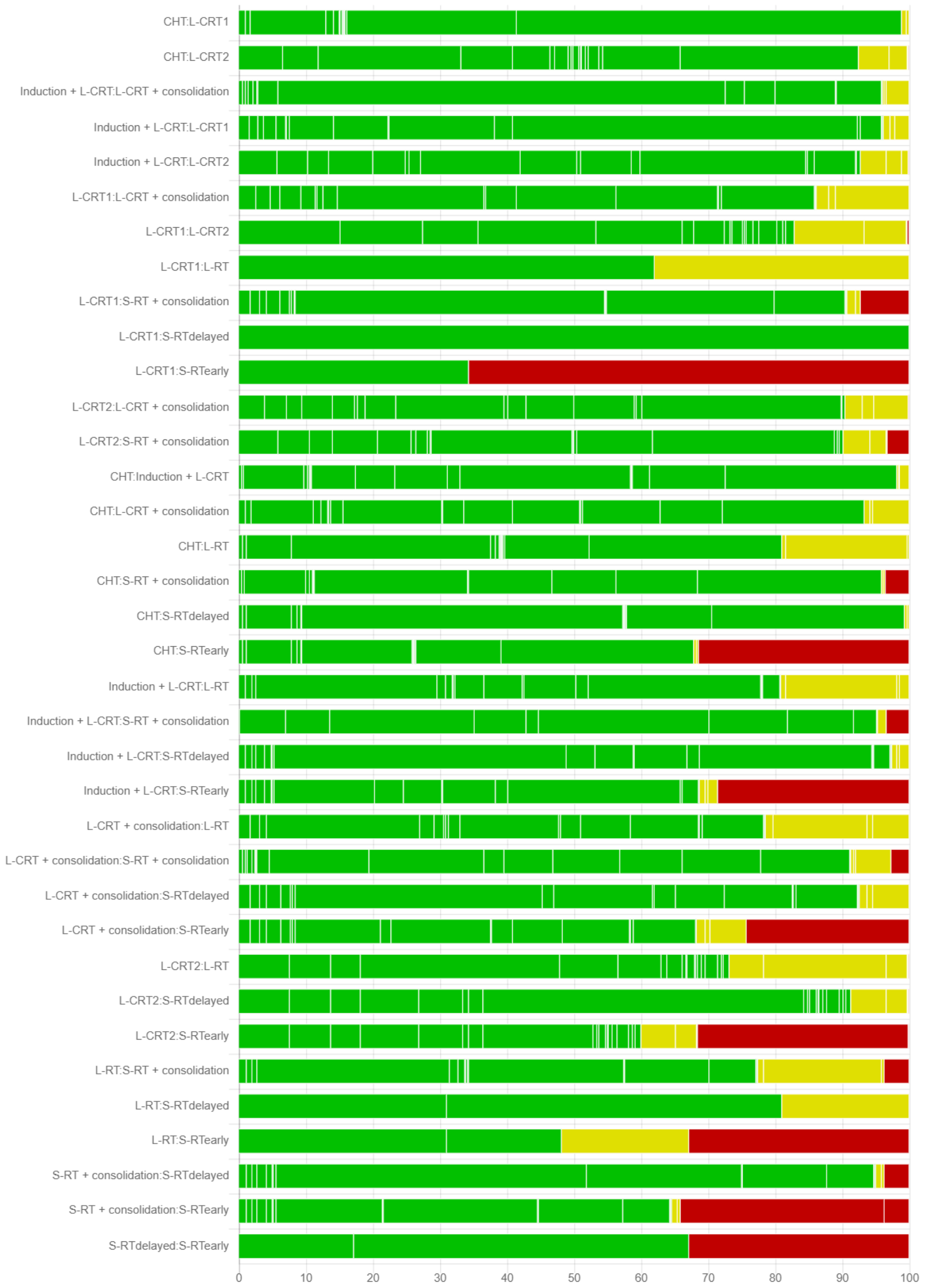

© 2024 Turri G et al. *JAMA Network Open*.

CINeMA report

Comparison	Number of studies	Within-study bias	Reporting bias	Indirectness	Imprecision	Heterogeneity	Incoherence	Confidence rating	Reason(s) for downgrading
CHT vs. L-CRT1	3	Some concerns	Low risk	No concerns	No concerns	Some concerns	Some concerns	Very low	["Within-study bias","Heterogeneity","Incoherence"]
CHT vs. L-CRT2	1	Some concerns	Low risk	No concerns	No concerns	No concerns	Some concerns	Low	["Within-study bias","Incoherence"]
Induction + L-CRT vs. L-CRT + consolidation	1	No concerns	Low risk	No concerns	Some concerns	No concerns	No concerns	Moderate	["Imprecision"]
Induction + L-CRT vs. L-CRT1	2	No concerns	Low risk	No concerns	No concerns	Some concerns	No concerns	Moderate	["Heterogeneity"]
Induction + L-CRT vs. L-CRT2	1	No concerns	Low risk	No concerns	Some concerns	No concerns	No concerns	Moderate	["Imprecision"]
L-CRT1 vs. L-CRT + consolidation	2	No concerns	Low risk	No concerns	No concerns	No concerns	No concerns	High	[]
L-CRT2 vs. L-CRT + consolidation	1	No concerns	Low risk	No concerns	Some concerns	No concerns	No concerns	Moderate	["Imprecision"]
L-CRT1 vs. L-CRT2	9	Some concerns	Low risk	No concerns	No concerns	Some concerns	No concerns	Low	["Within-study bias","Heterogeneity"]
L-CRT1 vs. L-RT	2	No concerns	Low risk	No concerns	No concerns	No concerns	Major concerns	Low	["Incoherence"]
L-CRT1 vs. S-RT + consolidation	3	Some concerns	Low risk	No concerns	No concerns	No concerns	No concerns	Moderate	["Within-study bias"]
L-CRT1 vs. S-RTdelayed	1	Major concerns	Low risk	No concerns	Some concerns	Some concerns	Major concerns	Very low	["Within-study bias","Imprecision","Heterogeneity","Incoherence"]
L-CRT1 vs. S-RTearly	2	Some concerns	Low risk	Some concerns	No concerns	No concerns	Major concerns	Very low	["Within-study bias","Indirectness","Incoherence"]
L-CRT2 vs. S-RT + consolidation	1	Some concerns	Low risk	No concerns	No concerns	Some concerns	No concerns	Low	["Within-study bias","Heterogeneity"]
CHT vs. Induction + L-CRT	0	Some concerns	Low risk	No concerns	No concerns	No concerns	Major concerns	Very low	["Within-study bias","Incoherence"]
CHT vs. L-CRT + consolidation	0	Some concerns	Low risk	No concerns	No concerns	No concerns	Major concerns	Very low	["Within-study bias","Incoherence"]
CHT vs. L-RT	0	Some concerns	Low risk	No concerns	No concerns	No concerns	Major concerns	Very low	["Within-study bias","Incoherence"]
CHT vs. S-RT + consolidation	0	Some concerns	Low risk	No concerns	No concerns	No concerns	Major concerns	Very low	["Within-study bias","Incoherence"]
CHT vs. S-RTdelayed	0	Major concerns	Low risk	No concerns	Major concerns	No concerns	Major concerns	Very low	["Within-study bias","Imprecision","Incoherence"]
CHT vs. S-RTearly	0	Some concerns	Low risk	Some concerns	No concerns	No concerns	Major concerns	Very low	["Within-study bias","Indirectness","Incoherence"]
Induction + L-CRT vs. L-RT	0	No concerns	Low risk	No concerns	No concerns	No concerns	Major concerns	Very low	["Incoherence"]
Induction + L-CRT vs. S-RT + consolidation	0	No concerns	Low risk	No concerns	Some concerns	Some concerns	Major concerns	Very low	["Imprecision","Heterogeneity","Incoherence"]
Induction + L-CRT vs. S-RTdelayed	0	Some concerns	Low risk	No concerns	No concerns	Some concerns	Major concerns	Very low	["Within-study bias","Heterogeneity","Incoherence"]
Induction + L-CRT vs. S-RTearly	0	Some concerns	Low risk	Some concerns	No concerns	No concerns	Major concerns	Very low	["Within-study bias","Indirectness","Incoherence"]
L-CRT + consolidation vs. L-RT	0	No concerns	Low risk	No concerns	No concerns	No concerns	Major concerns	Low	["Incoherence"]
L-CRT + consolidation vs.	0	No concerns	Low risk	No concerns	Major concerns	No concerns	Major concerns	Very low	["Imprecision","Incoherence"]

© 2024 Turri G et al. *JAMA Network Open*.

S-RT + consolidation									
L-CRT + consolidation vs. S-RTdelayed	0	Some concerns	Low risk	No concerns	No concerns	No concerns	Major concerns	Very low	["Within-study bias","Incoherence"]
L-CRT + consolidation vs. S-RTearly	0	Some concerns	Low risk	Some concerns	No concerns	No concerns	Major concerns	Very low	["Within-study bias","Indirectness"," Incoherence"]
L-CRT2 vs. L-RT	0	Some concerns	Low risk	No concerns	No concerns	No concerns	Major concerns	Very low	["Within-study bias","Incoherence"]
L-CRT2 vs. S-RTdelayed	0	Some concerns	Low risk	No concerns	Some concerns	No concerns	Major concerns	Very low	["Within-study bias","Imprecision"," Incoherence"]
L-CRT2 vs. S-RTearly	0	Some concerns	Low risk	Some concerns	No concerns	No concerns	Major concerns	Very low	["Within-study bias","Indirectness"," Incoherence"]
L-RT vs. S-RT + consolidation	0	No concerns	Low risk	No concerns	No concerns	No concerns	Major concerns	Low	["Incoherence"]
L-RT vs. S-RTdelayed	0	Some concerns	Low risk	No concerns	Major concerns	No concerns	Major concerns	Very low	["Within-study bias","Imprecision"," Incoherence"]
L-RT vs. S-RTearly	0	Some concerns	Low risk	Some concerns	No concerns	No concerns	Major concerns	Very low	["Within-study bias","Indirectness"," Incoherence"]
S-RT + consolidation vs. S-RTdelayed	0	Some concerns	Low risk	No concerns	No concerns	No concerns	Major concerns	Very low	["Within-study bias","Incoherence"]
S-RT + consolidation vs. S-RTearly	0	Some concerns	Low risk	Some concerns	No concerns	No concerns	Major concerns	Very low	["Within-study bias","Indirectness"," Incoherence"]
S-RTdelayed vs. S-RTearly	0	Major concerns	Low risk	Some concerns	Some concerns	No concerns	Major concerns	Very low	["Within-study bias","Indirectness"," Imprecision","Incoherence"]

© 2024 Turri G et al. *JAMA Network Open*.

eAppendix 6. Primary Outcome: **Sensitivity analyses for the primary outcome "PCR"**

Sensitivity 1 - excluding studies with high risk of bias

Characteristics of the network

Number of treatments:

8

Number of studies:

24

Number of individuals included:

12072

Number of individuals randomized to each treatment:

	Treatment name	N. individuals randomized
1	CHT	465
2	Induction + L-CRT	469
3	L-CRT + consolidation	288
4	L-CRT1	5435
5	L-CRT2	3137
6	L-RT	872
7	S-RT + consolidation	1090
8	S-RTearly	316

Pairwise meta-analysis
Risk ratios above 1 favor the first treatment of the comparison.

© 2024 Turri G et al. *JAMA Network Open*.

Study	logRR	SE(logRR)	RR	95%-CI	Weight (common)	Weight (random)
comparison = L-CRT2 VS L-CRT1						
Rodel 2015	0.2661	0.1368	1.30	[1.00; 1.71]	11.1%	4.8%
Aschele 2011	-0.0090	0.1663	0.99	[0.72; 1.37]	7.5%	4.7%
Gerard (2) 2010	0.3253	0.1906	1.38	[0.95; 2.01]	5.7%	4.6%
Deng 2016	0.7178	0.2495	2.05	[1.26; 3.34]	3.3%	4.3%
O'Connell 2014	0.0947	0.1171	1.10	[0.87; 1.38]	15.1%	4.9%
Schmoll 2021	0.2007	0.1588	1.22	[0.90; 1.67]	8.2%	4.8%
Jiao 2015	0.1823	0.2688	1.20	[0.71; 2.03]	2.9%	4.2%
Mohiuddin 2006	0.0158	0.3309	1.02	[0.53; 1.94]	1.9%	3.9%
Common effect model			**1.21**	**[1.08; 1.37]**	**55.7%**	--
Random effects model			**1.22**	**[1.07; 1.38]**	--	**36.3%**
Heterogeneity: $I^2 = 9\%$, $\tau^2 = 0.0030$, $p = 0.36$						
comparison = L-CRT2 VS CHT						
Deng 2016	1.4110	0.3351	4.10	[2.13; 7.91]	1.8%	3.9%
comparison = CHT VS L-CRT1						
Deng 2016	-0.6931	0.3713	0.50	[0.24; 1.04]	1.5%	3.7%
comparison = L-RT VS L-CRT1						
Bosset 2005	-0.9535	0.2268	0.39	[0.25; 0.60]	4.0%	4.4%
Gerard 2006	-1.1271	0.3097	0.32	[0.18; 0.59]	2.2%	4.0%
Common effect model			**0.36**	**[0.25; 0.52]**	**6.2%**	--
Random effects model			**0.36**	**[0.25; 0.52]**	--	**8.4%**
Heterogeneity: $I^2 = 0\%$, $\tau^2 = 0.0030$, $p = 0.65$						
comparison = L-CRT1 VS S-RTearly						
Bujko 2004	3.0782	1.0162	21.72	[2.96; 159.16]	0.2%	1.3%
Ngan 2012	2.4787	0.7275	11.93	[2.87; 49.63]	0.4%	2.0%
Common effect model			**14.61**	**[4.58; 46.58]**	**0.6%**	--
Random effects model			**14.62**	**[4.57; 46.73]**	--	**3.3%**
Heterogeneity: $I^2 = 0\%$, $\tau^2 = 0.0030$, $p = 0.63$						
comparison = Induction + L-CRT VS L-CRT2						
Fernandez-Martos 2015	0.0364	0.4785	1.04	[0.41; 2.65]	0.9%	3.1%
comparison = Induction + L-CRT VS L-CRT + consolidation						
Fokas 2019	-0.3810	0.2242	0.68	[0.44; 1.06]	4.1%	4.5%
comparison = S-RT + consolidation VS L-CRT1						
Bahadoer 2021	0.7181	0.1466	2.05	[1.54; 2.73]	9.6%	4.8%
Jin 2022	0.3508	0.2365	1.42	[0.89; 2.26]	3.7%	4.4%
Chakrabarti 2021	0.1621	0.4892	1.18	[0.45; 3.07]	0.9%	3.0%
Common effect model			**1.80**	**[1.42; 2.28]**	**14.2%**	--
Random effects model			**1.79**	**[1.39; 2.29]**	--	**12.2%**
Heterogeneity: $I^2 = 22\%$, $\tau^2 = 0.0030$, $p = 0.28$						
comparison = L-CRT1 VS L-CRT + consolidation						
Kim 2018	-0.7302	0.6804	0.48	[0.13; 1.83]	0.4%	2.2%
Moore 2017	0.4463	0.5788	1.56	[0.50; 4.86]	0.6%	2.6%
Common effect model			**0.95**	**[0.40; 2.26]**	**1.1%**	--
Random effects model			**0.95**	**[0.40; 2.27]**	--	**4.8%**
Heterogeneity: $I^2 = 42\%$, $\tau^2 = 0.0030$, $p = 0.19$						
comparison = L-CRT1 VS Induction + L-CRT						
Marechal 2012	0.0984	0.4446	1.10	[0.46; 2.64]	1.0%	3.3%
Conroy 2021	-0.8151	0.2162	0.44	[0.29; 0.68]	4.4%	4.5%
Common effect model			**0.53**	**[0.36; 0.77]**	**5.5%**	--
Random effects model			**0.53**	**[0.36; 0.78]**	--	**7.7%**
Heterogeneity: $I^2 = 71\%$, $\tau^2 = 0.0030$, $p = 0.06$						
comparison = S-RT + consolidation VS L-CRT2						
Bujko (2) 2013	0.3783	0.2483	1.46	[0.90; 2.38]	3.4%	4.3%
comparison = L-CRT2 VS L-CRT + consolidation						
Wang 2019	-0.5596	0.4039	0.57	[0.26; 1.26]	1.3%	3.5%
comparison = L-CRT1 VS CHT						
Mei 2023	0.2197	0.2331	1.25	[0.79; 1.97]	3.8%	4.4%
**Common effect model**			**1.13**	**[1.03; 1.24]**	**100.0%**	--
**Random effects model**			**1.13**	**[0.87; 1.46]**	--	**100.0%**

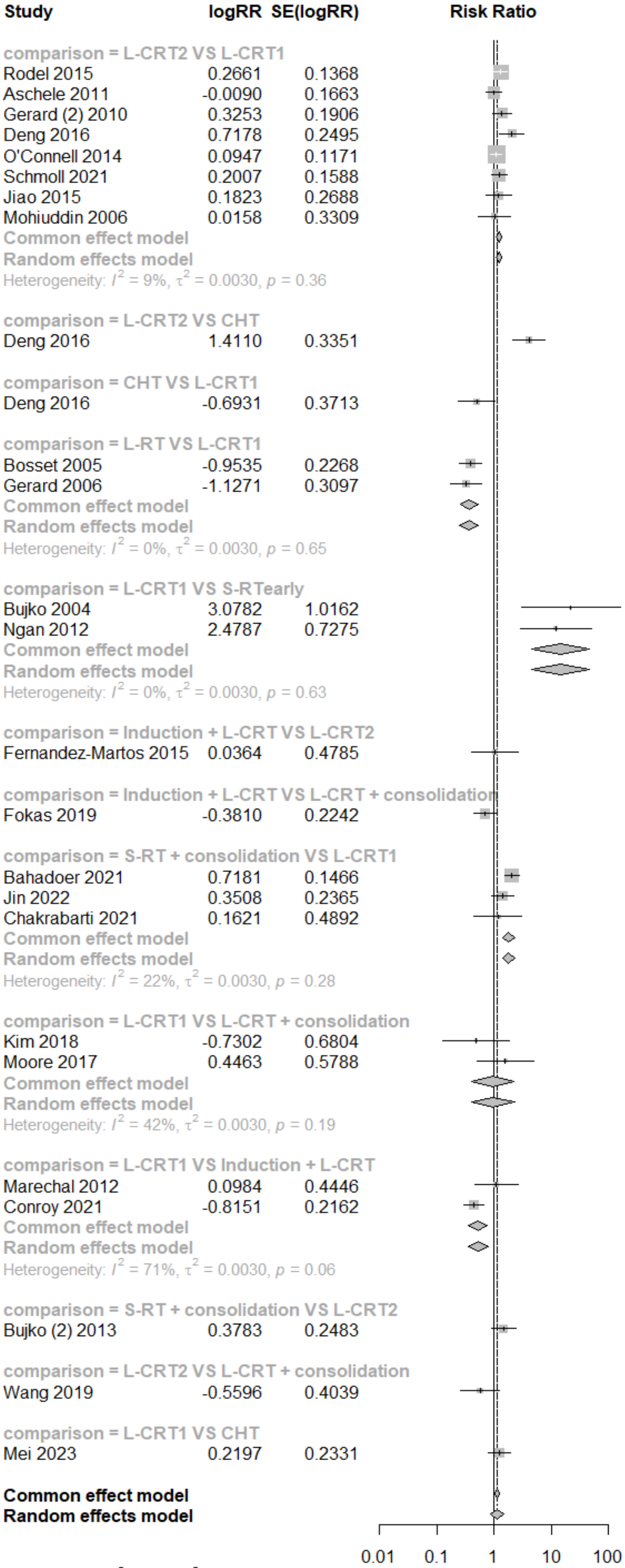

Heterogeneity: $I^2 = 81\%$, $\tau^2 = 0.3488$, $p < 0.01$
Residual heterogeneity: $I^2 = 18\%$, $\tau^2 = 0.0030$, $p = 0.26$
Test for subgroup differences (common effect): $\chi^2_{12} = 117.86$, df = 12 ($p < 0.01$)
Test for subgroup differences (random effects): $\chi^2_{12} = 112.17$, df = 12 ($p < 0.01$)

© 2024 Turri G et al. *JAMA Network Open*.

Network map The thickness of lines is proportional to the number of studies comparing the two treatments and the size of circles is proportional to the number of individuals for each treatment.

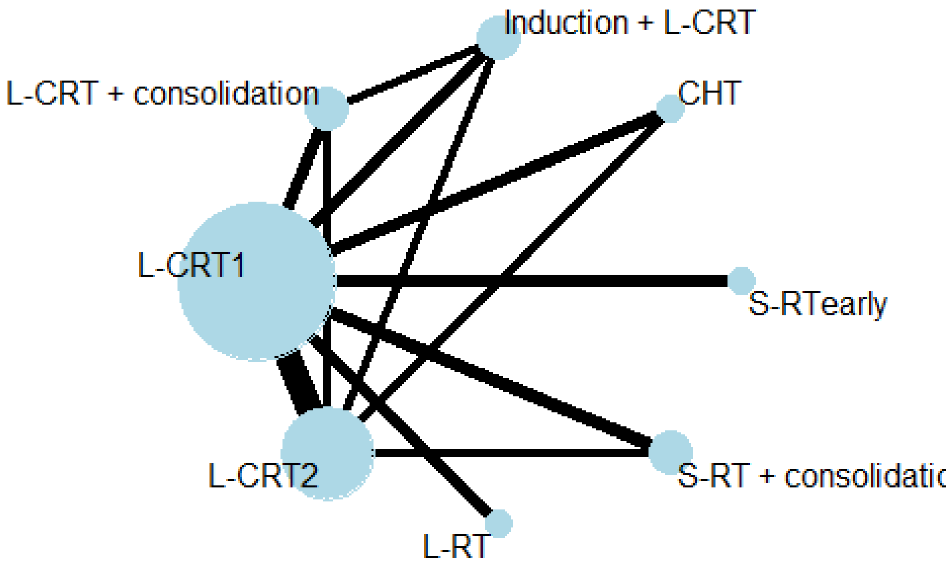

Netleague table Relative risks (RRs) and 95% confidence intervals (CIs) are reported. Results of the network meta-analysis are reported in the lower left part of the table and results from the pairwise meta-analysis are reported in the upper right part of the table. RRs higher than 1 favor the column-defining treatment.

	V1	V2	V3	V4	V5	V6	V7	V8
1	CHT	.	.	0.69 (0.45 to 1.07)	0.24 (0.12 to 0.49)	.	.	.
2	0.38 (0.22 to 0.66)	Induction + L-CRT	0.68 (0.41 to 1.14)	1.83 (1.19 to 2.83)	1.04 (0.39 to 2.74)	.	.	.
3	0.31 (0.17 to 0.56)	0.80 (0.53 to 1.22)	L-CRT + consolidation	1.05 (0.44 to 2.55)	1.75 (0.76 to 4.02)	.	.	.
4	0.60 (0.39 to 0.90)	1.57 (1.10 to 2.24)	1.95 (1.25 to 3.04)	L-CRT1	0.81 (0.70 to 0.95)	2.77 (1.85 to 4.16)	0.58 (0.43 to 0.78)	14.64 (4.52 to 47.42)
5	0.48 (0.31 to 0.73)	1.25 (0.86 to 1.82)	1.56 (0.99 to 2.45)	0.80 (0.69 to 0.93)	L-CRT2	.	0.69 (0.39 to 1.19)	.
6	1.65 (0.92 to 2.95)	4.34 (2.53 to 7.45)	5.40 (2.96 to 9.86)	2.77 (1.85 to 4.16)	3.46 (2.25 to 5.33)	L-RT	.	.
7	0.34 (0.21 to 0.55)	0.89 (0.57 to 1.39)	1.11 (0.66 to 1.86)	0.57 (0.44 to 0.74)	0.71 (0.53 to 0.95)	0.21 (0.13 to 0.33)	S-RT + consolidation	.
8	8.72 (2.51 to 30.34)	22.94 (6.72 to 78.33)	28.55 (8.13 to 100.30)	14.64 (4.52 to 47.42)	18.28 (5.59 to 59.76)	5.29 (1.52 to 18.32)	25.72 (7.71 to 85.76)	S-RTearly

Forest plot L-CRT-1 was used as a common comparator. RRs above 1 favor the strategy over the common comparator (L-CRT1).

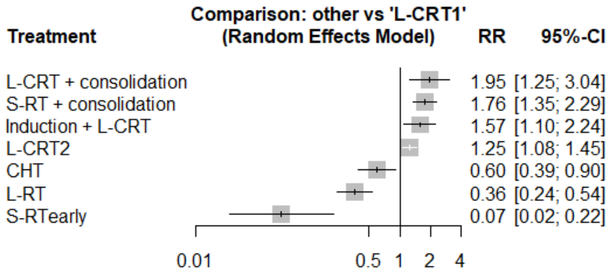

Assessment of heterogeneity and consistency

Global heterogeneity

© 2024 Turri G et al. *JAMA Network Open*.

We interpreted tau^2 as follows: heterogeneity low with tau^2≤0.010, moderate with 0.010<tau^2≤0.242, high with tau^2>0.242, and I^2 statistics as follows: not important (0%-40%), moderate (30%-60%), substantial (50%-90%), considerable (75%-100%).

```
tau^2= 0.0173 ; tau= 0.1316

I^2= 24.42 % ( 0 % to 56.69 %)
```

Consistency: global approach

Q statistic to assess consistency under the assumption of a full design-by-treatment interaction random effects model

Q statistics to assess homogeneity / consistency

	Q	df	p-value
Total	23.82	18	0.1611
Within designs	11.10	12	0.5207
Between designs	12.72	6	0.0477

Design-specific decomposition of within-designs Q statistic

Design	Q	df	p-value
L-CRT1:Induction + L-CRT	3.41	1	0.0646
L-CRT1:L-CRT + consolidation	1.73	1	0.1878
L-CRT1:L-CRT2	2.96	6	0.8139
L-CRT1:L-RT	0.20	1	0.6512
L-CRT1:S-RT + consolidation	2.55	2	0.2791
L-CRT1:S-RTearly	0.23	1	0.6314

Between-designs Q statistic after detaching of single designs

Detached design	Q	df	p-value
Induction + L-CRT:L-CRT + consolidation	11.10	5	0.0493
Induction + L-CRT:L-CRT2	12.44	5	0.0293
L-CRT + consolidation:L-CRT2	12.69	5	0.0264
L-CRT1:CHT	8.38	5	0.1366
L-CRT1:Induction + L-CRT	10.59	5	0.0601
L-CRT1:L-CRT + consolidation	9.80	5	0.0810
L-CRT1:L-CRT2	8.16	5	0.1476
L-CRT1:S-RT + consolidation	12.72	5	0.0261
L-CRT2:S-RT + consolidation	12.72	5	0.0261
L-CRT1:CHT:L-CRT2	3.76	4	0.4393

Q statistic to assess consistency under the assumption of a full design-by-treatment interaction random effects model

	Q	df	p-value	tau.within	tau2.within
Between designs	12.72	6	0.0477	0	0

Consistency: local approach

Separate indirect from direct evidence (SIDE) using back-calculation method

Random effects model:

comparison	k	prop	nma	direct	indir.	RoR	z	p-value
L-CRT1:CHT	2	0.92	1.68	1.45	10.20	0.14	-2.44	0.0147
CHT:L-CRT2	1	0.37	0.48	0.24	0.71	0.34	-2.35	0.0188
Induction + L-CRT:L-CRT + consolidation	1	0.67	0.80	0.68	1.12	0.61	-1.09	0.2773
L-CRT1:Induction + L-CRT	2	0.67	0.64	0.55	0.88	0.62	-1.23	0.2184
Induction + L-CRT:L-CRT2	1	0.15	1.25	1.04	1.30	0.80	-0.42	0.6778
L-CRT1:L-CRT + consolidation	2	0.25	0.51	0.95	0.42	2.28	1.58	0.1136

© 2024 Turri G et al. *JAMA Network Open*.

L-CRT + consolidation:L-CRT2	1	0.29	1.56	1.75	1.49	1.18	0.32	0.7492
L-CRT1:L-CRT2	8	0.89	0.80	0.81	0.70	1.16	0.59	0.5524
L-CRT1:S-RT + consolidation	3	0.79	0.57	0.58	0.55	1.05	0.15	0.8772
L-CRT2:S-RT + consolidation	1	0.27	0.71	0.69	0.72	0.95	-0.15	0.8772

Legend:
comparison - Treatment comparison
k - Number of studies providing direct evidence
prop - Direct evidence proportion
nma - Estimated treatment effect (RR) in network meta-analysis
direct - Estimated treatment effect (RR) derived from direct evidence
indir. - Estimated treatment effect (RR) derived from indirect evidence
RoR - Ratio of Ratios (direct versus indirect)
z - z-value of test for disagreement (direct versus indirect)
p-value - p-value of test for disagreement (direct versus indirect)

Netrank Higher p-score values indicate higher ranking of treatments

	P-score
L-CRT + consolidation	0.9249
S-RT + consolidation	0.8614
Induction + L-CRT	0.7620
L-CRT2	0.5930
L-CRT1	0.4290
CHT	0.2803
L-RT	0.1488
S-RTearly	0.0007

© 2024 Turri G et al. *JAMA Network Open*.

<u>Sensitivity 2 - excluding studies with high indirectness</u>

Number of treatments:

9

Number of studies:

23

Number of individuals included:

10811

Number of individuals randomized to each treatment:

	Treatment name	N. individuals randomized
1	CHT	465
2	Induction + L-CRT	469
3	L-CRT + consolidation	288
4	L-CRT1	4802
5	L-CRT2	3169
6	L-RT	367
7	S-RT + consolidation	1021
8	S-RTdelayed	75
9	S-RTearly	155

Pairwise meta-analysis
Risk ratios above 1 favor the first treatment of the comparison.

© 2024 Turri G et al. *JAMA Network Open*.

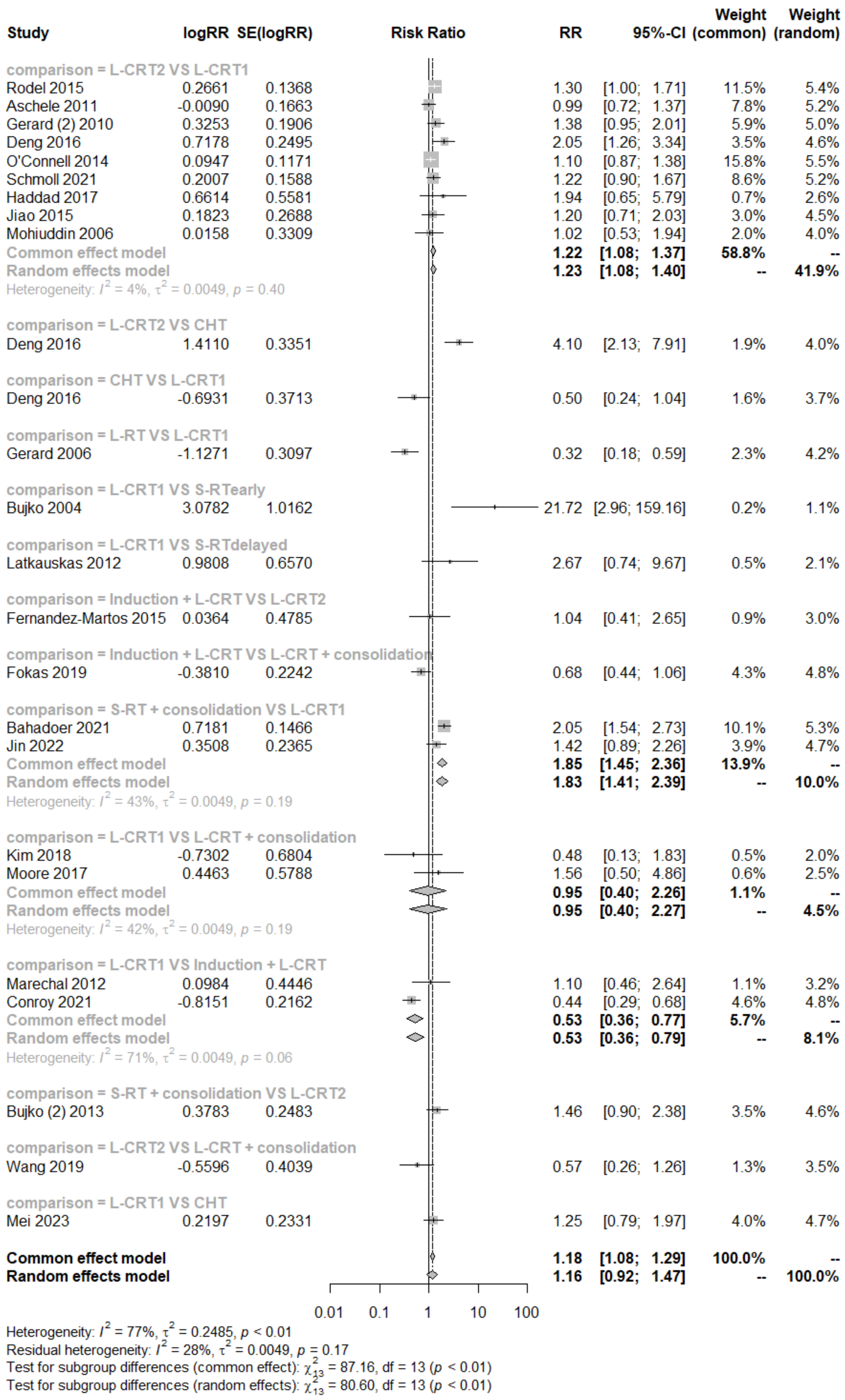

© 2024 Turri G et al. *JAMA Network Open*.

Network map The thickness of lines is proportional to the number of studies comparing the two treatments and the size of circles is proportional to the number of individuals for each treatment.

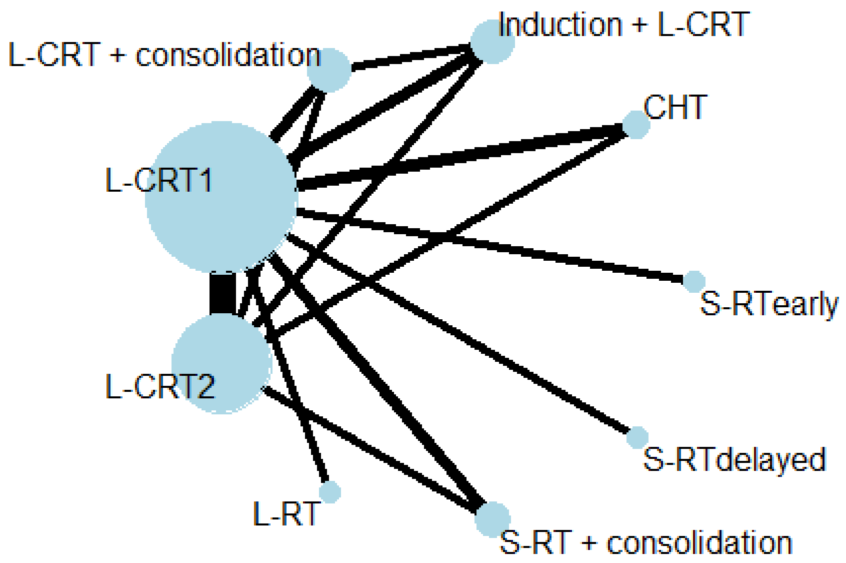

Netleague table Relative risks (RRs) and 95% confidence intervals (CIs) are reported. Results of the network meta-analysis are reported in the lower left part of the table and results from the pairwise meta-analysis are reported in the upper right part of the table. RRs higher than 1 favor the column-defining treatment.

	V1	V2	V3	V4	V5	V6	V7	V8	V9
1	CHT	.	.	0.69 (0.44 to 1.08)	0.24 (0.12 to 0.50)	.	.	.	.
2	0.38 (0.22 to 0.67)	Induction + L-CRT	0.68 (0.40 to 1.16)	1.82 (1.16 to 2.85)	1.04 (0.39 to 2.77)	.	.	.	.
3	0.31 (0.16 to 0.57)	0.80 (0.52 to 1.24)	L-CRT + consolidation	1.05 (0.43 to 2.57)	1.75 (0.75 to 4.07)	.	.	.	.
4	0.59 (0.39 to 0.91)	1.56 (1.08 to 2.25)	1.94 (1.23 to 3.05)	L-CRT1	0.80 (0.68 to 0.95)	3.09 (1.57 to 6.06)	0.56 (0.40 to 0.77)	2.67 (0.71 to 9.99)	21.72 (2.90 to 162.66
5	0.47 (0.30 to 0.73)	1.23 (0.84 to 1.81)	1.53 (0.96 to 2.43)	0.79 (0.68 to 0.92)	L-CRT2	.	0.69 (0.39 to 1.21)	.	.
6	1.83 (0.82 to 4.08)	4.80 (2.23 to 10.36)	5.97 (2.65 to 13.49)	3.09 (1.57 to 6.06)	3.90 (1.95 to 7.80)	L-RT	.	.	.
7	0.33 (0.20 to 0.55)	0.86 (0.54 to 1.37)	1.07 (0.63 to 1.83)	0.55 (0.41 to 0.74)	0.70 (0.51 to 0.95)	0.18 (0.09 to 0.37)	S-RT + consolidation	.	.
8	1.58 (0.39 to 6.35)	4.15 (1.05 to 16.35)	5.16 (1.28 to 20.88)	2.67 (0.71 to 9.99)	3.37 (0.89 to 12.75)	0.86 (0.20 to 3.81)	4.82 (1.25 to 18.64)	S-RTdelayed	.
9	12.90 (1.65 to 101.11)	33.80 (4.37 to 261.71)	42.04 (5.34 to 331.28)	21.72 (2.90 to 162.66)	27.45 (3.64 to 206.81)	7.04 (0.84 to 58.83)	39.28 (5.14 to 300.29)	8.14 (0.73 to 90.52)	S-RTearly

Forest plot L-CRT-1 was used as a common comparator. RRs above 1 favor the strategy over the common comparator (L-CRT1).

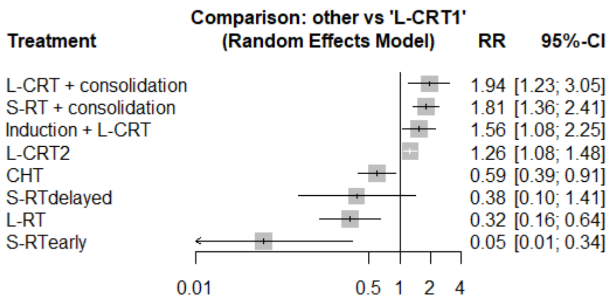

Assessment of heterogeneity and consistency

Global heterogeneity

© 2024 Turri G et al. *JAMA Network Open*.

We interpreted tau^2 as follows: heterogeneity low with tau^2≤0.010, moderate with 0.010<tau^2≤0.242, high with tau^2>0.242, and I^2 statistics as follows: not important (0%-40%), moderate (30%-60%), substantial (50%-90%), considerable (75%-100%).

```
tau^2= 0.0227 ; tau= 0.1505

I^2= 31.12 % ( 0 % to 61.64 %)
```

Consistency: global approach

Q statistic to assess consistency under the assumption of a full design-by-treatment interaction random effects model

```
Q statistics to assess homogeneity / consistency

                    Q df p-value
Total           23.23 16  0.1077
Within designs  10.65 10  0.3854
Between designs 12.58  6  0.0502

Design-specific decomposition of within-designs Q statistic

                           Design    Q df p-value
      L-CRT1:Induction + L-CRT 3.41  1  0.0646
 L-CRT1:L-CRT + consolidation 1.73  1  0.1878
                 L-CRT1:L-CRT2 3.76  7  0.8073
  L-CRT1:S-RT + consolidation 1.74  1  0.1867

Between-designs Q statistic after detaching of single designs

                       Detached design     Q df p-value
Induction + L-CRT:L-CRT + consolidation 10.98  5  0.0518
              Induction + L-CRT:L-CRT2 12.31  5  0.0308
          L-CRT + consolidation:L-CRT2 12.55  5  0.0280
                           L-CRT1:CHT  8.28  5  0.1414
              L-CRT1:Induction + L-CRT 10.48  5  0.0626
          L-CRT1:L-CRT + consolidation  9.64  5  0.0860
                         L-CRT1:L-CRT2  7.93  5  0.1604
           L-CRT1:S-RT + consolidation 12.57  5  0.0277
           L-CRT2:S-RT + consolidation 12.57  5  0.0277
                    L-CRT1:CHT:L-CRT2  3.78  4  0.4368

Q statistic to assess consistency under the assumption of a full design-by-treatment interaction random effects model

                    Q df p-value tau.within tau2.within
Between designs 12.02  6  0.0615     0.0515      0.0027
```

Consistency: local approach

```
Separate indirect from direct evidence (SIDE) using back-calculation method

Random effects model:

                             comparison k prop  nma direct indir.  RoR     z p-value
                             L-CRT1:CHT 2 0.92 1.68   1.45   9.60 0.15 -2.31  0.0206
                               CHT:L-CRT2 1 0.38 0.47   0.24   0.70 0.35 -2.27  0.0234
Induction + L-CRT:L-CRT + consolidation 1 0.66 0.80   0.68   1.10 0.62 -1.04  0.3004
               L-CRT1:Induction + L-CRT 2 0.67 0.64   0.55   0.87 0.63 -1.16  0.2468
               Induction + L-CRT:L-CRT2 1 0.15 1.23   1.04   1.27 0.82 -0.37  0.7098
           L-CRT1:L-CRT + consolidation 2 0.26 0.52   0.95   0.42 2.28  1.56  0.1190
           L-CRT + consolidation:L-CRT2 1 0.30 1.53   1.75   1.45 1.21  0.37  0.7117
                          L-CRT1:L-CRT2 9 0.89 0.79   0.80   0.69 1.16  0.58  0.5634
```

© 2024 Turri G et al. *JAMA Network Open*.

```
L-CRT1:S-RT + consolidation 2 0.76 0.55   0.56   0.54 1.03  0.08  0.9353
L-CRT2:S-RT + consolidation 1 0.29 0.70   0.69   0.70 0.97 -0.08  0.9353

Legend:
comparison - Treatment comparison
k          - Number of studies providing direct evidence
prop       - Direct evidence proportion
nma        - Estimated treatment effect (RR) in network meta-analysis
direct     - Estimated treatment effect (RR) derived from direct evidence
indir.     - Estimated treatment effect (RR) derived from indirect evidence
RoR        - Ratio of Ratios (direct versus indirect)
z          - z-value of test for disagreement (direct versus indirect)
p-value    - p-value of test for disagreement (direct versus indirect)
```

Netrank Higher p-score values indicate higher ranking of treatments

	P-score
L-CRT + consolidation	0.9237
S-RT + consolidation	0.8895
Induction + L-CRT	0.7810
L-CRT2	0.6439
L-CRT1	0.4912
CHT	0.3344
S-RTdelayed	0.2429
L-RT	0.1821
S-RTearly	0.0113

© 2024 Turri G et al. *JAMA Network Open*.

Sensitivity 3 - excluding CHT arm

Characteristics of the network

Number of treatments:

8

Number of studies:

24

Number of individuals included:

11201

Number of individuals randomized to each treatment:

	Treatment name	N. individuals randomized
1	Induction + L-CRT	469
2	L-CRT + consolidation	288
3	L-CRT1	5087
4	L-CRT2	3004
5	L-RT	872
6	S-RT + consolidation	1090
7	S-RTdelayed	75
8	S-RTearly	316

Pairwise meta-analysis
Risk ratios above 1 favor the first treatment of the comparison.

© 2024 Turri G et al. *JAMA Network Open*.

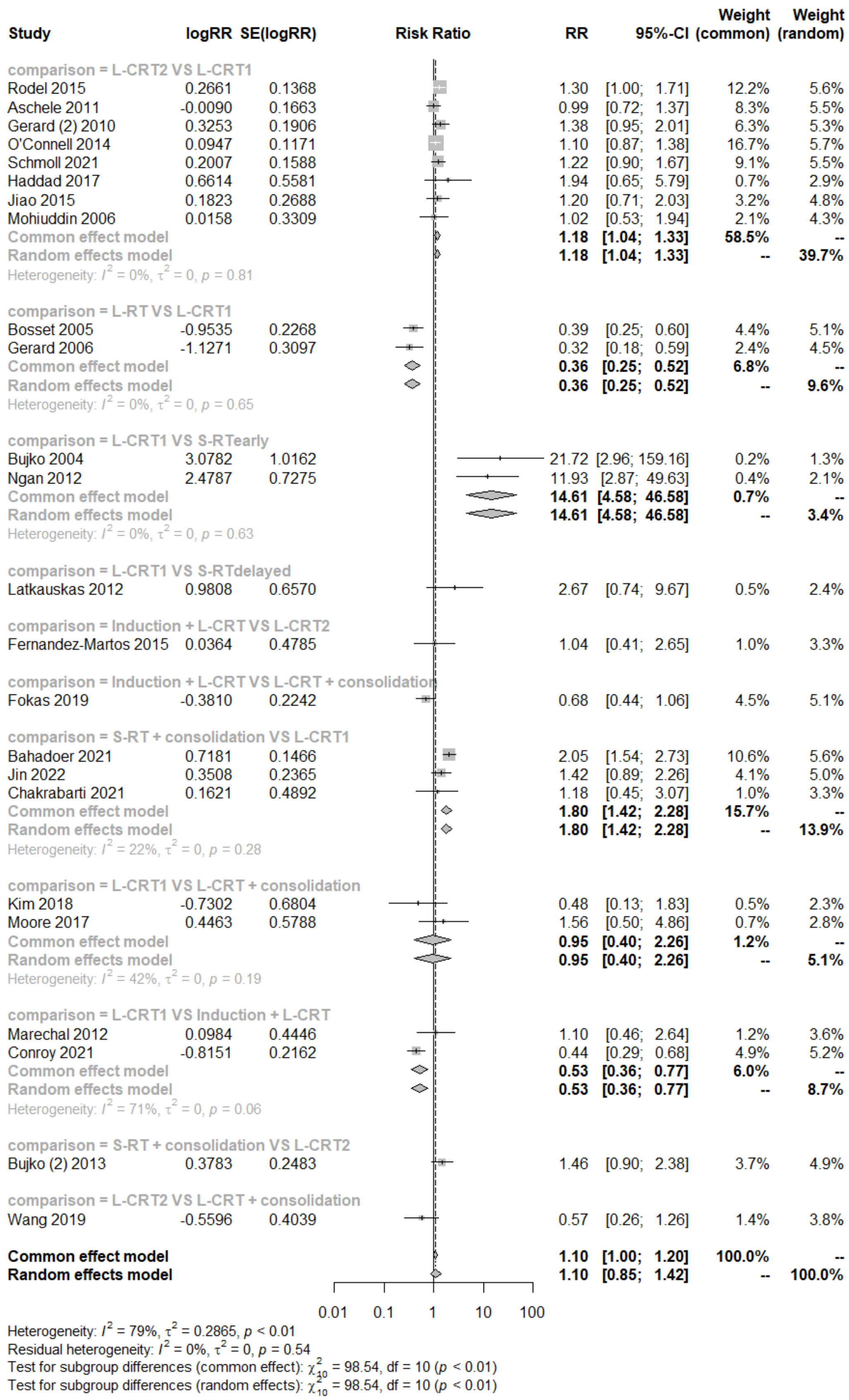

© 2024 Turri G et al. *JAMA Network Open*.

Network map The thickness of lines is proportional to the number of studies comparing the two treatments and the size of circles is proportional to the number of individuals for each treatment.

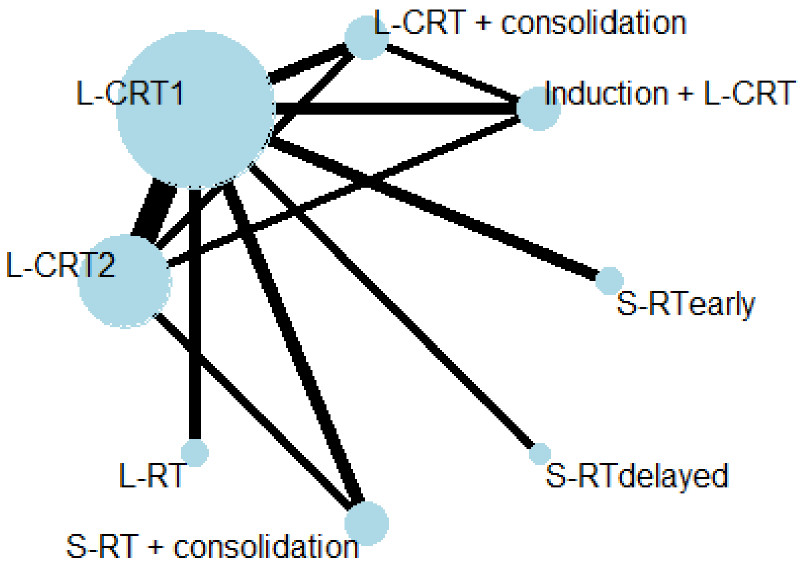

Netleague table Relative risks (RRs) and 95% confidence intervals (CIs) are reported. Results of the network meta-analysis are reported in the lower left part of the table and results from the pairwise meta-analysis are reported in the upper right part of the table. RRs higher than 1 favor the column-defining treatment.

	V1	V2	V3	V4	V5	V6	V7	V8
1	Induction + L-CRT	0.68 (0.44 to 1.06)	1.90 (1.30 to 2.78)	1.04 (0.41 to 2.65)	.	.	.	.
2	0.80 (0.55 to 1.16)	L-CRT + consolidation	1.05 (0.44 to 2.49)	1.75 (0.79 to 3.86)	.	.	.	.
3	1.61 (1.17 to 2.22)	2.01 (1.34 to 3.02)	L-CRT1	0.85 (0.75 to 0.96)	2.76 (1.93 to 3.95)	0.56 (0.44 to 0.70)	2.67 (0.74 to 9.67)	14.61 (4.58 to 46.58)
4	1.36 (0.97 to 1.89)	1.69 (1.12 to 2.56)	0.84 (0.75 to 0.95)	L-CRT2	.	0.69 (0.42 to 1.11)	.	.
5	4.44 (2.75 to 7.18)	5.55 (3.22 to 9.55)	2.76 (1.93 to 3.95)	3.27 (2.24 to 4.78)	L-RT	.	.	.
6	0.90 (0.61 to 1.32)	1.13 (0.71 to 1.78)	0.56 (0.45 to 0.69)	0.66 (0.53 to 0.84)	0.20 (0.13 to 0.31)	S-RT + consolidation	.	.
7	4.30 (1.14 to 16.19)	5.37 (1.39 to 20.71)	2.67 (0.74 to 9.67)	3.17 (0.87 to 11.54)	0.97 (0.25 to 3.68)	4.77 (1.29 to 17.59)	S-RTdelayed	.
8	23.54 (7.07 to 78.35)	29.40 (8.60 to 100.48)	14.61 (4.58 to 46.58)	17.35 (5.41 to 55.65)	5.30 (1.57 to 17.84)	26.13 (8.04 to 84.95)	5.48 (0.97 to 30.99)	S-RTearly

Forest plot L-CRT-1 was used as a common comparator. RRs above 1 favor the strategy over the common comparator (L-CRT1).

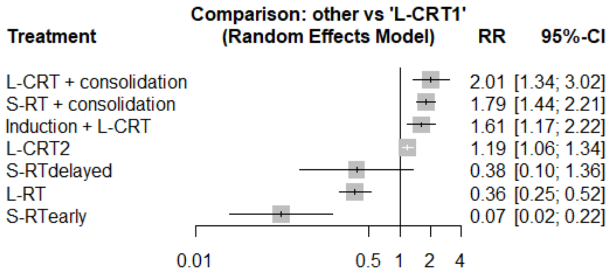

Assessment of heterogeneity and consistency

Global heterogeneity

© 2024 Turri G et al. *JAMA Network Open*.

We interpreted tau^2 as follows: heterogeneity low with tau^2≤0.010, moderate with 0.010<tau^2≤0.242, high with tau^2>0.242, and I^2 statistics as follows: not important (0%-40%), moderate (30%-60%), substantial (50%-90%), considerable (75%-100%).

tau^2= 0 ; tau= 0

I^2= 0 % (0 % to 49.97 %)

Consistency: global approach

Q statistic to assess consistency under the assumption of a full design-by-treatment interaction random effects model

Q statistics to assess homogeneity / consistency

	Q	df	p-value
Total	15.64	17	0.5498
Within designs	11.89	13	0.5364
Between designs	3.74	4	0.4421

Design-specific decomposition of within-designs Q statistic

Design	Q	df	p-value
L-CRT1:Induction + L-CRT	3.41	1	0.0646
L-CRT1:L-CRT + consolidation	1.73	1	0.1878
L-CRT1:L-CRT2	3.76	7	0.8073
L-CRT1:L-RT	0.20	1	0.6512
L-CRT1:S-RT + consolidation	2.55	2	0.2791
L-CRT1:S-RTearly	0.23	1	0.6314

Between-designs Q statistic after detaching of single designs

Detached design	Q	df	p-value
Induction + L-CRT:L-CRT + consolidation	2.03	3	0.5661
Induction + L-CRT:L-CRT2	3.38	3	0.3364
L-CRT + consolidation:L-CRT2	3.73	3	0.2918
L-CRT1:Induction + L-CRT	1.38	3	0.7104
L-CRT1:L-CRT + consolidation	0.93	3	0.8174
L-CRT1:L-CRT2	3.63	3	0.3046
L-CRT1:S-RT + consolidation	3.72	3	0.2931
L-CRT2:S-RT + consolidation	3.72	3	0.2931

Q statistic to assess consistency under the assumption of a full design-by-treatment interaction random effects model

	Q	df	p-value	tau.within	tau2.within
Between designs	3.74	4	0.4421	0	0

Consistency: local approach

Separate indirect from direct evidence (SIDE) using back-calculation method

Random effects model:

comparison	k	prop	nma	direct	indir.	RoR	z	p-value
Induction + L-CRT:L-CRT + consolidation	1	0.71	0.80	0.68	1.18	0.58	-1.31	0.1908
L-CRT1:Induction + L-CRT	2	0.70	0.62	0.53	0.91	0.58	-1.54	0.1243
Induction + L-CRT:L-CRT2	1	0.12	1.36	1.04	1.41	0.74	-0.60	0.5485
L-CRT1:L-CRT + consolidation	2	0.22	0.50	0.95	0.41	2.31	1.68	0.0938
L-CRT + consolidation:L-CRT2	1	0.27	1.69	1.75	1.67	1.05	0.09	0.9254
L-CRT1:L-CRT2	8	0.93	0.84	0.85	0.78	1.08	0.34	0.7350
L-CRT1:S-RT + consolidation	3	0.82	0.56	0.56	0.58	0.96	-0.14	0.8869
L-CRT2:S-RT + consolidation	1	0.23	0.66	0.69	0.66	1.04	0.14	0.8869

© 2024 Turri G et al. *JAMA Network Open*.

Legend:
comparison - Treatment comparison
k - Number of studies providing direct evidence
prop - Direct evidence proportion
nma - Estimated treatment effect (RR) in network meta-analysis
direct - Estimated treatment effect (RR) derived from direct evidence
indir. - Estimated treatment effect (RR) derived from indirect evidence
RoR - Ratio of Ratios (direct versus indirect)
z - z-value of test for disagreement (direct versus indirect)
p-value - p-value of test for disagreement (direct versus indirect)

Netrank Higher p-score values indicate higher ranking of treatments

	P-score
L-CRT + consolidation	0.9372
S-RT + consolidation	0.8572
Induction + L-CRT	0.7661
L-CRT2	0.5714
L-CRT1	0.4195
S-RTdelayed	0.2333
L-RT	0.2110
S-RTearly	0.0044

© 2024 Turri G et al. *JAMA Network Open*.

Sensitivity 4 - PCR on the Per Protocol Populations

Characteristics of the network

Number of treatments:

9

Number of studies:

27

Number of individuals included:

12558

Number of individuals randomized to each treatment:

	Treatment name	N. individuals randomized
1	CHT	959
2	Induction + L-CRT	437
3	L-CRT + consolidation	269
4	L-CRT1	5707
5	L-CRT2	3009
6	L-RT	836
7	S-RT + consolidation	965
8	S-RTdelayed	68
9	S-RTearly	308

Pairwise meta-analysis
Risk ratios above 1 favor the first treatment of the comparison.

© 2024 Turri G et al. *JAMA Network Open*.

Study	logRR	SE(logRR)	Risk Ratio	RR	95%-CI	Weight (common)	Weight (random)
comparison = L-CRT2 VS L-CRT1							
Rodel 2015	0.2813	0.1366		1.32	[1.01; 1.73]	9.4%	4.4%
Aschele 2011	-0.0187	0.1657		0.98	[0.71; 1.36]	6.4%	4.3%
Gerard (2) 2010	0.3185	0.1903		1.38	[0.95; 2.00]	4.8%	4.2%
Deng 2016	0.6837	0.2464		1.98	[1.22; 3.21]	2.9%	4.0%
O'Connell 2014	0.0912	0.1166		1.10	[0.87; 1.38]	12.9%	4.5%
Schmoll 2021	0.2517	0.1579		1.29	[0.94; 1.75]	7.0%	4.3%
Haddad 2017	0.8473	0.5455		2.33	[0.80; 6.80]	0.6%	2.5%
Jiao 2015	0.1823	0.2688		1.20	[0.71; 2.03]	2.4%	3.8%
Mohiuddin 2006	-0.0093	0.3265		0.99	[0.52; 1.88]	1.6%	3.5%
Common effect model				**1.23**	**[1.09; 1.38]**	**47.9%**	--
Random effects model				**1.23**	**[1.09; 1.39]**	--	**35.6%**
Heterogeneity: $I^2 = 12\%$, $\tau^2 = 0.0018$, $p = 0.33$							
comparison = L-CRT2 VS CHT							
Deng 2016	1.4309	0.3333		4.18	[2.18; 8.04]	1.6%	3.5%
comparison = CHT VS L-CRT1							
Deng 2016	-0.7472	0.3694		0.47	[0.23; 0.98]	1.3%	3.3%
comparison = L-RT VS L-CRT1							
Bosset 2005	-0.9618	0.2262		0.38	[0.25; 0.60]	3.4%	4.1%
Gerard 2006	-1.1514	0.3094		0.32	[0.17; 0.58]	1.8%	3.6%
Common effect model				**0.36**	**[0.25; 0.51]**	**5.2%**	--
Random effects model				**0.36**	**[0.25; 0.51]**	--	**7.7%**
Heterogeneity: $I^2 = 0\%$, $\tau^2 = 0.0018$, $p = 0.62$							
comparison = L-CRT1 VS S-RTearly							
Bujko 2004	3.1672	1.0157		23.74	[3.24; 173.80]	0.2%	1.1%
Ngan 2012	2.4913	0.7273		12.08	[2.90; 50.24]	0.3%	1.8%
Common effect model				**15.19**	**[4.77; 48.39]**	**0.5%**	--
Random effects model				**15.19**	**[4.76; 48.48]**	--	**2.9%**
Heterogeneity: $I^2 = 0\%$, $\tau^2 = 0.0018$, $p = 0.59$							
comparison = L-CRT1 VS S-RTdelayed							
Latkauskas 2012	0.9237	0.6555		2.52	[0.70; 9.10]	0.4%	2.0%
comparison = Induction + L-CRT VS L-CRT2							
Fernandez-Martos 2015	-0.0268	0.4771		0.97	[0.38; 2.48]	0.8%	2.8%
comparison = Induction + L-CRT VS L-CRT + consolidation							
Fokas 2019	-0.3417	0.2222		0.71	[0.46; 1.10]	3.5%	4.1%
comparison = S-RT + consolidation VS L-CRT1							
Bahadoer 2021	0.6815	0.1450		1.98	[1.49; 2.63]	8.3%	4.4%
Jin 2022	0.3462	0.2325		1.41	[0.90; 2.23]	3.2%	4.0%
Chakrabarti 2021	0.1981	0.4854		1.22	[0.47; 3.16]	0.7%	2.7%
Common effect model				**1.76**	**[1.39; 2.22]**	**12.3%**	--
Random effects model				**1.75**	**[1.37; 2.23]**	--	**11.1%**
Heterogeneity: $I^2 = 5\%$, $\tau^2 = 0.0018$, $p = 0.35$							
comparison = L-CRT1 VS L-CRT + consolidation							
Kim 2018	-0.8602	0.6768		0.42	[0.11; 1.59]	0.4%	2.0%
Moore 2017	0.4463	0.5788		1.56	[0.50; 4.86]	0.5%	2.3%
Common effect model				**0.90**	**[0.38; 2.13]**	**0.9%**	--
Random effects model				**0.90**	**[0.38; 2.13]**	--	**4.3%**
Heterogeneity: $I^2 = 54\%$, $\tau^2 = 0.0018$, $p = 0.14$							
comparison = L-CRT1 VS Induction + L-CRT							
Marechal 2012	0.0972	0.4417		1.10	[0.46; 2.62]	0.9%	2.9%
Conroy 2021	-0.8426	0.2148		0.43	[0.28; 0.66]	3.8%	4.1%
Common effect model				**0.52**	**[0.35; 0.75]**	**4.7%**	--
Random effects model				**0.52**	**[0.35; 0.76]**	--	**7.0%**
Heterogeneity: $I^2 = 73\%$, $\tau^2 = 0.0018$, $p = 0.06$							
comparison = S-RT + consolidation VS L-CRT2							
Bujko (2) 2013	0.3679	0.2472		1.44	[0.89; 2.35]	2.9%	3.9%
comparison = L-CRT2 VS L-CRT + consolidation							
Wang 2019	-0.4869	0.4000		0.61	[0.28; 1.35]	1.1%	3.2%
comparison = L-CRT1 VS CHT							
Mei 2023	0.2236	0.2315		1.25	[0.79; 1.97]	3.3%	4.0%
Schrag 2023	0.1060	0.1131		1.11	[0.89; 1.39]	13.7%	4.5%
Common effect model				**1.14**	**[0.93; 1.39]**	**16.9%**	--
Random effects model				**1.14**	**[0.92; 1.41]**	--	**8.5%**
Heterogeneity: $I^2 = 0\%$, $\tau^2 = 0.0018$, $p = 0.65$							
**Common effect model**				**1.14**	**[1.05; 1.23]**	**100.0%**	--
**Random effects model**				**1.16**	**[0.91; 1.48]**	--	**100.0%**

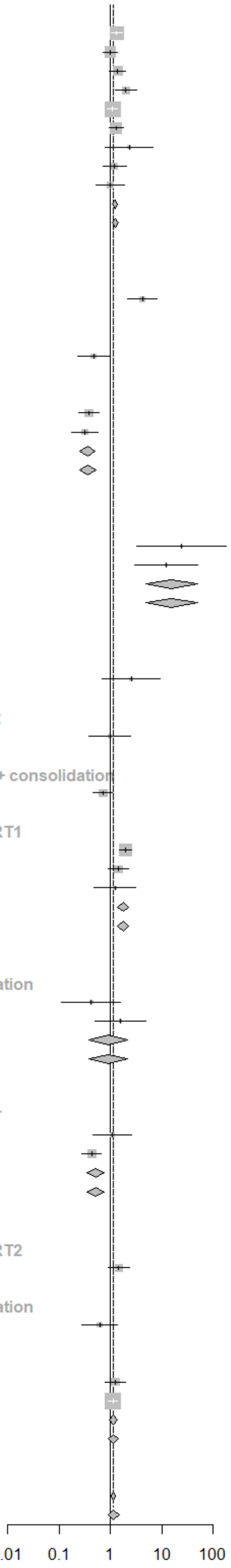

Heterogeneity: $I^2 = 80\%$, $\tau^2 = 0.3309$, $p < 0.01$
Residual heterogeneity: $I^2 = 16\%$, $\tau^2 = 0.0018$, $p = 0.27$
Test for subgroup differences (common effect): $\chi^2_{13} = 121.62$, df = 13 ($p < 0.01$)
Test for subgroup differences (random effects): $\chi^2_{13} = 118.15$, df = 13 ($p < 0.01$)

© 2024 Turri G et al. *JAMA Network Open*.

Network map The thickness of lines is proportional to the number of studies comparing the two treatments and the size of circles is proportional to the number of individuals for each treatment.

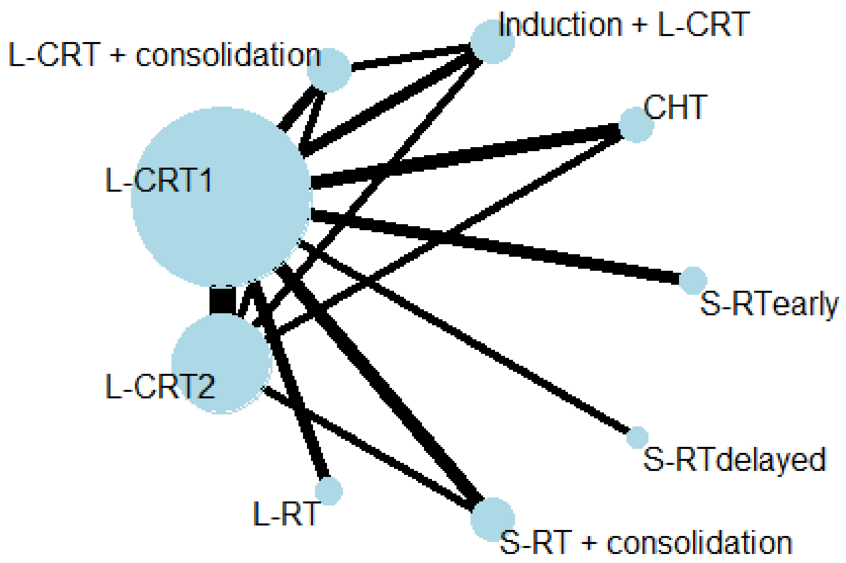

Netleague table Relative risks (RRs) and 95% confidence intervals (CIs) are reported. Results of the network meta-analysis are reported in the lower left part of the table and results from the pairwise meta-analysis are reported in the upper right part of the table. RRs higher than 1 favor the column-defining treatment.

	V1	V2	V3	V4	V5	V6	V7	V8	V9
1	CHT	.	.	0.80 (0.60 to 1.06)	0.24 (0.12 to 0.49)	.	.	.	.
2	0.47 (0.30 to 0.75)	Induction + L-CRT	0.71 (0.42 to 1.21)	1.85 (1.18 to 2.90)	0.97 (0.36 to 2.60)	.	.	.	.
3	0.39 (0.23 to 0.67)	0.83 (0.54 to 1.27)	L-CRT + consolidation	1.12 (0.46 to 2.72)	1.63 (0.70 to 3.77)	.	.	.	.
4	0.75 (0.56 to 0.99)	1.58 (1.09 to 2.28)	1.91 (1.21 to 3.00)	L-CRT1	0.80 (0.68 to 0.94)	2.81 (1.85 to 4.29)	0.59 (0.43 to 0.81)	2.52 (0.67 to 9.43)	15.23 (4.68 to 49.60)
5	0.58 (0.42 to 0.80)	1.23 (0.84 to 1.80)	1.48 (0.93 to 2.35)	0.78 (0.67 to 0.91)	L-CRT2	.	0.69 (0.39 to 1.22)	.	.
6	2.10 (1.27 to 3.49)	4.44 (2.54 to 7.76)	5.36 (2.89 to 9.97)	2.81 (1.85 to 4.29)	3.62 (2.31 to 5.66)	L-RT	.	.	.
7	0.43 (0.29 to 0.64)	0.91 (0.58 to 1.44)	1.10 (0.65 to 1.87)	0.58 (0.44 to 0.76)	0.74 (0.55 to 1.01)	0.21 (0.12 to 0.34)	S-RT + consolidation	.	.
8	1.88 (0.49 to 7.26)	3.97 (1.01 to 15.64)	4.80 (1.19 to 19.39)	2.52 (0.67 to 9.43)	3.24 (0.86 to 12.23)	0.89 (0.22 to 3.58)	4.35 (1.13 to 16.74)	S-RTdelayed	.
9	11.39 (3.38 to 38.34)	24.03 (6.98 to 82.74)	29.03 (8.19 to 102.87)	15.23 (4.68 to 49.60)	19.58 (5.95 to 64.40)	5.41 (1.55 to 18.95)	26.29 (7.82 to 88.38)	6.05 (1.03 to 35.54)	S-RTearly

Forest plot L-CRT-1 was used as a common comparator. RRs above 1 favor the strategy over the common comparator (L-CRT1).

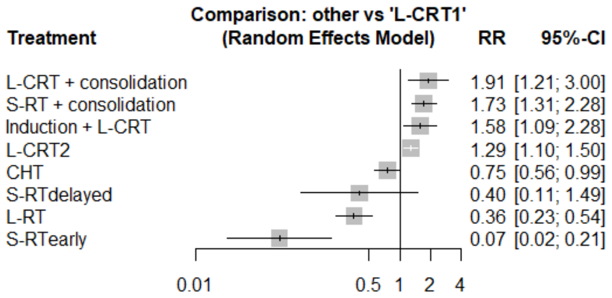

Assessment of heterogeneity and consistency

Global heterogeneity

© 2024 Turri G et al. *JAMA Network Open*.

We interpreted tau^2 as follows: heterogeneity low with tau^2≤0.010, moderate with 0.010<tau^2≤0.242, high with tau^2>0.242, and I^2 statistics as follows: not important (0%-40%), moderate (30%-60%), substantial (50%-90%), considerable (75%-100%).

```
## tau^2= 0.0237 ; tau= 0.154

## I^2= 30.79 % ( 0 % to 59.32 %)
```

Consistency: global approach

Q statistic to assess consistency under the assumption of a full design-by-treatment interaction random effects model

Q statistics to assess homogeneity / consistency

	Q	df	p-value
Total	28.90	20	0.0898
Within designs	13.79	14	0.4653
Between designs	15.10	6	0.0195

Design-specific decomposition of within-designs Q statistic

Design	Q	df	p-value
L-CRT1:CHT	0.21	1	0.6480
L-CRT1:Induction + L-CRT	3.66	1	0.0557
L-CRT1:L-CRT + consolidation	2.15	1	0.1424
L-CRT1:L-CRT2	5.13	7	0.6440
L-CRT1:L-RT	0.24	1	0.6209
L-CRT1:S-RT + consolidation	2.10	2	0.3496
L-CRT1:S-RTearly	0.29	1	0.5884

Between-designs Q statistic after detaching of single designs

Detached design	Q	df	p-value
Induction + L-CRT:L-CRT + consolidation	13.62	5	0.0182
Induction + L-CRT:L-CRT2	14.65	5	0.0120
L-CRT + consolidation:L-CRT2	15.10	5	0.0099
L-CRT1:CHT	7.39	5	0.1934
L-CRT1:Induction + L-CRT	12.83	5	0.0251
L-CRT1:L-CRT + consolidation	12.77	5	0.0256
L-CRT1:L-CRT2	9.66	5	0.0855
L-CRT1:S-RT + consolidation	15.09	5	0.0100
L-CRT2:S-RT + consolidation	15.09	5	0.0100
L-CRT1:CHT:L-CRT2	3.48	4	0.4810

Q statistic to assess consistency under the assumption of a full design-by-treatment interaction random effects model

	Q	df	p-value	tau.within	tau2.within
Between designs	15.10	6	0.0195	0	0

Consistency: local approach

Separate indirect from direct evidence (SIDE) using back-calculation method
Random effects model:

comparison	k	prop	nma	direct	indir.	RoR	z	p-value
L-CRT1:CHT	3	0.97	1.34	1.25	8.95	0.14	-2.46	0.0139
CHT:L-CRT2	1	0.19	0.58	0.24	0.72	0.33	-2.69	0.0071
Induction + L-CRT:L-CRT + consolidation	1	0.66	0.83	0.71	1.11	0.64	-0.97	0.3331
L-CRT1:Induction + L-CRT	2	0.66	0.63	0.54	0.87	0.62	-1.20	0.2317
Induction + L-CRT:L-CRT2	1	0.15	1.23	0.97	1.28	0.76	-0.50	0.6155
L-CRT1:L-CRT + consolidation	2	0.26	0.52	0.89	0.43	2.06	1.37	0.1708

L-CRT + consolidation:L-CRT2	1	0.30	1.48	1.63	1.42	1.14	0.26	0.7954
L-CRT1:L-CRT2	9	0.88	0.78	0.80	0.65	1.23	0.85	0.3973
L-CRT1:S-RT + consolidation	3	0.78	0.58	0.59	0.54	1.11	0.30	0.7677
L-CRT2:S-RT + consolidation	1	0.28	0.74	0.69	0.77	0.90	-0.30	0.7677

Legend:
comparison - Treatment comparison
k - Number of studies providing direct evidence
prop - Direct evidence proportion
nma - Estimated treatment effect (RR) in network meta-analysis
direct - Estimated treatment effect (RR) derived from direct evidence
indir. - Estimated treatment effect (RR) derived from indirect evidence
RoR - Ratio of Ratios (direct versus indirect)
z - z-value of test for disagreement (direct versus indirect)
p-value - p-value of test for disagreement (direct versus indirect)

Netrank Higher p-score values indicate higher ranking of treatments

	P-score
L-CRT + consolidation	0.9231
S-RT + consolidation	0.8703
Induction + L-CRT	0.7956
L-CRT2	0.6474
L-CRT1	0.4880
CHT	0.3553
S-RTdelayed	0.2374
L-RT	0.1794

© 2024 Turri G et al. *JAMA Network Open*.

eAppendix 7. Tolerability of treatment

Characteristics of the network

Number of treatments:

```
8
```

Number of studies:

```
25
```

Number of individuals included:

```
11987
```

Number of individuals randomized to each treatment:

	Treatment name	N. individuals randomized
1	CHT	1050
2	Induction + L-CRT	469
3	L-CRT + consolidation	288
4	L-CRT1	5373
5	L-CRT2	2529
6	L-RT	872
7	S-RT + consolidation	1090
8	S-RTearly	316

Pairwise meta-analysis
Risk ratios above 1 favor the first treatment of the comparison.

© 2024 Turri G et al. *JAMA Network Open*.

Study	logRR	SE(logRR)	RR	95%-CI	Weight (common)	Weight (random)
**comparison = L-CRT2 VS L-CRT1**						
Rodel 2015	0.0579	0.0272	1.06	[1.00; 1.12]	5.1%	4.2%
Aschele 2011	-0.2124	0.0363	0.81	[0.75; 0.87]	2.9%	4.0%
Gerard (2) 2010	-0.0621	0.0205	0.94	[0.90; 0.98]	9.0%	4.3%
Deng 2016	0.0650	0.0483	1.07	[0.97; 1.17]	1.6%	3.8%
Schmoll 2021	-0.3001	0.0343	0.74	[0.69; 0.79]	3.2%	4.1%
Haddad 2017	-0.3831	0.1617	0.68	[0.50; 0.94]	0.1%	1.6%
Jiao 2015	-0.1054	0.0636	0.90	[0.79; 1.02]	0.9%	3.4%
Mohiuddin 2006	0.0063	0.0647	1.01	[0.89; 1.14]	0.9%	3.4%
Common effect model			**0.92**	**[0.90; 0.95]**	**23.6%**	--
Random effects model			**0.91**	**[0.84; 0.99]**	--	**28.8%**
Heterogeneity: $I^2 = 93\%$, $\tau^2 = 0.0110$, $p < 0.01$						
**comparison = L-CRT2 VS CHT**						
Deng 2016	-0.0741	0.0369	0.93	[0.86; 1.00]	2.7%	4.0%
**comparison = CHT VS L-CRT1**						
Deng 2016	0.1391	0.0428	1.15	[1.06; 1.25]	2.0%	3.9%
**comparison = L-RT VS L-CRT1**						
Bosset 2005	0.2087	0.0228	1.23	[1.18; 1.29]	7.2%	4.3%
Gerard 2006	0.2107	0.0291	1.23	[1.17; 1.31]	4.4%	4.2%
Common effect model			**1.23**	**[1.19; 1.28]**	**11.6%**	--
Random effects model			**1.23**	**[1.06; 1.43]**	--	**8.4%**
Heterogeneity: $I^2 = 0\%$, $\tau^2 = 0.0110$, $p = 0.96$						
**comparison = L-CRT1 VS S-RTearly**						
Bujko 2004	-0.0463	0.0237	0.95	[0.91; 1.00]	6.7%	4.2%
Ngan 2012	.	.			0.0%	0.0%
**comparison = Induction + L-CRT VS L-CRT2**						
Fernandez-Martos 2015	0.0679	0.0919	1.07	[0.89; 1.28]	0.4%	2.8%
**comparison = Induction + L-CRT VS L-CRT + consolidation**						
Fokas 2019	0.0597	0.0466	1.06	[0.97; 1.16]	1.7%	3.8%
**comparison = S-RT + consolidation VS L-CRT1**						
Bahadoer 2021	-0.0567	0.0260	0.94	[0.90; 0.99]	5.5%	4.2%
Jin 2022	-0.2192	0.0371	0.80	[0.75; 0.86]	2.7%	4.0%
Chakrabarti 2021	-0.0022	0.0469	1.00	[0.91; 1.09]	1.7%	3.8%
Common effect model			**0.91**	**[0.88; 0.95]**	**10.0%**	--
Random effects model			**0.91**	**[0.80; 1.03]**	--	**12.1%**
Heterogeneity: $I^2 = 88\%$, $\tau^2 = 0.0110$, $p < 0.01$						
**comparison = L-CRT1 VS L-CRT + consolidation**						
Kim 2018	0.0430	0.0549	1.04	[0.94; 1.16]	1.2%	3.6%
Moore 2017	0.0400	0.0400	1.04	[0.96; 1.13]	2.3%	4.0%
Common effect model			**1.04**	**[0.98; 1.11]**	**3.6%**	--
Random effects model			**1.04**	**[0.89; 1.22]**	--	**7.6%**
Heterogeneity: $I^2 = 0\%$, $\tau^2 = 0.0110$, $p = 0.96$						
**comparison = L-CRT1 VS Induction + L-CRT**						
Marechal 2012	0.0357	0.0357	1.04	[0.97; 1.11]	2.9%	4.1%
Conroy 2021	0.0966	0.0237	1.10	[1.05; 1.15]	6.7%	4.2%
Common effect model			**1.08**	**[1.04; 1.12]**	**9.6%**	--
Random effects model			**1.07**	**[0.92; 1.24]**	--	**8.3%**
Heterogeneity: $I^2 = 50\%$, $\tau^2 = 0.0110$, $p = 0.16$						
**comparison = S-RT + consolidation VS L-CRT2**						
Bujko (2) 2013	-0.0513	0.0654	0.95	[0.84; 1.08]	0.9%	3.4%
**comparison = L-CRT2 VS L-CRT + consolidation**						
Wang 2019	0.0000	0.0339	1.00	[0.94; 1.07]	3.3%	4.1%
**comparison = L-CRT1 VS CHT**						
Mei 2023	0.0527	0.0295	1.05	[0.99; 1.12]	4.3%	4.2%
Schrag 2023	-0.0003	0.0139	1.00	[0.97; 1.03]	19.5%	4.3%
Common effect model			**1.01**	**[0.98; 1.03]**	**23.8%**	--
Random effects model			**1.03**	**[0.88; 1.19]**	--	**8.5%**
Heterogeneity: $I^2 = 62\%$, $\tau^2 = 0.0110$, $p = 0.10$						
**Common effect model**			**1.01**	**[0.99; 1.02]**	**100.0%**	--
**Random effects model**			**0.99**	**[0.94; 1.04]**	--	**100.0%**

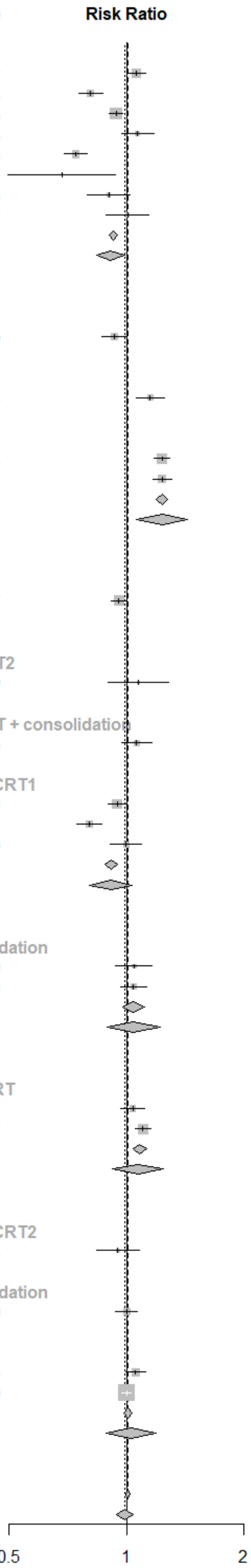

Heterogeneity: $I^2 = 93\%$, $\tau^2 = 0.0145$, $p < 0.01$
Residual heterogeneity: $I^2 = 89\%$, $\tau^2 = 0.0110$, $p < 0.01$
Test for subgroup differences (common effect): $\chi^2_{12} = 235.61$, df = 12 ($p < 0.01$)
Test for subgroup differences (random effects): $\chi^2_{12} = 18.71$, df = 12 ($p = 0.10$)

© 2024 Turri G et al. *JAMA Network Open*.

Network map The thickness of lines is proportional to the number of studies comparing the two treatments and the size of circles is proportional to the number of individuals for each treatment.

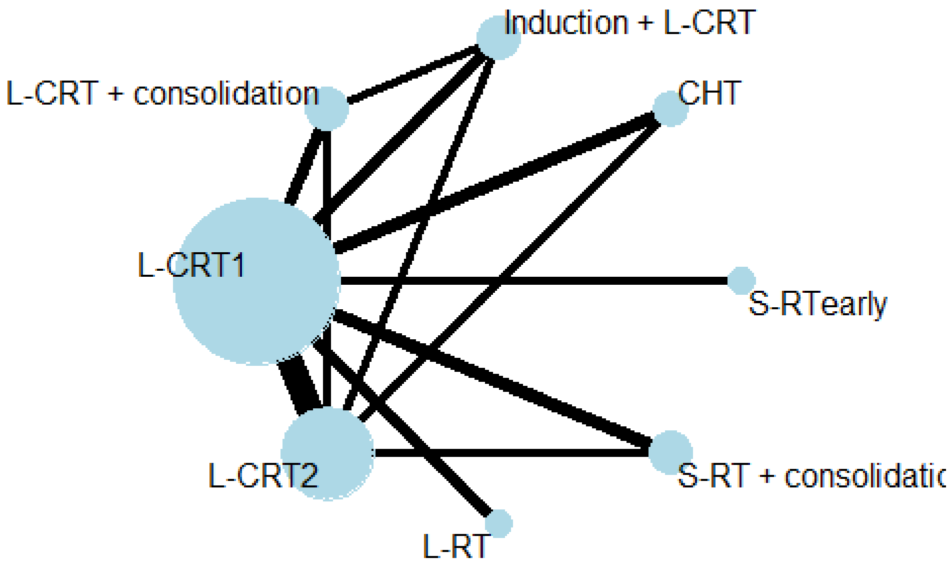

Netleague table Relative risks (RRs) and 95% confidence intervals (CIs) are reported. Results of the network meta-analysis are reported in the lower left part of the table and results from the pairwise meta-analysis are reported in the upper right part of the table. RRs higher than 1 favor the column-defining treatment.

	V1	V2	V3	V4	V5	V6	V7	V8
1	CHT	.	.	1.02 (0.92 to 1.14)	1.08 (0.90 to 1.29)	.	.	.
2	1.05 (0.92 to 1.21)	Induction + L-CRT	1.06 (0.88 to 1.28)	0.93 (0.82 to 1.06)	1.07 (0.84 to 1.37)	.	.	.
3	1.08 (0.94 to 1.24)	1.02 (0.91 to 1.16)	L-CRT + consolidation	0.96 (0.84 to 1.10)	1.00 (0.83 to 1.20)	.	.	.
4	1.01 (0.91 to 1.11)	0.95 (0.86 to 1.06)	0.93 (0.84 to 1.03)	L-CRT1	1.10 (1.02 to 1.18)	0.81 (0.72 to 0.92)	1.10 (0.99 to 1.22)	0.95 (0.80 to 1.14)
5	1.10 (0.99 to 1.23)	1.05 (0.94 to 1.17)	1.02 (0.92 to 1.14)	1.10 (1.03 to 1.17)	L-CRT2	.	1.05 (0.85 to 1.30)	.
6	0.82 (0.70 to 0.95)	0.77 (0.66 to 0.91)	0.76 (0.64 to 0.89)	0.81 (0.72 to 0.92)	0.74 (0.64 to 0.85)	L-RT	.	.
7	1.12 (0.97 to 1.28)	1.06 (0.92 to 1.22)	1.03 (0.90 to 1.18)	1.11 (1.01 to 1.22)	1.01 (0.91 to 1.13)	1.37 (1.17 to 1.60)	S-RT + consolidation	.
8	0.96 (0.79 to 1.17)	0.91 (0.75 to 1.11)	0.89 (0.73 to 1.09)	0.95 (0.80 to 1.14)	0.87 (0.72 to 1.05)	1.18 (0.95 to 1.46)	0.86 (0.71 to 1.05)	S-RTearly

Forest plot L-CRT-1 was used as a common comparator. RRs above 1 favor the strategy over the common comparator (L-CRT1).

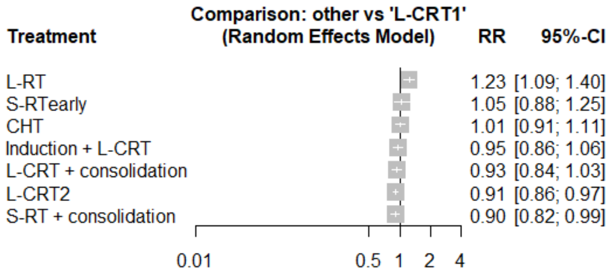

Assessment of heterogeneity and consistency

Global heterogeneity

© 2024 Turri G et al. *JAMA Network Open*.

We interpreted tau^2 as follows: heterogeneity low with tau^2≤0.010, moderate with 0.010<tau^2≤0.242, high with tau^2>0.242, and I^2 statistics as follows: not important (0%-40%), moderate (30%-60%), substantial (50%-90%), considerable (75%-100%).

```
tau^2= 0.0073 ; tau= 0.0856

I^2= 85.47 % ( 78.66 % to 90.11 %)
```

Consistency: global approach

Q statistic to assess consistency under the assumption of a full design-by-treatment interaction random effects model

```
Q statistics to assess homogeneity / consistency

                      Q df  p-value
Total            123.92 18 < 0.0001
Within designs   107.82 12 < 0.0001
Between designs   16.11  6   0.0132

Design-specific decomposition of within-designs Q statistic

                         Design     Q df  p-value
                    L-CRT1:CHT  2.64  1   0.1040
        L-CRT1:Induction + L-CRT  2.02  1   0.1555
     L-CRT1:L-CRT + consolidation  0.00  1   0.9648
                 L-CRT1:L-CRT2 85.91  6 < 0.0001
                    L-CRT1:L-RT  0.00  1   0.9569
      L-CRT1:S-RT + consolidation 17.25  2   0.0002

Between-designs Q statistic after detaching of single designs

                       Detached design     Q df p-value
 Induction + L-CRT:L-CRT + consolidation 14.30  5  0.0138
               Induction + L-CRT:L-CRT2 15.77  5  0.0075
            L-CRT + consolidation:L-CRT2 16.00  5  0.0069
                             L-CRT1:CHT 12.84  5  0.0249
                 L-CRT1:Induction + L-CRT 13.83  5  0.0167
             L-CRT1:L-CRT + consolidation 14.23  5  0.0142
                          L-CRT1:L-CRT2 14.70  5  0.0117
              L-CRT1:S-RT + consolidation 15.72  5  0.0077
              L-CRT2:S-RT + consolidation 15.72  5  0.0077
                      L-CRT1:CHT:L-CRT2  3.54  4  0.4717

Q statistic to assess consistency under the assumption of a full design-by-treatment interaction random effects model

                   Q df p-value tau.within tau2.within
Between designs 3.73  6  0.7133     0.0943      0.0089
```

Consistency: local approach

```
Separate indirect from direct evidence (SIDE) using back-calculation method

Random effects model:

                            comparison k prop  nma direct indir.  RoR     z p-value
                            L-CRT1:CHT 3 0.90 0.99   0.98   1.19 0.82 -1.15  0.2506
                            CHT:L-CRT2 1 0.35 1.10   1.08   1.12 0.96 -0.32  0.7468
Induction + L-CRT:L-CRT + consolidation 1 0.40 1.02   1.06   1.00 1.06  0.46  0.6419
              L-CRT1:Induction + L-CRT 2 0.64 1.05   1.07   1.01 1.06  0.55  0.5857
              Induction + L-CRT:L-CRT2 1 0.20 1.05   1.07   1.04 1.03  0.19  0.8461
          L-CRT1:L-CRT + consolidation 2 0.54 1.07   1.04   1.11 0.94 -0.63  0.5260
```

© 2024 Turri G et al. *JAMA Network Open*.

L-CRT + consolidation:L-CRT2	1	0.34	1.02	1.00	1.03	0.97	-0.29	0.7735
L-CRT1:L-CRT2	8	0.78	1.10	1.10	1.09	1.00	0.04	0.9708
L-CRT1:S-RT + consolidation	3	0.81	1.11	1.10	1.16	0.95	-0.43	0.6689
L-CRT2:S-RT + consolidation	1	0.25	1.01	1.05	1.00	1.05	0.43	0.6689

Legend:
comparison - Treatment comparison
k - Number of studies providing direct evidence
prop - Direct evidence proportion
nma - Estimated treatment effect (RR) in network meta-analysis
direct - Estimated treatment effect (RR) derived from direct evidence
indir. - Estimated treatment effect (RR) derived from indirect evidence
RoR - Ratio of Ratios (direct versus indirect)
z - z-value of test for disagreement (direct versus indirect)
p-value - p-value of test for disagreement (direct versus indirect)

Netrank Higher p-score values indicate higher ranking of treatments

	P-score
L-RT	0.9894
S-RTearly	0.7106
L-CRT1	0.6395
CHT	0.6333
Induction + L-CRT	0.4059
L-CRT + consolidation	0.2893
L-CRT2	0.1778
S-RT + consolidation	0.1542

© 2024 Turri G et al. *JAMA Network Open*.

eAppendix 8. Toxicity of treatment

Characteristics of the network

Number of treatments:

8

Number of studies:

22

Number of individuals included:

11568

Number of individuals randomized to each treatment:

	Treatment name	N. individuals randomized
1	CHT	885
2	Induction + L-CRT	469
3	L-CRT + consolidation	263
4	L-CRT1	5243
5	L-CRT2	3004
6	L-RT	367
7	S-RT + consolidation	1021
8	S-RTearly	316

Pairwise meta-analysis
Risk ratios above 1 favor the first treatment of the comparison.

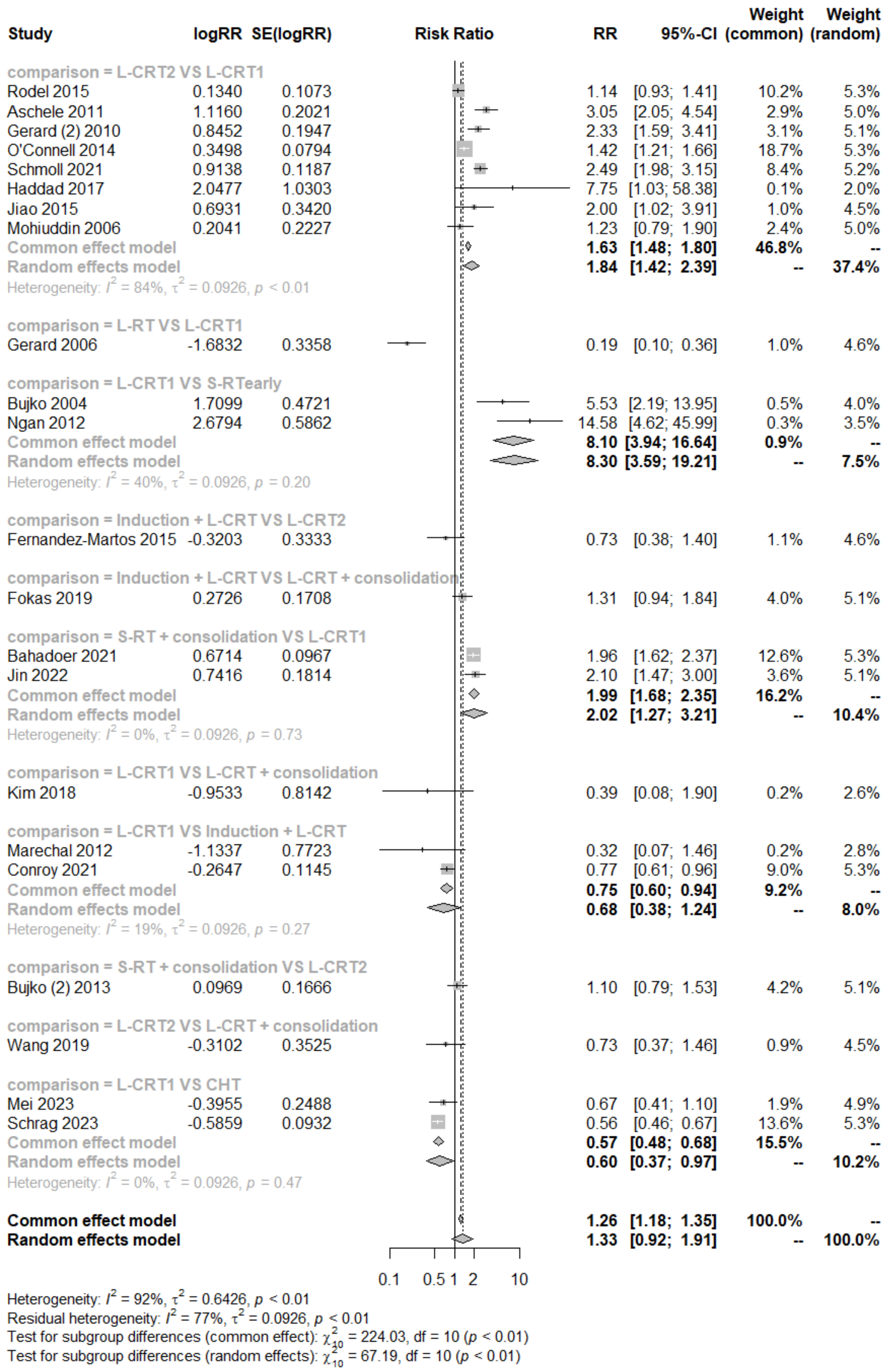

Network map The thickness of lines is proportional to the number of studies comparing the two treatments and the size of circles is proportional to the number of individuals for each treatment.

© 2024 Turri G et al. *JAMA Network Open*.

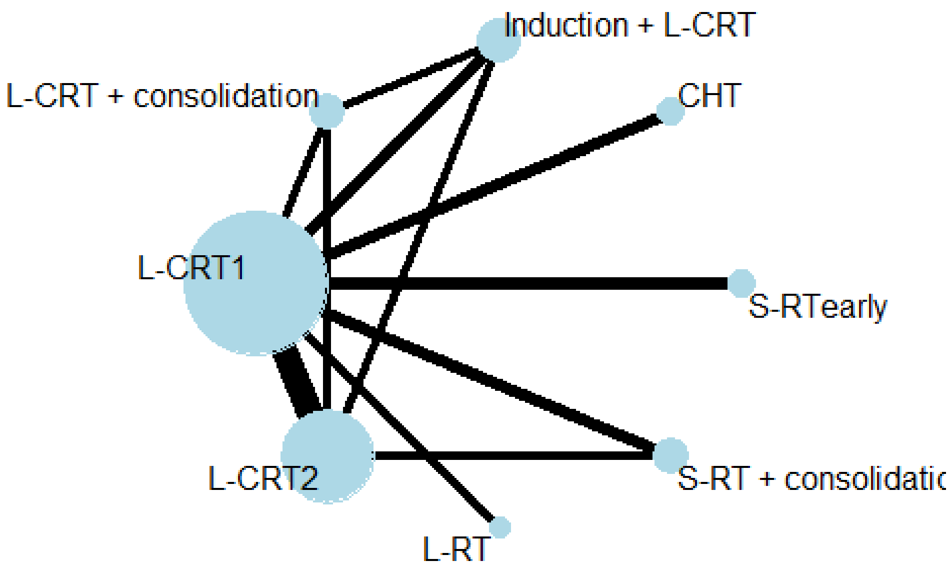

Netleague table Relative risks (RRs) and 95% confidence intervals (CIs) are reported. Results of the network meta-analysis are reported in the lower left part of the table and results from the pairwise meta-analysis are reported in the upper right part of the table. RRs higher than 1 favor the column-defining treatment.

	V1	V2	V3	V4	V5	V6	V7	V8
1	CHT	.	.	1.67 (1.06 to 2.64)	.	.	.	.
2	1.02 (0.54 to 1.93)	Induction + L-CRT	1.31 (0.69 to 2.51)	1.45 (0.83 to 2.54)	0.73 (0.31 to 1.71)	.	.	.
3	1.01 (0.49 to 2.11)	0.99 (0.58 to 1.68)	L-CRT + consolidation	2.59 (0.48 to 14.06)	1.36 (0.56 to 3.31)	.	.	.
4	1.67 (1.06 to 2.64)	1.63 (1.06 to 2.52)	1.65 (0.93 to 2.93)	L-CRT1	0.55 (0.43 to 0.70)	5.38 (2.27 to 12.74)	0.50 (0.32 to 0.77)	8.28 (3.63 to 18.89)
5	0.92 (0.55 to 1.54)	0.90 (0.58 to 1.41)	0.91 (0.51 to 1.61)	0.55 (0.44 to 0.69)	L-CRT2	.	0.91 (0.48 to 1.73)	.
6	8.99 (3.39 to 23.86)	8.78 (3.34 to 23.07)	8.88 (3.15 to 25.01)	5.38 (2.27 to 12.74)	9.75 (4.00 to 23.76)	L-RT	.	.
7	0.83 (0.46 to 1.50)	0.81 (0.46 to 1.42)	0.82 (0.42 to 1.60)	0.50 (0.34 to 0.72)	0.90 (0.61 to 1.34)	0.09 (0.04 to 0.24)	S-RT + consolidation	.
8	13.83 (5.38 to 35.52)	13.52 (5.32 to 34.34)	13.66 (5.00 to 37.31)	8.28 (3.63 to 18.89)	15.00 (6.38 to 35.27)	1.54 (0.47 to 5.07)	16.66 (6.75 to 41.12)	S-RTearly

Forest plot L-CRT-1 was used as a common comparator. RRs above 1 favor the strategy over the common comparator (L-CRT1).

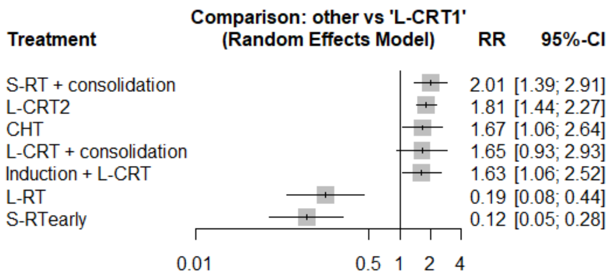

Assessment of heterogeneity and consistency

Global heterogeneity

We interpreted tau^2 as follows: heterogeneity low with tau^2≤0.010, moderate with 0.010<tau^2≤0.242, high with tau^2>0.242, and I^2 statistics as follows: not important (0%-40%), moderate (30%-60%), substantial (50%-90%), considerable (75%-100%).

© 2024 Turri G et al. *JAMA Network Open*.

```
tau^2= 0.0805 ; tau= 0.2837

I^2= 71.54 % ( 52.87 % to 82.82 %)
```

Consistency: global approach

Q statistic to assess consistency under the assumption of a full design-by-treatment interaction random effects model

```
Q statistics to assess homogeneity / consistency

                    Q df  p-value
Total           52.71 15 < 0.0001
Within designs  47.61 11 < 0.0001
Between designs  5.11  4   0.2765

Design-specific decomposition of within-designs Q statistic

                             Design     Q df  p-value
                        L-CRT1:CHT  0.51  1   0.4737
           L-CRT1:Induction + L-CRT  1.24  1   0.2657
                      L-CRT1:L-CRT2 44.08  7 < 0.0001
      L-CRT1:S-RT + consolidation  0.12  1   0.7328
                   L-CRT1:S-RTearly  1.66  1   0.1978

Between-designs Q statistic after detaching of single designs

                        Detached design    Q df p-value
Induction + L-CRT:L-CRT + consolidation 0.40  3  0.9403
               Induction + L-CRT:L-CRT2 4.82  3  0.1857
         L-CRT + consolidation:L-CRT2 1.66  3  0.6448
               L-CRT1:Induction + L-CRT 3.81  3  0.2827
         L-CRT1:L-CRT + consolidation 4.28  3  0.2329
                          L-CRT1:L-CRT2 5.11  3  0.1642
          L-CRT1:S-RT + consolidation 4.79  3  0.1882
          L-CRT2:S-RT + consolidation 4.79  3  0.1882

Q statistic to assess consistency under the assumption of a full design-by-treatment interaction random effects model

                   Q df p-value tau.within tau2.within
Between designs 2.00  4  0.7349     0.3105      0.0964
```

Consistency: local approach

```
Separate indirect from direct evidence (SIDE) using back-calculation method

Random effects model:

                             comparison k prop  nma direct indir.  RoR     z p-value
Induction + L-CRT:L-CRT + consolidation 1 0.67 0.99   1.31   0.56 2.35  1.48  0.1375
               L-CRT1:Induction + L-CRT 2 0.60 0.61   0.69   0.51 1.35  0.66  0.5094
               Induction + L-CRT:L-CRT2 1 0.27 0.90   0.73   0.98 0.74 -0.58  0.5623
         L-CRT1:L-CRT + consolidation 1 0.12 0.61   0.39   0.64 0.60 -0.56  0.5764
         L-CRT + consolidation:L-CRT2 1 0.41 0.91   1.36   0.69 1.99  1.17  0.2440
                          L-CRT1:L-CRT2 8 0.84 0.55   0.55   0.59 0.93 -0.25  0.8064
          L-CRT1:S-RT + consolidation 2 0.71 0.50   0.50   0.50 0.99 -0.03  0.9757
          L-CRT2:S-RT + consolidation 1 0.37 0.90   0.91   0.90 1.01  0.03  0.9757

Legend:
comparison - Treatment comparison
k          - Number of studies providing direct evidence
prop       - Direct evidence proportion
```

© 2024 Turri G et al. *JAMA Network Open*.

nma	- Estimated treatment effect (RR) in network meta-analysis
direct	- Estimated treatment effect (RR) derived from direct evidence
indir.	- Estimated treatment effect (RR) derived from indirect evidence
RoR	- Ratio of Ratios (direct versus indirect)
z	- z-value of test for disagreement (direct versus indirect)
p-value	- p-value of test for disagreement (direct versus indirect)

Netrank Higher p-score values indicate higher ranking of treatments

	P-score
S-RT + consolidation	0.8454
L-CRT2	0.7465
CHT	0.6675
L-CRT + consolidation	0.6590
Induction + L-CRT	0.6428
L-CRT1	0.2960
L-RT	0.1087
S-RTearly	0.0342

© 2024 Turri G et al. *JAMA Network Open*.

eAppendix 9. Dropouts by any cause

Characteristics of the network

Number of treatments:

9

Number of studies:

27

Number of individuals included:

13383

Number of individuals randomized to each treatment:

	Treatment name	N. individuals randomized
1	CHT	1050
2	Induction + L-CRT	469
3	L-CRT + consolidation	288
4	L-CRT1	6075
5	L-CRT2	3147
6	L-RT	872
7	S-RT + consolidation	1090
8	S-RTdelayed	75
9	S-RTearly	317

Pairwise meta-analysis
Risk ratios above 1 favor the first treatment of the comparison.

© 2024 Turri G et al. *JAMA Network Open*.

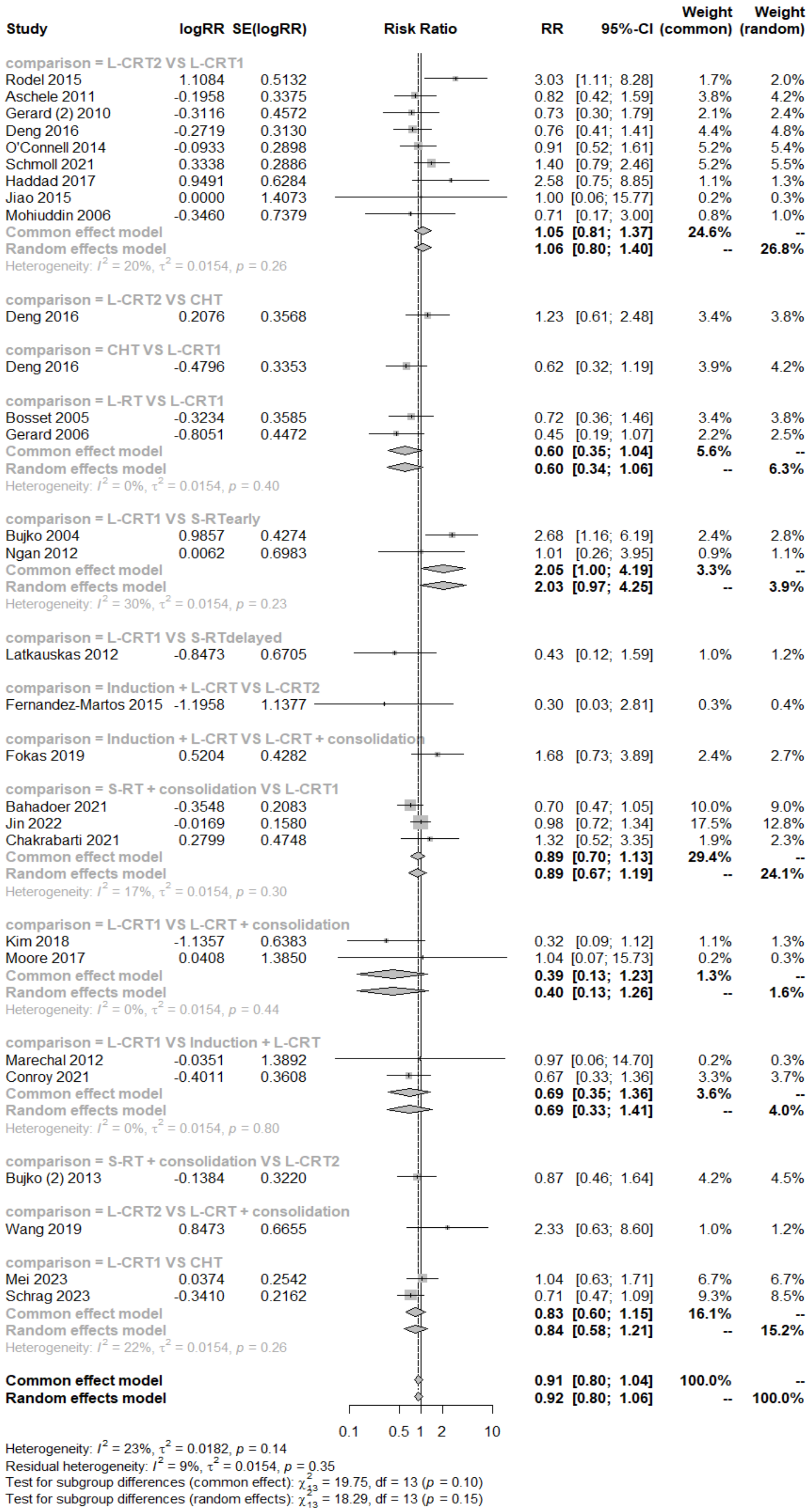

© 2024 Turri G et al. *JAMA Network Open*.

Network map The thickness of lines is proportional to the number of studies comparing the two treatments and the size of circles is proportional to the number of individuals for each treatment.

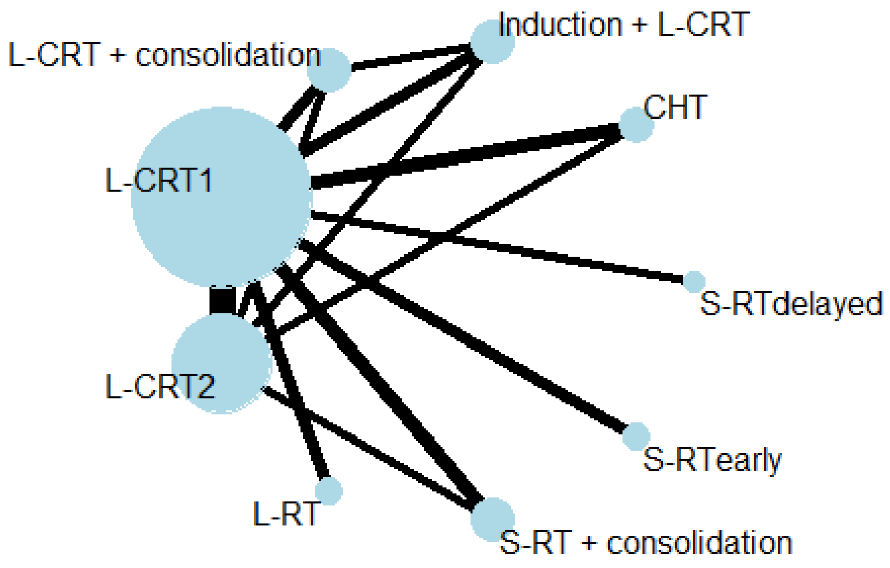

Netleague table Relative risks (RRs) and 95% confidence intervals (CIs) are reported. Results of the network meta-analysis are reported in the lower left part of the table and results from the pairwise meta-analysis are reported in the upper right part of the table. RRs higher than 1 favor the column-defining treatment.

	V1	V2	V3	V4	V5	V6	V7	V8	V9
1	CHT	.	.	1.02 (0.72 to 1.46)	0.81 (0.37 to 1.77)	.	.	.	.
2	0.74 (0.36 to 1.50)	Induction + L-CRT	1.68 (0.68 to 4.17)	1.45 (0.68 to 3.09)	0.30 (0.03 to 2.89)	.	.	.	.
3	1.02 (0.46 to 2.23)	1.38 (0.68 to 2.81)	L-CRT + consolidation	2.51 (0.78 to 8.10)	0.43 (0.11 to 1.65)	.	.	.	.
4	1.05 (0.74 to 1.48)	1.42 (0.76 to 2.66)	1.03 (0.51 to 2.09)	L-CRT1	0.94 (0.70 to 1.26)	1.68 (0.92 to 3.07)	1.12 (0.81 to 1.56)	2.01 (0.94 to 4.31)	0.43 (0.11 to 1.67)
5	0.91 (0.60 to 1.39)	1.24 (0.64 to 2.41)	0.90 (0.44 to 1.86)	0.87 (0.67 to 1.14)	L-CRT2	.	1.15 (0.56 to 2.35)	.	.
6	1.76 (0.88 to 3.53)	2.39 (1.00 to 5.70)	1.73 (0.68 to 4.38)	1.68 (0.92 to 3.07)	1.93 (1.00 to 3.72)	L-RT	.	.	.
7	1.15 (0.73 to 1.82)	1.56 (0.78 to 3.12)	1.13 (0.53 to 2.43)	1.10 (0.81 to 1.49)	1.26 (0.87 to 1.82)	0.65 (0.33 to 1.28)	S-RT + consolidation	.	.
8	2.11 (0.91 to 4.87)	2.86 (1.07 to 7.68)	2.07 (0.73 to 5.86)	2.01 (0.94 to 4.31)	2.31 (1.03 to 5.17)	1.20 (0.45 to 3.16)	1.83 (0.81 to 4.16)	S-RTearly	.
9	0.45 (0.11 to 1.82)	0.61 (0.14 to 2.72)	0.44 (0.10 to 2.04)	0.43 (0.11 to 1.67)	0.49 (0.12 to 1.96)	0.25 (0.06 to 1.12)	0.39 (0.10 to 1.57)	0.21 (0.04 to 1.01)	S-RTdelayed

Forest plot L-CRT-1 was used as a common comparator. RRs above 1 favor the strategy over the common comparator (L-CRT1).

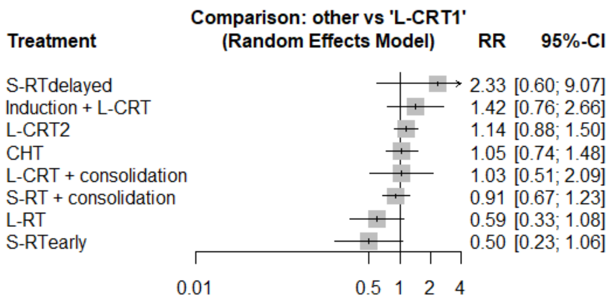

Assessment of heterogeneity and consistency

Global heterogeneity

© 2024 Turri G et al. *JAMA Network Open*.

We interpreted tau^2 as follows: heterogeneity low with tau^2≤0.010, moderate with 0.010<tau^2≤0.242, high with tau^2>0.242, and I^2 statistics as follows: not important (0%-40%), moderate (30%-60%), substantial (50%-90%), considerable (75%-100%).

```
tau^2= 0.0306 ; tau= 0.1748

I^2= 17.86 % ( 0 % to 51.45 %)
```

Consistency: global approach

Q statistic to assess consistency under the assumption of a full design-by-treatment interaction random effects model

Q statistics to assess homogeneity / consistency

	Q	df	p-value
Total	24.35	20	0.2274
Within designs	15.20	14	0.3648
Between designs	9.15	6	0.1652

Design-specific decomposition of within-designs Q statistic

Design	Q	df	p-value
L-CRT1:CHT	1.29	1	0.2569
L-CRT1:Induction + L-CRT	0.07	1	0.7987
L-CRT1:L-CRT + consolidation	0.60	1	0.4404
L-CRT1:L-CRT2	8.71	7	0.2740
L-CRT1:L-RT	0.71	1	0.4007
L-CRT1:S-RT + consolidation	2.40	2	0.3009
L-CRT1:S-RTearly	1.43	1	0.2315

Between-designs Q statistic after detaching of single designs

Detached design	Q	df	p-value
Induction + L-CRT:L-CRT + consolidation	8.67	5	0.1228
Induction + L-CRT:L-CRT2	7.42	5	0.1912
L-CRT + consolidation:L-CRT2	7.38	5	0.1942
L-CRT1:CHT	7.09	5	0.2143
L-CRT1:Induction + L-CRT	9.15	5	0.1034
L-CRT1:L-CRT + consolidation	5.41	5	0.3680
L-CRT1:L-CRT2	9.15	5	0.1033
L-CRT1:S-RT + consolidation	9.07	5	0.1063
L-CRT2:S-RT + consolidation	9.07	5	0.1063
L-CRT1:CHT:L-CRT2	5.49	4	0.2406

Q statistic to assess consistency under the assumption of a full design-by-treatment interaction random effects model

	Q	df	p-value	tau.within	tau2.within
Between designs	8.56	6	0.1999	0.1085	0.0118

Consistency: local approach

Separate indirect from direct evidence (SIDE) using back-calculation method

Random effects model:

comparison	k	prop	nma	direct	indir.	RoR	z	p-value
L-CRT1:CHT	3	0.96	0.95	0.98	0.52	1.87	0.68	0.4946
CHT:L-CRT2	1	0.29	0.91	0.81	0.96	0.85	-0.35	0.7229
Induction + L-CRT:L-CRT + consolidation	1	0.61	1.38	1.68	1.01	1.67	0.69	0.4920
L-CRT1:Induction + L-CRT	2	0.69	0.70	0.69	0.74	0.93	-0.10	0.9220
Induction + L-CRT:L-CRT2	1	0.09	1.24	0.30	1.42	0.21	-1.28	0.1993

© 2024 Turri G et al. *JAMA Network Open*.

L-CRT1:L-CRT + consolidation	2	0.36	0.97	0.40	1.62	0.25	-1.87	0.0619
L-CRT + consolidation:L-CRT2	1	0.29	0.90	0.43	1.22	0.35	-1.28	0.2009
L-CRT1:L-CRT2	9	0.82	0.87	0.94	0.62	1.52	1.17	0.2438
L-CRT1:S-RT + consolidation	3	0.85	1.10	1.12	0.99	1.13	0.29	0.7720
L-CRT2:S-RT + consolidation	1	0.27	1.26	1.15	1.30	0.88	-0.29	0.7720

Legend:
comparison - Treatment comparison
k - Number of studies providing direct evidence
prop - Direct evidence proportion
nma - Estimated treatment effect (RR) in network meta-analysis
direct - Estimated treatment effect (RR) derived from direct evidence
indir. - Estimated treatment effect (RR) derived from indirect evidence
RoR - Ratio of Ratios (direct versus indirect)
z - z-value of test for disagreement (direct versus indirect)
p-value - p-value of test for disagreement (direct versus indirect)

Netrank Higher p-score values indicate higher ranking of treatments

	P-score
S-RTdelayed	0.8802
Induction + L-CRT	0.7914
L-CRT2	0.6712
CHT	0.5525
L-CRT + consolidation	0.5192
L-CRT1	0.4899
S-RT + consolidation	0.3812
L-RT	0.1322
S-RTearly	0.0822

© 2024 Turri G et al. *JAMA Network Open*.

eAppendix 10. Pre-operative treatment related deaths

Characteristics of the network

Number of treatments:

8

Number of studies:

24

Number of individuals included:

11963

Number of individuals randomized to each treatment:

	Treatment name	N. individuals randomized
1	CHT	1050
2	Induction + L-CRT	469
3	L-CRT + consolidation	288
4	L-CRT1	5359
5	L-CRT2	2519
6	L-RT	872
7	S-RT + consolidation	1090
8	S-RTearly	316

Pairwise meta-analysis
Risk ratios above 1 favor the first treatment of the comparison.

© 2024 Turri G et al. *JAMA Network Open*.

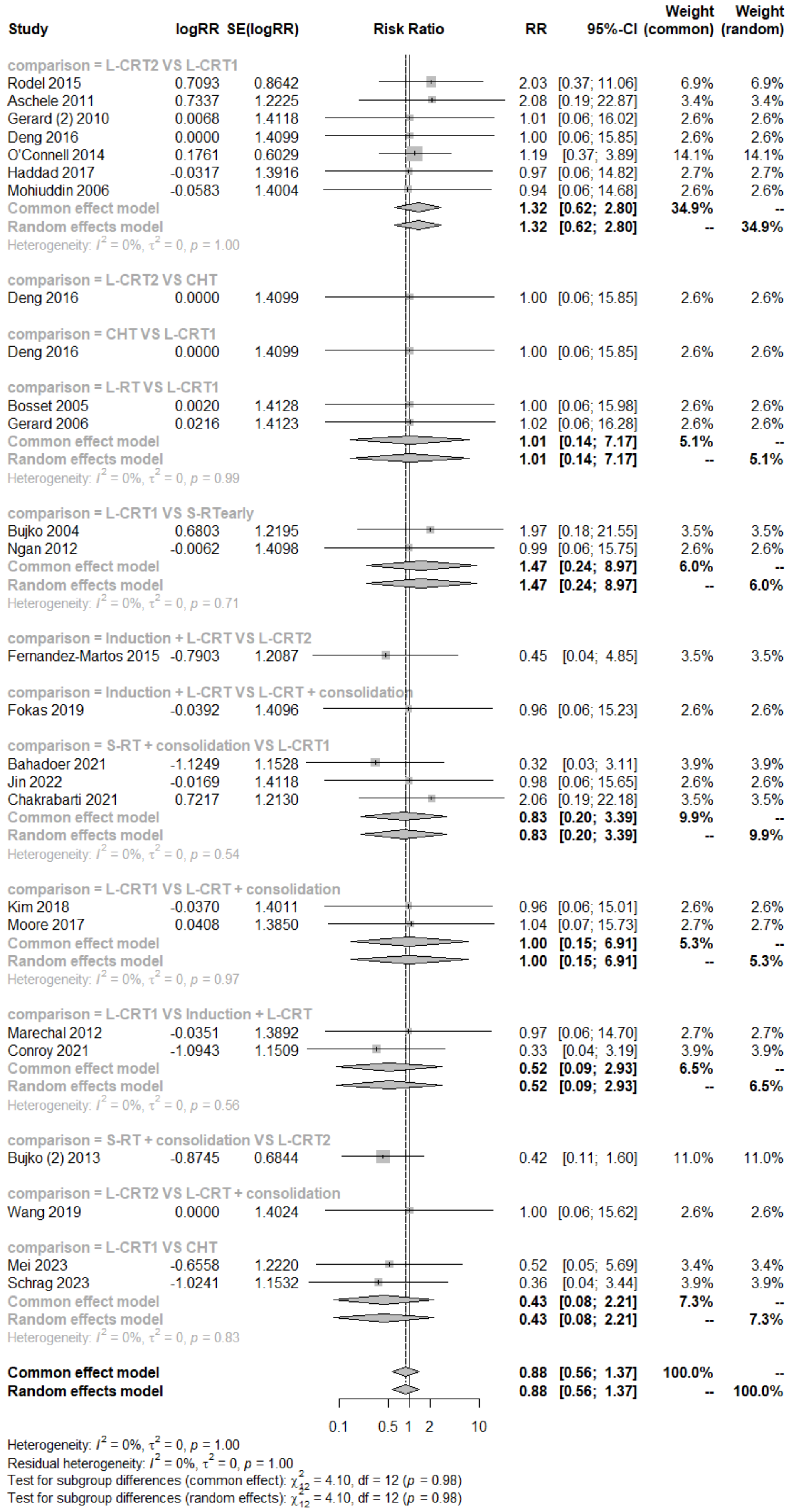

© 2024 Turri G et al. *JAMA Network Open*.

Network map The thickness of lines is proportional to the number of studies comparing the two treatments and the size of circles is proportional to the number of individuals for each treatment.

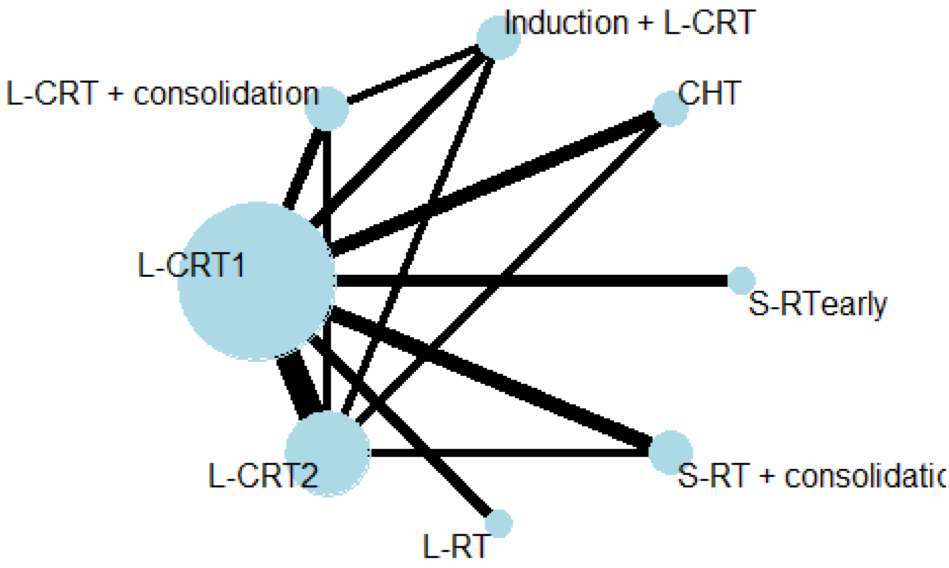

Netleague table Relative risks (RRs) and 95% confidence intervals (CIs) are reported. Results of the network meta-analysis are reported in the lower left part of the table and results from the pairwise meta-analysis are reported in the upper right part of the table. RRs higher than 1 favor the column-defining treatment.

	V1	V2	V3	V4	V5	V6	V7	V8
1	CHT	.	.	1.87 (0.46 to 7.70)	1.00 (0.06 to 15.85)	.	.	.
2	1.46 (0.23 to 9.50)	Induction + L-CRT	0.96 (0.06 to 15.23)	1.94 (0.34 to 11.03)	0.45 (0.04 to 4.85)	.	.	.
3	1.60 (0.23 to 11.33)	1.09 (0.20 to 5.85)	L-CRT + consolidation	1.00 (0.14 to 6.88)	1.00 (0.06 to 15.62)	.	.	.
4	1.90 (0.49 to 7.39)	1.30 (0.35 to 4.77)	1.19 (0.29 to 4.93)	L-CRT1	0.76 (0.36 to 1.61)	0.99 (0.14 to 7.00)	1.21 (0.29 to 4.94)	1.47 (0.24 to 8.97)
5	1.28 (0.30 to 5.55)	0.88 (0.23 to 3.39)	0.80 (0.18 to 3.50)	0.68 (0.35 to 1.31)	L-CRT2	.	2.40 (0.63 to 9.17)	.
6	1.88 (0.17 to 20.33)	1.28 (0.12 to 13.45)	1.17 (0.10 to 13.19)	0.99 (0.14 to 7.00)	1.46 (0.19 to 11.57)	L-RT	.	.
7	2.67 (0.50 to 14.41)	1.83 (0.36 to 9.16)	1.67 (0.30 to 9.28)	1.41 (0.50 to 3.95)	2.09 (0.75 to 5.79)	1.43 (0.16 to 13.03)	S-RT + consolidation	.
8	2.79 (0.29 to 26.82)	1.91 (0.21 to 17.71)	1.74 (0.17 to 17.43)	1.47 (0.24 to 8.97)	2.18 (0.32 to 14.95)	1.49 (0.10 to 21.39)	1.05 (0.13 to 8.38)	S-RTearly

Forest plot L-CRT-1 was used as a common comparator. RRs above 1 favor the strategy over the common comparator (L-CRT1).

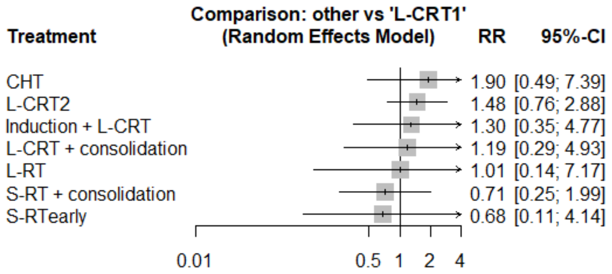

Assessment of heterogeneity and consistency

Global heterogeneity

© 2024 Turri G et al. *JAMA Network Open*.

We interpreted tau^2 as follows: heterogeneity low with tau^2≤0.010, moderate with 0.010<tau^2≤0.242, high with tau^2>0.242, and I^2 statistics as follows: not important (0%-40%), moderate (30%-60%), substantial (50%-90%), considerable (75%-100%).

```
tau^2= 0 ; tau= 0

I^2= 0 % ( 0 % to 48.92 %)
```

Consistency: global approach

Q statistic to assess consistency under the assumption of a full design-by-treatment interaction random effects model

Q statistics to assess homogeneity / consistency

	Q	df	p-value
Total	3.31	18	0.9999
Within designs	2.33	12	0.9987
Between designs	0.98	6	0.9863

Design-specific decomposition of within-designs Q statistic

Design	Q	df	p-value
L-CRT1:CHT	0.05	1	0.8265
L-CRT1:Induction + L-CRT	0.34	1	0.5571
L-CRT1:L-CRT + consolidation	0.00	1	0.9685
L-CRT1:L-CRT2	0.56	5	0.9898
L-CRT1:L-RT	0.00	1	0.9922
L-CRT1:S-RT + consolidation	1.24	2	0.5386
L-CRT1:S-RTearly	0.14	1	0.7127

Between-designs Q statistic after detaching of single designs

Detached design	Q	df	p-value
Induction + L-CRT:L-CRT + consolidation	0.97	5	0.9651
Induction + L-CRT:L-CRT2	0.54	5	0.9905
L-CRT + consolidation:L-CRT2	0.95	5	0.9668
L-CRT1:CHT	0.78	5	0.9780
L-CRT1:Induction + L-CRT	0.51	5	0.9919
L-CRT1:L-CRT + consolidation	0.91	5	0.9692
L-CRT1:L-CRT2	0.79	5	0.9780
L-CRT1:S-RT + consolidation	0.88	5	0.9714
L-CRT2:S-RT + consolidation	0.88	5	0.9714
L-CRT1:CHT:L-CRT2	0.71	4	0.9502

Q statistic to assess consistency under the assumption of a full design-by-treatment interaction random effects model

	Q	df	p-value	tau.within	tau2.within
Between designs	0.98	6	0.9863	0	0

Consistency: local approach

Separate indirect from direct evidence (SIDE) using back-calculation method

Random effects model:

comparison	k	prop	nma	direct	indir.	RoR	z	p-value
L-CRT1:CHT	3	0.93	0.53	0.53	0.45	1.18	0.06	0.9490
CHT:L-CRT2	1	0.28	1.28	1.00	1.41	0.71	-0.21	0.8355
Induction + L-CRT:L-CRT + consolidation	1	0.37	1.09	0.96	1.18	0.82	-0.11	0.9088
L-CRT1:Induction + L-CRT	2	0.56	0.77	0.52	1.30	0.40	-0.69	0.4909
Induction + L-CRT:L-CRT2	1	0.33	0.88	0.45	1.20	0.38	-0.66	0.5078

© 2024 Turri G et al. *JAMA Network Open*.

comparison	k	prop	nma	direct	indir.	RoR	z	p-value
L-CRT1:L-CRT + consolidation	2	0.54	0.84	1.00	0.69	1.46	0.26	0.7953
L-CRT + consolidation:L-CRT2	1	0.29	0.80	1.00	0.73	1.37	0.19	0.8509
L-CRT1:L-CRT2	7	0.78	0.68	0.76	0.45	1.69	0.64	0.5215
L-CRT1:S-RT + consolidation	3	0.54	1.41	1.21	1.68	0.72	-0.31	0.7532
L-CRT2:S-RT + consolidation	1	0.58	2.09	2.40	1.72	1.39	0.31	0.7532

Legend:
comparison - Treatment comparison
k - Number of studies providing direct evidence
prop - Direct evidence proportion
nma - Estimated treatment effect (RR) in network meta-analysis
direct - Estimated treatment effect (RR) derived from direct evidence
indir. - Estimated treatment effect (RR) derived from indirect evidence
RoR - Ratio of Ratios (direct versus indirect)
z - z-value of test for disagreement (direct versus indirect)
p-value - p-value of test for disagreement (direct versus indirect)

Netrank Higher p-score values indicate higher ranking of treatments

	P-score
CHT	0.7391
L-CRT2	0.6839
Induction + L-CRT	0.5751
L-CRT + consolidation	0.5297
L-RT	0.4674
L-CRT1	0.4224
S-RTearly	0.3156
S-RT + consolidation	0.2669

© 2024 Turri G et al. *JAMA Network Open*.

eAppendix 11. Rate of randomized patients who underwent surgery

Characteristics of the network

Characteristics of the network

Number of treatments:

```
9
```

Number of studies:

```
27
```

Number of individuals included:

```
13413
```

Number of individuals randomized to each treatment:

	Treatment name	N. individuals randomized
1	CHT	1050
2	Induction + L-CRT	469
3	L-CRT + consolidation	288
4	L-CRT1	6084
5	L-CRT2	3169
6	L-RT	872
7	S-RT + consolidation	1090
8	S-RTdelayed	75
9	S-RTearly	316

Pairwise meta-analysis
Risk ratios above 1 favor the first treatment of the comparison.

Study	logRR	SE(logRR)	RR	95%-CI	Weight (common)	Weight (random)
comparison = L-CRT2 VS L-CRT1						
Rodel 2015	-0.0152	0.0082	0.98	[0.97; 1.00]	17.9%	8.6%
Aschele 2011	0.0097	0.0140	1.01	[0.98; 1.04]	6.1%	6.2%
Gerard (2) 2010	0.0068	0.0109	1.01	[0.99; 1.03]	10.1%	7.5%
Deng 2016	0.0341	0.0392	1.03	[0.96; 1.12]	0.8%	1.6%
O'Connell 2014	0.0035	0.0108	1.00	[0.98; 1.03]	10.3%	7.5%
Schmoll 2021	-0.0510	0.0164	0.95	[0.92; 0.98]	4.5%	5.4%
Haddad 2017	-0.1859	0.1178	0.83	[0.66; 1.05]	0.1%	0.2%
Jiao 2015	0.0000	0.0097	1.00	[0.98; 1.02]	12.9%	8.0%
Mohiuddin 2006	0.0251	0.0536	1.03	[0.92; 1.14]	0.4%	0.9%
Common effect model			**1.00**	**[0.99; 1.00]**	**63.1%**	--
Random effects model			**1.00**	**[0.98; 1.01]**	--	**46.0%**
Heterogeneity: $I^2 = 51\%$, $\tau^2 = 0.0002$, $p = 0.04$						
comparison = L-CRT2 VS CHT						
Deng 2016	-0.0199	0.0342	0.98	[0.92; 1.05]	1.0%	2.0%
comparison = CHT VS L-CRT1						
Deng 2016	0.0541	0.0374	1.06	[0.98; 1.14]	0.9%	1.8%
comparison = L-RT VS L-CRT1						
Bosset 2005	0.0083	0.0161	1.01	[0.98; 1.04]	4.7%	5.5%
Gerard 2006	0.0243	0.0131	1.02	[1.00; 1.05]	7.0%	6.6%
Common effect model			**1.02**	**[1.00; 1.04]**	**11.7%**	--
Random effects model			**1.02**	**[0.99; 1.05]**	--	**12.1%**
Heterogeneity: $I^2 = 0\%$, $\tau^2 = 0.0002$, $p = 0.44$						
comparison = L-CRT1 VS S-RTearly						
Bujko 2004	-0.0890	0.0322	0.91	[0.86; 0.97]	1.2%	2.2%
Ngan 2012	-0.0125	0.0177	0.99	[0.95; 1.02]	3.8%	5.0%
Common effect model			**0.97**	**[0.94; 1.00]**	**5.0%**	--
Random effects model			**0.97**	**[0.93; 1.00]**	--	**7.2%**
Heterogeneity: $I^2 = 77\%$, $\tau^2 = 0.0002$, $p = 0.04$						
comparison = L-CRT1 VS S-RTdelayed						
Latkauskas 2012	0.0572	0.0439	1.06	[0.97; 1.15]	0.6%	1.3%
comparison = Induction + L-CRT VS L-CRT2						
Fernandez-Martos 2015	0.0625	0.0361	1.06	[0.99; 1.14]	0.9%	1.9%
comparison = Induction + L-CRT VS L-CRT + consolidation						
Fokas 2019	-0.0392	0.0302	0.96	[0.91; 1.02]	1.3%	2.5%
comparison = S-RT + consolidation VS L-CRT1						
Bahadoer 2021	0.0367	0.0215	1.04	[0.99; 1.08]	2.6%	4.0%
Jin 2022	0.0046	0.0428	1.00	[0.92; 1.09]	0.7%	1.4%
Chakrabarti 2021	-0.0360	0.0609	0.96	[0.86; 1.09]	0.3%	0.7%
Common effect model			**1.02**	**[0.99; 1.06]**	**3.6%**	--
Random effects model			**1.02**	**[0.98; 1.06]**	--	**6.1%**
Heterogeneity: $I^2 = 0\%$, $\tau^2 = 0.0002$, $p = 0.47$						
comparison = L-CRT1 VS L-CRT + consolidation						
Kim 2018	0.1300	0.0701	1.14	[0.99; 1.31]	0.2%	0.6%
Moore 2017	0.0000	0.0404	1.00	[0.92; 1.08]	0.7%	1.5%
Common effect model			**1.03**	**[0.96; 1.11]**	**1.0%**	--
Random effects model			**1.03**	**[0.96; 1.11]**	--	**2.1%**
Heterogeneity: $I^2 = 61\%$, $\tau^2 = 0.0002$, $p = 0.11$						
comparison = L-CRT1 VS Induction + L-CRT						
Marechal 2012	0.0013	0.0505	1.00	[0.91; 1.11]	0.5%	1.0%
Conroy 2021	0.0275	0.0246	1.03	[0.98; 1.08]	2.0%	3.3%
Common effect model			**1.02**	**[0.98; 1.07]**	**2.5%**	--
Random effects model			**1.02**	**[0.97; 1.07]**	--	**4.4%**
Heterogeneity: $I^2 = 0\%$, $\tau^2 = 0.0002$, $p = 0.64$						
comparison = S-RT + consolidation VS L-CRT2						
Bujko (2) 2013	0.0104	0.0242	1.01	[0.96; 1.06]	2.1%	3.4%
comparison = L-CRT2 VS L-CRT + consolidation						
Wang 2019	-0.0728	0.0555	0.93	[0.83; 1.04]	0.4%	0.9%
comparison = L-CRT1 VS CHT						
Mei 2023	-0.0039	0.0267	1.00	[0.95; 1.05]	1.7%	3.0%
Schrag 2023	0.0266	0.0167	1.03	[0.99; 1.06]	4.3%	5.3%
Common effect model			**1.02**	**[0.99; 1.05]**	**6.0%**	--
Random effects model			**1.02**	**[0.98; 1.05]**	--	**8.3%**
Heterogeneity: $I^2 = 0\%$, $\tau^2 = 0.0002$, $p = 0.33$						
**Common effect model**			**1.00**	**[0.99; 1.01]**	**100.0%**	--
**Random effects model**			**1.00**	**[0.99; 1.01]**	--	**100.0%**

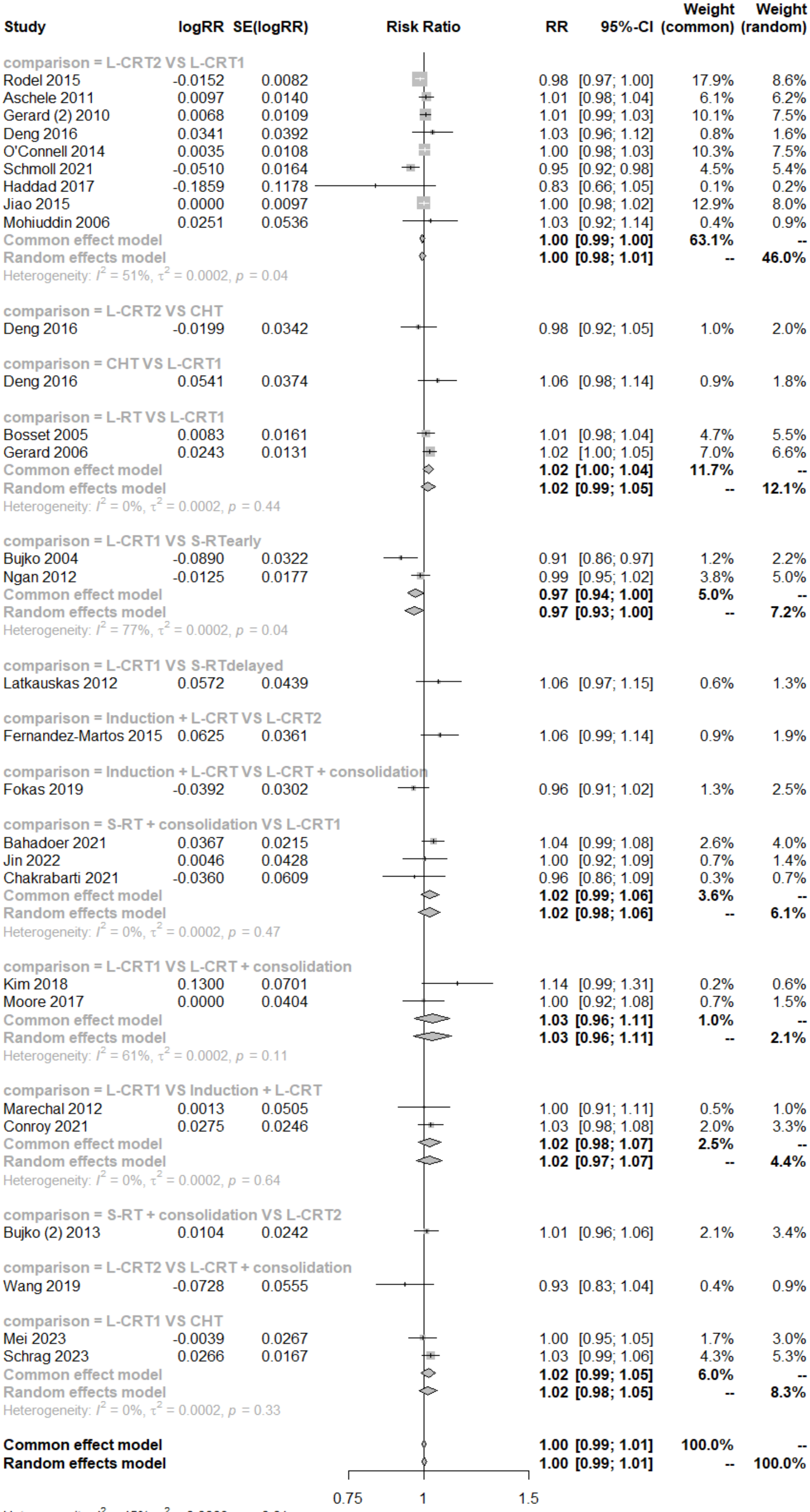

Heterogeneity: $I^2 = 45\%$, $\tau^2 = 0.0003$, $p < 0.01$
Residual heterogeneity: $I^2 = 43\%$, $\tau^2 = 0.0002$, $p = 0.03$
Test for subgroup differences (common effect): $\chi^2_{13} = 24.16$, df = 13 ($p = 0.03$)
Test for subgroup differences (random effects): $\chi^2_{13} = 17.94$, df = 13 ($p = 0.16$)

© 2024 Turri G et al. *JAMA Network Open*.

Network map The thickness of lines is proportional to the number of studies comparing the two treatments and the size of circles is proportional to the number of individuals for each treatment.

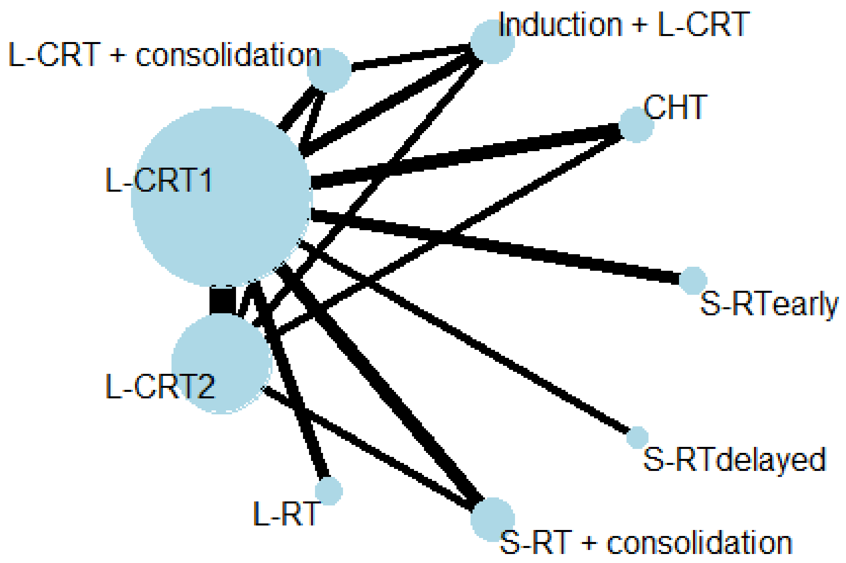

Netleague table Relative risks (RRs) and 95% confidence intervals (CIs) are reported. Results of the network meta-analysis are reported in the lower left part of the table and results from the pairwise meta-analysis are reported in the upper right part of the table. RRs higher than 1 favor the column-defining treatment.

	V1	V2	V3	V4	V5	V6	V7	V8	V9
1	CHT	.	.	1.00 (0.96 to 1.03)	1.02 (0.95 to 1.10)	.	.	.	.
2	1.00 (0.95 to 1.05)	Induction + L-CRT	0.96 (0.90 to 1.03)	0.98 (0.93 to 1.03)	1.06 (0.98 to 1.15)	.	.	.	.
3	0.98 (0.93 to 1.04)	0.98 (0.93 to 1.03)	L-CRT + consolidation	0.97 (0.90 to 1.04)	1.08 (0.96 to 1.20)	.	.	.	.
4	1.00 (0.96 to 1.03)	0.99 (0.96 to 1.03)	1.01 (0.96 to 1.06)	L-CRT1	1.00 (0.99 to 1.02)	0.98 (0.95 to 1.01)	0.98 (0.94 to 1.02)	1.06 (0.97 to 1.16)	0.96 (0.93 to 1.00)
5	1.00 (0.97 to 1.04)	1.00 (0.96 to 1.04)	1.02 (0.97 to 1.07)	1.01 (0.99 to 1.02)	L-CRT2	.	0.99 (0.93 to 1.05)	.	.
6	0.98 (0.94 to 1.02)	0.98 (0.93 to 1.03)	0.99 (0.94 to 1.05)	0.98 (0.95 to 1.01)	0.97 (0.94 to 1.01)	L-RT	.	.	.
7	0.98 (0.94 to 1.03)	0.98 (0.93 to 1.03)	1.00 (0.94 to 1.06)	0.99 (0.95 to 1.02)	0.98 (0.94 to 1.01)	1.00 (0.96 to 1.05)	S-RT + consolidation	.	.
8	1.05 (0.96 to 1.16)	1.05 (0.95 to 1.16)	1.07 (0.97 to 1.19)	1.06 (0.97 to 1.16)	1.05 (0.96 to 1.15)	1.08 (0.98 to 1.19)	1.07 (0.97 to 1.18)	S-RTdelayed	.
9	0.96 (0.91 to 1.01)	0.96 (0.91 to 1.01)	0.98 (0.92 to 1.04)	0.96 (0.93 to 1.00)	0.96 (0.92 to 1.00)	0.98 (0.93 to 1.03)	0.98 (0.93 to 1.03)	0.91 (0.82 to 1.01)	S-RTearly

Forest plot L-CRT-1 was used as a common comparator. RRs above 1 favor the strategy over the common comparator (L-CRT1).

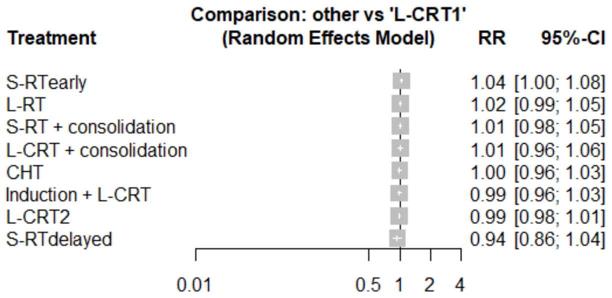

Assessment of heterogeneity and consistency

Global heterogeneity

© 2024 Turri G et al. *JAMA Network Open*.

We interpreted tau^2 as follows: heterogeneity low with tau^2≤0.010, moderate with 0.010<tau^2≤0.242, high with tau^2>0.242, and I^2 statistics as follows: not important (0%-40%), moderate (30%-60%), substantial (50%-90%), considerable (75%-100%).

tau^2= 3e-04 ; tau= 0.0164

I^2= 44.04 % (6.35 % to 66.56 %)

Consistency: global approach

Q statistic to assess consistency under the assumption of a full design-by-treatment interaction random effects model

Q statistics to assess homogeneity / consistency

	Q	df	p-value
Total	35.74	20	0.0165
Within designs	25.44	14	0.0304
Between designs	10.30	6	0.1127

Design-specific decomposition of within-designs Q statistic

Design	Q	df	p-value
L-CRT1:CHT	0.94	1	0.3320
L-CRT1:Induction + L-CRT	0.22	1	0.6403
L-CRT1:L-CRT + consolidation	2.58	1	0.1080
L-CRT1:L-CRT2	15.27	7	0.0327
L-CRT1:L-RT	0.60	1	0.4393
L-CRT1:S-RT + consolidation	1.52	2	0.4674
L-CRT1:S-RTearly	4.31	1	0.0378

Between-designs Q statistic after detaching of single designs

Detached design	Q	df	p-value
Induction + L-CRT:L-CRT + consolidation	9.45	5	0.0923
Induction + L-CRT:L-CRT2	6.15	5	0.2915
L-CRT + consolidation:L-CRT2	9.18	5	0.1020
L-CRT1:CHT	8.11	5	0.1505
L-CRT1:Induction + L-CRT	9.21	5	0.1010
L-CRT1:L-CRT + consolidation	7.36	5	0.1953
L-CRT1:L-CRT2	9.57	5	0.0882
L-CRT1:S-RT + consolidation	9.84	5	0.0798
L-CRT2:S-RT + consolidation	9.84	5	0.0798
L-CRT1:CHT:L-CRT2	7.05	4	0.1333

Q statistic to assess consistency under the assumption of a full design-by-treatment interaction random effects model

	Q	df	p-value	tau.within	tau2.within
Between designs	8.50	6	0.2037	0.0148	0.0002

Consistency: local approach

Separate indirect from direct evidence (SIDE) using back-calculation method

Random effects model:

comparison	k	prop	nma	direct	indir.	RoR	z	p-value
L-CRT1:CHT	3	0.92	1.00	1.00	1.02	0.99	-0.22	0.8271
CHT:L-CRT2	1	0.22	1.00	1.02	1.00	1.02	0.47	0.6363
Induction + L-CRT:L-CRT + consolidation	1	0.55	0.98	0.96	1.01	0.95	-0.90	0.3681
L-CRT1:Induction + L-CRT	2	0.58	1.01	1.02	0.99	1.03	0.85	0.3946
Induction + L-CRT:L-CRT2	1	0.26	1.00	1.06	0.98	1.09	1.79	0.0732

© 2024 Turri G et al. *JAMA Network Open*.

L-CRT1:L-CRT + consolidation	2	0.45	0.99	1.04	0.95	1.09	1.67	0.0952
L-CRT + consolidation:L-CRT2	1	0.19	1.02	1.08	1.01	1.07	1.01	0.3107
L-CRT1:L-CRT2	9	0.91	1.01	1.00	1.05	0.96	-1.51	0.1305
L-CRT1:S-RT + consolidation	3	0.66	0.99	0.98	1.00	0.98	-0.53	0.5961
L-CRT2:S-RT + consolidation	1	0.39	0.98	0.99	0.97	1.02	0.53	0.5961

Legend:
comparison - Treatment comparison
k - Number of studies providing direct evidence
prop - Direct evidence proportion
nma - Estimated treatment effect (RR) in network meta-analysis
direct - Estimated treatment effect (RR) derived from direct evidence
indir. - Estimated treatment effect (RR) derived from indirect evidence
RoR - Ratio of Ratios (direct versus indirect)
z - z-value of test for disagreement (direct versus indirect)
p-value - p-value of test for disagreement (direct versus indirect)

Netrank Higher p-score values indicate higher ranking of treatments

	P-score
S-RTearly	0.8927
L-RT	0.7185
S-RT + consolidation	0.6686
L-CRT + consolidation	0.6181
L-CRT1	0.4658
CHT	0.3896
Induction + L-CRT	0.3578
L-CRT2	0.2828
S-RTdelayed	0.1062

© 2024 Turri G et al. *JAMA Network Open*.

eAppendix 12. Rate of R0 resections

Characteristics of the network

Number of treatments:

```
8
```

Number of studies:

```
18
```

Number of individuals included:

```
9145
```

Number of individuals randomized to each treatment:

	Treatment name	N. individuals randomized
1	CHT	1050
2	Induction + L-CRT	441
3	L-CRT + consolidation	228
4	L-CRT1	4007
5	L-CRT2	2093
6	S-RT + consolidation	1090
7	S-RTdelayed	75
8	S-RTearly	161

Pairwise meta-analysis
Risk ratios above 1 favor the first treatment of the comparison.

© 2024 Turri G et al. *JAMA Network Open*.

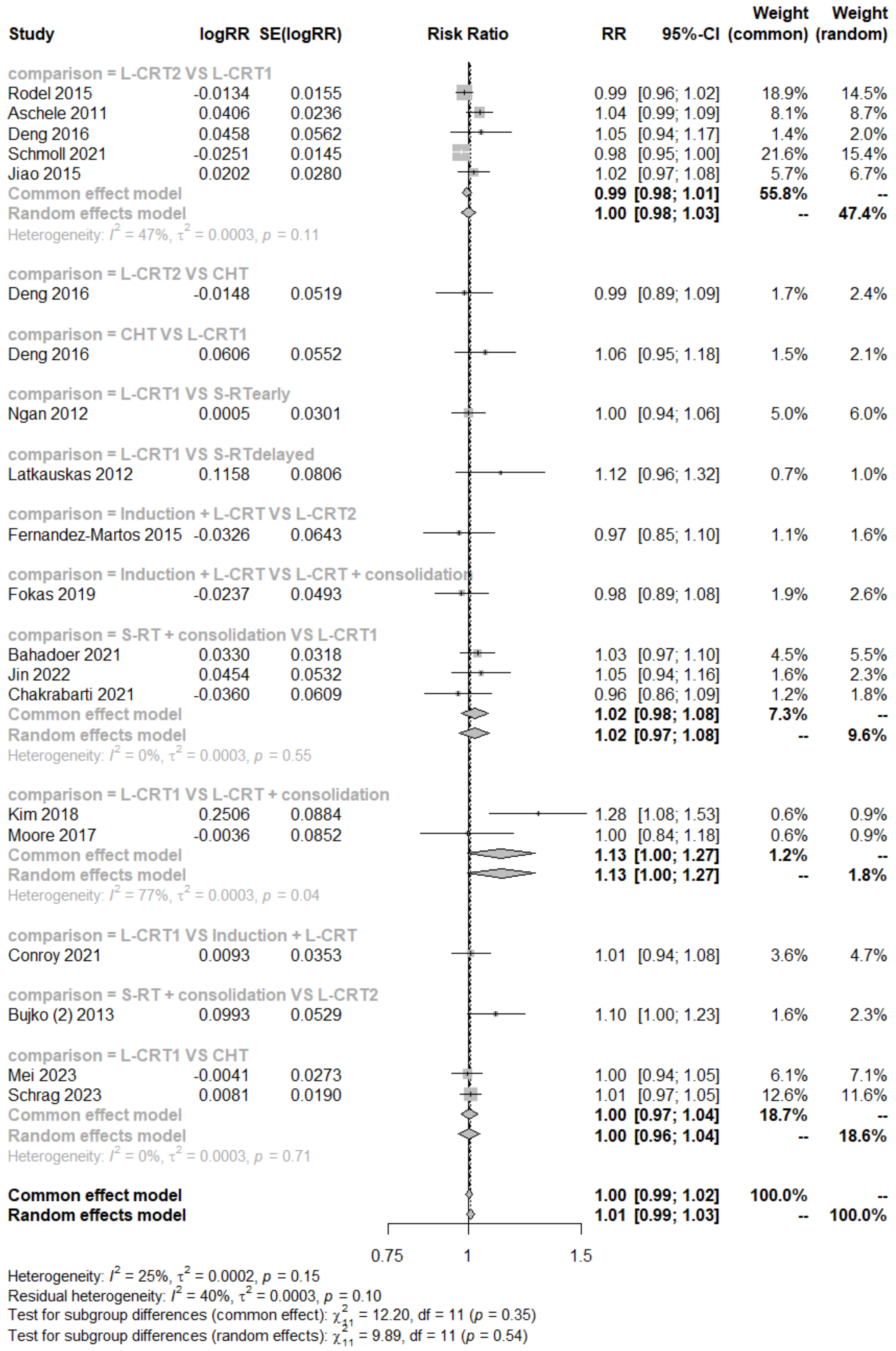

Network map The thickness of lines is proportional to the number of studies comparing the two treatments and the size of circles is proportional to the number of individuals for each treatment.

© 2024 Turri G et al. *JAMA Network Open*.

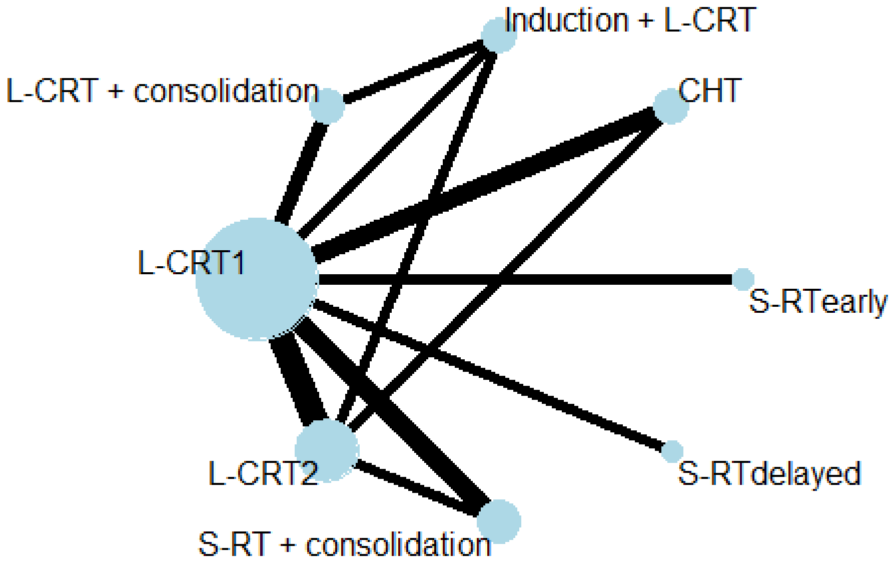

Netleague table Relative risks (RRs) and 95% confidence intervals (CIs) are reported. Results of the network meta-analysis are reported in the lower left part of the table and results from the pairwise meta-analysis are reported in the upper right part of the table. RRs higher than 1 favor the column-defining treatment.

	V1	V2	V3	V4	V5	V6	V7	V8
1	CHT	.	.	1.00 (0.97 to 1.05)	1.01 (0.91 to 1.13)	.	.	.
2	1.04 (0.96 to 1.12)	Induction + L-CRT	0.98 (0.88 to 1.08)	0.99 (0.91 to 1.07)	0.97 (0.85 to 1.10)	.	.	.
3	1.06 (0.96 to 1.17)	1.02 (0.94 to 1.11)	L-CRT + consolidation	0.89 (0.78 to 1.00)	.	.	.	.
4	1.00 (0.96 to 1.04)	0.97 (0.91 to 1.03)	0.95 (0.87 to 1.03)	L-CRT1	1.00 (0.97 to 1.03)	0.98 (0.92 to 1.03)	1.12 (0.95 to 1.32)	1.00 (0.93 to 1.07)
5	1.01 (0.96 to 1.05)	0.97 (0.91 to 1.03)	0.95 (0.87 to 1.04)	1.00 (0.98 to 1.03)	L-CRT2	0.91 (0.81 to 1.01)	.	.
6	0.97 (0.91 to 1.03)	0.93 (0.86 to 1.01)	0.91 (0.82 to 1.01)	0.96 (0.92 to 1.01)	0.96 (0.91 to 1.01)	S-RT + consolidation	.	.
7	1.13 (0.95 to 1.33)	1.08 (0.91 to 1.29)	1.06 (0.88 to 1.28)	1.12 (0.95 to 1.32)	1.12 (0.95 to 1.32)	1.17 (0.98 to 1.38)	S-RTdelayed	.
8	1.00 (0.93 to 1.09)	0.97 (0.88 to 1.06)	0.95 (0.84 to 1.06)	1.00 (0.93 to 1.07)	1.00 (0.92 to 1.08)	1.04 (0.95 to 1.13)	0.89 (0.75 to 1.06)	S-RTearly

Forest plot L-CRT-1 was used as a common comparator. RRs above 1 favor the strategy over the common comparator (L-CRT1).

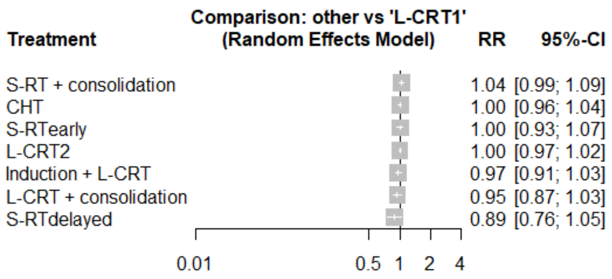

Assessment of heterogeneity and consistency

Global heterogeneity

We interpreted tau^2 as follows: heterogeneity low with tau^2≤0.010, moderate with 0.010<tau^2≤0.242, high with tau^2>0.242, and I^2 statistics as follows: not important (0%-40%), moderate (30%-60%), substantial (50%-90%), considerable (75%-100%).

© 2024 Turri G et al. *JAMA Network Open*.

```
tau^2= 4e-04 ; tau= 0.0204

I^2= 31.7 % ( 0 % to 64.7 %)
```

Consistency: global approach

Q statistic to assess consistency under the assumption of a full design-by-treatment interaction random effects model

```
Q statistics to assess homogeneity / consistency

                     Q df p-value
Total            17.57 12  0.1294
Within designs   12.39  7  0.0885
Between designs   5.18  5  0.3941

Design-specific decomposition of within-designs Q statistic

                         Design    Q df p-value
                    L-CRT1:CHT 0.13  1  0.7143
L-CRT1:L-CRT + consolidation 4.29  1  0.0384
                 L-CRT1:L-CRT2 6.76  3  0.0800
 L-CRT1:S-RT + consolidation 1.21  2  0.5459

Between-designs Q statistic after detaching of single designs

                      Detached design    Q df p-value
Induction + L-CRT:L-CRT + consolidation 2.96  4  0.5652
            Induction + L-CRT:L-CRT2 5.17  4  0.2704
                          L-CRT1:CHT 4.64  4  0.3264
            L-CRT1:Induction + L-CRT 3.79  4  0.4345
        L-CRT1:L-CRT + consolidation 2.96  4  0.5652
                       L-CRT1:L-CRT2 5.07  4  0.2803
         L-CRT1:S-RT + consolidation 3.80  4  0.4331
         L-CRT2:S-RT + consolidation 3.80  4  0.4331
                  L-CRT1:CHT:L-CRT2 3.75  3  0.2902

Q statistic to assess consistency under the assumption of a full design-by-treatment interaction random effects model

                   Q df p-value tau.within tau2.within
Between designs 4.55  5  0.4738     0.0220      0.0005
```

Consistency: local approach

```
Separate indirect from direct evidence (SIDE) using back-calculation method

Random effects model:

                             comparison k prop  nma direct indir.  RoR     z p-value
                             L-CRT1:CHT 3 0.95 1.00   1.00   1.03 0.97 -0.34  0.7338
                            CHT:L-CRT2 1 0.17 1.01   1.01   1.00 1.01  0.18  0.8568
Induction + L-CRT:L-CRT + consolidation 1 0.65 1.02   0.98   1.11 0.88 -1.41  0.1581
               L-CRT1:Induction + L-CRT 1 0.63 1.04   1.01   1.08 0.93 -1.05  0.2942
               Induction + L-CRT:L-CRT2 1 0.25 0.97   0.97   0.97 1.00  0.00  0.9989
           L-CRT1:L-CRT + consolidation 2 0.51 1.06   1.13   0.99 1.14  1.41  0.1581
                          L-CRT1:L-CRT2 5 0.91 1.00   1.00   1.05 0.95 -1.17  0.2422
            L-CRT1:S-RT + consolidation 3 0.81 0.96   0.98   0.90 1.08  1.19  0.2339
            L-CRT2:S-RT + consolidation 1 0.23 0.96   0.91   0.98 0.93 -1.19  0.2339

Legend:
comparison - Treatment comparison
k          - Number of studies providing direct evidence
```

© 2024 Turri G et al. *JAMA Network Open*.

prop	- Direct evidence proportion
nma	- Estimated treatment effect (RR) in network meta-analysis
direct	- Estimated treatment effect (RR) derived from direct evidence
indir.	- Estimated treatment effect (RR) derived from indirect evidence
RoR	- Ratio of Ratios (direct versus indirect)
z	- z-value of test for disagreement (direct versus indirect)
p-value	- p-value of test for disagreement (direct versus indirect)

Netrank Higher p-score values indicate higher ranking of treatments

	P-score
S-RT + consolidation	0.9156
CHT	0.6396
L-CRT1	0.6114
S-RTearly	0.5972
L-CRT2	0.5698
Induction + L-CRT	0.3192
L-CRT + consolidation	0.2265
S-RTdelayed	0.1206

© 2024 Turri G et al. *JAMA Network Open*.

eAppendix 13. Rate of negative CRM

Characteristics of the network

Number of treatments:

```
8
```

Number of studies:

```
11
```

Number of individuals included:

```
4963
```

Number of individuals randomized to each treatment:

	Treatment name	N. individuals randomized
1	CHT	585
2	Induction + L-CRT	415
3	L-CRT + consolidation	203
4	L-CRT1	2312
5	L-CRT2	756
6	S-RT + consolidation	462
7	S-RTdelayed	75
8	S-RTearly	155

Pairwise meta-analysis
Risk ratios above 1 favor the first treatment of the comparison.

© 2024 Turri G et al. *JAMA Network Open*.

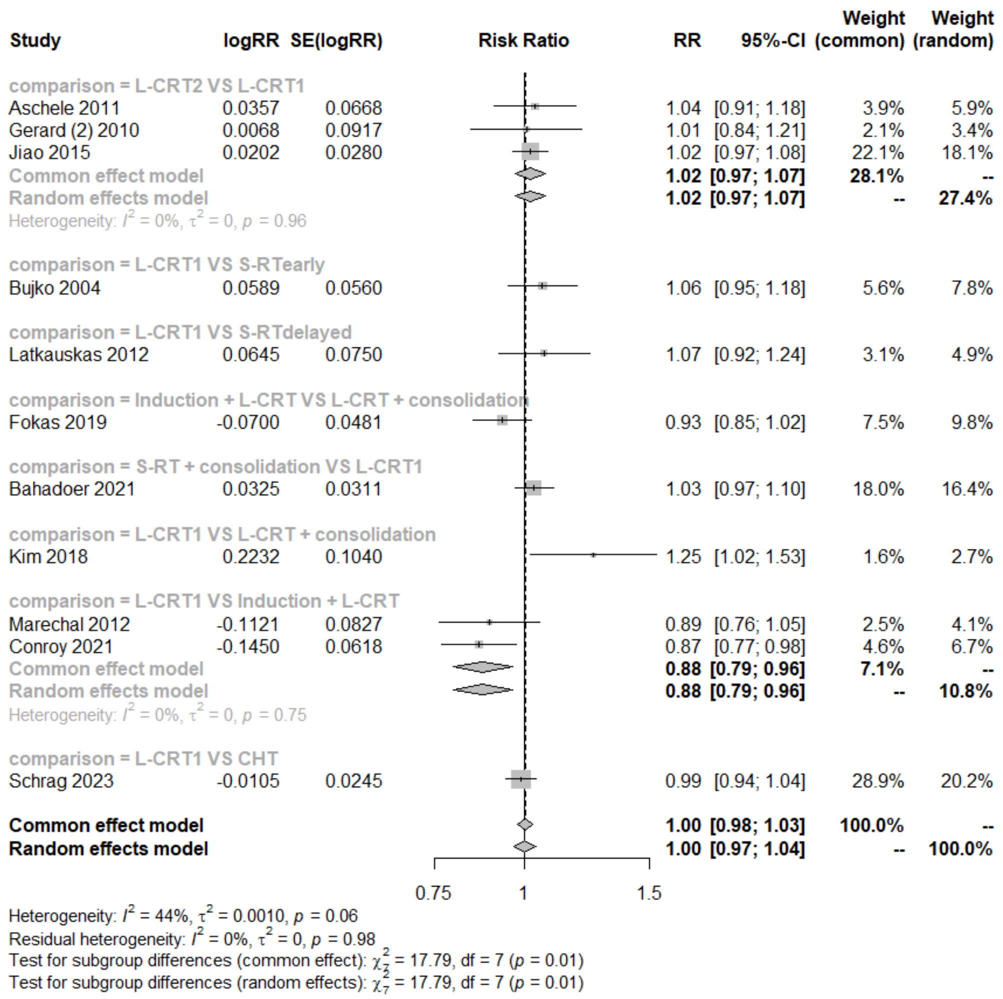

Network map The thickness of lines is proportional to the number of studies comparing the two treatments and the size of circles is proportional to the number of individuals for each treatment.

© 2024 Turri G et al. *JAMA Network Open*.

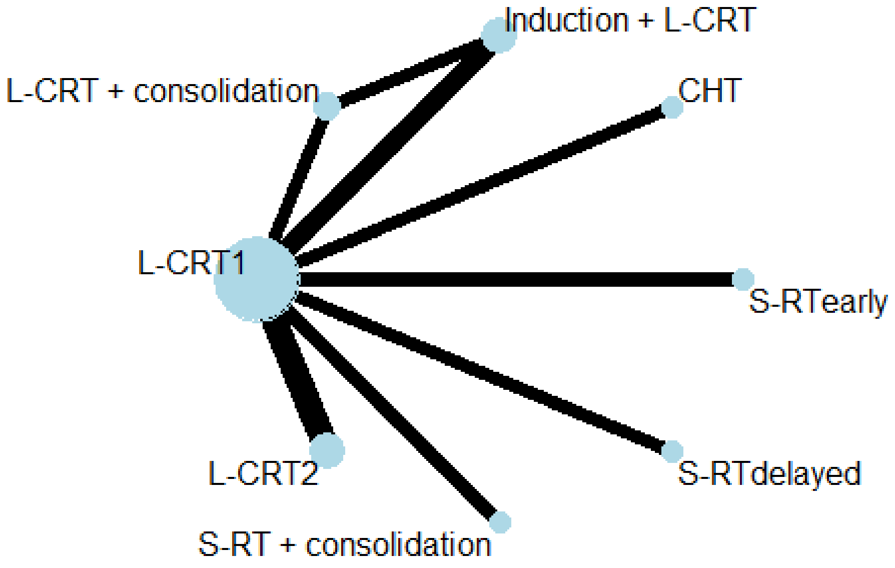

Netleague table Relative risks (RRs) and 95% confidence intervals (CIs) are reported. Results of the network meta-analysis are reported in the lower left part of the table and results from the pairwise meta-analysis are reported in the upper right part of the table. RRs higher than 1 favor the column-defining treatment.

	V1	V2	V3	V4	V5	V6	V7	V8
1	CHT	.	.	1.01 (0.84 to 1.22)	.	.	.	.
2	0.96 (0.76 to 1.22)	Induction + L-CRT	0.93 (0.76 to 1.14)	1.14 (0.97 to 1.34)	.	.	.	.
3	1.01 (0.78 to 1.32)	1.06 (0.89 to 1.25)	L-CRT + consolidation	0.80 (0.61 to 1.05)	.	.	.	.
4	1.01 (0.84 to 1.22)	1.05 (0.91 to 1.22)	1.00 (0.83 to 1.20)	L-CRT1	0.98 (0.86 to 1.11)	0.97 (0.80 to 1.17)	1.07 (0.85 to 1.35)	1.06 (0.86 to 1.31)
5	0.99 (0.79 to 1.24)	1.03 (0.85 to 1.25)	0.98 (0.78 to 1.22)	0.98 (0.86 to 1.11)	L-CRT2	.	.	.
6	0.98 (0.75 to 1.28)	1.02 (0.80 to 1.30)	0.97 (0.74 to 1.26)	0.97 (0.80 to 1.17)	0.99 (0.79 to 1.24)	S-RT + consolidation	.	.
7	1.08 (0.80 to 1.45)	1.12 (0.85 to 1.48)	1.06 (0.79 to 1.44)	1.07 (0.85 to 1.35)	1.09 (0.84 to 1.42)	1.10 (0.82 to 1.49)	S-RTdelayed	.
8	1.07 (0.81 to 1.42)	1.12 (0.86 to 1.44)	1.06 (0.80 to 1.41)	1.06 (0.86 to 1.31)	1.08 (0.85 to 1.39)	1.10 (0.82 to 1.46)	0.99 (0.73 to 1.36)	S-RTearly

Forest plot L-CRT-1 was used as a common comparator. RRs above 1 favor the strategy over the common comparator (L-CRT1).

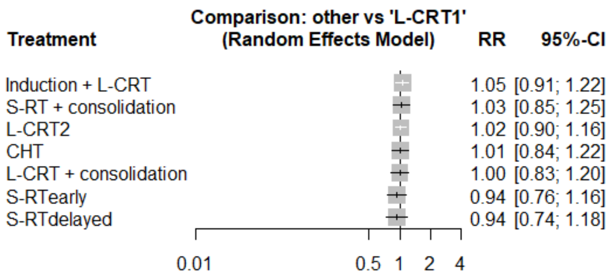

Assessment of heterogeneity and consistency

Global heterogeneity

We interpreted tau^2 as follows: heterogeneity low with tau^2≤0.010, moderate with 0.010<tau^2≤0.242, high with tau^2>0.242, and I^2 statistics as follows: not important (0%-40%), moderate (30%-60%), substantial (50%-90%), considerable (75%-100%).

© 2024 Turri G et al. *JAMA Network Open*.

```
tau^2= 0.0085 ; tau= 0.0922

I^2= 66.25 % ( 12.07 % to 87.05 %)
```

Consistency: global approach

Q statistic to assess consistency under the assumption of a full design-by-treatment interaction random effects model

```
Q statistics to assess homogeneity / consistency

                      Q df p-value
Total             11.85  4  0.0185
Within designs     0.17  3  0.9816
Between designs   11.68  1  0.0006

Design-specific decomposition of within-designs Q statistic

                      Design    Q df p-value
 L-CRT1:Induction + L-CRT 0.10  1  0.7495
          L-CRT1:L-CRT2 0.07  2  0.9644

Between-designs Q statistic after detaching of single designs

                       Detached design    Q df p-value
 Induction + L-CRT:L-CRT + consolidation 0.00  0      --
              L-CRT1:Induction + L-CRT 0.00  0      --
           L-CRT1:L-CRT + consolidation 0.00  0      --

Q statistic to assess consistency under the assumption of a full design-by-treatment interaction random effects model

                    Q df p-value tau.within tau2.within
Between designs 11.68  1  0.0006          0           0
```

Consistency: local approach

```
Separate indirect from direct evidence (SIDE) using back-calculation method

Random effects model:

                             comparison k prop  nma direct indir.  RoR     z p-value
Induction + L-CRT:L-CRT + consolidation 1 0.71 1.06   0.93   1.42 0.65 -2.20  0.0276
              L-CRT1:Induction + L-CRT 2 0.82 0.95   0.88   1.34 0.65 -2.20  0.0276
          L-CRT1:L-CRT + consolidation 1 0.48 1.00   1.25   0.82 1.53  2.20  0.0276

Legend:
 comparison - Treatment comparison
 k          - Number of studies providing direct evidence
 prop       - Direct evidence proportion
 nma        - Estimated treatment effect (RR) in network meta-analysis
 direct     - Estimated treatment effect (RR) derived from direct evidence
 indir.     - Estimated treatment effect (RR) derived from indirect evidence
 RoR        - Ratio of Ratios (direct versus indirect)
 z          - z-value of test for disagreement (direct versus indirect)
 p-value    - p-value of test for disagreement (direct versus indirect)
```

Netrank Higher p-score values indicate higher ranking of treatments

```
                    P-score
Induction + L-CRT    0.7006
S-RT + consolidation 0.6056
L-CRT2               0.5824
```

© 2024 Turri G et al. *JAMA Network Open*.

CHT	0.5310
L-CRT1	0.4795
L-CRT + consolidation	0.4794
S-RTearly	0.3130
S-RTdelayed	0.3085

© 2024 Turri G et al. *JAMA Network Open*.

eAppendix 14. Rate of ypN0

Characteristics of the network

Number of treatments:

9

Number of studies:

21

Number of individuals included:

10070

Number of individuals randomized to each treatment:

	Treatment name	N. individuals randomized
1	CHT	885
2	Induction + L-CRT	469
3	L-CRT + consolidation	288
4	L-CRT1	4498
5	L-CRT2	1732
6	L-RT	872
7	S-RT + consolidation	1090
8	S-RTdelayed	75
9	S-RTearly	161

Pairwise meta-analysis
Risk ratios above 1 favor the first treatment of the comparison.

© 2024 Turri G et al. *JAMA Network Open*.

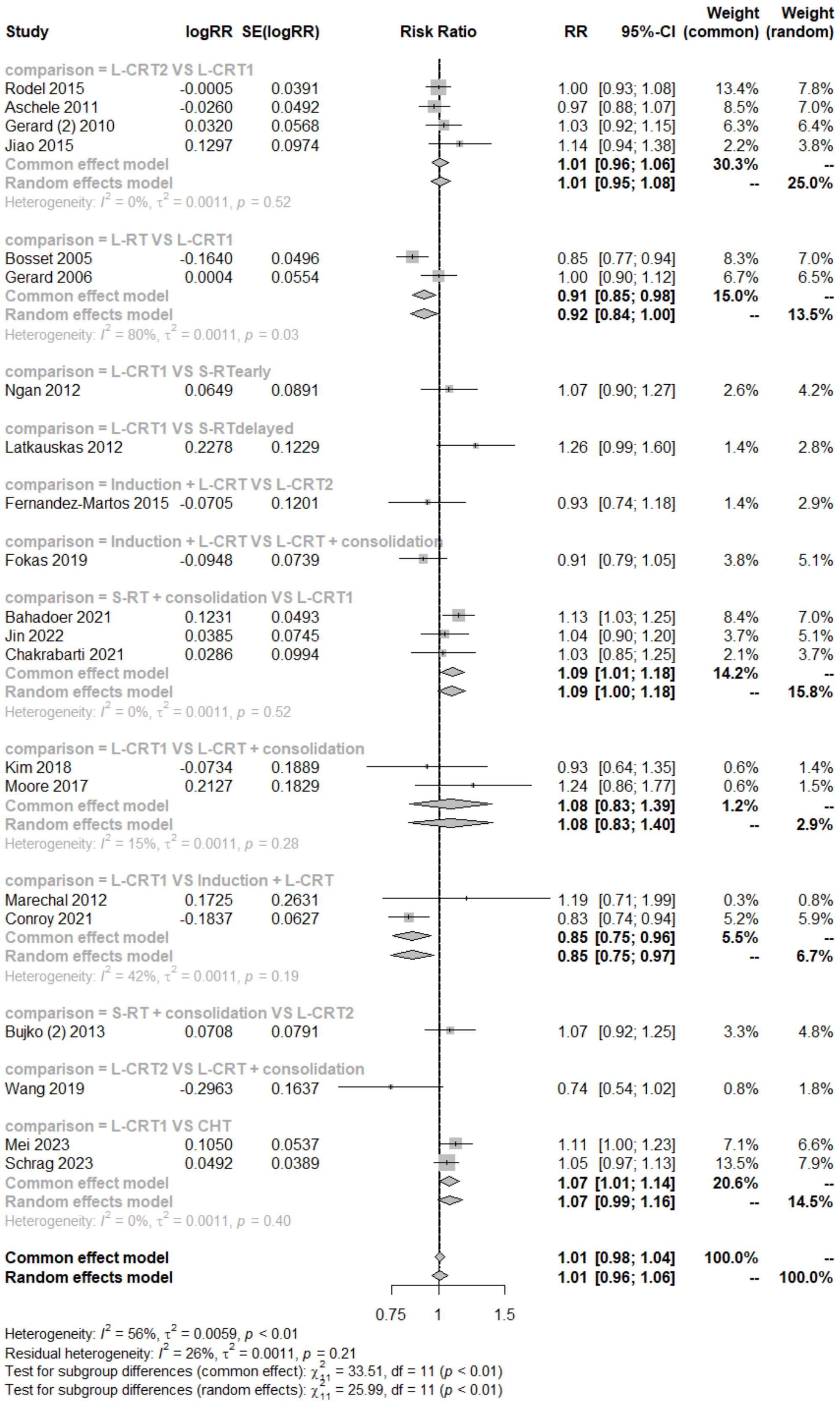

Network map The thickness of lines is proportional to the number of studies comparing the two treatments and the size of circles is proportional to the number of individuals for each treatment.

© 2024 Turri G et al. *JAMA Network Open*.

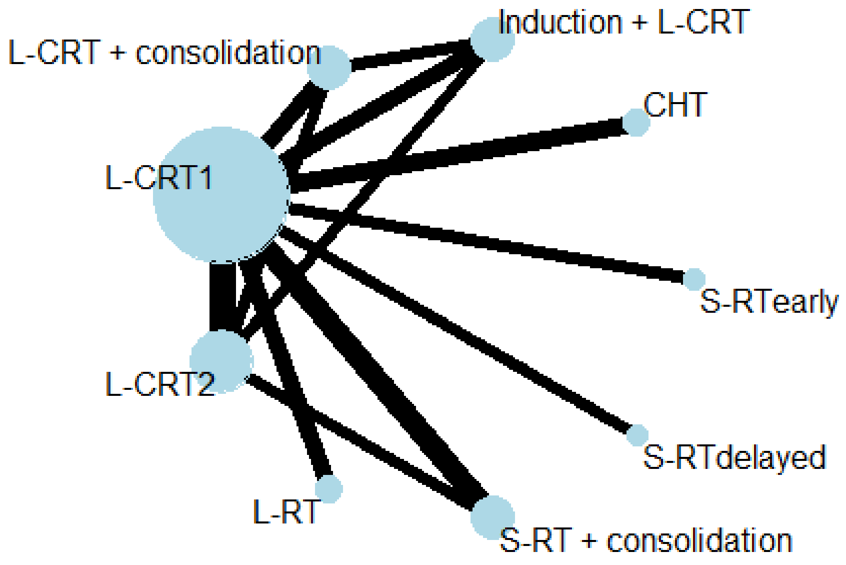

Netleague table Relative risks (RRs) and 95% confidence intervals (CIs) are reported. Results of the network meta-analysis are reported in the lower left part of the table and results from the pairwise meta-analysis are reported in the upper right part of the table. RRs higher than 1 favor the column-defining treatment.

	V1	V2	V3	V4	V5	V6	V7	V8	V9
1	CHT	.	.	0.93 (0.85 to 1.02)	.	.	.	.	.
2	0.86 (0.74 to 1.00)	Induction + L-CRT	0.91 (0.77 to 1.08)	1.17 (1.01 to 1.35)	0.93 (0.72 to 1.20)	.	.	.	.
3	0.81 (0.68 to 0.97)	0.95 (0.82 to 1.09)	L-CRT + consolidation	0.93 (0.71 to 1.21)	1.34 (0.96 to 1.88)	.	.	.	.
4	0.93 (0.85 to 1.02)	1.08 (0.96 to 1.22)	1.14 (0.98 to 1.33)	L-CRT1	0.99 (0.92 to 1.06)	1.09 (0.99 to 1.20)	0.92 (0.84 to 1.02)	1.26 (0.97 to 1.63)	1.07 (0.88 to 1.30)
5	0.91 (0.82 to 1.02)	1.07 (0.94 to 1.21)	1.13 (0.96 to 1.31)	0.98 (0.92 to 1.05)	L-CRT2	.	0.93 (0.78 to 1.12)	.	.
6	1.01 (0.89 to 1.16)	1.18 (1.01 to 1.38)	1.25 (1.04 to 1.49)	1.09 (0.99 to 1.20)	1.11 (0.99 to 1.25)	L-RT	.	.	.
7	0.86 (0.76 to 0.97)	1.00 (0.87 to 1.15)	1.06 (0.89 to 1.25)	0.92 (0.85 to 1.00)	0.94 (0.85 to 1.03)	0.85 (0.74 to 0.96)	S-RT + consolidation	.	.
8	1.17 (0.89 to 1.54)	1.36 (1.02 to 1.81)	1.44 (1.07 to 1.94)	1.26 (0.97 to 1.63)	1.28 (0.98 to 1.67)	1.15 (0.87 to 1.52)	1.36 (1.04 to 1.79)	S-RTdelayed	.
9	0.99 (0.80 to 1.23)	1.16 (0.92 to 1.45)	1.22 (0.95 to 1.57)	1.07 (0.88 to 1.30)	1.08 (0.88 to 1.34)	0.98 (0.78 to 1.22)	1.16 (0.93 to 1.44)	0.85 (0.61 to 1.18)	S-RTearly

Forest plot L-CRT-1 was used as a common comparator. RRs above 1 favor the strategy over the common comparator (L-CRT1).

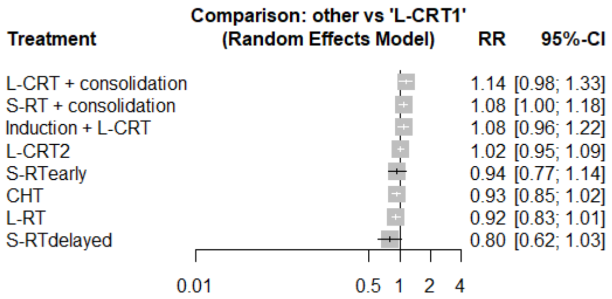

Assessment of heterogeneity and consistency

Global heterogeneity

We interpreted tau^2 as follows: heterogeneity low with tau^2≤0.010, moderate with 0.010<tau^2≤0.242, high with tau^2>0.242, and I^2 statistics as follows: not important (0%-40%), moderate (30%-60%), substantial (50%-90%), considerable (75%-100%).

© 2024 Turri G et al. *JAMA Network Open*.

tau^2= 0.0023 ; tau= 0.0475

I^2= 32.23 % (0 % to 64.22 %)

Consistency: global approach

Q statistic to assess consistency under the assumption of a full design-by-treatment interaction random effects model

Q statistics to assess homogeneity / consistency

	Q	df	p-value
Total	19.18	13	0.1176
Within designs	12.09	9	0.2081
Between designs	7.09	4	0.1312

Design-specific decomposition of within-designs Q statistic

Design	Q	df	p-value
L-CRT1:CHT	0.71	1	0.3996
L-CRT1:Induction + L-CRT	1.73	1	0.1878
L-CRT1:L-CRT + consolidation	1.18	1	0.2767
L-CRT1:L-CRT2	2.26	3	0.5195
L-CRT1:L-RT	4.90	1	0.0269
L-CRT1:S-RT + consolidation	1.31	2	0.5204

Between-designs Q statistic after detaching of single designs

Detached design	Q	df	p-value
Induction + L-CRT:L-CRT + consolidation	6.08	3	0.1076
Induction + L-CRT:L-CRT2	5.00	3	0.1719
L-CRT + consolidation:L-CRT2	5.97	3	0.1130
L-CRT1:Induction + L-CRT	3.30	3	0.3480
L-CRT1:L-CRT + consolidation	3.00	3	0.3915
L-CRT1:L-CRT2	6.92	3	0.0744
L-CRT1:S-RT + consolidation	7.09	3	0.0692
L-CRT2:S-RT + consolidation	7.09	3	0.0692

Q statistic to assess consistency under the assumption of a full design-by-treatment interaction random effects model

	Q	df	p-value	tau.within	tau2.within
Between designs	6.19	4	0.1855	0.0369	0.0014

Consistency: local approach

Separate indirect from direct evidence (SIDE) using back-calculation method

Random effects model:

comparison	k	prop	nma	direct	indir.	RoR	z	p-value
Induction + L-CRT:L-CRT + consolidation	1	0.67	0.95	0.91	1.03	0.89	-0.79	0.4280
L-CRT1:Induction + L-CRT	2	0.62	0.92	0.86	1.05	0.82	-1.63	0.1034
Induction + L-CRT:L-CRT2	1	0.24	1.07	0.93	1.11	0.84	-1.19	0.2336
L-CRT1:L-CRT + consolidation	2	0.32	0.87	1.08	0.79	1.36	1.87	0.0620
L-CRT + consolidation:L-CRT2	1	0.22	1.13	1.34	1.07	1.26	1.18	0.2373
L-CRT1:L-CRT2	4	0.82	0.98	0.99	0.98	1.01	0.10	0.9209
L-CRT1:S-RT + consolidation	3	0.80	0.92	0.92	0.92	1.01	0.08	0.9337
L-CRT2:S-RT + consolidation	1	0.30	0.94	0.93	0.94	0.99	-0.08	0.9337

Legend:

comparison - Treatment comparison

k - Number of studies providing direct evidence

© 2024 Turri G et al. *JAMA Network Open*.

prop	- Direct evidence proportion
nma	- Estimated treatment effect (RR) in network meta-analysis
direct	- Estimated treatment effect (RR) derived from direct evidence
indir.	- Estimated treatment effect (RR) derived from indirect evidence
RoR	- Ratio of Ratios (direct versus indirect)
z	- z-value of test for disagreement (direct versus indirect)
p-value	- p-value of test for disagreement (direct versus indirect)

Netrank Higher p-score values indicate higher ranking of treatments

	P-score
L-CRT + consolidation	0.9141
S-RT + consolidation	0.8155
Induction + L-CRT	0.7881
L-CRT2	0.5824
L-CRT1	0.5085
S-RTearly	0.3354
CHT	0.2597
L-RT	0.2247
S-RTdelayed	0.0715

© 2024 Turri G et al. *JAMA Network Open*.

eAppendix 15. Rate of post-operative complications Clavien-Dindo III or Greater

Characteristics of the network

Number of treatments:

```
6
```

Number of studies:

```
9
```

Number of individuals included:

```
3525
```

Number of individuals randomized to each treatment:

	Treatment name	N. individuals randomized
1	Induction + L-CRT	356
2	L-CRT + consolidation	212
3	L-CRT1	1629
4	L-CRT2	883
5	S-RT + consolidation	295
6	S-RTearly	150

Pairwise meta-analysis

Risk ratios above 1 favor the first treatment of the comparison.

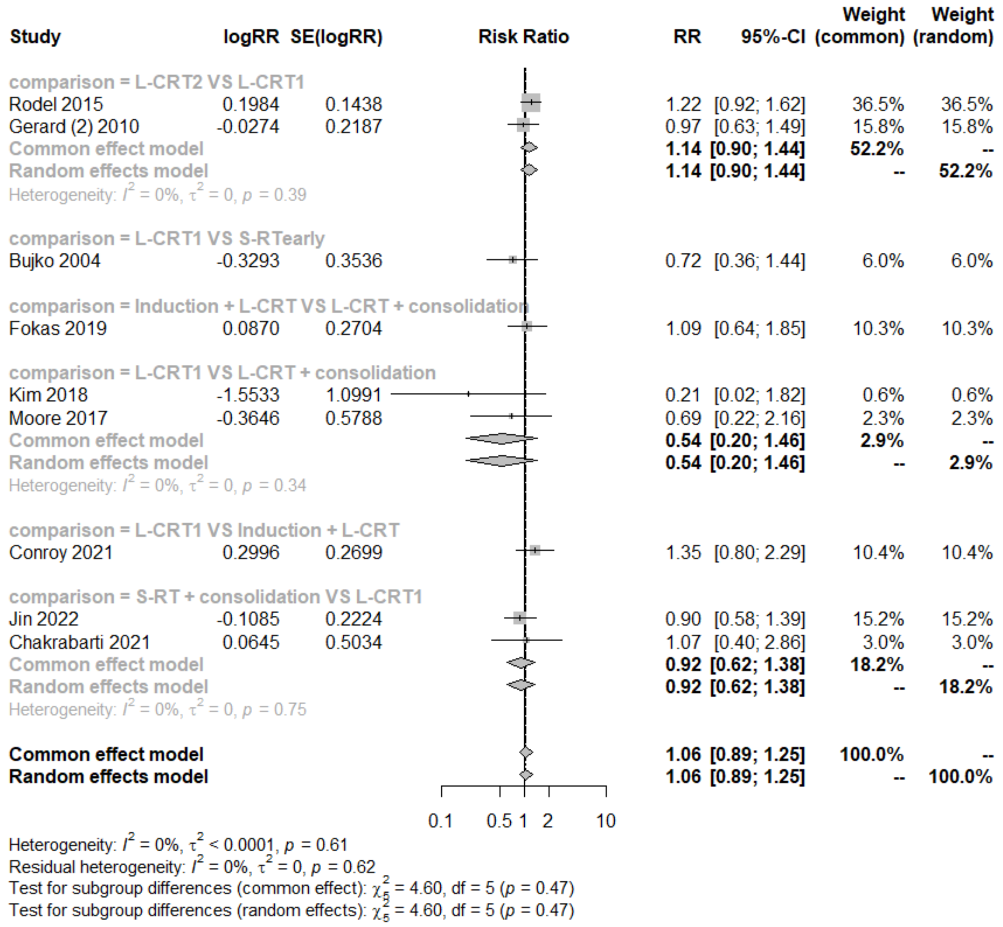

© 2024 Turri G et al. *JAMA Network Open*.

Network map The thickness of lines is proportional to the number of studies comparing the two treatments and the size of circles is proportional to the number of individuals for each treatment.

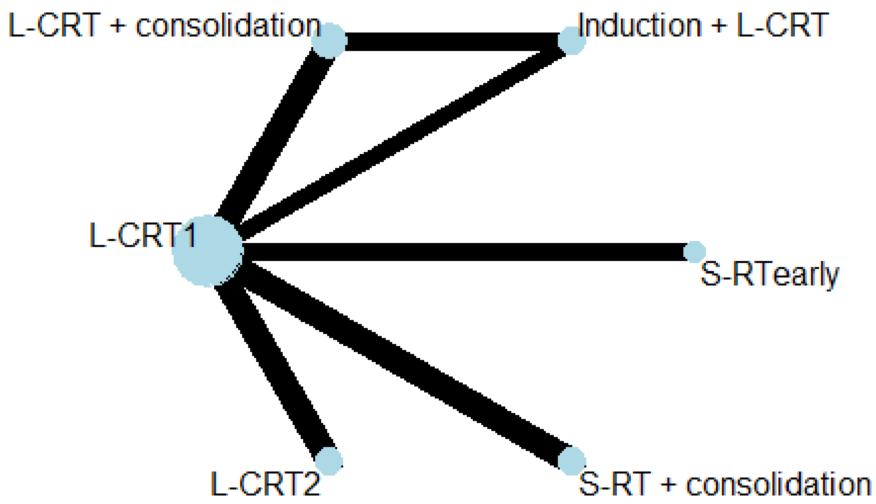

Netleague table Relative risks (RRs) and 95% confidence intervals (CIs) are reported. Results of the network meta-analysis are reported in the lower left part of the table and results from the pairwise meta-analysis are reported in the upper right part of the table. RRs higher than 1 favor the column-defining treatment.

	V1	V2	V3	V4	V5	V6
1	Induction + L-CRT	1.09 (0.63 to 1.89)	0.74 (0.43 to 1.28)	.	.	.
2	0.90 (0.55 to 1.48)	L-CRT + consolidation	1.87 (0.68 to 5.14)	.	.	.
3	0.89 (0.54 to 1.47)	0.99 (0.53 to 1.83)	L-CRT1	0.88 (0.68 to 1.15)	1.08 (0.71 to 1.64)	0.72 (0.35 to 1.46)
4	0.79 (0.45 to 1.38)	0.87 (0.45 to 1.71)	0.88 (0.68 to 1.15)	L-CRT2	.	.
5	0.97 (0.51 to 1.85)	1.07 (0.51 to 2.25)	1.08 (0.71 to 1.64)	1.22 (0.75 to 2.00)	S-RT + consolidation	.
6	0.64 (0.27 to 1.53)	0.71 (0.28 to 1.82)	0.72 (0.35 to 1.46)	0.81 (0.38 to 1.73)	0.67 (0.29 to 1.52)	S-RTearly

Forest plot L-CRT-1 was used as a common comparator. RRs above 1 favor the strategy over the common comparator (L-CRT1).

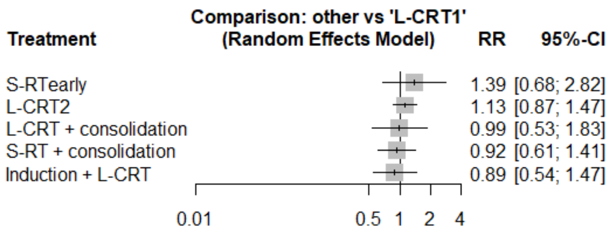

Assessment of heterogeneity and consistency

Global heterogeneity

© 2024 Turri G et al. *JAMA Network Open*.

We interpreted tau^2 as follows: heterogeneity low with tau^2≤0.010, moderate with 0.010<tau^2≤0.242, high with tau^2>0.242, and I^2 statistics as follows: not important (0%-40%), moderate (30%-60%), substantial (50%-90%), considerable (75%-100%).

```
tau^2= 0.0058 ; tau= 0.0764

I^2= 5.98 % ( 0 % to 80.45 %)
```

Consistency: global approach

Q statistic to assess consistency under the assumption of a full design-by-treatment interaction random effects model

```
Q statistics to assess homogeneity / consistency

                    Q df p-value
Total            4.25  4  0.3727
Within designs   1.76  3  0.6239
Between designs  2.50  1  0.1142

Design-specific decomposition of within-designs Q statistic

                           Design    Q df p-value
L-CRT1:L-CRT + consolidation 0.92  1  0.3386
              L-CRT1:L-CRT2 0.74  1  0.3882
 L-CRT1:S-RT + consolidation 0.10  1  0.7532

Between-designs Q statistic after detaching of single designs

                      Detached design    Q df p-value
Induction + L-CRT:L-CRT + consolidation 0.00  0      --
            L-CRT1:Induction + L-CRT 0.00  0      --
        L-CRT1:L-CRT + consolidation 0.00  0      --

Q statistic to assess consistency under the assumption of a full design-by-treatment interaction random effects model

                   Q df p-value tau.within tau2.within
Between designs 2.50  1  0.1142  0                   0
```

Consistency: local approach

```
Separate indirect from direct evidence (SIDE) using back-calculation method

Random effects model:

                             comparison k prop  nma direct indir.  RoR     z p-value
Induction + L-CRT:L-CRT + consolidation 1 0.81 0.90   1.09   0.40 2.75  1.55  0.1201
               L-CRT1:Induction + L-CRT 1 0.81 1.12   1.35   0.49 2.75  1.55  0.1201
           L-CRT1:L-CRT + consolidation 2 0.37 1.01   0.54   1.47 0.36 -1.55  0.1201

Legend:
 comparison - Treatment comparison
 k          - Number of studies providing direct evidence
 prop       - Direct evidence proportion
 nma        - Estimated treatment effect (RR) in network meta-analysis
 direct     - Estimated treatment effect (RR) derived from direct evidence
 indir.     - Estimated treatment effect (RR) derived from indirect evidence
 RoR        - Ratio of Ratios (direct versus indirect)
 z          - z-value of test for disagreement (direct versus indirect)
 p-value    - p-value of test for disagreement (direct versus indirect)
```

Netrank Higher p-score values indicate higher ranking of treatments

© 2024 Turri G et al. *JAMA Network Open*.

	P-score
S-RTearly	0.7915
L-CRT2	0.6713
L-CRT + consolidation	0.4601
L-CRT1	0.4368
S-RT + consolidation	0.3408
Induction + L-CRT	0.2994

© 2024 Turri G et al. *JAMA Network Open*.

eAppendix 16. Rate of anastomotic leak

Characteristics of the network

Number of treatments:

```
9
```

Number of studies:

```
17
```

Number of individuals included:

```
8333
```

Number of individuals randomized to each treatment:

	Treatment name	N. individuals randomized
1	CHT	424
2	Induction + L-CRT	294
3	L-CRT + consolidation	126
4	L-CRT1	3752
5	L-CRT2	2481
6	L-RT	360
7	S-RT + consolidation	670
8	S-RTdelayed	68
9	S-RTearly	158

Pairwise meta-analysis
Risk ratios above 1 favor the first treatment of the comparison.

© 2024 Turri G et al. *JAMA Network Open*.

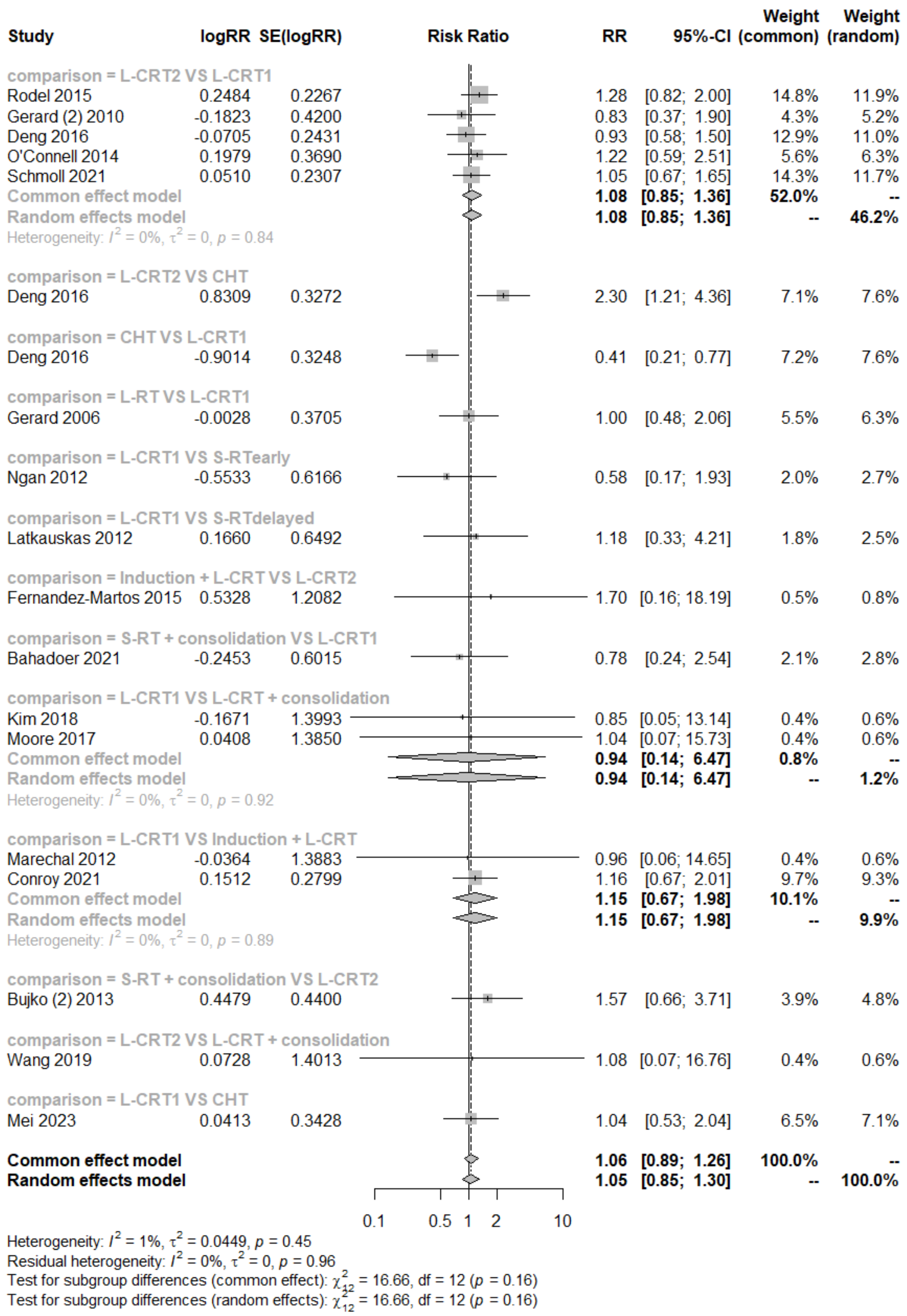

Network map The thickness of lines is proportional to the number of studies comparing the two treatments and the size of circles is proportional to the number of individuals for each treatment.

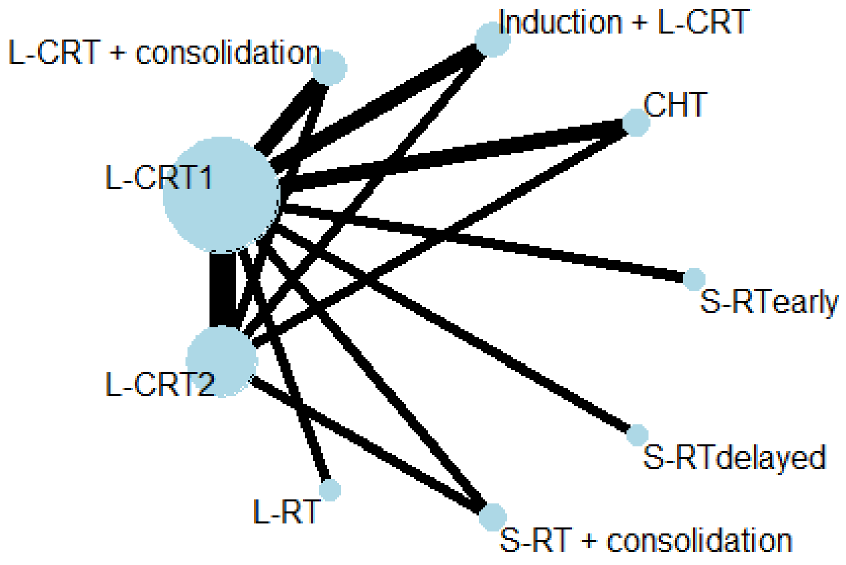

Netleague table Relative risks (RRs) and 95% confidence intervals (CIs) are reported. Results of the network meta-analysis are reported in the lower left part of the table and results from the pairwise meta-analysis are reported in the upper right part of the table. RRs higher than 1 favor the column-defining treatment.

	V1	V2	V3	V4	V5	V6	V7	V8	V9
1	CHT	.	.	0.61 (0.38 to 0.97)	0.44 (0.23 to 0.83)	.	.	.	.
2	0.69 (0.34 to 1.36)	Induction + L-CRT	.	0.87 (0.51 to 1.48)	1.70 (0.16 to 18.19)	.	.	.	.
3	0.59 (0.11 to 3.04)	0.86 (0.16 to 4.55)	L-CRT + consolidation	1.06 (0.15 to 7.33)	0.93 (0.06 to 14.49)	.	.	.	.
4	0.62 (0.39 to 0.96)	0.90 (0.53 to 1.52)	1.04 (0.21 to 5.07)	L-CRT1	0.93 (0.73 to 1.18)	1.00 (0.49 to 2.07)	1.28 (0.39 to 4.15)	1.18 (0.33 to 4.21)	0.58 (0.17 to 1.93)
5	0.57 (0.36 to 0.92)	0.83 (0.47 to 1.47)	0.97 (0.20 to 4.73)	0.93 (0.74 to 1.17)	L-CRT2	.	0.64 (0.27 to 1.51)	.	.
6	0.62 (0.26 to 1.45)	0.90 (0.37 to 2.21)	1.05 (0.18 to 5.95)	1.00 (0.49 to 2.07)	1.08 (0.50 to 2.31)	L-RT	.	.	.
7	0.48 (0.21 to 1.10)	0.70 (0.29 to 1.68)	0.81 (0.14 to 4.55)	0.78 (0.38 to 1.58)	0.83 (0.41 to 1.68)	0.77 (0.28 to 2.14)	S-RT + consolidation	.	.
8	0.73 (0.19 to 2.80)	1.06 (0.27 to 4.20)	1.23 (0.16 to 9.36)	1.18 (0.33 to 4.21)	1.27 (0.35 to 4.63)	1.18 (0.27 to 5.09)	1.52 (0.35 to 6.54)	S-RTdelayed	.
9	0.35 (0.10 to 1.28)	0.52 (0.14 to 1.93)	0.60 (0.08 to 4.38)	0.58 (0.17 to 1.93)	0.62 (0.18 to 2.12)	0.57 (0.14 to 2.35)	0.74 (0.18 to 3.01)	0.49 (0.08 to 2.82)	S-RTearly

Forest plot L-CRT-1 was used as a common comparator. RRs above 1 favor the strategy over the common comparator (L-CRT1).

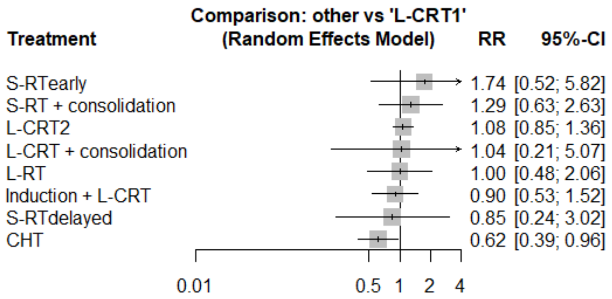

Assessment of heterogeneity and consistency

Global heterogeneity

We interpreted tau^2 as follows: heterogeneity low with tau^2≤0.010, moderate with 0.010<tau^2≤0.242, high with tau^2>0.242, and I^2 statistics as follows: not important (0%-40%), moderate (30%-60%), substantial (50%-90%), considerable (75%-100%).

© 2024 Turri G et al. *JAMA Network Open*.

tau^2= 0 ; tau= 0

I^2= 0 % (0 % to 60.23 %)

Consistency: global approach

Q statistic to assess consistency under the assumption of a full design-by-treatment interaction random effects model

Q statistics to assess homogeneity / consistency

	Q	df	p-value
Total	5.88	10	0.8251
Within designs	1.00	5	0.9624
Between designs	4.88	5	0.4308

Design-specific decomposition of within-designs Q statistic

Design	Q	df	p-value
L-CRT1:Induction + L-CRT	0.02	1	0.8947
L-CRT1:L-CRT + consolidation	0.01	1	0.9159
L-CRT1:L-CRT2	0.97	3	0.8077

Between-designs Q statistic after detaching of single designs

Detached design	Q	df	p-value
Induction + L-CRT:L-CRT2	4.51	4	0.3414
L-CRT + consolidation:L-CRT2	4.88	4	0.3000
L-CRT1:CHT	1.85	4	0.7641
L-CRT1:Induction + L-CRT	4.51	4	0.3414
L-CRT1:L-CRT + consolidation	4.88	4	0.3000
L-CRT1:L-CRT2	4.48	4	0.3454
L-CRT1:S-RT + consolidation	3.79	4	0.4346
L-CRT2:S-RT + consolidation	3.79	4	0.4346
L-CRT1:CHT:L-CRT2	1.54	3	0.6739

Q statistic to assess consistency under the assumption of a full design-by-treatment interaction random effects model

	Q	df	p-value	tau.within	tau2.within
Between designs	4.88	5	0.4308	0	0

Consistency: local approach

Separate indirect from direct evidence (SIDE) using back-calculation method

Random effects model:

comparison	k	prop	nma	direct	indir.	RoR	z	p-value
L-CRT1:CHT	2	0.94	1.62	1.64	1.39	1.18	0.16	0.8712
CHT:L-CRT2	1	0.55	0.57	0.44	0.80	0.55	-1.24	0.2159
L-CRT1:Induction + L-CRT	2	0.95	1.11	1.15	0.54	2.13	0.61	0.5431
Induction + L-CRT:L-CRT2	1	0.06	0.83	1.70	0.80	2.13	0.61	0.5431
L-CRT1:L-CRT + consolidation	2	0.67	0.96	0.94	1.00	0.94	-0.04	0.9714
L-CRT + consolidation:L-CRT2	1	0.33	0.97	0.93	0.99	0.94	-0.04	0.9714
L-CRT1:L-CRT2	5	0.95	0.93	0.93	0.93	1.00	-0.00	0.9966
L-CRT1:S-RT + consolidation	1	0.36	0.78	1.28	0.58	2.20	1.04	0.2975
L-CRT2:S-RT + consolidation	1	0.66	0.83	0.64	1.40	0.46	-1.04	0.2975

Legend:
comparison - Treatment comparison
k - Number of studies providing direct evidence
prop - Direct evidence proportion

© 2024 Turri G et al. *JAMA Network Open*.

nma	- Estimated treatment effect (RR) in network meta-analysis
direct	- Estimated treatment effect (RR) derived from direct evidence
indir.	- Estimated treatment effect (RR) derived from indirect evidence
RoR	- Ratio of Ratios (direct versus indirect)
z	- z-value of test for disagreement (direct versus indirect)
p-value	- p-value of test for disagreement (direct versus indirect)

Netrank Higher p-score values indicate higher ranking of treatments

	P-score
S-RTearly	0.7871
S-RT + consolidation	0.6921
L-CRT2	0.5900
L-CRT + consolidation	0.5152
L-RT	0.4965
L-CRT1	0.4893
S-RTdelayed	0.4042
Induction + L-CRT	0.4023
CHT	0.1233

© 2024 Turri G et al. *JAMA Network Open*.

eAppendix 17. Locoregional recurrence at 3 years

Characteristics of the network

Number of treatments:

7

Number of studies:

11

Number of individuals included:

5749

Number of individuals randomized to each treatment:

	Treatment name	N. individuals randomized
1	CHT	165
2	Induction + L-CRT	387
3	L-CRT + consolidation	210
4	L-CRT1	2319
5	L-CRT2	1486
6	S-RT + consolidation	1021
7	S-RTearly	161

Pairwise meta-analysis
Risk ratios above 1 favor the first treatment of the comparison.

© 2024 Turri G et al. *JAMA Network Open*.

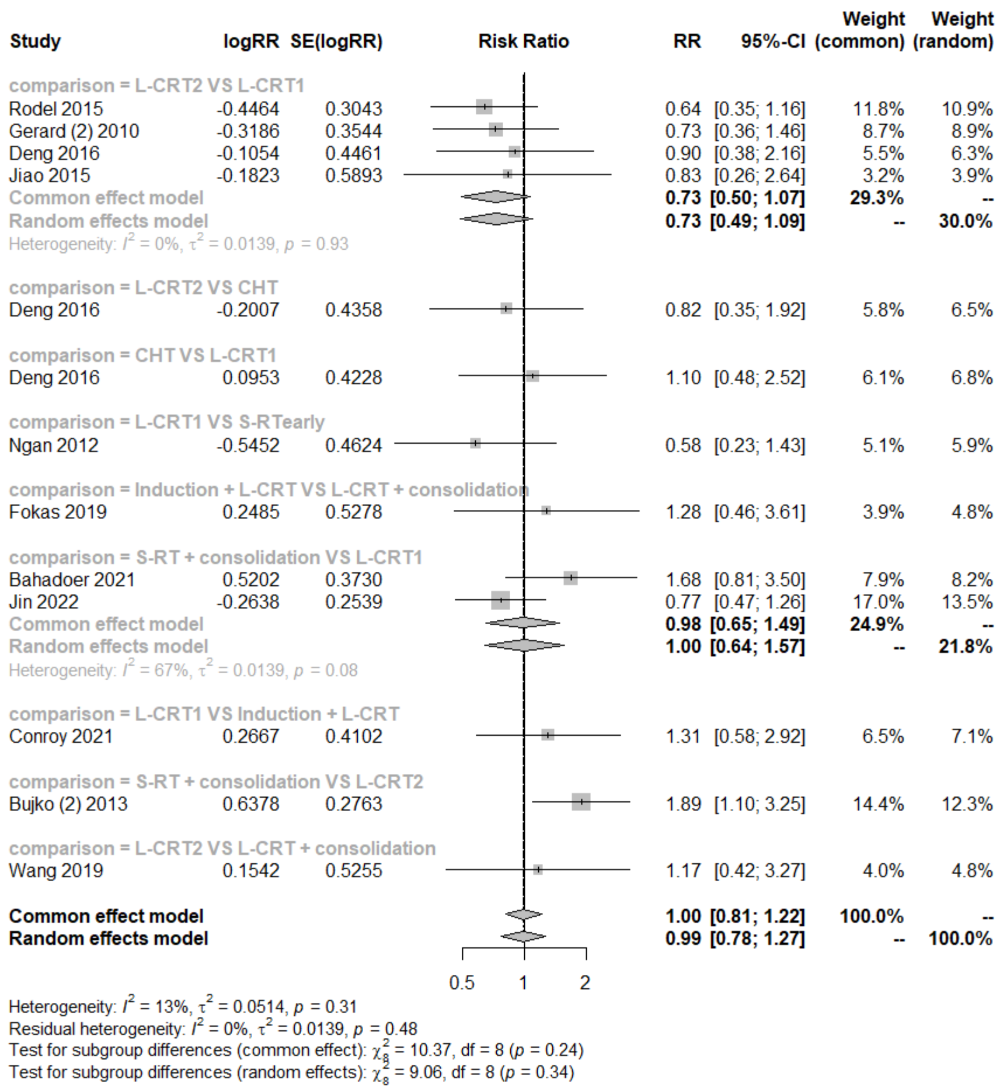

Network map The thickness of lines is proportional to the number of studies comparing the two treatments and the size of circles is proportional to the number of individuals for each treatment.

© 2024 Turri G et al. *JAMA Network Open*.

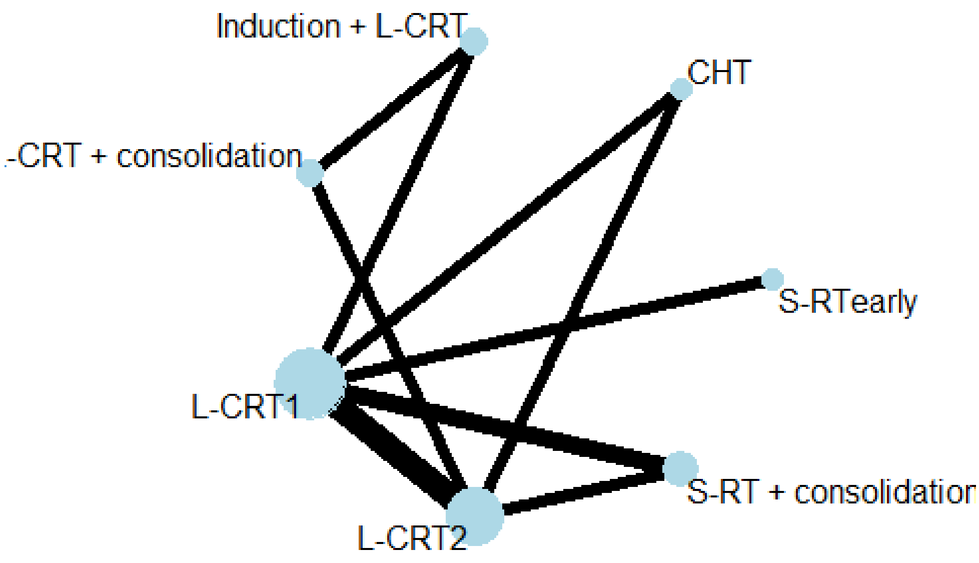

Netleague table Relative risks (RRs) and 95% confidence intervals (CIs) are reported. Results of the network meta-analysis are reported in the lower left part of the table and results from the pairwise meta-analysis are reported in the upper right part of the table. RRs higher than 1 favor the column-defining treatment.

	V1	V2	V3	V4	V5	V6	V7
1	CHT	.	.	1.10 (0.48 to 2.52)	1.22 (0.52 to 2.87)	.	.
2	1.26 (0.46 to 3.46)	Induction + L-CRT	1.28 (0.46 to 3.61)	0.77 (0.34 to 1.71)	.	.	.
3	1.64 (0.55 to 4.85)	1.30 (0.57 to 2.95)	L-CRT + consolidation	.	0.86 (0.31 to 2.40)	.	.
4	0.96 (0.46 to 2.00)	0.76 (0.37 to 1.54)	0.59 (0.25 to 1.35)	L-CRT1	1.37 (0.94 to 2.00)	1.02 (0.67 to 1.53)	0.58 (0.23 to 1.43)
5	1.42 (0.68 to 2.98)	1.13 (0.53 to 2.38)	0.87 (0.38 to 1.97)	1.48 (1.07 to 2.05)	L-CRT2	0.53 (0.31 to 0.91)	.
6	0.89 (0.40 to 1.95)	0.70 (0.32 to 1.53)	0.54 (0.22 to 1.30)	0.92 (0.65 to 1.31)	0.62 (0.42 to 0.92)	S-RT + consolidation	.
7	0.56 (0.17 to 1.79)	0.44 (0.14 to 1.39)	0.34 (0.10 to 1.16)	0.58 (0.23 to 1.43)	0.39 (0.15 to 1.02)	0.63 (0.24 to 1.66)	S-RTearly

Forest plot L-CRT-1 was used as a common comparator. RRs above 1 favor the strategy over the common comparator (L-CRT1).

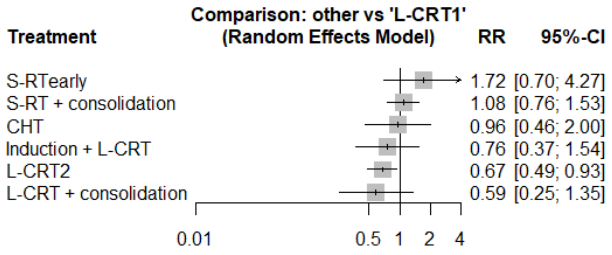

Assessment of heterogeneity and consistency

Global heterogeneity

We interpreted tau^2 as follows: heterogeneity low with tau^2≤0.010, moderate with 0.010<tau^2≤0.242, high with tau^2>0.242, and I^2 statistics as follows: not important (0%-40%), moderate (30%-60%), substantial (50%-90%), considerable (75%-100%).

© 2024 Turri G et al. *JAMA Network Open*.

tau^2= 0 ; tau= 0

I^2= 0 % (0 % to 70.81 %)

Consistency: global approach

Q statistic to assess consistency under the assumption of a full design-by-treatment interaction random effects model

Q statistics to assess homogeneity / consistency

	Q	df	p-value
Total	4.20	6	0.6495
Within designs	3.20	3	0.3613
Between designs	1.00	3	0.8018

Design-specific decomposition of within-designs Q statistic

Design	Q	df	p-value
L-CRT1:L-CRT2	0.18	2	0.9117
L-CRT1:S-RT + consolidation	3.02	1	0.0823

Between-designs Q statistic after detaching of single designs

Detached design	Q	df	p-value
Induction + L-CRT:L-CRT + consolidation	1.00	2	0.6077
L-CRT + consolidation:L-CRT2	1.00	2	0.6077
L-CRT1:Induction + L-CRT	1.00	2	0.6077
L-CRT1:L-CRT2	0.95	2	0.6221
L-CRT1:S-RT + consolidation	0.28	2	0.8711
L-CRT2:S-RT + consolidation	0.28	2	0.8711

Q statistic to assess consistency under the assumption of a full design-by-treatment interaction random effects model

	Q	df	p-value	tau.within	tau2.within
Between designs	0.88	3	0.8308	0.0945	0.0089

Consistency: local approach

Separate indirect from direct evidence (SIDE) using back-calculation method

Random effects model:

comparison	k	prop	nma	direct	indir.	RoR	z	p-value
L-CRT1:CHT	1	0.79	1.04	0.91	1.72	0.53	-0.70	0.4852
CHT:L-CRT2	1	0.75	1.42	1.22	2.24	0.55	-0.70	0.4852
Induction + L-CRT:L-CRT + consolidation	1	0.63	1.30	1.28	1.33	0.97	-0.04	0.9681
L-CRT1:Induction + L-CRT	1	0.78	1.32	1.31	1.35	0.97	-0.04	0.9681
L-CRT + consolidation:L-CRT2	1	0.63	0.87	0.86	0.89	0.97	-0.04	0.9681
L-CRT1:L-CRT2	4	0.73	1.48	1.37	1.84	0.74	-0.79	0.4299
L-CRT1:S-RT + consolidation	2	0.72	0.92	1.02	0.73	1.40	0.85	0.3956
L-CRT2:S-RT + consolidation	1	0.51	0.62	0.53	0.74	0.71	-0.85	0.3956

Legend:
comparison - Treatment comparison
k - Number of studies providing direct evidence
prop - Direct evidence proportion
nma - Estimated treatment effect (RR) in network meta-analysis
direct - Estimated treatment effect (RR) derived from direct evidence
indir. - Estimated treatment effect (RR) derived from indirect evidence
RoR - Ratio of Ratios (direct versus indirect)

© 2024 Turri G et al. *JAMA Network Open*.

z	- z-value of test for disagreement (direct versus indirect)
p-value	- p-value of test for disagreement (direct versus indirect)

Netrank Higher p-score values indicate higher ranking of treatments

	P-score
S-RTearly	0.8989
S-RT + consolidation	0.6972
L-CRT1	0.6090
CHT	0.5527
Induction + L-CRT	0.3623
L-CRT2	0.2047
L-CRT + consolidation	0.1751

© 2024 Turri G et al. *JAMA Network Open*.

eAppendix 18. Locoregional recurrence at 5 years

Characteristics of the network

Number of treatments:

```
7
```

Number of studies:

```
7
```

Number of individuals included:

```
4886
```

Number of individuals randomized to each treatment:

	Treatment name	N. individuals randomized
1	CHT	585
2	Induction + L-CRT	54
3	L-CRT1	2370
4	L-CRT2	887
5	L-RT	367
6	S-RT + consolidation	462
7	S-RTearly	161

Pairwise meta-analysis
Risk ratios above 1 favor the first treatment of the comparison.

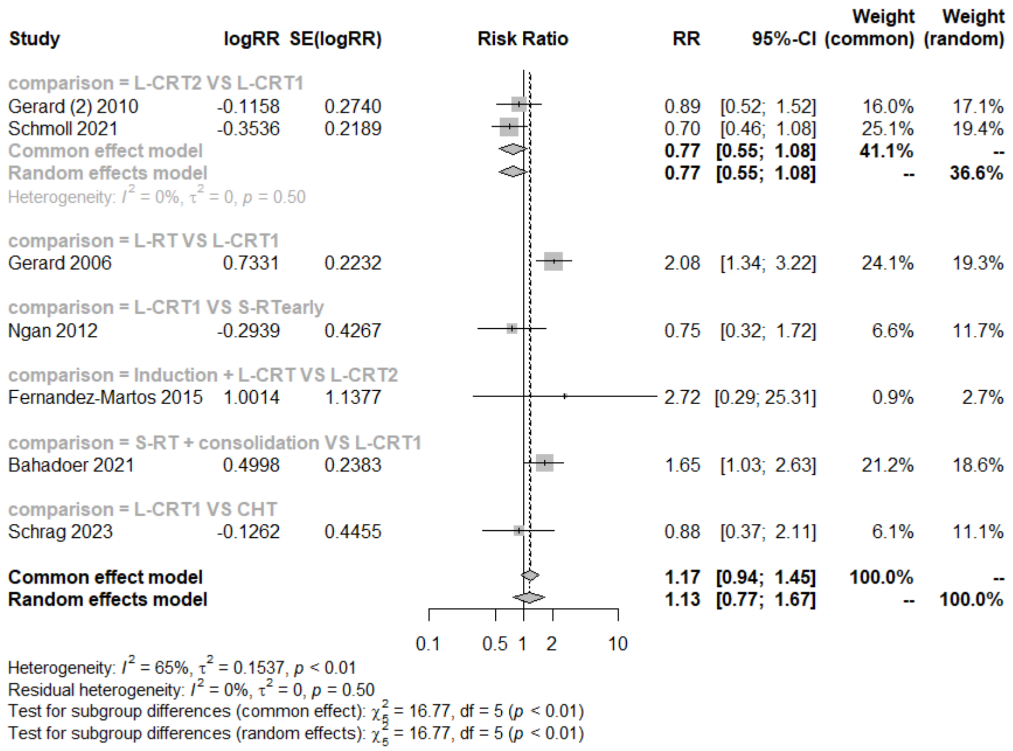

Network map The thickness of lines is proportional to the number of studies comparing the two treatments and the size of circles is proportional to the number of individuals for each treatment.

© 2024 Turri G et al. *JAMA Network Open*.

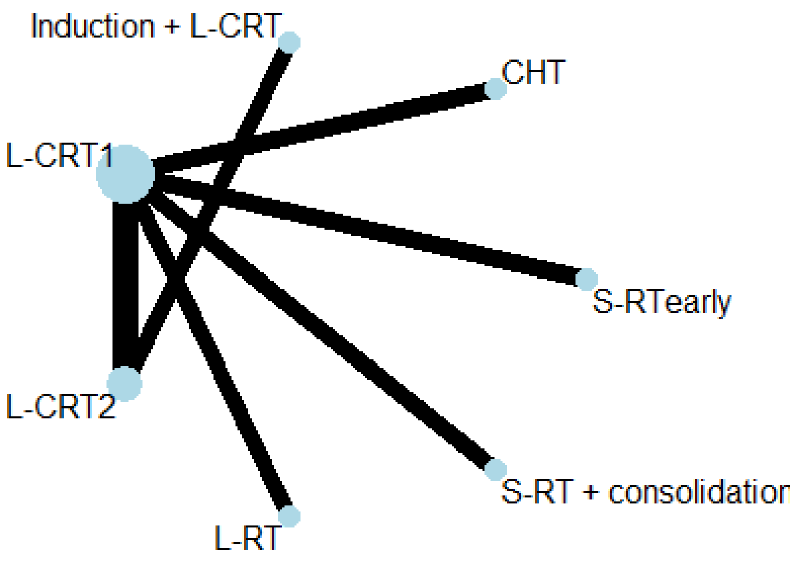

Netleague table Relative risks (RRs) and 95% confidence intervals (CIs) are reported. Results of the network meta-analysis are reported in the lower left part of the table and results from the pairwise meta-analysis are reported in the upper right part of the table. RRs higher than 1 favor the column-defining treatment.

	V1	V2	V3	V4	V5	V6	V7
1	CHT	.	1.13 (0.47 to 2.72)	.	.	.	.
2	0.54 (0.05 to 6.07)	Induction + L-CRT	.	2.72 (0.29 to 25.31)	.	.	.
3	1.13 (0.47 to 2.72)	2.10 (0.22 to 19.99)	L-CRT1	1.30 (0.93 to 1.82)	0.48 (0.31 to 0.74)	0.61 (0.38 to 0.97)	0.75 (0.32 to 1.72)
4	1.47 (0.58 to 3.75)	2.72 (0.29 to 25.31)	1.30 (0.93 to 1.82)	L-CRT2	.	.	.
5	0.55 (0.21 to 1.45)	1.01 (0.10 to 10.02)	0.48 (0.31 to 0.74)	0.37 (0.21 to 0.64)	L-RT	.	.
6	0.69 (0.26 to 1.85)	1.27 (0.13 to 12.72)	0.61 (0.38 to 0.97)	0.47 (0.26 to 0.83)	1.26 (0.67 to 2.39)	S-RT + consolidation	.
7	0.85 (0.25 to 2.83)	1.56 (0.14 to 17.32)	0.75 (0.32 to 1.72)	0.57 (0.23 to 1.41)	1.55 (0.60 to 3.99)	1.23 (0.47 to 3.20)	S-RTearly

Forest plot L-CRT-1 was used as a common comparator. RRs above 1 favor the strategy over the common comparator (L-CRT1).

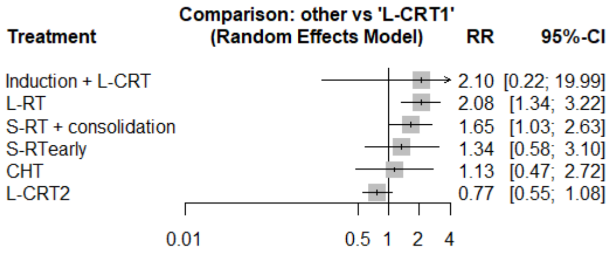

Assessment of heterogeneity and consistency

Global heterogeneity

We interpreted tau^2 as follows: heterogeneity low with tau^2≤0.010, moderate with 0.010<tau^2≤0.242, high with tau^2>0.242, and I^2 statistics as follows: not important (0%-40%), moderate (30%-60%), substantial (50%-90%), considerable (75%-100%).

© 2024 Turri G et al. *JAMA Network Open*.

tau^2= 0 ; tau= 0

I^2= 0 % (NA % to NA %)

Consistency: global approach

Q statistic to assess consistency under the assumption of a full design-by-treatment interaction random effects model

Q statistics to assess homogeneity / consistency

	Q	df	p-value
Total	0.46	1	0.4976
Within designs	0.46	1	0.4976
Between designs	0.00	0	--

Design-specific decomposition of within-designs Q statistic

Design	Q	df	p-value
L-CRT1:L-CRT2	0.46	1	0.4976

Q statistic to assess consistency under the assumption of a full design-by-treatment interaction random effects model

	Q	df	p-value	tau.within	tau2.within
Between designs	0.00	0	--	0	0

Consistency: local approach

Separate indirect from direct evidence (SIDE) using back-calculation method

Random effects model:

comparison k nma direct indir. RoR z p-value

Legend:
comparison - Treatment comparison
k - Number of studies providing direct evidence
nma - Estimated treatment effect (RR) in network meta-analysis
direct - Estimated treatment effect (RR) derived from direct evidence
indir. - Estimated treatment effect (RR) derived from indirect evidence
RoR - Ratio of Ratios (direct versus indirect)
z - z-value of test for disagreement (direct versus indirect)
p-value - p-value of test for disagreement (direct versus indirect)

Netrank Higher p-score values indicate higher ranking of treatments

	P-score
L-RT	0.8278
S-RT + consolidation	0.6778
Induction + L-CRT	0.6612
S-RTearly	0.5206
CHT	0.4077
L-CRT1	0.3081
L-CRT2	0.0967

© 2024 Turri G et al. *JAMA Network Open*.

eAppendix 19. Locoregional failure at 3 years

Characteristics of the network

Number of treatments:

7

Number of studies:

10

Number of individuals included:

5234

Number of individuals randomized to each treatment:

	Treatment name	N. individuals randomized
1	CHT	165
2	Induction + L-CRT	387
3	L-CRT + consolidation	210
4	L-CRT1	2319
5	L-CRT2	1232
6	S-RT + consolidation	760
7	S-RTearly	161

Pairwise meta-analysis
Risk ratios above 1 favor the first treatment of the comparison.

© 2024 Turri G et al. *JAMA Network Open*.

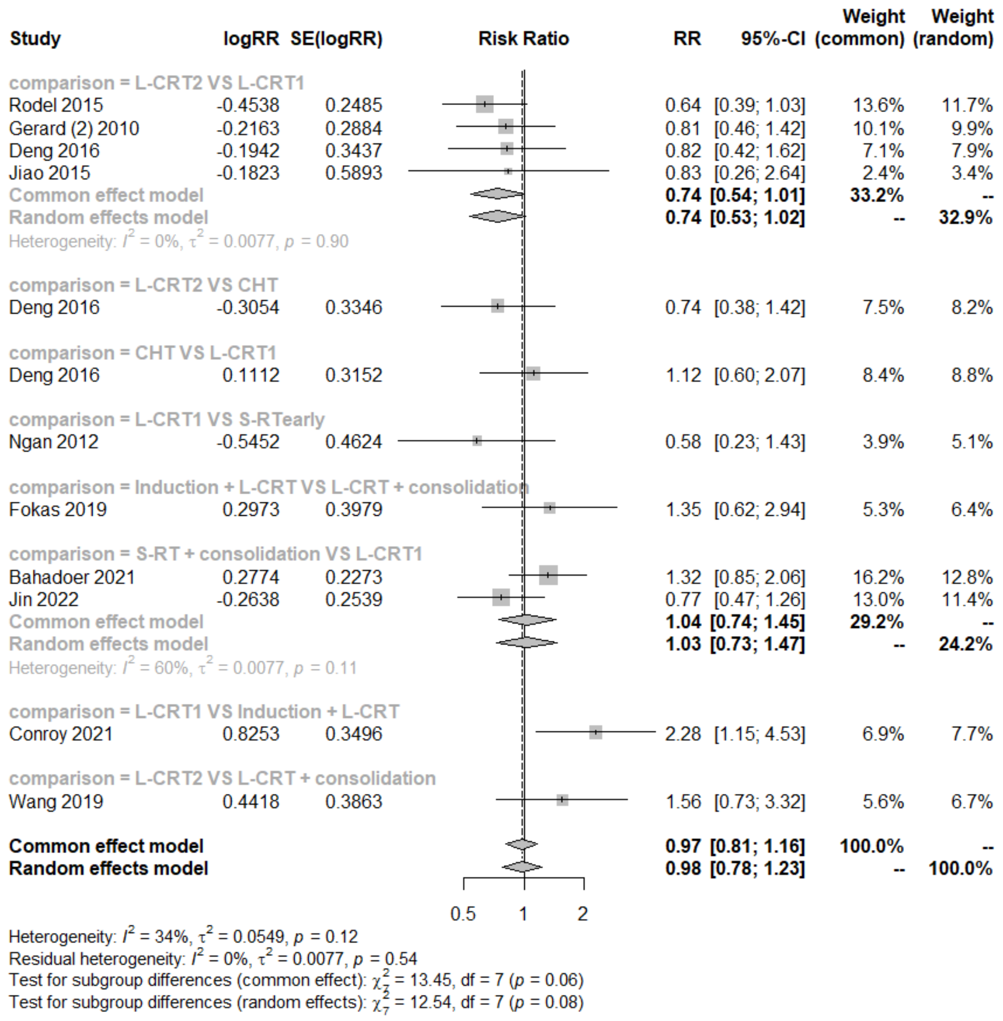

Network map The thickness of lines is proportional to the number of studies comparing the two treatments and the size of circles is proportional to the number of individuals for each treatment.

© 2024 Turri G et al. *JAMA Network Open*.

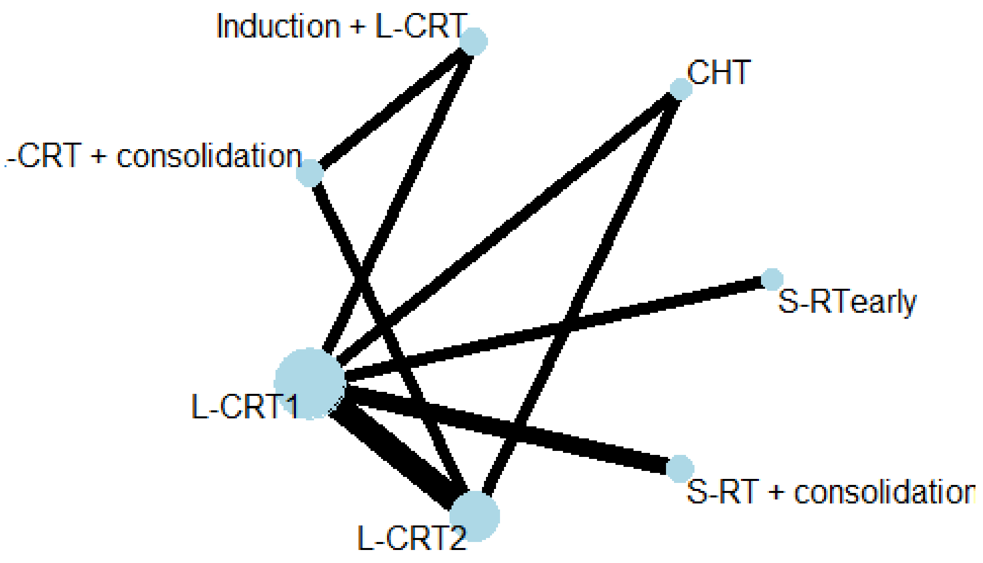

Netleague table Relative risks (RRs) and 95% confidence intervals (CIs) are reported. Results of the network meta-analysis are reported in the lower left part of the table and results from the pairwise meta-analysis are reported in the upper right part of the table. RRs higher than 1 favor the column-defining treatment.

	V1	V2	V3	V4	V5	V6	V7
1	CHT	.	.	1.12 (0.60 to 2.07)	1.36 (0.70 to 2.61)	.	.
2	2.17 (0.98 to 4.81)	Induction + L-CRT	1.35 (0.62 to 2.94)	0.44 (0.22 to 0.87)	.	.	.
3	2.57 (1.13 to 5.82)	1.18 (0.63 to 2.22)	L-CRT + consolidation	.	0.64 (0.30 to 1.37)	.	.
4	1.05 (0.60 to 1.84)	0.48 (0.27 to 0.87)	0.41 (0.22 to 0.78)	L-CRT1	1.36 (0.99 to 1.86)	0.96 (0.69 to 1.34)	0.58 (0.23 to 1.43)
5	1.46 (0.83 to 2.57)	0.67 (0.36 to 1.25)	0.57 (0.31 to 1.06)	1.39 (1.02 to 1.88)	L-CRT2	.	.
6	1.02 (0.53 to 1.94)	0.47 (0.24 to 0.92)	0.40 (0.19 to 0.81)	0.96 (0.69 to 1.34)	0.70 (0.44 to 1.09)	S-RT + consolidation	.
7	0.61 (0.21 to 1.77)	0.28 (0.10 to 0.83)	0.24 (0.08 to 0.72)	0.58 (0.23 to 1.43)	0.42 (0.16 to 1.09)	0.60 (0.23 to 1.58)	S-RTearly

Forest plot L-CRT-1 was used as a common comparator. RRs above 1 favor the strategy over the common comparator (L-CRT1).

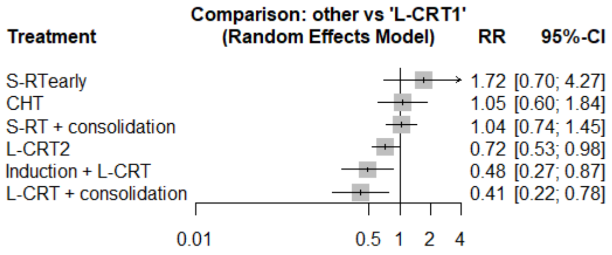

Assessment of heterogeneity and consistency

Global heterogeneity

We interpreted tau^2 as follows: heterogeneity low with tau^2≤0.010, moderate with 0.010<tau^2≤0.242, high with tau^2>0.242, and I^2 statistics as follows: not important (0%-40%), moderate (30%-60%), substantial (50%-90%), considerable (75%-100%).

© 2024 Turri G et al. *JAMA Network Open*.

```
tau^2= 0 ; tau= 0

I^2= 0 % ( 0 % to 74.62 %)
```

Consistency: global approach

Q statistic to assess consistency under the assumption of a full design-by-treatment interaction random effects model

```
Q statistics to assess homogeneity / consistency

                    Q df p-value
Total            3.43  5  0.6341
Within designs   2.99  3  0.3938
Between designs  0.44  2  0.8011

Design-specific decomposition of within-designs Q statistic

                       Design    Q df p-value
               L-CRT1:L-CRT2 0.47  2  0.7925
 L-CRT1:S-RT + consolidation 2.52  1  0.1123

Between-designs Q statistic after detaching of single designs

                      Detached design    Q df p-value
 Induction + L-CRT:L-CRT + consolidation 0.14  1  0.7129
        L-CRT + consolidation:L-CRT2 0.14  1  0.7129
            L-CRT1:Induction + L-CRT 0.14  1  0.7129
                       L-CRT1:L-CRT2 0.43  1  0.5110

Q statistic to assess consistency under the assumption of a full design-by-treatment interaction random effects model

                   Q df p-value tau.within tau2.within
Between designs 0.44  2  0.8011          0           0
```

Consistency: local approach

```
Separate indirect from direct evidence (SIDE) using back-calculation method

Random effects model:

                              comparison k prop  nma direct indir.  RoR     z p-value
                              L-CRT1:CHT 1 0.81 0.95   0.89   1.22 0.73 -0.43  0.6650
                               CHT:L-CRT2 1 0.74 1.46   1.36   1.81 0.75 -0.43  0.6650
 Induction + L-CRT:L-CRT + consolidation 1 0.65 1.18   1.35   0.93 1.45  0.56  0.5788
                L-CRT1:Induction + L-CRT 1 0.73 2.06   2.28   1.57 1.45  0.56  0.5788
            L-CRT + consolidation:L-CRT2 1 0.67 0.57   0.64   0.44 1.45  0.56  0.5788
                           L-CRT1:L-CRT2 4 0.94 1.39   1.36   1.98 0.69 -0.56  0.5788

Legend:
 comparison - Treatment comparison
 k          - Number of studies providing direct evidence
 prop       - Direct evidence proportion
 nma        - Estimated treatment effect (RR) in network meta-analysis
 direct     - Estimated treatment effect (RR) derived from direct evidence
 indir.     - Estimated treatment effect (RR) derived from indirect evidence
 RoR        - Ratio of Ratios (direct versus indirect)
 z          - z-value of test for disagreement (direct versus indirect)
 p-value    - p-value of test for disagreement (direct versus indirect)
```

Netrank Higher p-score values indicate higher ranking of treatments

© 2024 Turri G et al. *JAMA Network Open*.

	P-score
S-RTearly	0.9159
S-RT + consolidation	0.6904
CHT	0.6898
L-CRT1	0.6555
L-CRT2	0.3439
Induction + L-CRT	0.1436
L-CRT + consolidation	0.0610

© 2024 Turri G et al. *JAMA Network Open*.

eAppendix 20. Locoregional failure at 5 years

Characteristics of the network

Number of treatments:

```
7
```

Number of studies:

```
6
```

Number of individuals included:

```
3792
```

Number of individuals randomized to each treatment:

	Treatment name	N. individuals randomized
1	CHT	585
2	Induction + L-CRT	54
3	L-CRT1	1823
4	L-CRT2	340
5	L-RT	367
6	S-RT + consolidation	462
7	S-RTearly	161

Pairwise meta-analysis
Risk ratios above 1 favor the first treatment of the comparison.

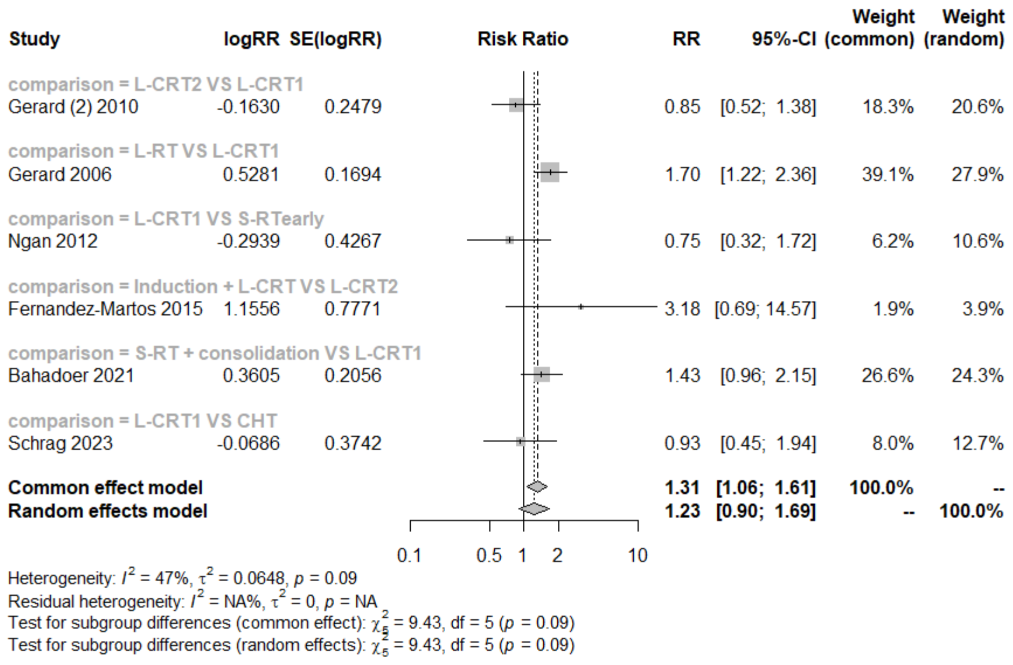

Network map The thickness of lines is proportional to the number of studies comparing the two treatments and the size of circles is proportional to the number of individuals for each treatment.

© 2024 Turri G et al. *JAMA Network Open*.

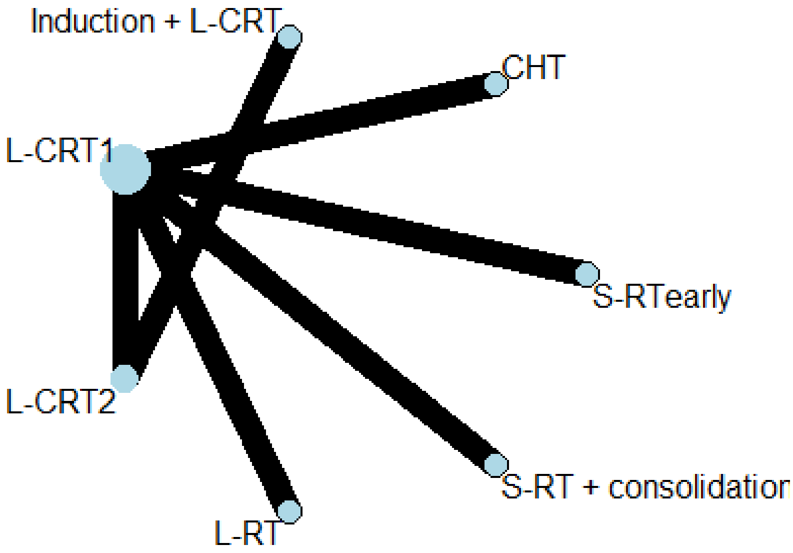

Netleague table Relative risks (RRs) and 95% confidence intervals (Cis) are reported. Results of the network meta-analysis are reported in the lower left part of the table and results from the pairwise meta-analysis are reported in the upper right part of the table. RRs higher than 1 favor the column-defining treatment.

	V1	V2	V3	V4	V5	V6	V7
1	CHT	.	1.07 (0.51 to 2.23)	.	.	.	.
2	0.40 (0.07 to 2.30)	Induction + L-CRT	.	3.18 (0.69 to 14.57)	.	.	.
3	1.07 (0.51 to 2.23)	2.70 (0.55 to 13.35)	L-CRT1	1.18 (0.72 to 1.91)	0.59 (0.42 to 0.82)	0.70 (0.47 to 1.04)	0.75 (0.32 to 1.72)
4	1.26 (0.52 to 3.04)	3.18 (0.69 to 14.57)	1.18 (0.72 to 1.91)	L-CRT2	.	.	.
5	0.63 (0.28 to 1.41)	1.59 (0.31 to 8.14)	0.59 (0.42 to 0.82)	0.50 (0.28 to 0.90)	L-RT	.	.
6	0.75 (0.32 to 1.72)	1.88 (0.36 to 9.78)	0.70 (0.47 to 1.04)	0.59 (0.32 to 1.11)	1.18 (0.70 to 1.99)	S-RT + consolidation	.
7	0.80 (0.26 to 2.43)	2.01 (0.33 to 12.22)	0.75 (0.32 to 1.72)	0.63 (0.24 to 1.67)	1.26 (0.51 to 3.11)	1.07 (0.42 to 2.70)	S-RTearly

Forest plot L-CRT-1 was used as a common comparator. RRs above 1 favor the strategy over the common comparator (L-CRT1).

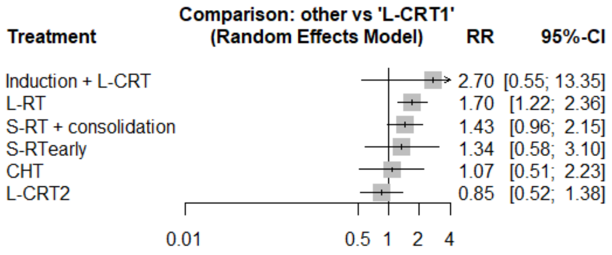

Assessment of heterogeneity and consistency

Global heterogeneity

We interpreted tau^2 as follows: heterogeneity low with tau^2≤0.010, moderate with 0.010<tau^2≤0.242, high with tau^2>0.242, and I^2 statistics as follows: not important (0%-40%), moderate (30%-60%), substantial (50%-90%), considerable (75%-100%).

© 2024 Turri G et al. *JAMA Network Open*.

Tau^2= NA ; tau= NA

I^2= NA % (NA % to NA %)

Consistency: global approach

Q statistic to assess consistency under the assumption of a full design-by-treatment interaction random effects model

Q statistics to assess homogeneity / consistency

	Q	df	p-value
Total	0.00	0	--
Within designs	0.00	0	--
Between designs	0.00	0	--

Q statistic to assess consistency under the assumption of a full design-by-treatment interaction random effects model

	Q	df	p-value	tau.within	tau2.within
Between designs	0.00	0	--	0	0

Consistency: local approach

Separate indirect from direct evidence (SIDE) using back-calculation method

Random effects model:

comparison k nma direct indir. RoR z p-value

Legend:
comparison – Treatment comparison
k - Number of studies providing direct evidence
nma - Estimated treatment effect (RR) in network meta-analysis
direct - Estimated treatment effect (RR) derived from direct evidence
indir. – Estimated treatment effect (RR) derived from indirect evidence
RoR - Ratio of Ratios (direct versus indirect)
z - z-value of test for disagreement (direct versus indirect)
p-value - p-value of test for disagreement (direct versus indirect)

Netrank Higher p-score values indicate higher ranking of treatments

	P-score
Induction + L-CRT	0.8215
L-RT	0.7627
S-RT + consolidation	0.6180
S-Rtearly	0.5341
CHT	0.3577
L-CRT1	0.2617
L-CRT2	0.1444

© 2024 Turri G et al. *JAMA Network Open*.

eAppendix 21. Distant recurrence rate at 3 years

Characteristics of the network

Number of treatments:

```
5
```

Number of studies:

```
8
```

Number of individuals included:

```
4811
```

Number of individuals randomized to each treatment:

	Treatment name	N. individuals randomized
1	Induction + L-CRT	387
2	L-CRT + consolidation	150
3	L-CRT1	1992
4	L-CRT2	1261
5	S-RT + consolidation	1021

Pairwise meta-analysis

Risk ratios above 1 favor the first treatment of the comparison.

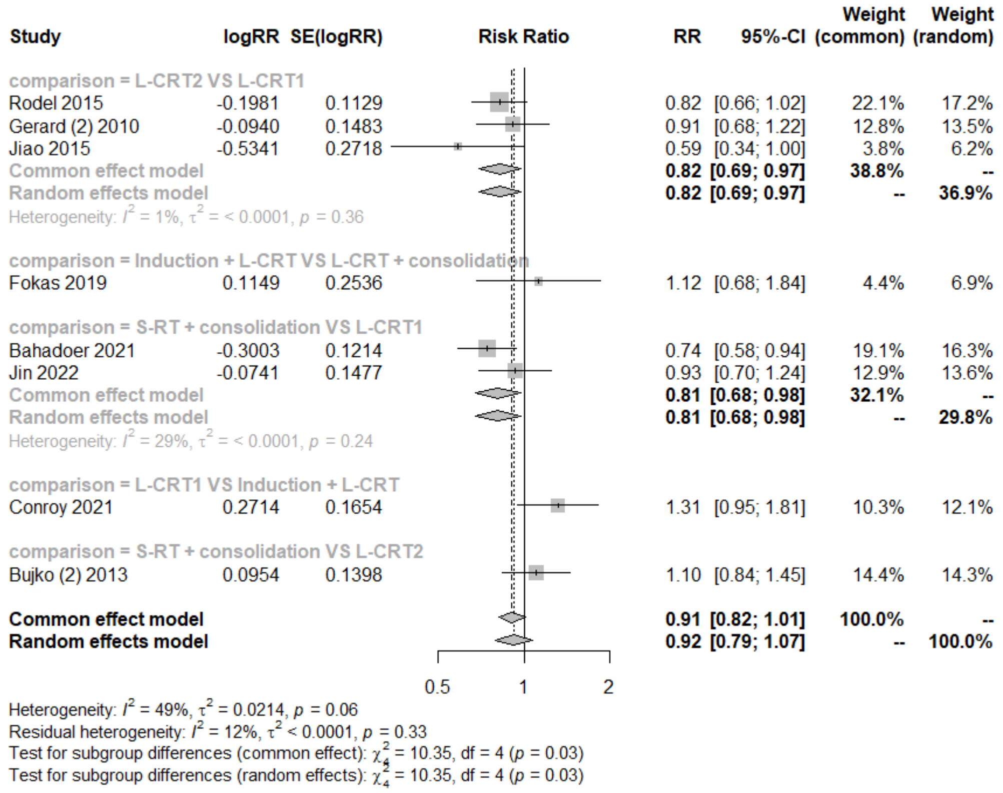

Network map The thickness of lines is proportional to the number of studies comparing the two treatments and the size of circles is proportional to the number of individuals for each treatment.

© 2024 Turri G et al. *JAMA Network Open*.

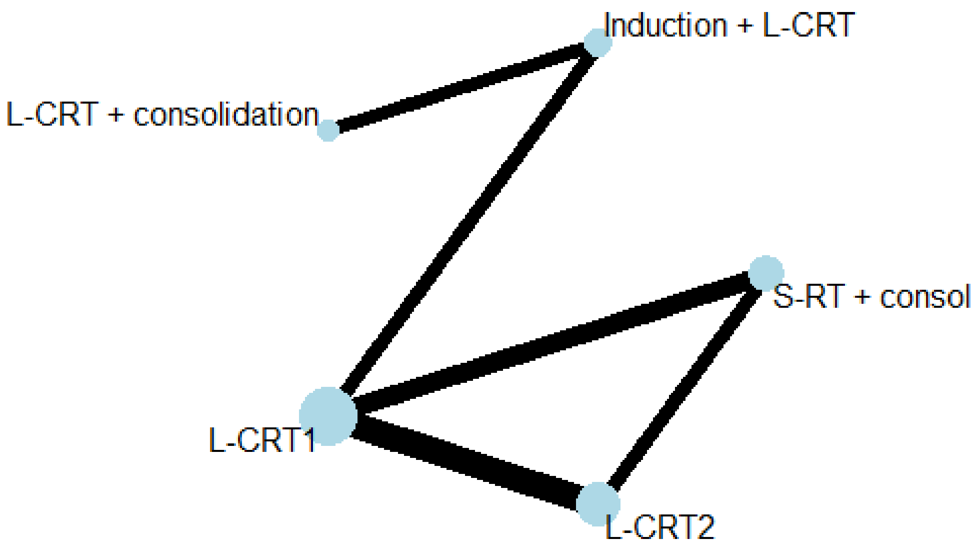

Netleague table Relative risks (RRs) and 95% confidence intervals (CIs) are reported. Results of the network meta-analysis are reported in the lower left part of the table and results from the pairwise meta-analysis are reported in the upper right part of the table. RRs higher than 1 favor the column-defining treatment.

	V1	V2	V3	V4	V5
1	Induction + L-CRT	1.12 (0.68 to 1.84)	0.76 (0.55 to 1.05)	.	.
2	1.12 (0.68 to 1.84)	L-CRT + consolidation	.	.	.
3	0.76 (0.55 to 1.05)	0.68 (0.38 to 1.23)	L-CRT1	1.22 (1.03 to 1.44)	1.23 (1.03 to 1.48)
4	0.95 (0.66 to 1.36)	0.85 (0.46 to 1.56)	1.24 (1.07 to 1.44)	L-CRT2	0.91 (0.69 to 1.20)
5	0.91 (0.64 to 1.31)	0.82 (0.44 to 1.51)	1.20 (1.02 to 1.41)	0.96 (0.80 to 1.16)	S-RT + consolidation

Forest plot L-CRT-1 was used as a common comparator. RRs above 1 favor the strategy over the common comparator (L-CRT1).

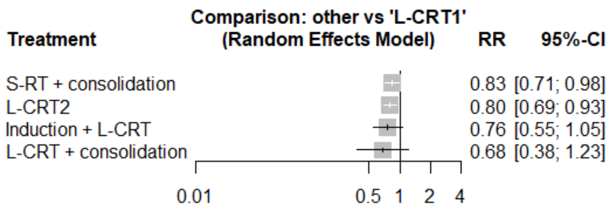

Assessment of heterogeneity and consistency

Global heterogeneity

We interpreted tau^2 as follows: heterogeneity low with tau^2≤0.010, moderate with 0.010<tau^2≤0.242, high with tau^2>0.242, and I^2 statistics as follows: not important (0%-40%), moderate (30%-60%), substantial (50%-90%), considerable (75%-100%).

© 2024 Turri G et al. *JAMA Network Open*.

tau^2= 0 ; tau= 0

I^2= 0 % (0 % to 79.2 %)

Consistency: global approach

Q statistic to assess consistency under the assumption of a full design-by-treatment interaction random effects model

Q statistics to assess homogeneity / consistency

	Q	df	p-value
Total	3.75	4	0.4415
Within designs	3.42	3	0.3313
Between designs	0.33	1	0.5680

Design-specific decomposition of within-designs Q statistic

Design	Q	df	p-value
L-CRT1:L-CRT2	2.02	2	0.3641
L-CRT1:S-RT + consolidation	1.40	1	0.2368

Between-designs Q statistic after detaching of single designs

Detached design	Q	df	p-value
L-CRT1:L-CRT2	0.00	0	--
L-CRT1:S-RT + consolidation	0.00	0	--
L-CRT2:S-RT + consolidation	0.00	0	--

Q statistic to assess consistency under the assumption of a full design-by-treatment interaction random effects model

	Q	df	p-value	tau.within	tau2.within
Between designs	0.24	1	0.6210	0.0566	0.0032

Consistency: local approach

Separate indirect from direct evidence (SIDE) using back-calculation method

Random effects model:

comparison	k	prop	nma	direct	indir.	RoR	z	p-value
L-CRT1:L-CRT2	3	0.80	1.24	1.22	1.36	0.90	-0.57	0.5680
L-CRT1:S-RT + consolidation	2	0.75	1.20	1.23	1.11	1.11	0.57	0.5680
L-CRT2:S-RT + consolidation	1	0.45	0.96	0.91	1.01	0.90	-0.57	0.5680

Legend:
comparison - Treatment comparison
k - Number of studies providing direct evidence
prop - Direct evidence proportion
nma - Estimated treatment effect (RR) in network meta-analysis
direct - Estimated treatment effect (RR) derived from direct evidence
indir. - Estimated treatment effect (RR) derived from indirect evidence
RoR - Ratio of Ratios (direct versus indirect)
z - z-value of test for disagreement (direct versus indirect)
p-value - p-value of test for disagreement (direct versus indirect)

Netrank Higher p-score values indicate higher ranking of treatments

	P-score
L-CRT1	0.9585
S-RT + consolidation	0.5226
L-CRT2	0.4174

© 2024 Turri G et al. *JAMA Network Open*.

Induction + L-CRT	0.3565
L-CRT + consolidation	0.2449

© 2024 Turri G et al. *JAMA Network Open*.

eAppendix 22. Distant recurrence rate at 5 years

Characteristics of the network

Number of treatments:

```
5
```

Number of studies:

```
5
```

Number of individuals included:

```
3016
```

Number of individuals randomized to each treatment:

	Treatment name	N. individuals randomized
1	Induction + L-CRT	54
2	L-CRT1	1452
3	L-CRT2	887
4	S-RT + consolidation	462
5	S-RTearly	161

Pairwise meta-analysis
Risk ratios above 1 favor the first treatment of the comparison.

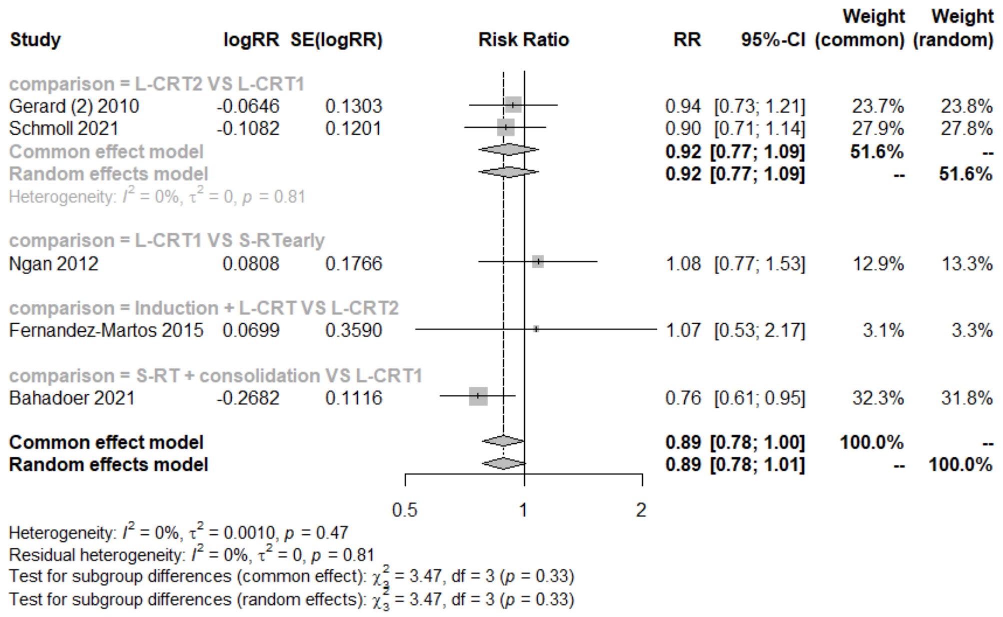

Network map The thickness of lines is proportional to the number of studies comparing the two treatments and the size of circles is proportional to the number of individuals for each treatment.

© 2024 Turri G et al. *JAMA Network Open*.

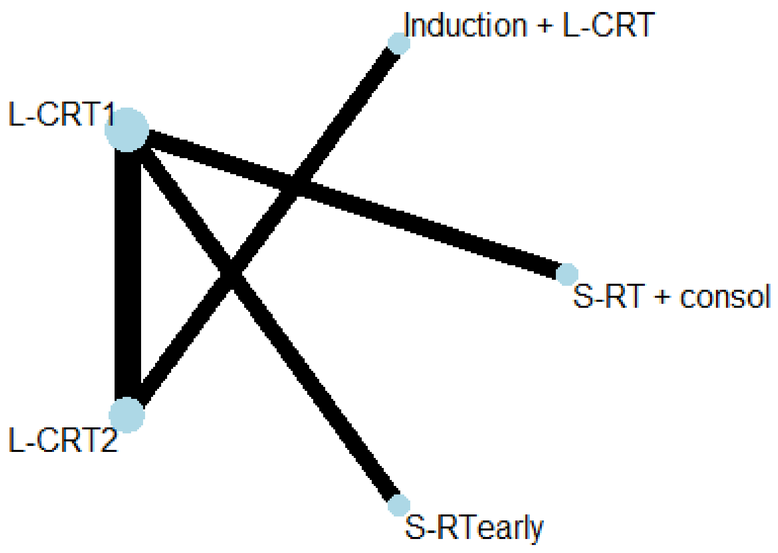

Netleague table Relative risks (RRs) and 95% confidence intervals (CIs) are reported. Results of the network meta-analysis are reported in the lower left part of the table and results from the pairwise meta-analysis are reported in the upper right part of the table. RRs higher than 1 favor the column-defining treatment.

	V1	V2	V3	V4	V5
1	Induction + L-CRT	.	1.07 (0.53 to 2.17)	.	.
2	0.98 (0.48 to 2.03)	L-CRT1	1.09 (0.92 to 1.30)	1.08 (0.77 to 1.53)	1.31 (1.05 to 1.63)
3	1.07 (0.53 to 2.17)	1.09 (0.92 to 1.30)	L-CRT2	.	.
4	1.06 (0.48 to 2.38)	1.08 (0.77 to 1.53)	0.99 (0.67 to 1.46)	S-RTearly	.
5	1.28 (0.60 to 2.74)	1.31 (1.05 to 1.63)	1.20 (0.91 to 1.58)	1.21 (0.80 to 1.82)	S-RT + consolidation

Forest plot L-CRT-1 was used as a common comparator. RRs above 1 favor the strategy over the common comparator (L-CRT1).

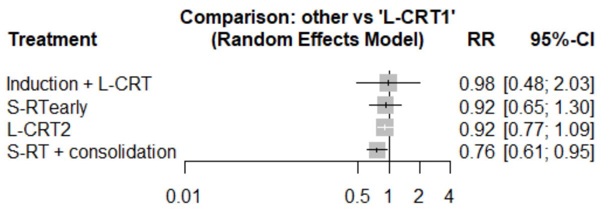

Assessment of heterogeneity and consistency

Global heterogeneity

We interpreted tau^2 as follows: heterogeneity low with tau^2≤0.010, moderate with 0.010<tau^2≤0.242, high with tau^2>0.242, and I^2 statistics as follows: not important (0%-40%), moderate (30%-60%), substantial (50%-90%), considerable (75%-100%).

© 2024 Turri G et al. *JAMA Network Open*.

```
tau^2= 0 ; tau= 0

I^2= 0 % ( NA % to NA %)
```

Consistency: global approach

Q statistic to assess consistency under the assumption of a full design-by-treatment interaction random effects model

```
Q statistics to assess homogeneity / consistency

                    Q df p-value
Total            0.06  1  0.8056
Within designs   0.06  1  0.8056
Between designs  0.00  0      --

Design-specific decomposition of within-designs Q statistic

       Design    Q df p-value
L-CRT1:L-CRT2 0.06  1  0.8056

Q statistic to assess consistency under the assumption of a full design-by-treatment interaction random effects model

                   Q df p-value tau.within tau2.within
Between designs 0.00  0      --          0           0
```

Consistency: local approach

```
Separate indirect from direct evidence (SIDE) using back-calculation method

Random effects model:

comparison k nma direct indir. RoR z p-value

Legend:
 comparison - Treatment comparison
 k          - Number of studies providing direct evidence
 nma        - Estimated treatment effect (RR) in network meta-analysis
 direct     - Estimated treatment effect (RR) derived from direct evidence
 indir.     - Estimated treatment effect (RR) derived from indirect evidence
 RoR        - Ratio of Ratios (direct versus indirect)
 z          - z-value of test for disagreement (direct versus indirect)
 p-value    - p-value of test for disagreement (direct versus indirect)
```

Netrank Higher p-score values indicate higher ranking of treatments

	P-score
L-CRT1	0.7573
Induction + L-CRT	0.5898
S-RTearly	0.5232
L-CRT2	0.4910
S-RT + consolidation	0.1387

© 2024 Turri G et al. *JAMA Network Open*.

eAppendix 23. Disease-Free Survival at 3 years

Characteristics of the network

Number of treatments:

```
7
```

Number of studies:

```
13
```

Number of individuals included:

```
7409
```

Number of individuals randomized to each treatment:

	Treatment name	N. individuals randomized
1	CHT	165
2	Induction + L-CRT	387
3	L-CRT + consolidation	210
4	L-CRT1	3156
5	L-CRT2	2395
6	S-RT + consolidation	1021
7	S-RTdelayed	75

Pairwise meta-analysis
Risk ratios above 1 favor the first treatment of the comparison.

© 2024 Turri G et al. *JAMA Network Open*.

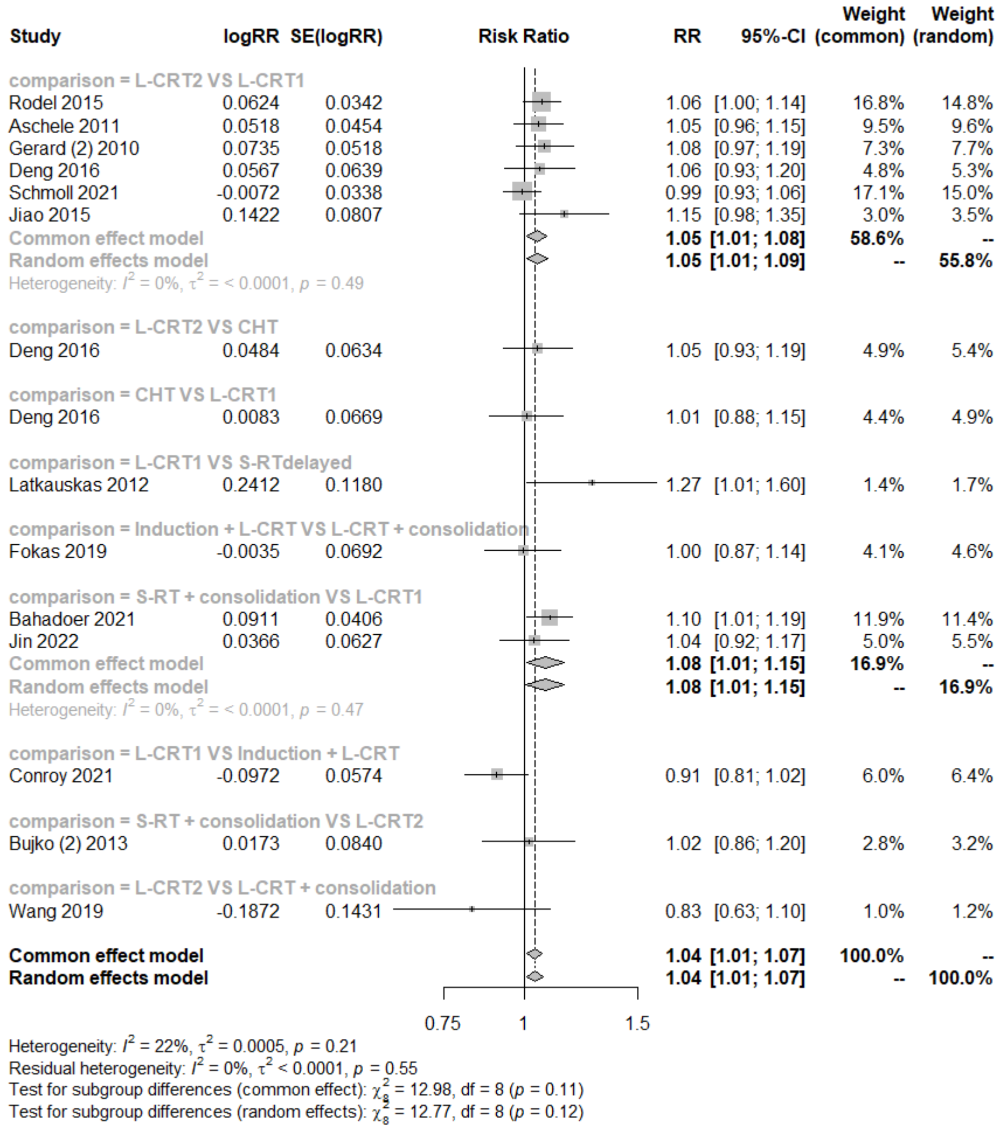

Network map The thickness of lines is proportional to the number of studies comparing the two treatments and the size of circles is proportional to the number of individuals for each treatment.

© 2024 Turri G et al. *JAMA Network Open*.

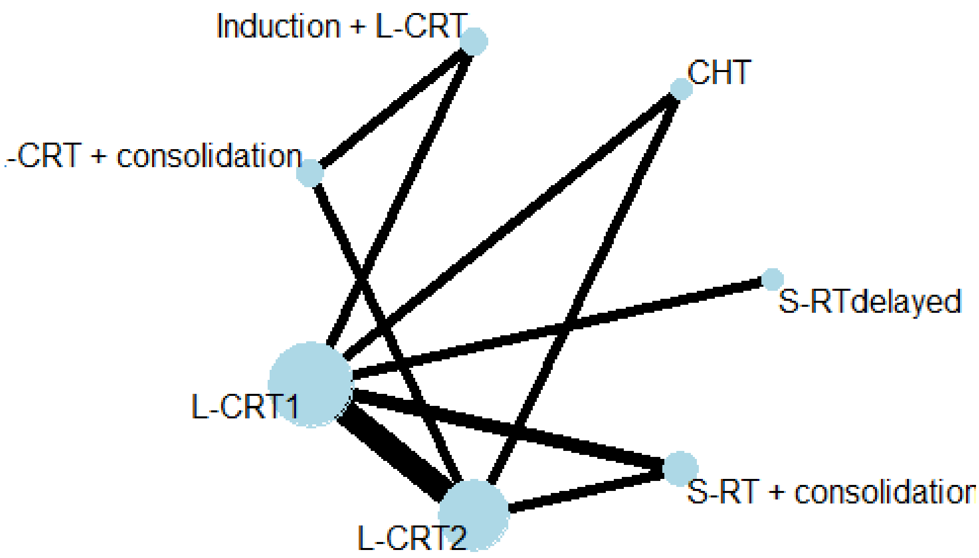

Netleague table Relative risks (RRs) and 95% confidence intervals (CIs) are reported. Results of the network meta-analysis are reported in the lower left part of the table and results from the pairwise meta-analysis are reported in the upper right part of the table. RRs higher than 1 favor the column-defining treatment.

	V1	V2	V3	V4	V5	V6	V7
1	CHT	.	.	1.01 (0.88 to 1.15)	0.95 (0.84 to 1.08)	.	.
2	0.90 (0.77 to 1.04)	Induction + L-CRT	1.00 (0.87 to 1.14)	1.10 (0.98 to 1.23)	.	.	.
3	0.87 (0.72 to 1.05)	0.97 (0.86 to 1.10)	L-CRT + consolidation	.	1.21 (0.91 to 1.60)	.	.
4	1.00 (0.89 to 1.12)	1.12 (1.01 to 1.24)	1.15 (0.99 to 1.33)	L-CRT1	0.96 (0.92 to 0.99)	0.93 (0.87 to 0.99)	1.27 (1.01 to 1.60)
5	0.96 (0.86 to 1.07)	1.07 (0.96 to 1.20)	1.10 (0.94 to 1.28)	0.96 (0.92 to 0.99)	L-CRT2	0.98 (0.83 to 1.16)	.
6	0.93 (0.82 to 1.06)	1.04 (0.92 to 1.18)	1.07 (0.91 to 1.25)	0.93 (0.87 to 0.99)	0.97 (0.91 to 1.04)	S-RT + consolidation	.
7	1.27 (0.99 to 1.65)	1.42 (1.10 to 1.84)	1.46 (1.11 to 1.92)	1.27 (1.01 to 1.60)	1.33 (1.05 to 1.68)	1.37 (1.08 to 1.74)	S-RTdelayed

Forest plot L-CRT-1 was used as a common comparator. RRs above 1 favor the strategy over the common comparator (L-CRT1).

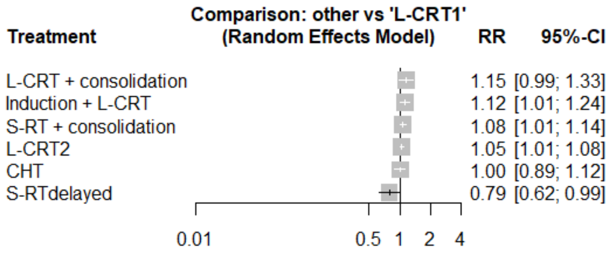

Assessment of heterogeneity and consistency

Global heterogeneity

We interpreted tau^2 as follows: heterogeneity low with tau^2≤0.010, moderate with 0.010<tau^2≤0.242, high with tau^2>0.242, and I^2 statistics as follows: not important (0%-40%), moderate (30%-60%), substantial (50%-90%), considerable (75%-100%).

© 2024 Turri G et al. *JAMA Network Open*.

tau^2= 0 ; tau= 0

I^2= 0 % (0 % to 64.8 %)

Consistency: global approach

Q statistic to assess consistency under the assumption of a full design-by-treatment interaction random effects model

Q statistics to assess homogeneity / consistency

	Q	df	p-value
Total	5.60	8	0.6917
Within designs	4.94	5	0.4228
Between designs	0.66	3	0.8829

Design-specific decomposition of within-designs Q statistic

Design	Q	df	p-value
L-CRT1:L-CRT2	4.41	4	0.3532
L-CRT1:S-RT + consolidation	0.53	1	0.4654

Between-designs Q statistic after detaching of single designs

Detached design	Q	df	p-value
Induction + L-CRT:L-CRT + consolidation	0.05	2	0.9742
L-CRT + consolidation:L-CRT2	0.05	2	0.9742
L-CRT1:Induction + L-CRT	0.05	2	0.9742
L-CRT1:L-CRT2	0.66	2	0.7195
L-CRT1:S-RT + consolidation	0.64	2	0.7276
L-CRT2:S-RT + consolidation	0.64	2	0.7276

Q statistic to assess consistency under the assumption of a full design-by-treatment interaction random effects model

	Q	df	p-value	tau.within	tau2.within
Between designs	0.66	3	0.8829	0	0

Consistency: local approach

Separate indirect from direct evidence (SIDE) using back-calculation method

Random effects model:

comparison	k	prop	nma	direct	indir.	RoR	z	p-value
L-CRT1:CHT	1	0.74	1.00	0.99	1.02	0.97	-0.20	0.8408
CHT:L-CRT2	1	0.82	0.96	0.95	0.98	0.97	-0.20	0.8408
Induction + L-CRT:L-CRT + consolidation	1	0.83	0.97	1.00	0.87	1.14	0.78	0.4362
L-CRT1:Induction + L-CRT	1	0.89	0.89	0.91	0.79	1.14	0.78	0.4362
L-CRT + consolidation:L-CRT2	1	0.29	1.10	1.21	1.06	1.14	0.78	0.4362
L-CRT1:L-CRT2	6	0.95	0.96	0.96	0.97	0.98	-0.24	0.8087
L-CRT1:S-RT + consolidation	2	0.86	0.93	0.93	0.94	0.99	-0.15	0.8804
L-CRT2:S-RT + consolidation	1	0.17	0.97	0.98	0.97	1.01	0.15	0.8804

Legend:
comparison - Treatment comparison
k - Number of studies providing direct evidence
prop - Direct evidence proportion
nma - Estimated treatment effect (RR) in network meta-analysis
direct - Estimated treatment effect (RR) derived from direct evidence
indir. - Estimated treatment effect (RR) derived from indirect evidence
RoR - Ratio of Ratios (direct versus indirect)

© 2024 Turri G et al. *JAMA Network Open*.

z	- z-value of test for disagreement (direct versus indirect)
p-value	- p-value of test for disagreement (direct versus indirect)

Netrank Higher p-score values indicate higher ranking of treatments

	P-score
L-CRT + consolidation	0.8687
Induction + L-CRT	0.8106
S-RT + consolidation	0.6872
L-CRT2	0.5319
CHT	0.3326
L-CRT1	0.2568
S-RTdelayed	0.0121

© 2024 Turri G et al. *JAMA Network Open*.

eAppendix 24. Disease-Free Survival at 5 years

Characteristics of the network

Number of treatments:

```
6
```

Number of studies:

```
7
```

Number of individuals included:

```
5302
```

Number of individuals randomized to each treatment:

	Treatment name	N. individuals randomized
1	CHT	585
2	Induction + L-CRT	54
3	L-CRT1	2585
4	L-CRT2	1249
5	L-RT	367
6	S-RT + consolidation	462

Pairwise meta-analysis
Risk ratios above 1 favor the first treatment of the comparison.

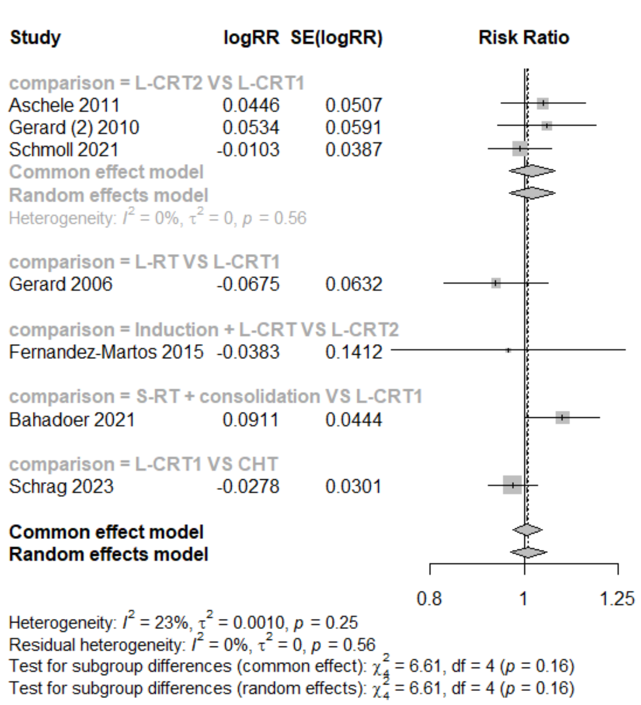

Network map The thickness of lines is proportional to the number of studies comparing the two treatments and the size of circles is proportional to the number of individuals for each treatment.

© 2024 Turri G et al. *JAMA Network Open*.

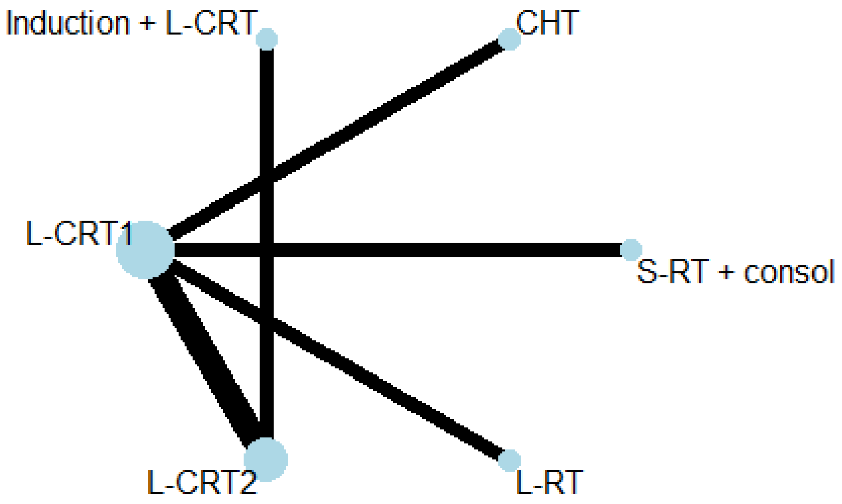

Netleague table Relative risks (RRs) and 95% confidence intervals (CIs) are reported. Results of the network meta-analysis are reported in the lower left part of the table and results from the pairwise meta-analysis are reported in the upper right part of the table. RRs higher than 1 favor the column-defining treatment.

	V1	V2	V3	V4	V5	V6
1	CHT	.	1.03 (0.97 to 1.09)	.	.	.
2	1.05 (0.79 to 1.40)	Induction + L-CRT	.	0.96 (0.73 to 1.27)	.	.
3	1.03 (0.97 to 1.09)	0.98 (0.74 to 1.30)	L-CRT1	0.98 (0.93 to 1.03)	1.07 (0.95 to 1.21)	0.91 (0.84 to 1.00)
4	1.01 (0.93 to 1.09)	0.96 (0.73 to 1.27)	0.98 (0.93 to 1.03)	L-CRT2	.	.
5	1.10 (0.96 to 1.26)	1.05 (0.77 to 1.43)	1.07 (0.95 to 1.21)	1.09 (0.95 to 1.25)	L-RT	.
6	0.94 (0.84 to 1.04)	0.90 (0.67 to 1.20)	0.91 (0.84 to 1.00)	0.93 (0.84 to 1.03)	0.85 (0.73 to 0.99)	S-RT + consolidation

Forest plot L-CRT-1 was used as a common comparator. RRs above 1 favor the strategy over the common comparator (L-CRT1).

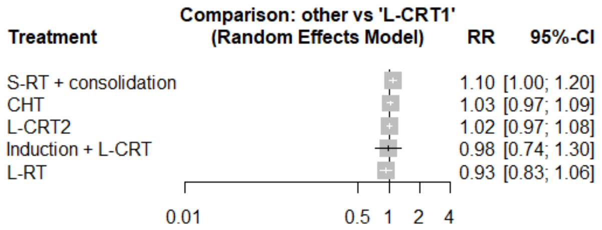

Assessment of heterogeneity and consistency

Global heterogeneity

We interpreted tau^2 as follows: heterogeneity low with tau^2≤0.010, moderate with 0.010<tau^2≤0.242, high with tau^2>0.242, and I^2 statistics as follows: not important (0%-40%), moderate (30%-60%), substantial (50%-90%), considerable (75%-100%).

© 2024 Turri G et al. *JAMA Network Open*.

tau^2= 0 ; tau= 0

I^2= 0 % (0 % to 89.6 %)

Consistency: global approach

Q statistic to assess consistency under the assumption of a full design-by-treatment interaction random effects model

Q statistics to assess homogeneity / consistency

	Q	df	p-value
Total	1.17	2	0.5581
Within designs	1.17	2	0.5581
Between designs	0.00	0	--

Design-specific decomposition of within-designs Q statistic

Design	Q	df	p-value
L-CRT1:L-CRT2	1.17	2	0.5581

Q statistic to assess consistency under the assumption of a full design-by-treatment interaction random effects model

	Q	df	p-value	tau.within	tau2.within
Between designs	0.00	0	--	0	0

Consistency: local approach

Separate indirect from direct evidence (SIDE) using back-calculation method

Random effects model:

comparison k nma direct indir. RoR z p-value

Legend:

comparison - Treatment comparison
k - Number of studies providing direct evidence
nma - Estimated treatment effect (RR) in network meta-analysis
direct - Estimated treatment effect (RR) derived from direct evidence
indir. - Estimated treatment effect (RR) derived from indirect evidence
RoR - Ratio of Ratios (direct versus indirect)
z - z-value of test for disagreement (direct versus indirect)
p-value - p-value of test for disagreement (direct versus indirect)

Netrank Higher p-score values indicate higher ranking of treatments

	P-score
S-RT + consolidation	0.9049
CHT	0.6128
L-CRT2	0.5523
Induction + L-CRT	0.4135
L-CRT1	0.3698
L-RT	0.1465

© 2024 Turri G et al. *JAMA Network Open*.

eAppendix 25. Overall survival at 3 years

Characteristics of the network

Number of treatments:

```
7
```

Number of studies:

```
11
```

Number of individuals included:

```
5576
```

Number of individuals randomized to each treatment:

	Treatment name	N. individuals randomized
1	CHT	165
2	Induction + L-CRT	387
3	L-CRT + consolidation	210
4	L-CRT1	2232
5	L-CRT2	1486
6	S-RT + consolidation	1021
7	S-RTdelayed	75

Pairwise meta-analysis
Risk ratios above 1 favor the first treatment of the comparison.

© 2024 Turri G et al. *JAMA Network Open*.

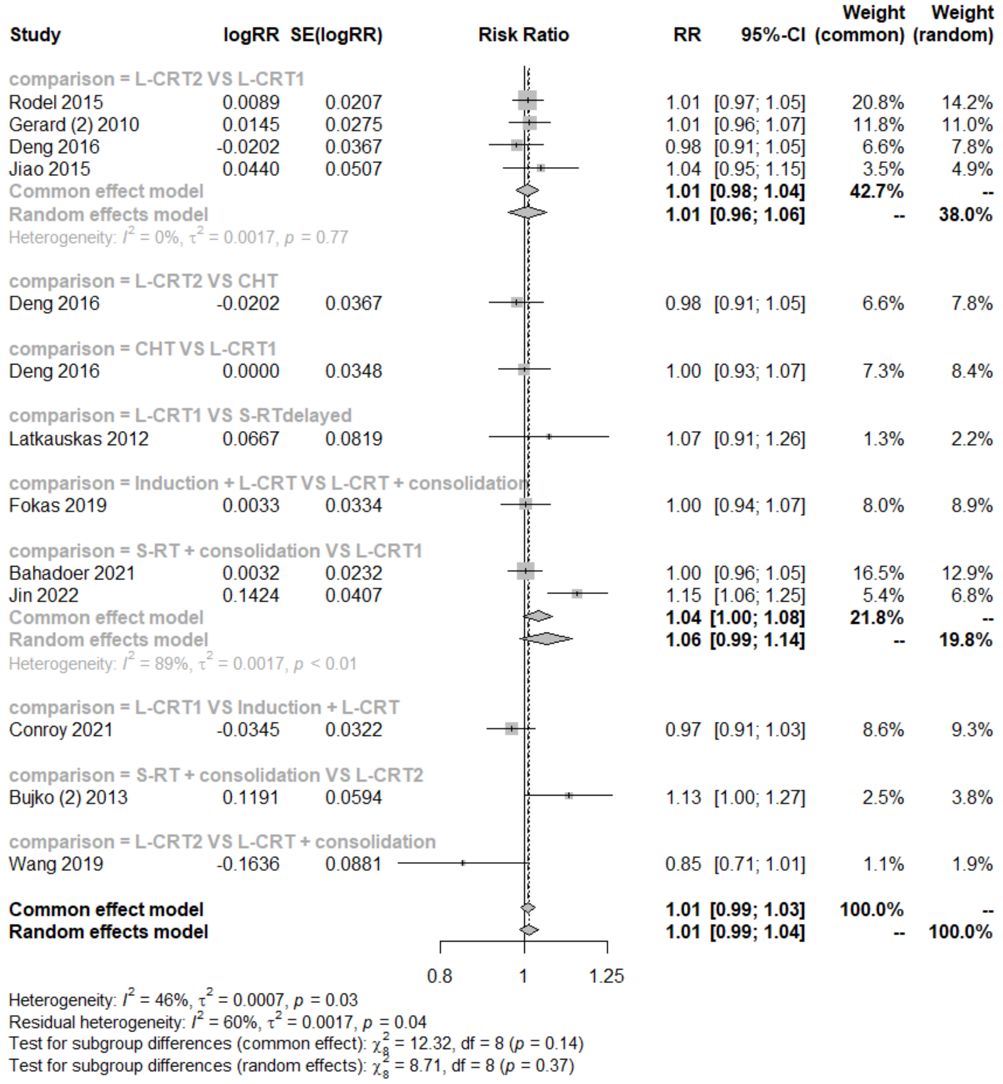

Network map The thickness of lines is proportional to the number of studies comparing the two treatments and the size of circles is proportional to the number of individuals for each treatment.

© 2024 Turri G et al. *JAMA Network Open*.

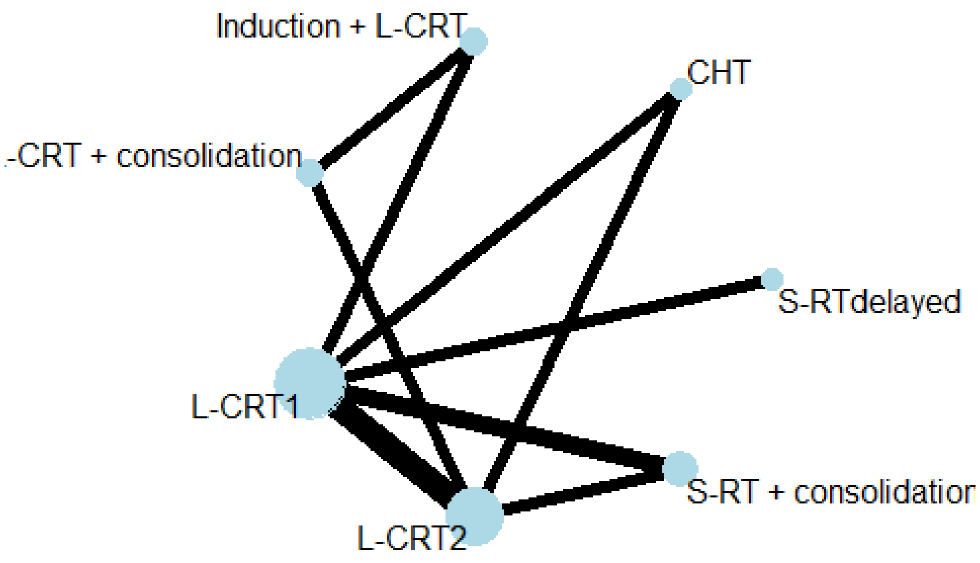

Netleague table Relative risks (RRs) and 95% confidence intervals (CIs) are reported. Results of the network meta-analysis are reported in the lower left part of the table and results from the pairwise meta-analysis are reported in the upper right part of the table. RRs higher than 1 favor the column-defining treatment.

	V1	V2	V3	V4	V5	V6	V7
1	CHT	.	.	1.00 (0.90 to 1.11)	1.02 (0.92 to 1.14)	.	.
2	0.95 (0.84 to 1.08)	Induction + L-CRT	1.00 (0.91 to 1.11)	1.04 (0.94 to 1.14)	.	.	.
3	0.93 (0.81 to 1.08)	0.98 (0.89 to 1.07)	L-CRT + consolidation	.	1.18 (0.97 to 1.42)	.	.
4	1.01 (0.92 to 1.11)	1.06 (0.97 to 1.16)	1.08 (0.96 to 1.21)	L-CRT1	0.99 (0.94 to 1.04)	0.94 (0.88 to 1.01)	1.07 (0.89 to 1.28)
5	1.01 (0.92 to 1.11)	1.06 (0.96 to 1.17)	1.08 (0.96 to 1.22)	1.00 (0.96 to 1.05)	L-CRT2	0.89 (0.77 to 1.02)	.
6	0.94 (0.84 to 1.05)	0.99 (0.88 to 1.10)	1.01 (0.88 to 1.15)	0.93 (0.87 to 0.99)	0.93 (0.86 to 1.00)	S-RT + consolidation	.
7	1.08 (0.88 to 1.32)	1.13 (0.93 to 1.38)	1.16 (0.93 to 1.43)	1.07 (0.89 to 1.28)	1.07 (0.89 to 1.28)	1.15 (0.95 to 1.39)	S-RTdelayed

Forest plot L-CRT-1 was used as a common comparator. RRs above 1 favor the strategy over the common comparator (L-CRT1).

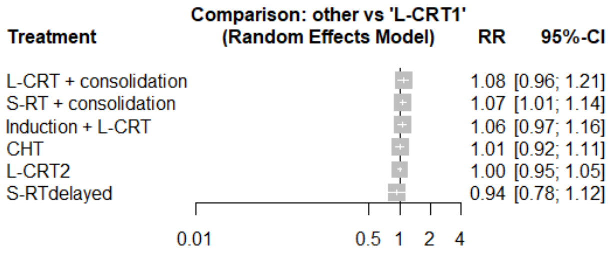

Assessment of heterogeneity and consistency

Global heterogeneity

We interpreted tau^2 as follows: heterogeneity low with tau^2≤0.010, moderate with 0.010<tau^2≤0.242, high with tau^2>0.242, and I^2 statistics as follows: not important (0%-40%), moderate (30%-60%), substantial (50%-90%), considerable (75%-100%).

© 2024 Turri G et al. *JAMA Network Open*.

tau^2= 0.0016 ; tau= 0.0401

I^2= 56.51 % (0 % to 81.28 %)

Consistency: global approach

Q statistic to assess consistency under the assumption of a full design-by-treatment interaction random effects model

Q statistics to assess homogeneity / consistency

	Q	df	p-value
Total	13.80	6	0.0320
Within designs	9.24	3	0.0262
Between designs	4.55	3	0.2074

Design-specific decomposition of within-designs Q statistic

Design	Q	df	p-value
L-CRT1:L-CRT2	0.41	2	0.8140
L-CRT1:S-RT + consolidation	8.83	1	0.0030

Between-designs Q statistic after detaching of single designs

Detached design	Q	df	p-value
Induction + L-CRT:L-CRT + consolidation	2.71	2	0.2584
L-CRT + consolidation:L-CRT2	2.71	2	0.2584
L-CRT1:Induction + L-CRT	2.71	2	0.2584
L-CRT1:L-CRT2	1.57	2	0.4564
L-CRT1:S-RT + consolidation	2.71	2	0.2583
L-CRT2:S-RT + consolidation	2.71	2	0.2583

Q statistic to assess consistency under the assumption of a full design-by-treatment interaction random effects model

	Q	df	p-value	tau.within	tau2.within
Between designs	2.06	3	0.5604	0.0446	0.0020

Consistency: local approach

Separate indirect from direct evidence (SIDE) using back-calculation method

Random effects model:

comparison	k	prop	nma	direct	indir.	RoR	z	p-value
L-CRT1:CHT	1	0.81	0.99	1.00	0.96	1.05	0.36	0.7159
CHT:L-CRT2	1	0.78	1.01	1.02	0.98	1.04	0.36	0.7159
Induction + L-CRT:L-CRT + consolidation	1	0.82	0.98	1.00	0.88	1.14	1.09	0.2743
L-CRT1:Induction + L-CRT	1	0.83	0.94	0.97	0.84	1.14	1.09	0.2743
L-CRT + consolidation:L-CRT2	1	0.39	1.08	1.18	1.03	1.14	1.09	0.2743
L-CRT1:L-CRT2	4	0.87	1.00	0.99	1.09	0.91	-1.28	0.1998
L-CRT1:S-RT + consolidation	2	0.82	0.93	0.94	0.88	1.07	0.76	0.4468
L-CRT2:S-RT + consolidation	1	0.27	0.93	0.89	0.95	0.94	-0.76	0.4468

Legend:
comparison - Treatment comparison
k - Number of studies providing direct evidence
prop - Direct evidence proportion
nma - Estimated treatment effect (RR) in network meta-analysis
direct - Estimated treatment effect (RR) derived from direct evidence
indir. - Estimated treatment effect (RR) derived from indirect evidence
RoR - Ratio of Ratios (direct versus indirect)

© 2024 Turri G et al. *JAMA Network Open*.

z	- z-value of test for disagreement (direct versus indirect)
p-value	- p-value of test for disagreement (direct versus indirect)

Netrank Higher p-score values indicate higher ranking of treatments

	P-score
S-RT + consolidation	0.7965
L-CRT + consolidation	0.7953
Induction + L-CRT	0.6967
CHT	0.4112
L-CRT1	0.3256
L-CRT2	0.3090
S-RTdelayed	0.1657

© 2024 Turri G et al. *JAMA Network Open*.

eAppendix 26. Overall survival at 5 years

Characteristics of the network

Number of treatments:

```
7
```

Number of studies:

```
8
```

Number of individuals included:

```
5625
```

Number of individuals randomized to each treatment:

	Treatment name	N. individuals randomized
1	CHT	585
2	Induction + L-CRT	54
3	L-CRT1	2747
4	L-CRT2	1249
5	L-RT	367
6	S-RT + consolidation	462
7	S-RTearly	161

Pairwise meta-analysis
Risk ratios above 1 favor the first treatment of the comparison.

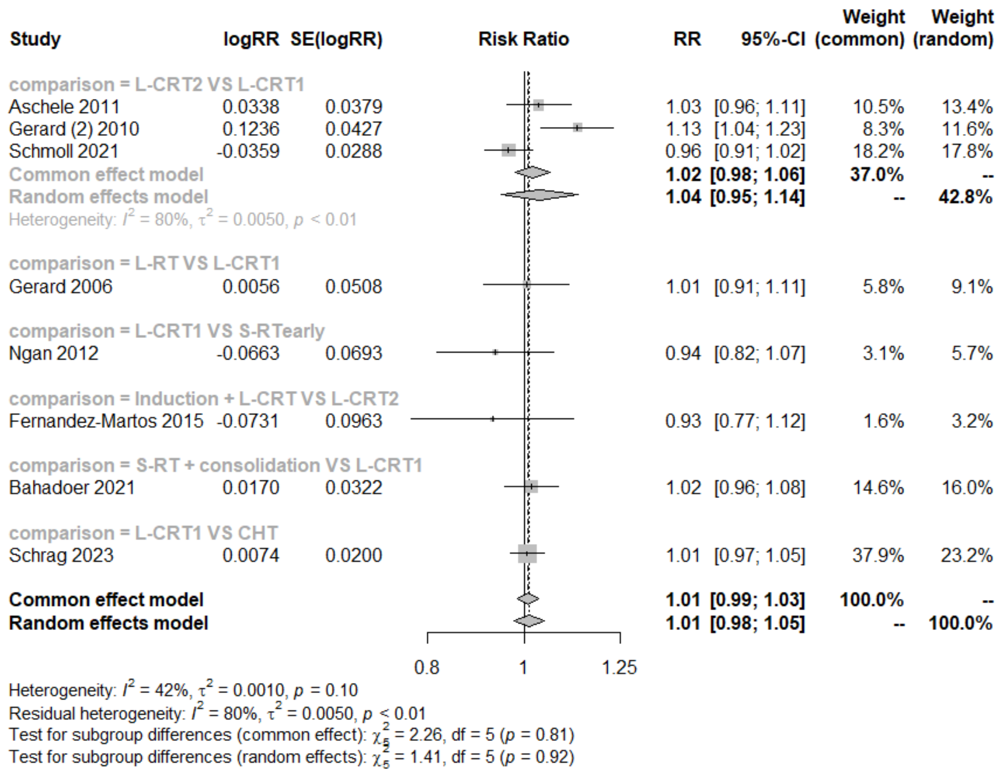

Network map The thickness of lines is proportional to the number of studies comparing the two treatments and the size of circles is proportional to the number of individuals for each treatment.

© 2024 Turri G et al. *JAMA Network Open*.

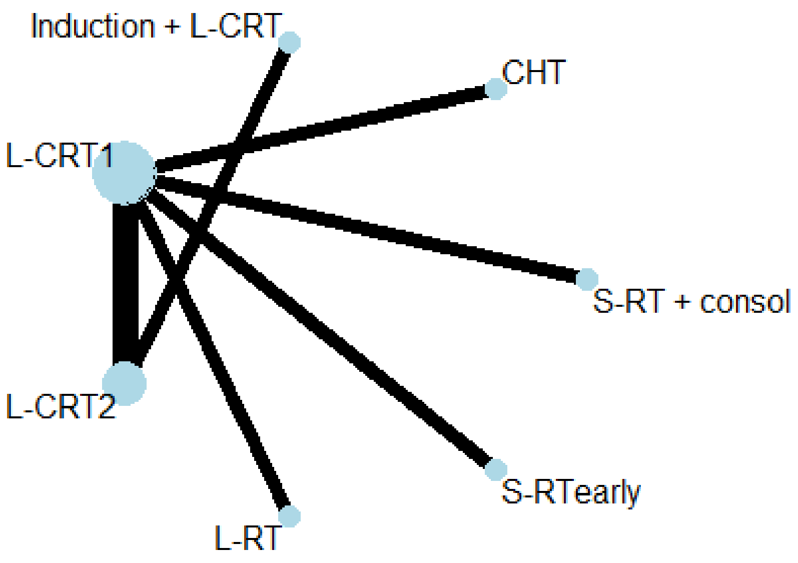

Netleague table Relative risks (RRs) and 95% confidence intervals (CIs) are reported. Results of the network meta-analysis are reported in the lower left part of the table and results from the pairwise meta-analysis are reported in the upper right part of the table. RRs higher than 1 favor the column-defining treatment.

	V1	V2	V3	V4	V5	V6	V7
1	CHT	.	0.99 (0.86 to 1.15)	.	.	.	.
2	1.03 (0.77 to 1.38)	Induction + L-CRT	.	0.93 (0.74 to 1.18)	.	.	.
3	0.99 (0.86 to 1.15)	0.96 (0.75 to 1.24)	L-CRT1	0.96 (0.88 to 1.06)	0.99 (0.84 to 1.18)	0.94 (0.77 to 1.14)	0.98 (0.84 to 1.15)
4	0.96 (0.81 to 1.14)	0.93 (0.74 to 1.18)	0.96 (0.88 to 1.06)	L-CRT2	.	.	.
5	0.99 (0.79 to 1.24)	0.96 (0.71 to 1.30)	0.99 (0.84 to 1.18)	1.03 (0.85 to 1.25)	L-RT	.	.
6	0.93 (0.73 to 1.18)	0.90 (0.66 to 1.24)	0.94 (0.77 to 1.14)	0.97 (0.78 to 1.20)	0.94 (0.73 to 1.22)	S-RTearly	.
7	0.98 (0.79 to 1.20)	0.95 (0.71 to 1.27)	0.98 (0.84 to 1.15)	1.02 (0.85 to 1.22)	0.99 (0.79 to 1.24)	1.05 (0.82 to 1.35)	S-RT + consolidation

Forest plot L-CRT-1 was used as a common comparator. RRs above 1 favor the strategy over the common comparator (L-CRT1).

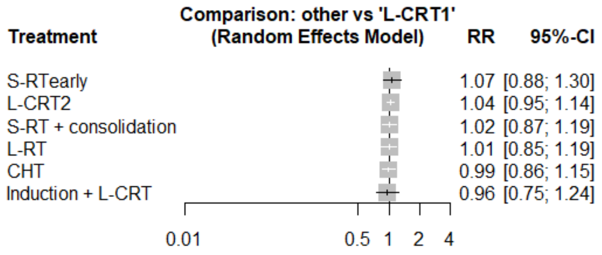

Assessment of heterogeneity and consistency

Global heterogeneity

We interpreted tau^2 as follows: heterogeneity low with tau^2≤0.010, moderate with 0.010<tau^2≤0.242, high with tau^2>0.242, and I^2 statistics as follows: not important (0%-40%), moderate (30%-60%), substantial (50%-90%), considerable (75%-100%).

© 2024 Turri G et al. *JAMA Network Open*.

tau^2= 0.0051 ; tau= 0.0712

I^2= 79.58 % (35.15 % to 93.57 %)

Consistency: global approach

Q statistic to assess consistency under the assumption of a full design-by-treatment interaction random effects model

Q statistics to assess homogeneity / consistency

	Q	df	p-value
Total	9.80	2	0.0075
Within designs	9.80	2	0.0075
Between designs	0.00	0	--

Design-specific decomposition of within-designs Q statistic

Design	Q	df	p-value
L-CRT1:L-CRT2	9.80	2	0.0075

Q statistic to assess consistency under the assumption of a full design-by-treatment interaction random effects model

	Q	df	p-value	tau.within	tau2.within
Between designs	0.00	0	--	0.0712	0.0051

Consistency: local approach

Separate indirect from direct evidence (SIDE) using back-calculation method

Random effects model:

comparison k nma direct indir. RoR z p-value

Legend:
comparison - Treatment comparison
k - Number of studies providing direct evidence
nma - Estimated treatment effect (RR) in network meta-analysis
direct - Estimated treatment effect (RR) derived from direct evidence
indir. - Estimated treatment effect (RR) derived from indirect evidence
RoR - Ratio of Ratios (direct versus indirect)
z - z-value of test for disagreement (direct versus indirect)
p-value - p-value of test for disagreement (direct versus indirect)

Netrank Higher p-score values indicate higher ranking of treatments

	P-score
S-RTearly	0.6908
L-CRT2	0.6342
S-RT + consolidation	0.5198
L-RT	0.4734
L-CRT1	0.4180
CHT	0.4144
Induction + L-CRT	0.3494

© 2024 Turri G et al. *JAMA Network Open*.

eReferences

1. Higgins JPT, Thomas J, Chandler J. *Cochrane Handbook for Systematic Reviews of Interventions, 2nd Edition*. (Wiley, ed.). Chichester; 2019. https://training.cochrane.org/handbook/current. Accessed March 7, 2023.

2. Cook RJ, Sackett DL. The number needed to treat: a clinically useful measure of treatment effect. *BMJ*. 1995;310(6977):452. doi:10.1136/BMJ.310.6977.452

3. Veroniki AA, Bender R, Glasziou P, Straus SE, Tricco AC. The number needed to treat in pairwise and network meta-analysis and its graphical representation. *J Clin Epidemiol*. 2019;111:11-22. doi:10.1016/J.JCLINEPI.2019.03.007

4. Lu G, Ades AE. Combination of direct and indirect evidence in mixed treatment comparisons. *Stat Med*. 2004;23(20):3105-3124. doi:10.1002/sim.1875

5. Shim SR, Kim SJ, Lee J, Rücker G. Network meta-analysis: application and practice using R software. *Epidemiol Health*. 2019;41:e2019013. doi:10.4178/EPIH.E2019013

6. Higgins J, Thomas J, Chandler J, et al. *Cochrane Handbook for Systematic Reviews of Interventions Version 6.3 (Updated February 2022)*.; 2022. www.training.cochrane.org/handbook.

7. Cipriani A, Higgins JPT, Geddes JR, Salanti G. Conceptual and technical challenges in network meta-analysis. *Ann Intern Med*. 2013;159(2):130-137. doi:10.7326/0003-4819-159-2-201307160-00008

8. Ostuzzi G, Schneider-Thoma J, Tedeschi F, Leucht S, Barbui C. Crossroads of methodological choices in research synthesis: insights from two network meta-analyses on preventing relapse in schizophrenia. *BMJ Ment Heal*. 2023;26(1):e300677. doi:10.1136/BMJMENT-2023-300677

9. Ostuzzi G, Bertolini F, Tedeschi F, et al. Oral and long-acting antipsychotics for relapse prevention in schizophrenia-spectrum disorders: a network meta-analysis of 92 randomized trials including 22,645 participants. *World Psychiatry*. 2022;21(2):295-307. doi:10.1002/wps.20972

10. Bucher HC, Guyatt GH, Griffith LE, Walter SD. The results of direct and indirect treatment comparisons in meta-analysis of randomized controlled trials. *J Clin Epidemiol*. 1997;50(6):683-691. doi:10.1016/S0895-4356(97)00049-8

11. Higgins JPT, Jackson D, Barrett JK, Lu G, Ades AE, White IR. Consistency and inconsistency in network meta-analysis: concepts and models for multi-arm studies. *Res Synth Methods*. 2012;3(2):98-110. doi:10.1002/JRSM.1044

12. White IR, Barrett JK, Jackson D, Higgins JPT. Consistency and inconsistency in network meta-analysis: model estimation using multivariate meta-regression. *Res Synth Methods*. 2012;3(2):111-125. doi:10.1002/JRSM.1045

13. Chaimani A, Higgins JPT, Mavridis D, Spyridonos P, Salanti G. Graphical tools for network meta-analysis in STATA. *PLoS One*. 2013;8(10). doi:10.1371/JOURNAL.PONE.0076654

14. White IR. Network meta-analysis. *Stata J*. 2015;15(4):951-985. doi:10.1177/1536867x1501500403

15. Egger M, Smith GD, Schneider M, Minder C. Bias in meta-analysis detected by a simple, graphical test. *BMJ*. 1997;315(7109):629-634. doi:10.1136/BMJ.315.7109.629

16. Salanti G, Giovane C Del, Chaimani A, Caldwell DM, Higgins JPT. Evaluating the quality of evidence from a network meta-analysis. *PLoS One*. 2014;9(7). doi:10.1371/journal.pone.0099682

17. Nikolakopoulou A, Higgins JPT, Papakonstantinou T, et al. Cinema: An approach for assessing confidence in the results of a network meta-analysis. *PLoS Med*. 2020;17(4). doi:10.1371/JOURNAL.PMED.1003082

© 2024 Turri G et al. *JAMA Network Open*.
