# Supplementary material for: Treatment of Locally Advanced Rectal Cancer in the Era of Total Neoadjuvant Therapy: A Systematic Review and Network Meta-Analysis
Source: JAMA Netw Open. 2024 Jun 4;7(6):e2414702. doi: 10.1001/jamanetworkopen.2024.14702 (PMC11151159; doi:10.1001/jamanetworkopen.2024.14702)
Supplement: Supplement 2. — Data Sharing Statement [file jamanetwopen-e2414702-s002.pdf]

Data Sharing Statement

Turri. Treatment of Locally Advanced Rectal Cancer in the Era of Total Neoadjuvant Therapy. *JAMA Netw Open*. Published June 04, 2024. doi:10.1001/jamanetworkopen.2024.14702

Data
Data available: Yes
Data types: Other (please specify)
Additional Information: Data extracted from randomized clinical trials will be made available upon reasonable request to the corresponding author.
How to access data: Data extracted from randomized clinical trials will be made available upon reasonable request to the corresponding author.
When available: With publication

Supporting Documents
Document types: None

Additional Information
Who can access the data: Data extracted from randomized clinical trials will be made available upon reasonable request to the corresponding author.
Types of analyses: Data extracted from randomized clinical trials will be made available upon reasonable request to the corresponding author.
Mechanisms of data availability: Data extracted from randomized clinical trials will be made available upon reasonable request to the corresponding author.
